# Bi-directional nucleosome sliding by the Chd1 chromatin remodeler integrates intrinsic sequence-dependent and ATP-dependent nucleosome positioning

**DOI:** 10.1093/nar/gkad738

**Published:** 2023-09-20

**Authors:** Sangwoo Park, Giovanni B Brandani, Taekjip Ha, Gregory D Bowman

**Affiliations:** Department of Biophysics and Biophysical Chemistry, Johns Hopkins University School of Medicine, Baltimore, MD 21205, USA; Department of Biophysics, Graduate School of Science, Kyoto University, Japan; Department of Biophysics and Biophysical Chemistry, Johns Hopkins University School of Medicine, Baltimore, MD 21205, USA; Department of Biomedical Engineering, Johns Hopkins University, Baltimore, MD 21205, USA; Howard Hughes Medical Institute, Baltimore, MD 21205, USA; TC Jenkins Department of Biophysics, Johns Hopkins University, Baltimore, MD 21218, USA; TC Jenkins Department of Biophysics, Johns Hopkins University, Baltimore, MD 21218, USA

## Abstract

Chromatin remodelers use a helicase-type ATPase motor to shift DNA around the histone core. Although not directly reading out the DNA sequence, some chromatin remodelers exhibit a sequence-dependent bias in nucleosome positioning, which presumably reflects properties of the DNA duplex. Here, we show how nucleosome positioning by the Chd1 remodeler is influenced by local DNA perturbations throughout the nucleosome footprint. Using site-specific DNA cleavage coupled with next-generation sequencing, we show that nucleosomes shifted by Chd1 can preferentially localize DNA perturbations – poly(dA:dT) tracts, DNA mismatches, and single-nucleotide insertions – about a helical turn outside the Chd1 motor domain binding site, super helix location 2 (SHL2). This phenomenon occurs with both the Widom 601 positioning sequence and the natural +1 nucleosome sequence from the *Saccharomyces cerevisiae* SWH1 gene. Our modeling indicates that localization of DNA perturbations about a helical turn outward from SHL2 results from back-and-forth sliding due to remodeler action on both sides of the nucleosome. Our results also show that barrier effects from DNA perturbations can be extended by the strong phasing of nucleosome positioning sequences.

## Graphical Abstract

**Graphical Abstract:**
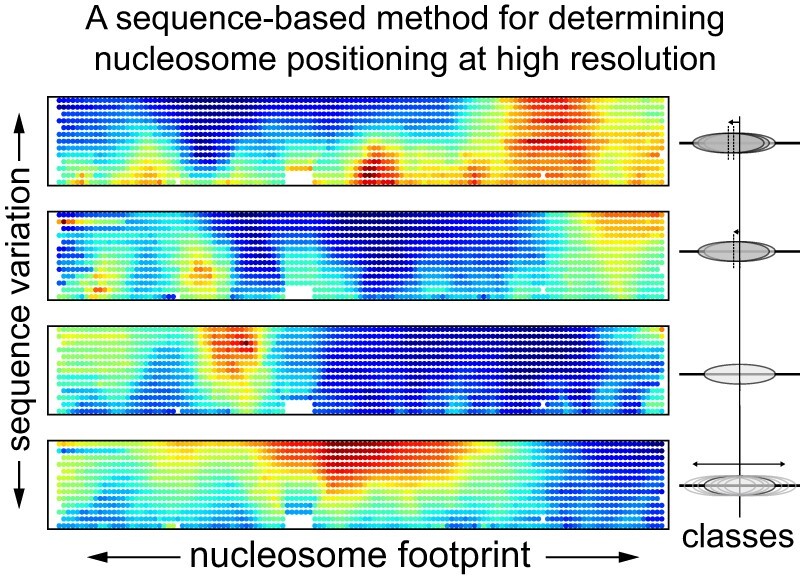

## INTRODUCTION

The nucleosome is the basic packaging unit of all eukaryotic genomes. Due to reduced accessibility of DNA as it wraps around the histone core, nucleosomes are inherently repressive (1). Although possessing some intrinsic dynamics, nucleosomes must be actively reorganized to change their positions, composition, and occupancy throughout the genome, which typically requires action of ATP-dependent chromatin remodeling enzymes (2–5).

Chd1 is a monomeric chromatin remodeler that can assemble and reposition nucleosomes into evenly spaced arrays (6–10). *In vivo*, Chd1 is associated with active transcription, where it is believed to be important in reestablishing the chromatin barrier after passage of RNA polymerase II (11–14). Like other chromatin remodelers, Chd1 engages with nucleosomal DNA with its ATPase motor ∼20 bp from the dyad, a site known as super helix location 2 (SHL2) (6–9). With its ATPase at this internal site, Chd1 shifts DNA unidirectionally toward the dyad in a step-wise cycle, which repositions nucleosomes along DNA (15). Although action at one SHL2 site is always unidirectional, Chd1 can shift nucleosomes back-and-forth by virtue of the 2-fold symmetry of the nucleosome, which provides an SHL2 site on each side of the dyad (often referred to as SHL-2 and SHL+2) (16).

Nucleosome sliding by Chd1 can be influenced by DNA sequence. *In vitro*, nucleosome sliding experiments are often performed using strong positioning sequences, such as the Widom 601 (17), which ensure that nucleosomes share the same initial placement on the DNA. The Widom 601 sequence is asymmetric, and notably has a different number of TpA (TA) dinucleotide steps on each side of the dyad. Chd1 has been shown to preferentially shift the nucleosome dyad toward the side with fewer TA steps, known as the TA-poor side of the 601 (18,19). With the introduction of long poly[dA:dT) tracts on the TA-poor side, the sliding direction reverses, with the dyad instead shifting toward the TA-rich side (18).

**Figure 1.**
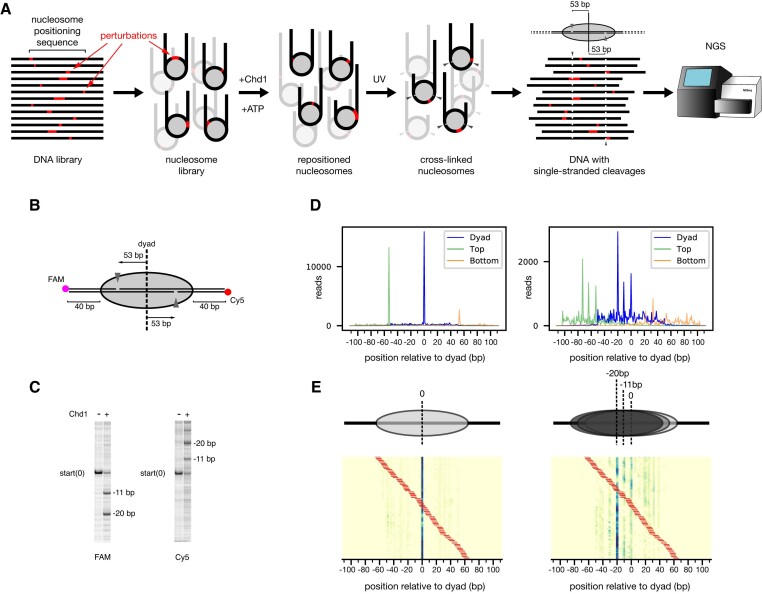
(**A**) Schematic workflow of Slide-seq. After synthesis of a DNA library, based on a nucleosome positioning sequence, the DNA is incorporated into nucleosomes, and the nucleosomes are then repositioned by the Chd1 remodeler. To determine nucleosome positions, DNA is site-specifically photo-cross-linked to histones, which cleaves the DNA backbone 53 bp from the dyad. Next-generation sequencing these DNA fragments reveals the locations of DNA cleavages and thus the positions of DNA on the histone core for each distinct sequence. (**B**) Schematic of the photo-cross-linking sites on the nucleosome. When histone H2B(S53C) is labeled with azido phenacyl bromide (APB), it cross-links to only one strand of the duplex on each side of the nucleosome, 53 bp from the dyad. **(C**) Example of cleavage products resulting from photo-cross-linking nucleosomes, before and after sliding by Chd1. Here, the photo-crosslinked DNA cleavage was visualized on a urea denaturing gel, scanned separately for FAM (top strand) and Cy5 (bottom strand). (**D**) Next-generation sequencing data revealing the location of DNA cleavage sides, before (left) and after sliding (right) by Chd1. Green peaks represent cleavage sites on the top strand, orange peaks on the bottom strand, and blue peaks are calculated dyad positions. The DNA sequence for this experiment was the canonical Widom 601 DNA. (**E**) An example heatmap from the 601 poly(dA:dT) library with 8 bp tract length, before (left) and after sliding (right) by Chd1. Tract location is indicated by the red bars. Cartoons above the heatmaps show the most populated nucleosome positions.

While these experiments showed that Chd1 activity is sensitive to the DNA sequence on the nucleosome, it remained unclear where in the nucleosome the sequence differences affected remodeler action. We therefore developed a sequencing-based approach (Slide-seq) to evaluate how variations in DNA alter the distribution of nucleosome positions (Figure 1). Using this pipeline, we found that poly(dA:dT) tract length and position strongly correlated with interference of nucleosome repositioning, with tracts as short as 3 bp influencing repositioning of Widom 601 nucleosomes by Chd1. Unexpectedly, repositioned nucleosomes showed poly(dA:dT) tracts enriched at SHL-3, about a helical turn away from the Chd1 binding site toward the nucleosome edge. Through biochemistry and simulations, we show that unidirectional nucleosome movement is most strongly blocked when DNA perturbations are located within the SHL-2 binding site. However, in Slide-seq experiments, when nucleosomes were allowed to shift back-and-forth, DNA perturbations such as mismatches and single-nucleotide insertions were found at SHL-3, similar to what was observed with poly(dA:dT) tracts. In addition to the Widom 601 sequence, we also tested nucleosome libraries derived from the sequence of the +1 nucleosome in the SWH1 gene, a natural positioning sequence in *S. cerevisiae*. With back-and-forth sliding, SWH1 +1 nucleosomes also showed mismatches and insertions at SHL+/-3, suggesting that this behavior may be a common response to remodeling by Chd1.

Through kinetic modeling and simulations, we demonstrate that strong nucleosome positioning sequences, such as the Widom 601 and SWH1 +1, can extend the barrier from DNA-based interference. With interference at one SHL2, the Chd1 remodeler preferentially acts at the opposite SHL2. This asymmetric action shifts interfering sequences from SHL2 back to SHL3, with strong phasing of the DNA sequence favoring a ∼10 bp shift. Thus, when capable of sliding back-and-forth, nucleosomes can accumulate elements that interfere with remodeler action outside the remodeler-targeted SHL2 sites. These results have implications for how strong positioning sequences that are naturally found *in vivo* coordinate with ATP-dependent nucleosome sliding.

## MATERIALS AND METHODS

### Nucleosome library reconstitution and biochemical assays

All DNA libraries were custom synthesized (Custom Array Inc.) and amplified by emulsion PCR (20) with unmodified or modified primers. The 601 nucleosome positioning sequence (17) and SWH1 +1 sequences were used as the base for all nucleosomes (Supplementary Table S1). For NGS experiments, the core nucleosome positioning sequence was flanked by 40 bp DNA on either side (40N40). To generate the mismatch and insertion/deletion libraries, a pool of single-stranded DNA was first prepared by amplifying with one 5′-phosphorylated primer and then treating with lambda exonuclease, which preferentially digests the phosphorylated strand. Duplex DNA was generated by heat annealing each pool of single-stranded DNA with the complementary strand of the unmodified positioning sequence (601 or SWH1 +1), and then purified using mini-prep columns (Qiagen). The DNA strands with abasic site were prepared by splint ligation of three DNA fragments purchased from Integrated DNA Technologies (IDT), with one oligo containing the 1,2-dideoxyribose modification, an abasic site analog. The ligated strands were purified on urea polyacrylamide gels (6%, 19:1 acrylamide:bis-acrylamide, 8 M urea) and annealed together to make double strand DNAs in specific top/bottom strand combinations. All nucleosomes were made using *Xenopus laevis* histones that contained H2B (S53C) for site-specific cross-linking with azido phenacyl bromide (APB). Nucleosome libraries were reconstituted by salt-gradient dialysis (21) using a 1:1.2 molar ratio of DNA to histone octamer.

#### Nucleosome sliding assays

All nucleosome sliding reactions were done with 150 nM nucleosome, 50 nM Chd1 (for Slide-seq experiments) or 50 nM nucleosome, 100 nM Chd1 (for individual sliding experiments) with 2 mM ATP in 1× SlideBuffer (20 mM Tris–HCl pH 7.5, 50 mM KCl, 5 mM MgCl_2_, 5% sucrose, 0.1 mg/ml BSA and 5 mM DTT). Reactions were carried out at room temperature and stopped with quench buffer (20 mM Tris–HCl pH 7.5, 50 mM KCl, 0.1mg/ml BSA, 5 mM DTT and 5 mM EDTA) after 5 min (for NGS experiments) or at indicated time points and with 1 μg/μl salmon sperm DNA (for native gel experiments). Although we did not perform time courses for Slide-seq experiments, they were carried out under the same conditions that we previously showed were sufficient to allow centering of 601 nucleosomes to reach steady state (22). While we expect that in many cases the DNA perturbations did not increase the time to steady state, even in cases where steady state was not achieved, the patterns we observed still reflect the changes in nucleosome distributions that arise from sliding rates affected by DNA perturbations. For gel-based sliding experiments, samples were resolved by native PAGE (6% acrylamide:bis-acrylamide, 60:1) at 4°C.

#### Histone-DNA photo-crosslinking and cleavage

After nucleosome sliding by Chd1, nucleosomal DNA was cross-linked to H2B(S53C) labeled with APB (23). Briefly, prior to nucleosome sliding reactions, nucleosomes were labelled with APB in the dark for 2 h and quenched with 5 mM DTT. After sliding, cross-links to DNA were induced by UV illumination for 15 seconds. DNA was processed by incubation at 70°C for 20 min, phenol chloroform extraction, and then ethanol precipitation. Crosslinked DNA was cleaved in 0.1 M NaOH at 90°C for 30 min and quenched with the same volume of 0.1 M HCl. The final fragmented DNA was collected through ethanol precipitation and could be visualized after separation in urea-polyacrylamide gels (8%, 19:1 acrylamide:bis-acrylamide, 8 M urea).

#### NGS library preparation and sequencing

A schematic of the library preparation and processing is given in Supplementary Figure S1. To preferentially sequence DNAs that were cleaved, a streptavidin pull-down was performed (Invitrogen T1 dynabeads), which removed both biotinylated 5′ end fragments as well as biotinylated uncleaved fragments. After ethanol precipitation of the supernatant, the cleaved single-stranded DNA was filled in to make duplex by Bst 2.0 warmstart polymerase (NEB) at 65°C, and the further cleaned up by AMPure XP beads (Beckman). The final double strand DNA fragments pool was ligated with adapters and amplified with Illumina index primers by using NEBNext Ultra II NGS library prep kit (NEB). The prepared NGS library was sequenced as 150 × 150 bp pair-end sequencing in the Miseq or Hiseq 2500.

### NGS library data analysis

First, sequencing reads were aligned to the Widom 601 or SWH1 sequences using Bowtie2 software (24). Using the alignment, sequence variations indicated the location of mismatches or insertions/deletions, and a premature truncation in the read indicated the location of cleavage sites. Data were discarded if variation in the 601 sequence, SWH1 sequence, or cleavage sites could not be identified. The final sort file, consisting of variant sequences with site-specific cleavage, contained 10–30% of total reads. With the two copies of H2B(S53C), cleavage occurs on both strand, each being 53 nt from the 5′ side of the dyad. Since the sequence variations and cleavage sites must be in the same read, only sequence variations located 3′ to the cleavage site can be detected. We used a linear regression model to combine data from both strands at each position to calculate the dyad position. In some cases, such as when the variation was 5′ to a cleavage site, the dyad was determined from one cleavage on one strand. Where noise was significant, the signal was boosted from a simple uniform subtraction of cleavage counts over position. The nucleosome positioning signal was defined as the sum of the cleavage counts at the top strand –53 bp and bottom strand + 53 bp locations. All data analysis was done by custom python scripts, which can be found on https://github.com/spark159/slide-seq.

#### NMF score map

For each sequence, nucleosome positioning signals were first normalized by total reads and converted into probabilities. Since nucleosome positioning data is nonnegative, we used nonnegative matrix factorization (NMF) to approximately decompose all positioning data of each library into basis patterns with corresponding weights. For each basis pattern, we mapped associated weight values onto the locations of perturbations on the nucleosomal DNA for each sequence. In the cases where multiple scores were assigned onto single locations due to the overlaps of perturbations from different sequences, the average score was used.

#### Clustering analysis

As the resemblance metric for nucleosome positioning data, the similarity score between two positioning probabilities was defined by exponential of negative Jensen-Shannon divergence of the probability pair. After computing all pair-wise similarity scores, all positioning data of libraries was clustered through the Spectral clustering algorithm. The cluster number was determined through trial and error as the minimal number that produced well isolated clusters.

#### KL-divergence maps

To quantify how much the nucleosome positioning signal deviated from the original positioning through perturbations, the Kullback–Leibler divergence (KL-divergence) metric was used for comparing perturbed positioning probabilities with respect to the original nucleosome positioning. Then, KL-divergence values were mapped on the locations of perturbation in the sequence. In cases where the same location had multiple KL-divergence values due to overlaps of perturbation from difference sequences, the averaged value was used.

### Molecular Dynamics

For molecular dynamics (MD) simulations, we employed the same coarse-grained (CG) nucleosome model previously used to investigate nucleosome sliding and remodeling (25–27). Within this model, the histone octamer is represented using 1 bead per amino acid according to the AICG2 + structure-based model (28,29). Unlike in previous studies, the nucleosomal DNA was represented using 3 beads per nucleotide (corresponding to base, phosphate and sugar groups) according to the recently developed sequence-dependent MADna model (30). This model has been carefully parametrized against extensive all-atom MD simulations, and it has been shown to reproduce critical futures of DNA elasticity that are not always well captured using other CG models. For comparison, we also re-ran part of our simulations with the previously used 3SPN.2C model (31) showing that our key findings are not affected by the specific DNA model employed. In our nucleosome model, proteins and DNA interact via excluded volume interactions, long-range Debye–Hückel electrostatics at the salt concentration of 300 mM, and short-range hydrogen bonds using a structure-based distance- and angle-dependent potential with a bond constant of 2.4 k_B_T (25). The nucleosome model used the 601 DNA sequence (17)starting from the conformation observed in the cryo-EM structure of the Chd1-nucleosome complex in the nucleotide-free state, with PDB id 7TN2 (7). All simulations were performed using the software GENESIS, where several popular CG potentials have been recently implemented (32).

For each considered sequence, we performed 100 independent MD runs starting from the same conformation based on PDB id 7TN2 and integrated the Langevin dynamics of the system at a temperature of 300 K for 10^6^ MD timesteps of 10 fs each. The conformations observed during the MD trajectories were saved every 1000 MD steps to study the stability of the initial +1 nt tracking strand defect at SHL2. The initial 601 nucleosome positioning was stable on the considered timescales. Nucleosomal DNA sliding was quantitatively analyzed using collective variables s_i_ tracking the progress of phosphate groups along the direction of the tracking strand backbone at the histone-DNA contact points with SHLs i = 2.5 and i = 1.5. This ‘phosphate progress’ s_i_ is initially equal to 0 nt; if DNA slides toward the dyad by 1 bp at the considered histone-DNA contact point (at SHL2.5 or SHL1.5), the phosphate progress will be close to +1 nt, while sliding in the opposite direction will bring the phosphate progress close to –1 nt. The size of the tracking strand defect at SHL2 is given by the difference between the phosphate progresses at SHL1.5 and SHL2.5 plus 1 nt, since the initial 7TN2 conformation already has a defect of +1 nt at this location compared to the canonical nucleosome: d_2_ = s_1.5_ - s_2.5_ + 1 nt. For each considered sequence, we compute the free energy landscape along the defect coordinate d_2_ to evaluate how the defect cost is affected by the placement of poly(dA:dT) tracts. We define the +1 nt defect free energy cost from the probabilities of observing a defect coordinate with values higher or lower than +0.5 nt: ΔF_d2_ = –log P(d_2_ > 0.5)+log P(d_2_ < 0.5).

To explore quantitatively how poly(dA:dT) tracts affect the energy cost of defects at SHL±2, we made Gaussian fits to the change in +1 nt defect free energy relative to the original 601 sequence as a function of the poly(dA:dT) tract location k relative to the dyad: ΔF_d+/−2_(k) = A exp (-(k-μ)^2^/2σ^2^), with Gaussian height A, mean μ, and standard deviation σ. One Gaussian fit was made for each considered poly(dA:dT) tract length (from 4 bp to 14 bp).

#### Kinetic model methods

ATP-driven sliding of nucleosomes by Chd1 were modeled using a master equation:


\begin{equation*}\frac{{{\mathrm{d}}{{\mathrm{P}}}_{\mathrm{i}}}}{{{\mathrm{dt}}}} = - \mathop \sum \limits_{{\mathrm{j}} \ne {\mathrm{i}}} {{\mathrm{k}}}_{{\mathrm{ji}}}{{\mathrm{P}}}_{\mathrm{i}} + \mathop \sum \limits_{{\mathrm{j}} \ne {\mathrm{i}}} {{\mathrm{k}}}_{{\mathrm{ij}}}{{\mathrm{P}}}_{\mathrm{j}} = \mathop \sum \limits_{\mathrm{j}} {{\mathrm{k}}}_{{\mathrm{ij}}}{{\mathrm{P}}}_{\mathrm{j}}\end{equation*}

where k_ij_ represents the rate of sliding from position j to i, and by construction ${{\mathrm{k}}}_{{\mathrm{ii}}} = - \mathop \sum \limits_{{\mathrm{j}} \ne {\mathrm{i}}} {{\mathrm{k}}}_{{\mathrm{ji}}}$. All DNA shifts were assumed to be 1 bp steps, so that k_ij_ = 0 for |i-j| > 1. The initial optimal 601 position is considered i = 0, with i allowed to be any integer between –N/2 and +N/2, where N + 1 = 225 (the length of DNA, though the precise length does not impact our results). The rate constants k_ij_ may be estimated by taking into account both the natural free energy landscape of the 601 sequence, and the activity of Chd1 at either SHL-2 or SHL+2.

The rotational preference of A/T steps on the 601 sequence causes the positioning free energy profile to adopt a sinusoidal shape with an amplitude of ∼10 k_B_T (27,33), which explains the observation that the probability distribution of nucleosome positions upon both spontaneous and active sliding display sharp peaks separated by multiples of ∼10 bp relative to the initial optimal position at ${\mathrm{i}} = 0$. Based on this, the 601 free energy landscape is assumed to have a sinusoidal shape,${\mathrm{F}}( {\mathrm{i}} ) = - \frac{{\mathrm{A}}}{2}\cos \frac{{2{\mathrm{\pi }}( {{\mathrm{i}} - {{\mathrm{i}}}_0} )}}{{\mathrm{\Delta }}}$, with amplitude ${\mathrm{A}} = 10$ k_B_T (27,33), periodicity ${\mathrm{\Delta}}$ = 10.5 bp (=147/14), and optimal location ${{\mathrm{i}}}_0 = 0$. The Chd1 remodeler can bind two symmetric locations on the nucleosome, either SHL-2 or SHL+2, and from there use energy from ATP hydrolysis to push the nucleosomal DNA toward the dyad, so that the nucleosome position ${\mathrm{i}}$ moves in the negative or positive direction, respectively. The overall rate of sliding takes into account the underlying 601 free energy profile, F(i), the energy provided by ATP hydrolysis, ${\mathrm{\Delta }}{{\mathrm{F}}}_{{\mathrm{ATP}}} = 20$ k_B_T (34), and the free energy cost of forming the +1 nt defects at SHL+/-2, ${\mathrm{\Delta }}{{\mathrm{F}}}_{{\mathrm{d}} + / - 2}\sim 0 - 2$ k_B_T, measured relative to that of the 601 sequence based on the MD simulations.

To fully define our model, we need to estimate the rates of sliding between each neighboring nucleosome positions i and i + 1 when the remodeler is bound at either SHL-2 or SHL+2. ATP-driven remodeling induces the forward sliding of nucleosomal DNA from the remodeler binding location toward the dyad: from i to i + 1 when binding at SHL+2, with rate


\begin{eqnarray*} && {\mathrm{k}}\left( {{\mathrm{i}} + 1{\mathrm{|i}},{\mathrm{shl}}+2} \right) = {\mathrm{D}}\exp \left( { - \frac{{{\mathrm{\Delta }}{{\mathrm{F}}}_{{\mathrm{d}} + 2}\left( {\mathrm{i}} \right)}}{{{{\mathrm{k}}}_{\mathrm{B}}{\mathrm{T}}}}} \right)\nonumber\\ && \quad \exp \left( { - \frac{{{\mathrm{F}}\left( {{\mathrm{i}} + 1} \right) - {\mathrm{F}}\left( {\mathrm{i}} \right)}}{{2{{\mathrm{k}}}_{\mathrm{B}}{\mathrm{T}}}}} \right)\exp \left( { - \frac{{{\mathrm{\Delta }}{{\mathrm{F}}}_{{\mathrm{ATP}}}}}{{{{\mathrm{k}}}_{\mathrm{B}}{\mathrm{T}}}}} \right)\end{eqnarray*}

and from i + 1 to i when binding at SHL-2, with rate


\begin{eqnarray*} && {\mathrm{k}}\left( {{\mathrm{i|i}} + 1,{\mathrm{shl}}-2} \right) = {\mathrm{D}}\exp \left( { - \frac{{{\mathrm{\Delta }}{{\mathrm{F}}}_{{\mathrm{d}} - 2}\left( {{\mathrm{i}} + 1} \right)}}{{{{\mathrm{k}}}_{\mathrm{B}}{\mathrm{T}}}}} \right)\nonumber\\ && \quad \exp \left( { - \frac{{{\mathrm{F}}\left( {\mathrm{i}} \right) - {\mathrm{F}}\left( {{\mathrm{i}} + 1} \right)}}{{2{{\mathrm{k}}}_{\mathrm{B}}{\mathrm{T}}}}} \right)\exp \left( { - \frac{{{\mathrm{\Delta }}{{\mathrm{F}}}_{{\mathrm{ATP}}}}}{{{{\mathrm{k}}}_{\mathrm{B}}{\mathrm{T}}}}} \right)\end{eqnarray*}

whereas spontaneous backward sliding away from the dyad will move the nucleosome from i + 1 to i when the remodeler binds at SHL+2 with rate


\begin{eqnarray*} && {\mathrm{k}}\left( {{\mathrm{i|i}} + 1,{\mathrm{shl}} + 2} \right) = {\mathrm{D}}\exp \left( { - \frac{{{\mathrm{\Delta }}{{\mathrm{F}}}_{{\mathrm{d}} + 2}\left( {\mathrm{i}} \right)}}{{{{\mathrm{k}}}_{\mathrm{B}}{\mathrm{T}}}}} \right)\nonumber\\ && \exp \left( { - \frac{{{\mathrm{F}}\left( {\mathrm{i}} \right) - {\mathrm{F}}\left( {{\mathrm{i}} + 1} \right)}}{{2{{\mathrm{k}}}_{\mathrm{B}}{\mathrm{T}}}}} \right)\end{eqnarray*}

and from i to i + 1 when the remodeler binds at SHL-2 with rate


\begin{eqnarray*} && {\mathrm{k}}\left( {{\mathrm{i}} + 1{\mathrm{|i}},{\mathrm{shl}} - 2} \right) = {\mathrm{D}}\exp \left( { - \frac{{{\mathrm{\Delta }}{{\mathrm{F}}}_{{\mathrm{d}} - 2}\left( {{\mathrm{i}} + 1} \right)}}{{{{\mathrm{k}}}_{\mathrm{B}}{\mathrm{T}}}}} \right)\nonumber\\ && \quad \exp \left( { - \frac{{{\mathrm{F}}\left( {{\mathrm{i}} + 1} \right) - {\mathrm{F}}\left( {\mathrm{i}} \right)}}{{2{{\mathrm{k}}}_{\mathrm{B}}{\mathrm{T}}}}} \right)\end{eqnarray*}

where D is an overall rate constant with units of inverse time.

To derive these expressions, we considered that in the absence of ATP, the kinetics should satisfy the detailed balance condition, k(i + 1|i)P_eq,B_(i) = k(i|i + 1)P_eq,B_(i + 1), with the steady state probabilities following the Boltzmann distribution P_eq,B_(i) ∼ exp(–F(i)/k_B_T). ATP hydrolysis effectively shifts the free energy difference upon successful sliding by an amount equal to ΔF_ATP_. Since sliding by chromatin remodelers requires the generation of intermediate +1 nt defects at SHL+/-2, a barrier height ΔF_d+/–2_ to reach such states suppresses sliding by an Arrhenius-like term exp(–ΔF_d+/−2_/k_B_T), but it does not change the free energy of the initial and final states. Assuming that nucleosomes are always bound by Chd1 at SHL-2 or SHL+2 with equal probability, and that Chd1 jumps from one side to the other relatively fast, the total rates of sliding from i to i + 1 and back are:


\begin{eqnarray*} && {\mathrm{k}}\left( {{\mathrm{i}} + 1{\mathrm{|i}}} \right) = {\mathrm{k}}\left( {{\mathrm{i}} + 1{\mathrm{|i}},{\mathrm{shl}} - 2} \right) + {\mathrm{k}}\left( {{\mathrm{i}} + 1{\mathrm{|i}},{\mathrm{shl}} + 2} \right)\nonumber\\ && \quad \approx {\mathrm{k}}\left( {{\mathrm{i}} + 1{\mathrm{|i}},{\mathrm{shl}} + 2} \right)\end{eqnarray*}

\begin{eqnarray*} && {\mathrm{k}}\left( {{\mathrm{i|i}} + 1} \right) = {\mathrm{k}}\left( {{\mathrm{i|i}} + 1,{\mathrm{shl}} - 2} \right) + {\mathrm{k}}\left( {{\mathrm{i|i}} + 1,{\mathrm{shl}} + 2} \right)\nonumber\\ && \quad \approx {\mathrm{k}}\left( {{\mathrm{i|i}} + 1,{\mathrm{shl}} - 2} \right)\end{eqnarray*}

where the approximation results from the fact that the ATP hydrolysis free energy dominates compared to all the other terms, ΔF_ATP_ = 20 k_B_T >> F(i + 1) –F(i) ∼ ΔF_d+/−2_ ∼ k_B_T, making Chd1-induced sliding from its binding site toward the dyad dominate compared to backsliding. The steady state probabilities satisfy:


\begin{eqnarray*} && {{\mathrm{p}}}_{{\mathrm{eq}}}\left( {{\mathrm{i}} + 1} \right)/{{\mathrm{p}}}_{{\mathrm{eq}}}\left( {\mathrm{i}} \right) = {\mathrm{k}}\left( {{\mathrm{i}} + 1{\mathrm{|i}}} \right)/{\mathrm{k}}\left( {{\mathrm{i|i}} + 1} \right)\nonumber\\ && \quad = \exp \left( { - \frac{{{\mathrm{\Delta }}{{\mathrm{F}}}_{{\mathrm{d}} + 2}\left( {\mathrm{i}} \right) - {\mathrm{\Delta }}{{\mathrm{F}}}_{{\mathrm{d}} - 2}\left( {{\mathrm{i}} + 1} \right)}}{{{{\mathrm{k}}}_{\mathrm{B}}{\mathrm{T}}}}} \right)\nonumber\\ && \qquad \exp \left( { - \frac{{{\mathrm{F}}\left( {{\mathrm{i}} + 1} \right) - {\mathrm{F}}\left( {\mathrm{i}} \right)}}{{{{\mathrm{k}}}_{\mathrm{B}}{\mathrm{T}}}}} \right)\end{eqnarray*}

From which the effective free energy of the system can be defined as ${{\mathrm{F}}}_{{\mathrm{eff}}}( {\mathrm{i}} ) = - {{\mathrm{k}}}_{{\mathrm{B}}}{\mathrm{T}}\log {{\mathrm{p}}}_{{\mathrm{eq}}}( {\mathrm{i}} )$, giving:


\begin{eqnarray*} && {{\mathrm{F}}}_{{\mathrm{eff}}}\left( {{\mathrm{i}} + 1} \right){\mathrm{\ }} - {{\mathrm{F}}}_{{\mathrm{eff}}}\left( {\mathrm{i}} \right){\mathrm{\ }} = {\mathrm{\Delta }}{{\mathrm{F}}}_{{\mathrm{d}} + 2}\left( {\mathrm{i}} \right){\mathrm{\ }}\nonumber\\ && \quad - {\mathrm{\ \Delta }}{{\mathrm{F}}}_{{\mathrm{d}} - 2}\left( {{\mathrm{i}} + 1} \right) + {\mathrm{F}}\left( {{\mathrm{i}} + 1} \right){\mathrm{\ }} - {\mathrm{F}}\left( {\mathrm{i}} \right)\end{eqnarray*}

The final effective nucleosome free energy is given by the original underlying 601 free energy plus a term that takes into account the activation barrier due to the formation of +1 nt defects at the Chd1 binding site: for example, when defects at SHL-2 cost a lot of energy, sliding in the forward direction for that side is inhibited, corresponding to an increase in free energy to shift DNA from position i to i –1. Conveniently, the final effective free energy depends only on terms that can be estimated from the past literature and from the MD simulations presented here.

The estimated cost of a +1 nt defect in the context of a poly(dA:dT) tract was given by ΔF_d-2_(i|x) = ΔF_d-2_ (k = x-i|MD), where ΔF_d-2_(i|x) is the position-dependent defect energy cost at SHL2 given a poly(dA:dT) tract initially at x relative to the dyad, and ΔF_d-2_(k = x-i|MD) is the energy cost measured from MD simulations a function of the tract location k = x-i relative to the dyad. Poly(dA:dT) tracts increased the free energy cost of defects given by a Gaussian function with a height of 2 k_B_T centered at k = –21 bp and with a standard deviation σ = 0.22L + 1.1 bp, according to a linear fit to our MD simulation results.

To quantify the effect of poly(dA:dT) tracts on remodeling, two effective energy barriers as a function of poly(dA:dT) tract location x and length L were defined as ${\mathrm{\Delta }}{{\mathrm{F}}}_{10} = {{\mathrm{F}}}_{{\mathrm{eff}}}( { - 10{\mathrm{bp}}} ) - {{\mathrm{F}}}_{{\mathrm{eff}}}( {0{\mathrm{bp}}} ) = - \log {{\mathrm{p}}}_{{\mathrm{eq}}}( { - 10{\mathrm{bp}}} ) + \log {{\mathrm{p}}}_{{\mathrm{eq}}}( {0{\mathrm{bp}}} )$ (the difference in the effective free energies at –10 bp and 0 bp, where ΔF_10_ represents the overall negative remodeling barrier introduced by the poly(dA:dT) tract); and ${\mathrm{\Delta }}{\mathrm{{F}}}_1 = {{\mathrm{F}}}_{{\mathrm{eff}}}( { - 1{\mathrm{bp}}} ) - {{\mathrm{F}}}_{{\mathrm{eff}}}( {0{\mathrm{bp}}} )$ (the initial remodeling barrier, which considered only the initial cost of defects due to the poly(dA:dT) tract). Note that although the effective free energy of the system is determined by the steady state probabilities of nucleosome positions, the free energy barriers are helpful to quantify how difficult it is for nucleosomes to be repositioned, regardless of whether the steady state is actually reached.

## RESULTS

### Poly(dA:dT) tracts can influence local and global positioning of the Widom 601 sequence

We used next-generation sequencing (NGS) to determine nucleosome positions at high-resolution for a population of related sequences (Figure 1). To identify the location of the histone core on DNA, we adapted a site-specific DNA cleavage method described by Bartholomew and coworkers (23). In this method, DNA can be site-specifically cleaved on the nucleosome, 53 bp from the dyad, using a histone core containing H2B(S53C) labelled with the photo-cross-linker azido phenacylbromide (Figure 1B,C). After UV cross-linking and processing, each strand of the DNA duplex is cleaved only once on one side of the nucleosome. The sites of cleavage can be determined by NGS, which in turn reveals positioning of the histone core. Since the two H2B(S53C) sites are located 53 bp from the dyad, the locations of these two independent cross-linking sites can be used to determine the position of the nucleosome dyad for each unique sequence in the library (Figure 1D,E).

To determine the precise positions and lengths where poly(dA:dT) tracts affect activity of Chd1, we generated a library where every position of the canonical 145 bp 601 sequence (17) is the starting point for a tract of 3–15 bp. After amplifying the library using emulsion PCR (35), nucleosomes were assembled using the standard salt gradient dialysis method, then UV cross-linked and processed for sequencing, either before or after repositioning by yeast Chd1. We found that a number of poly(dA:dT) tracts strongly affected nucleosome positioning during salt gradient dialysis, prior to repositioning by Chd1. We first describe how the lengths and locations of poly(dA:dT) tracts altered the expected sharp dyad positioning of the canonical Widom sequence. We then discuss how the length and position of poly(dA:dT) tracts influenced nucleosome repositioning by Chd1.

**Figure 2.**
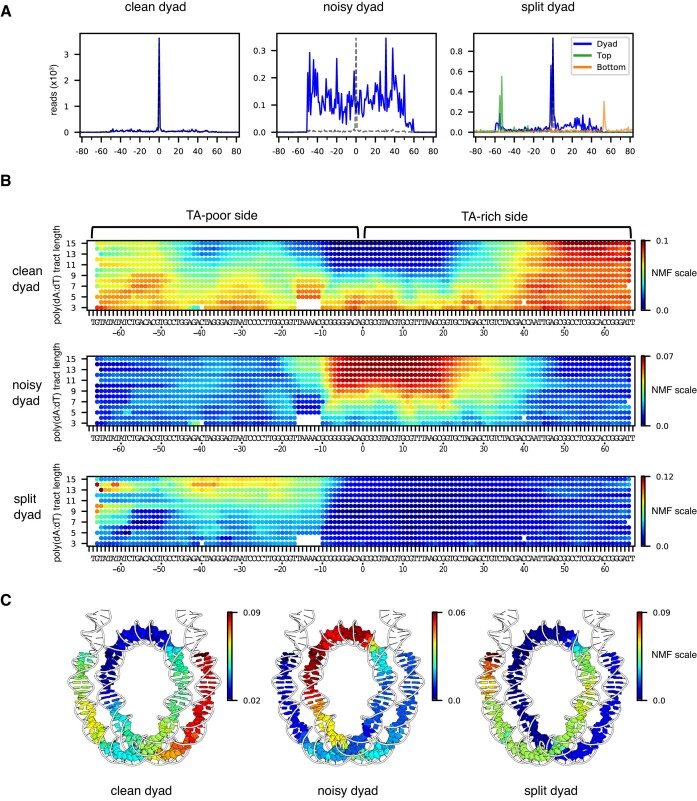
NMF analysis for nucleosome positioning on the 601 poly(dA:dT) library before sliding reveals the asymmetric nature of nucleosome stability within the Widom 601 library. (**A**) For nonnegative matrix factorization (NMF) analysis, all nucleosome positioning signals of the 601 poly(dA:dT) library were categorized based on three basis patterns: (1) a single dominant peak as expected for the 601 (clean dyad), (2) a noisy pattern (noisy dyad), and (3) doublet peaks for the top strand and calculated dyad (split dyad). Calculated dyad positions are shown in blue, and H2B(S53C) cleavage sites in orange and green. Y-axis values are relative frequencies. (**B**) A geographic heat map of NMF scores for each dyad pattern. Poly(dA:dT) tract lengths are indicated on the left. (**C**) NMF scores for nucleosomes containing 10 bp poly(dA:dT) tracts mapped onto the nucleosome structure (6WZ5).

After generating a library of nucleosomes containing poly(dA:dT) tracts, dyad positions were mapped and sorted based on poly(dA:dT) position and length (Supplementary Figures S1 and S2, Supplementary Table S2). Based on the similarity of nucleosome positions and perturbation patterns, the poly(dA:dT) library data was clustered into 6 groups (Supplementary Figure S3). To visualize where and how poly(dA:dT) tracts most strongly affected nucleosome positioning, we used a machine learning analysis method, called non-negative matrix factorization (NMF) (36) (Figure 2). With this method, the complex pattern of dyad positions for each sequence could be linearly decomposed into a few basis patterns with corresponding weights. Here, these weights are referred to as the NMF scores for each pattern. For a given length of poly(dA:dT) tract (3-mer, 4-mer, etc), all NMF scores were mapped onto the Widom 601 sequence according to tract location. This mapping construction produced a ‘geographic’ heat map indicating where poly(dA:dT) tracts of different lengths were most prevalent for each dyad pattern. We found three predominant dyad patterns, which we refer to as ‘clean’, ‘noisy’ and ‘split’ (Figure 2A). For each pattern, high NMF scores indicate the locations and lengths of poly(dA:dT) tracts that produced a similar distribution.

The clean dyad pattern resembles the canonical Widom 601, and therefore shows where poly(dA:dT) tracts did not disrupt the 601-directed positioning of the histone core. This pattern was enriched in the shorter tracts, and those located 40 bp or more away from the dyad on the TA-rich side (Figure 2B). To visualize these enrichment patterns, we mapped the NMF scores for the 10 bp poly(dA:dT) tracts on the nucleosome structure (Figure 2C). In contrast to the unique 601-directed dyad position seen for the clean dyad pattern, the noisy dyad pattern had a broad distribution (Figure 2A), which indicates that these poly(dA:dT) tracts interfered with positioning of the 601 sequence. The noisy pattern was enriched in the longer poly(dA:dT) tracts (>8 bp) and extended over a ∼40 bp segment that overlapped the canonical 601 dyad and was mostly on the TA-rich side of the 601 sequence (Figure 2B). Here, we refer to the TA-rich side of the 601 sequence with positive SHLs and sequence numbering (SHL+2, SHL+3, etc), and the TA-poor side of the 601 with negative SHLs and sequence numbering (SHL-2, SHL-3, etc). The TA steps have been shown to be important sequence elements that allow nucleosomal DNA to wrap more favorably (37), and therefore the elimination of these sequence elements by overlapping poly(dA:dT) tracts may explain disruptions of the canonical 601 dyad position. However, the noisy dyad pattern also had strong NMF scores with some shorter, specifically placed poly(dA:dT). In particular, poly(dA:dT) tracts as short as 6 bp were enriched at SHL+1 and SHL+2, between positions [8:13] and [19:24] (Supplementary Table S2). Interestingly, several of these sites correspond with where the minor groove of DNA would be outward-facing and therefore widest on canonical 601 nucleosomes, suggesting that these poly(dA:dT) tracts may interfere with positioning due to their intrinsically narrow minor groove width (38).

The split dyad pattern arose from a 2 nt shift in H2B cross-link in only the top strand, which is on the TA-poor side of the 601 sequence (Figure 2A). This pattern is reminiscent of a previously observed shift of entry-side DNA upon binding of Chd1 to the TA-poor side of Widom 601 nucleosomes (39). As shown by cryo-EM work, entry-side DNA is shifted toward the dyad upon binding of remodeler ATPases to SHL2 in a nucleotide-free or ADP-bound state (7,40,41). Here, a similar shift appears to be stimulated with poly(dA:dT) sequences located on the TA-poor side of the Widom 601 sequence (Figure 2B,C). This pattern suggests that poly(dA:dT) tracts on the TA-poor side do not affect the global positioning of the nucleosome, but instead cause a local shift of entry/exit DNA around the cross-linking site, without the need for binding of a remodeler ATPase at SHL-2.

### Poly(dA:dT) tracts that affect nucleosome positioning are sometimes outside of the Chd1 binding site

**Figure 3.**
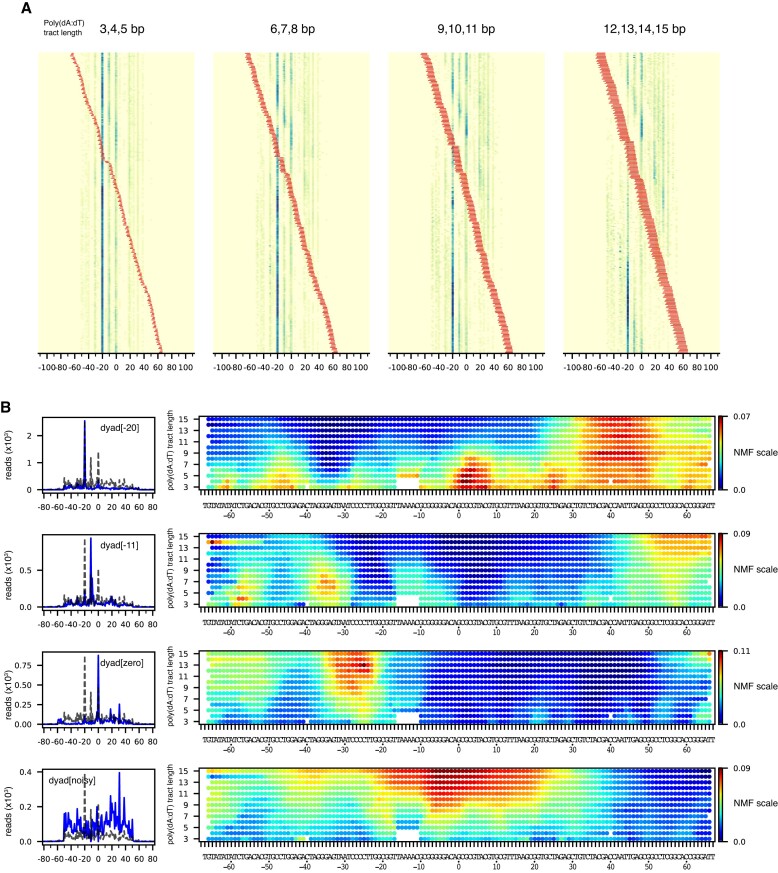
Poly(dA:dT) tracts alter the distribution of nucleosomes after sliding by Chd1. (**A**) Heatmaps for estimated nucleosome dyads signals after sliding by Chd1. The sequences are grouped according to the poly(dA:dT) tract lengths, 3–5, 6–8, 9–11 and 12–15 bp, and sorted by tract locations (red). (**B**) Dyad patterns (left) and NMF heatmaps (right) for the four dominant classes of calculated dyads. In the dyad patterns, the dotted lines indicate the dyad distribution for Chd1 sliding of the canonical Widom 601 nucleosome.

The poly(dA:dT) tract library also showed where different lengths and positions of tracts affected nucleosome positioning by the Chd1 remodeler (Figure 3A, Supplementary Figure S4). Previously, Chd1 was shown to preferentially shift Widom 601 nucleosomes flanked by 40 bp DNA on either side (40N40) toward the TA-poor side (18). After nucleosome sliding by Chd1, we obtained a similar distribution for many nucleosomes in the poly(dA:dT) library, with the strongest peak 20 bp away from the starting position. However, many lengths and positions of poly(dA:dT) tracts influenced nucleosome positioning by Chd1.

We generated NMF plots based on four common nucleosome repositioning patterns: (i) a 20 bp shift toward the TA-poor side (dyad[-20]), similar to the canonical Widom 601; (ii) a predominant 11 bp shift toward the TA-poor side (dyad[-11]); (iii) no shift (dyad[zero]) and (iv) a noisy dyad pattern (Figure 3B). The noisy dyad pattern correlated with the positions and lengths of polyA(dA:dT) tracts that disrupted the canonical 601 positioning prior to remodeling by Chd1 (noisy dyad, Figure 2).

**Figure 4.**
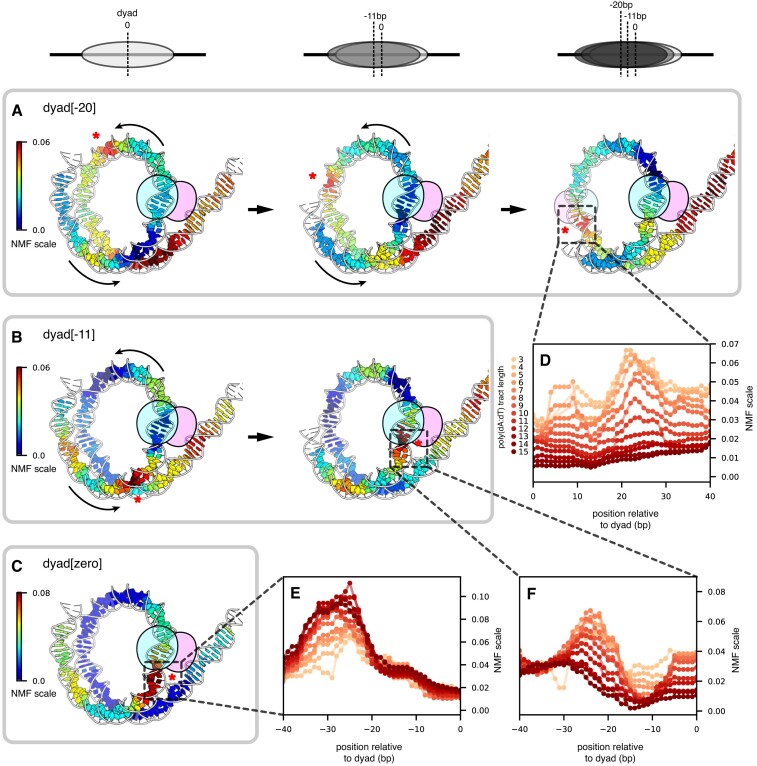
Poly(dA:dT) tracts on the downstream side of Chd1 binding sites bias nucleosome repositioning. (**A**) NMF scores for the 8 bp poly(dA:dT) tracts with the dyad[-20] pattern, mapped onto a nucleosome-Chd1 complex solved by cryo-EM (7TN2). Shown are NMF scores at three translational positions of the 601 sequence: at the starting position for 601 (left), after an 11 bp shift (middle), and after a 20 bp shift (right). (**B**) NMF scores for the 8 bp poly(dA:dT) tracts with the dyad[-11] pattern, mapped onto a nucleosome-Chd1 complex (7TN2). Shown are NMF scores at two translational positions of the 601 sequence: at the starting position for 601 (left), and after an 11 bp shift (right). (**C**) NMF scores for the 8 bp poly(dA:dT) tracts with the dyad[zero] pattern, mapped onto a nucleosome-Chd1 complex (7TN2). Shown are NMF scores at the starting position for 601. (**D**) NMF scores for all tract lengths with the dyad[-20] pattern, corresponding to the region around the opposing SHL+2 after a 20 bp shift. (**E**) NMF scores for all tract lengths with the dyad[zero] pattern, corresponding to the region around SHL-2. (**F**) NMF scores for all tract lengths with the dyad[-11] pattern, corresponding to the region around SHL-2 after an 11 bp shift. Asterisks highlight prevalent sites (high NMF scores), and where these sites would be repositioned after nucleosome sliding.

Since Chd1 is known to bind and translocate DNA when bound to SHL2, we can interpret the influence of the poly(dA:dT) tracts by their position relative to the remodeler binding site. To visualize how these tracts may affect the remodeler at SHL-2, we mapped the NMF heatmaps onto the nucleosome before and after repositioning (Figure 4). Poly(dA:dT) tracts that favored the dyad[-20] pattern, and therefore had little effect on Chd1 activity, were primarily located on the opposite side of the nucleosome, the TA-rich side (Figure 4A). Notably, one cluster was enriched between SHL0 and SHL+1, which places these tracts between SHL+2 and SHL+3 after a 20 bp shift, a location that would likely diminish Chd1 action on the TA-rich side. In contrast, the sequences that interfered with the expected Chd1 sliding pattern were closer to the SHL-2 site on the TA-poor side (Figure 4B, C).

Poly(dA:dT) sequences that appeared to block sliding by Chd1 (dyad[zero]) were distributed asymmetrically with respect to the ATPase motor binding site (Figure 4C). Like other remodelers, the Chd1 ATPase motor directly contacts both strands of DNA at SHL+/-2, 16–24 bp from the dyad (7–9). Although many poly(dA:dT) tracts that increased the dyad[zero] population overlapped with the SHL-2 binding site, several were notable for being completely outside the binding site, and in particular around SHL-3 (e.g. A_8_[-36:-29], A_8_[-35:-28], and A_8_[-34:-27]; Supplementary Table S3).

The poly(dA:dT) tracts that favored the dyad[-11] pattern were predominantly located between SHL-3 and SHL-4 (Figure 3B). These poly(dA:dT) tracts allowed for an initial 11 bp shift of the histone core, but then reduced or blocked further movement. After an 11 bp shift, most tracts enriched in the dyad[-11] pattern would be located between SHL-2 and SHL-3 (Figure 4B). These experiments therefore indicate that poly(dA:dT) strongly affected nucleosome positioning when they overlapped with the ATPase binding site and were within one helical turn of the binding site on the downstream (SHL-3) side.

### Simulations predict that the costs of DNA distortions at SHL-2 are affected by poly(dA:dT) tracts at SHL-2 but not SHL-3

One perplexing finding from these experiments was the accumulation of poly(dA:dT) tracts outside the Chd1 binding site. Are these tracts expected to directly interfere with Chd1 activity? Nucleosome sliding by chromatin remodelers has been hypothesized to proceed through changes in DNA twist (42,43). As seen in a recent high-resolution structure of Chd1 in the nucleotide-free state, DNA at SHL2 was under-twisted by the ATPase motor into an A-form-like structure (7). Importantly, this A-form-like DNA structure allows one strand, called the tracking strand, to accommodate an additional nucleotide. The A-form-like structure is believed to represent a key intermediate in creation of a twist defect, an under-twisted structure of DNA that absorbs a full bp.

**Figure 5.**
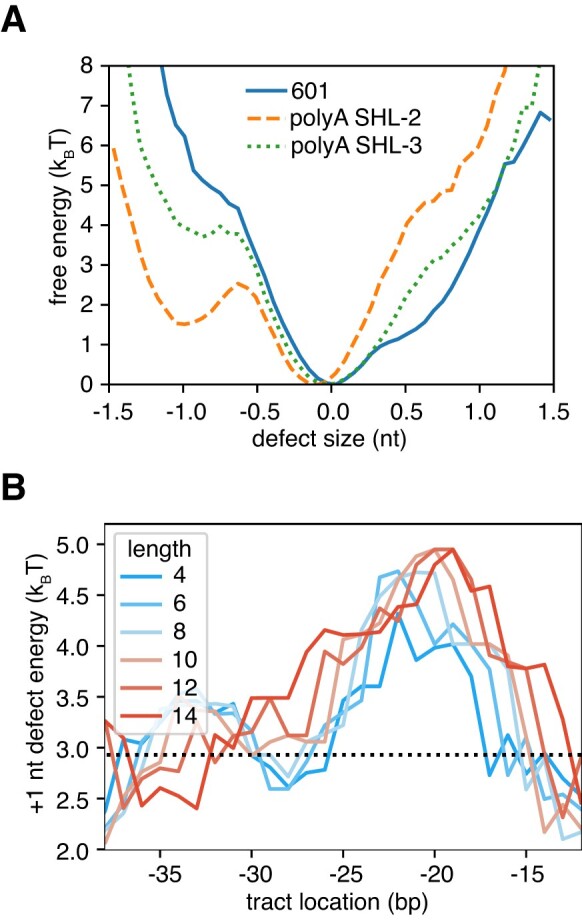
Free energy landscape of a DNA distortion at SHL-2, and its modulation by poly(dA:dT) tracts. (**A**) The free energy (negative logarithm of the probability distribution) for a tracking strand twist defect at SHL-2 for three nucleosomal DNA sequences: 601 (solid, blue line), 601 with a 10 bp poly(dA:dT) tract inserted at SHL-2 (dashed, orange), and 601 with a 10 bp poly(dA:dT) tract inserted at SHL-3 (dotted, green). (**B**) The free energy cost of +1 nt defects, ΔF_d-2_= –log P(d_-2_> 0.5)+log P(d_-2_< 0.5), for 601 nucleosomes with varying poly(dA:dT) tract location and length (from 4 bp to 14 bp). The defect cost for the original 601 sequence is shown as a black line. Errors on the energy estimates, which are not shown on the plot, are on the order of 0.2 k_B_T.

One way that DNA sequence could affect nucleosome sliding is by altering the intrinsic stability of structural intermediates (26,27). To estimate the cost of poly(dA:dT) tracts for a twist defect intermediate, we performed coarse-grained MD simulations of nucleosomes to calculate the probability (and thus the free energy) of shifting the tracking strand at SHL-2. Performed in the absence of the ATPase motor, these simulations varied in the locations and lengths of poly(dA:dT) tracts around SHL-2. In comparing a 10 bp poly(dA:dT) tract centered on SHL-2 or SHL-3, the simulations showed that the tracking strand was much less likely to occupy a +1 nt shifted position when a 10 bp poly(dA:dT) sequence was located at SHL-2, yet the shift (at SHL-2) was unaffected by a poly(dA:dT) sequence at SHL-3 (Figure 5A). With a range of locations and sizes of poly(dA:dT) tracts, a +1 nt shift of the tracking strand at SHL-2 was found to be most expensive when the tracts were centered at SHL-2 (Figure 5B). To quantitatively analyze the penalty of poly(dA:dT) tracts, the differences in free energy of a +1 nt shift (between canonical 601 and poly(dA:dT) tracts) were fitted with a Gaussian function as a function of the poly(dA:dT) tract location. These Gaussian fits revealed a common peak around 21 bp from the dyad (that is, centered at SHL-2), with an amplitude corresponding to ∼2 k_B_T for poly(dA:dT) tracts longer than 4 bp (Supplementary Figure S5). These results indicated that poly(dA:dT) tracts at SHL-3 did not affect the intrinsic cost of a +1 nt shift of the tracking strand at SHL-2.

### DNA perturbations at SHL-2 interfere with Chd1 binding and nucleosome sliding activity

Since coarse-grained simulations suggest that poly(dA:dT) tracts would not have a direct impact on an initial formation of twist defects when located at SHL-3, what might be the source of poly(dA:dT) enrichment around SHL-3 in the nucleosome sliding experiments? For the sliding experiments, nucleosomes start off in a central location, and the final distribution results from remodeler action at the two SHL2 sites. Although nucleosome sliding may be perturbed when a particular sequence reaches SHL-2, that sequence can be shifted back toward SHL-3 by action of a remodeler on SHL+2, on the opposite side of the nucleosome. Therefore, the most interfering sequences do not settle at SHL-2 during back-and-forth sliding experiments. Instead, these sequences may be ‘pushed back’ from SHL-2 to SHL-3 due to remodeler action on SHL+2.

To test where Chd1 was directly affected by DNA sequence, we investigated the impacts of localized DNA perturbations on a nucleosome substrate that should slide only in one direction. As shown previously, Chd1 requires entry side DNA for shifting nucleosomes, and therefore asymmetric nucleosomes such as 80N0 are initially only shifted toward one side (16). To locally disrupt DNA, and potentially Chd1-DNA interactions, we placed pairs of abasic sites (1AP) or 2 bp DNA mismatches (2bp mm) on the nucleosome, either at both SHL2 sites (SHL+/-2, 20 bp from the dyad, overlapping with the Chd1 binding sites), or both SHL2.7 sites (SHL+/-2.7, 27 bp from the dyad, just outside the Chd1 binding sites). Since 80N0 nucleosomes are initially only shifted toward the 80 bp linker, these experiments were designed to reveal how much perturbations at the SHL-2 and SHL-2.7 sites affect Chd1 activity, without bias from normal activity or binding at the SHL+2 or SHL+2.7 sites.

**Figure 6.**
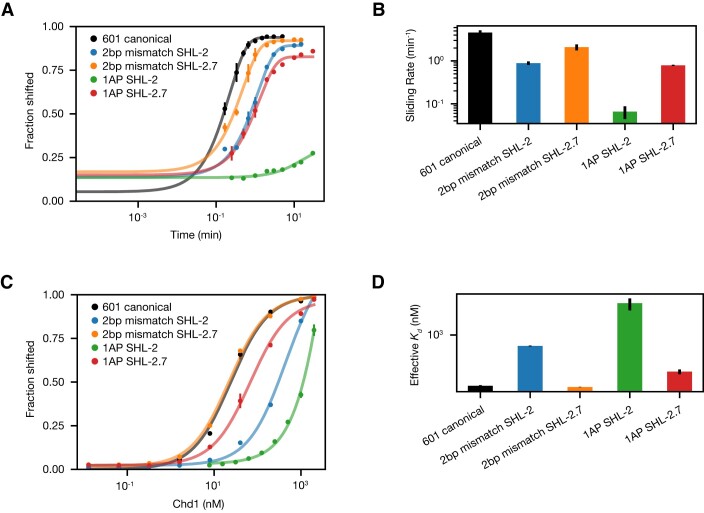
Chd1 sliding activity and binding are most strongly affected by DNA perturbations at SHL-2. (**A**) Quantification of nucleosome sliding reactions, using 80N0 nucleosomes with the indicated defect at either SHL-2 or SHL-2.7. Shown are the means plus standard deviations from three replicates. Lines show the best single exponential fits. (**B**) Nucleosome sliding rates, based on fits shown in (A). Rates (min^−1^) were calculated to be 4.6 ± 0.6 (601 canonical); 0.89 ± 0.09 (2 bp mismatch, SHL-2); 2.1 ± 0.3 (2 bp mismatch, SHL-2.7); 0.07 ± 0.02 (1AP SHL-2); and 0.79 ± 0.02 (1AP SHL-2.7). For each construct, the average value was determined from three or more reactions, with error bars indicating standard deviations. (**C**) Binding titrations for nucleosomes shown in (A), carried out in the presence of AMP-PNP and salmon sperm DNA. (**D**) Observed binding affinities as shown in (C). Observed *K*_d_ values (nM) were calculated to be 24 ± 1 (601 canonical); 450 ± 15 (2 bp mismatch, SHL-2); 22.2 ± 0.3 (2 bp mismatch, SHL-2.7); 10 000 ± 4000 (1AP SHL-2); and 68 ± 12 (1AP SHL-2.7). Binding reactions were performed three or more times, with error bars indicating the standard deviations of fit values. Representative gels for sliding and binding reactions are shown in Supplementary Figure S6.

As monitored by shifts on native acrylamide gels, nucleosomes with defects at SHL+/-2 were affected more strongly than those at SHL+/-2.7, and pairs of opposing abasic sites were much more deleterious than double mismatches (Figure 6A,B, Supplementary Figure S6). With abasic sites at SHL+/-2, virtually no nucleosome repositioning was observed. In contrast, nucleosomes were robustly shifted ∼10 bp (∼5 times slower than canonical 601 nucleosomes) with the abasic pair at SHL+/-2.7. The SHL+/-2 site also showed slower repositioning (∼5-fold) than SHL+/-2.7 (∼2-fold) with two consecutive mismatches. To determine if these differences in rates were correlated with poorer binding, these different nucleosome substrates were bound to Chd1 in the presence of salmon sperm competitor DNA. The binding experiments suggest that these DNA perturbations at SHL+/-2 interfered with Chd1 binding, as the apparent affinities decreased by 50- to over 500-fold (Figure 6C, D).

These experiments show that DNA perturbations centered on the Chd1 binding site (SHL+/-2) have a much greater impact on nucleosome sliding and binding than those just outside (SHL+/-2.7). Given the detectable effects at SHL+/-2.7, Chd1 binding or activity may be altered by perturbations outside its binding site. However, given the relatively mild impact on sliding rate, we expect that, in the context of a centered nucleosome, the major effects on Chd1 activity likely arise from perturbations overlapping the Chd1 binding site.

### With back-and-forth sliding by Chd1, nucleosomes show accumulation of DNA perturbations at SHL-3

To see how site-specific DNA perturbations influence nucleosome positioning by Chd1 in the context of back-and-forth sliding, we performed Slide-seq experiments on 40N40 nucleosomes based on two 601-based libraries. In one library, one to five consecutive mismatches were introduced, where non-A•T bp were replaced with an A on the top strand or T on the bottom strand. In the second library, the canonical 601 sequence was interrupted by single-nucleotide insertions, where an additional A nucleotide was introduced on either the top or the bottom strand along the Widom 601 sequence. Both of these perturbations relied upon having one strand maintain the canonical 601 sequence, and the other strand possess a discrete difference. To achieve this, the canonical and altered 601 sequences were separately amplified with PCR, and then one of the two strands selectively destroyed with lambda exonuclease, which targets 5′ phosphorylated substrates (44). By annealing the remaining strands together (e.g. the top strand from amplification of the canonical 601 sequence and bottom strand of amplification of a variant 601 sequence), the desired DNA template—properly base-paired except at the site of the sequence difference—could be produced (Supplementary Figure S7). With these modified duplexes, nucleosomes were generated through salt gradient dialysis and then cross-linked and sequenced before and after sliding by Chd1.

Before sliding by Chd1, the mismatch library of 601 nucleosomes showed similar trends as those of the poly(dA:dT) library. Whereas a majority of mismatch positions yielded a sharp dyad signal like the canonical 601 (‘clean dyad’), sites on and around the dyad gave a broader distribution of dyad positions (‘noisy dyad’, Supplementary Figures S8-S10, Supplementary Table S4). Unlike the poly(dA:dT) library, though, the most disruptive sites were on the TA-poor side of the dyad. Interestingly, the mismatches also produced a ‘split dyad’ signal, which, like the poly(dA:dT) library, was due to a cross-linking doublet on the TA-poor side of the 601 sequence. For the poly(dA:dT) tracts, the cross-linking doublet was most prominent with long tracts spanning SHL-2 to SHL-4. For the mismatches, however, the cross-linking doublet was limited to those at SHL-2, located 16–25 bp from the dyad (Supplementary Figure S10). Interestingly, this is precisely where chromatin remodelers distort nucleosomal DNA to allow absorption of an extra nucleotide, which stimulates movement of entry DNA toward the remodeler (7,39,41).

**Figure 7.**
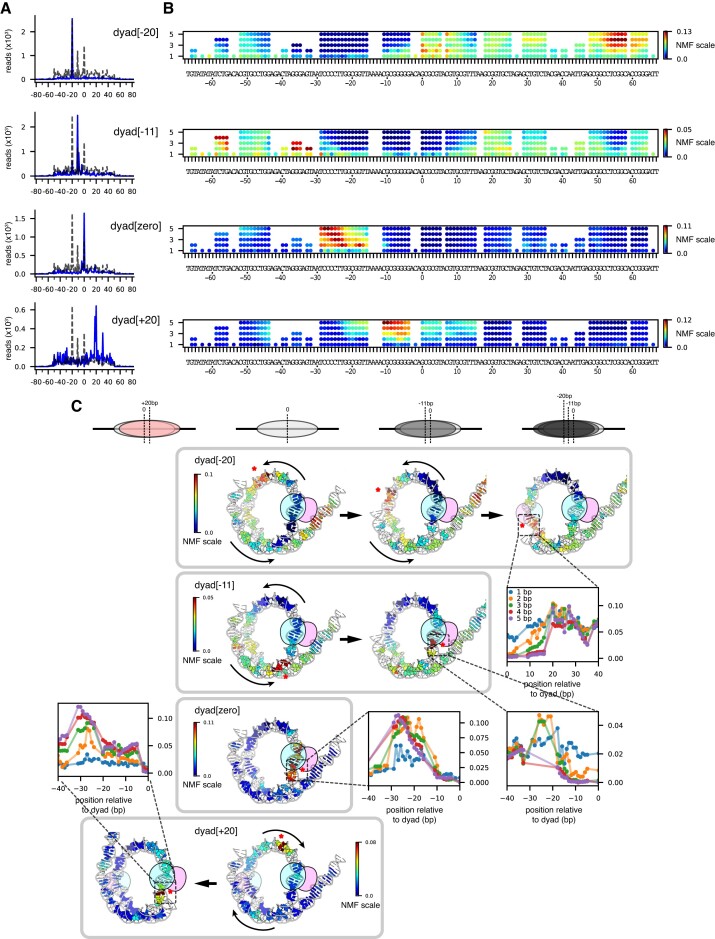
Mismatches bias the distribution of nucleosome positions after sliding by Chd1. (**A**) Four basis dyad patterns observed after sliding by Chd1, which were used for NMF scoring (blue). The distribution of the canonical 601 is shown with dotted lines. (**B**) NMF scores mapped onto the Widom 601 DNA sequence based on the dyad[-20], dyad[-11], dyad[zero], and dyad[+20] patterns. (**C**) NMF scores for 3 bp mismatches mapped onto a Chd1-nucleosome complex (7TN2) at different translational positions, as shown in Figure 4. Asterisks highlight prevalent sites (high NMF scores), and where these sites would be repositioned after nucleosome sliding.

After remodeling with Chd1, both the mismatch library and the single-nt insertion library showed position-dependent effects on nucleosome positioning. Both libraries gave trends similar to the poly(dA:dT) library: some nucleosomes were shifted 20 bp toward the TA-poor side (dyad[-20]), some were predominantly shifted only 11 bp (dyad[-11]), and others remained at the starting position (dyad[zero]) (Figure 7, Supplementary Figures S11-S14). As with poly(dA:dT) tracts, DNA perturbations (≥2 bp mismatches and single-nt insertions) between SHL-2 and SHL-3 were enriched in the dyad[zero] population, including perturbations that were completely outside the Chd1 binding site (e.g. M_2_[-29:-28] and I_1_[-29^-28], Supplementary Tables S4-S7). The preferred position of these perturbations outside the SHL-2 binding site could be due to Chd1 action at SHL+2 on the opposite side, which would shift the perturbations toward SHL-3. Consistent with this possibility, both mismatch and insertion libraries also produced nucleosomes where Chd1 shifted the dyad toward the TA-rich side of the 601 sequence (dyad[+20]), and thus acted at the TA-rich SHL+2 (Figure 7 and Supplementary Figure S14). In many cases, the canonical 601 dyad[0] position was shifted by Chd1 to both dyad[-20] and dyad[+20], evidence of remodeler action on both sides (e.g. mismatches between -11 and -4, and insertions between -6 and -4, Supplementary Tables S4–S7). The shift toward the TA-rich side corresponded with DNA perturbations that would shift over to SHL-2 on the TA-poor side upon Chd1 acting at SHL+2. As seen from the heatmaps, a similar behavior of shifting toward the TA-rich side was also apparent in the poly(dA:dT) library (Figure 3A). However, the nucleosome dyad patterns for the poly(dA:dT) library were not as strongly affected as the mismatch and insertion libraries, perhaps reflecting the stronger or more localized impact of the mismatches/insertions on Chd1 activity.

### Nucleosome remodeling using a natural yeast positioning sequence

For nucleosome sliding experiments, the Widom 601 has the great advantage of favoring a unique dyad position. However, since the 601 is artificial, there is a possibility that some remodeling characteristics may not reflect the properties of nucleosomes made from natural sequences. To address this concern, we searched for a natural positioning sequence from *S. cerevisiae*, based on nucleosome dyad mapping carried out by Widom and colleagues (45). As we will describe in detail elsewhere, we identified the beginning of the SWH1 gene (+1 nucleosome position, Supplementary Table S1) as giving relatively unique nucleosome positioning *in vitro*. We therefore generated mismatch and single-nt insertion libraries for the SWH1 +1 sequence, and used Slide-seq to measure how these modifications affect nucleosome repositioning by Chd1.

**Figure 8.**
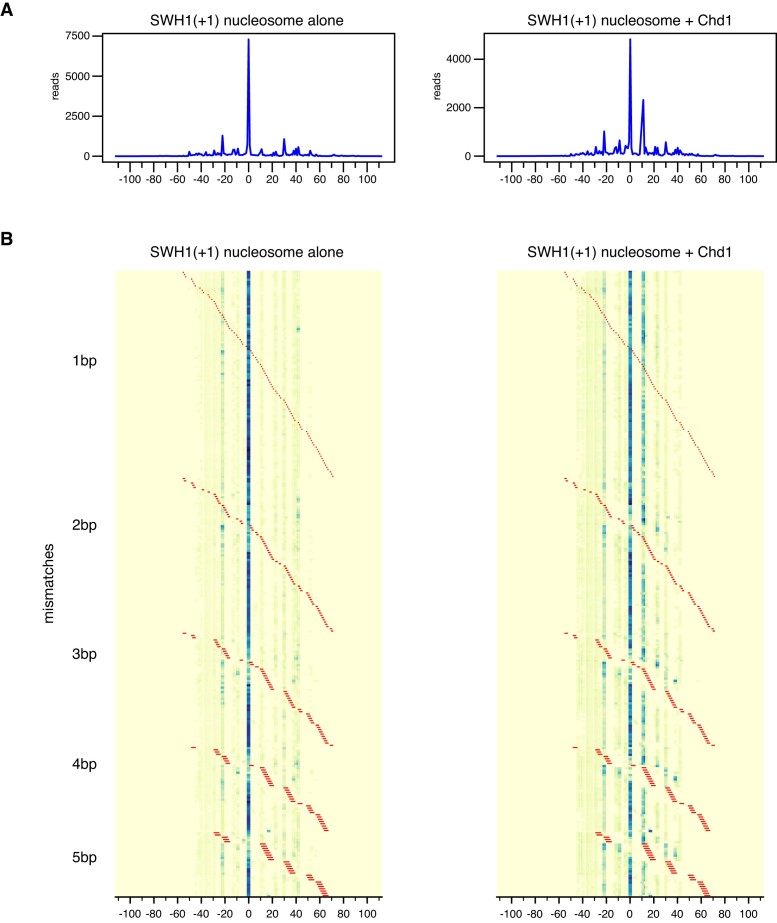
The +1 nucleosome position for the natural yeast sequence SWH1 yields a well-defined nucleosome position that can be altered by Chd1 and mismatches. Heat maps of calculated dyad positions for a SWH1 +1 library containing 1–5 mismatches. Left side shows nucleosome positions prior to remodeling by Chd1, right side after Chd1 + ATP. Sites and lengths of mismatches are indicated with red lines.

After salt dialysis and before sliding by Chd1, the SWH1 +1 sequence produced a nearly uniquely positioned nucleosome, comparable to the Widom 601 (Figure 8). As with the 601, nucleosome positioning with the SWH1 +1 sequence was most sensitive to mismatches and single-nt insertions within 20 bp of the dyad (Supplementary Figures S15−S16). After being shifted by Chd1, the SWH1 +1 nucleosomes preferentially occupied the original salt-dialyzed position, with a second peak 11 bp on one side. Although the majority of Chd1-treated nucleosomes remained at the original dyad location, the differences in nucleosome positioning due to mismatches and insertions suggested that nucleosomes were in a dynamic equilibrium. For example, nucleosomes with DNA perturbations initially positioned between the dyad and SHL+/-2 (e.g. I_1_[13^14], I_1_[14^15], and M_2_[-10:-9]) showed a redistribution that suggested Chd1 action on each side (Supplementary Figures S17-S18, Supplementary Tables S8-S11). In these cases, the nucleosome redistribution can be understood by Chd1 action at one SHL2 that would shift these perturbations onto the opposite SHL2, interfering with back-and-forth sliding.

With the SWH1 +1 sequence, we observed that DNA perturbations had a similar effect as for the 601 sequence, where nucleosome positions after sliding were influenced by perturbations outside the Chd1 binding site. In particular, even though insertions at I_1_[26^27] and I_1_[30^31] are outside the binding site, they prevented Chd1 from generating the preferred +11 shift (Supplementary Figures S17-S18, Supplementary Tables S8-S11). Similarly, the +11 species was enriched with insertions starting at SHL-2 (I_1_[-21^-20] to I_1_[-17^-16]), on the opposite side from where Chd1 acts to shift DNA to the dyad[+11] position. After an +11 bp shift, these insertions would instead be located around SHL-3, between -27 to -32 bp from the dyad, which would not be expected to strongly interfere with a shift back to the starting dyad[zero] position. Thus, the final distribution of nucleosome positions was clearly impacted by such DNA perturbations lying outside the Chd1 binding site.

### A kinetic model for nucleosome repositioning by Chd1

To better understand the impact of unfavorable sequences/disruptions on nucleosome positioning, we developed a kinetic model for back-and-forth nucleosome sliding. Based on a general master equation, our model assumes that nucleosomes shift in single-bp steps, with the distribution of nucleosome positions arising from the rates at which DNA shifts between neighboring positions. The rates are explicitly defined by three factors: the free energy available from ATP hydrolysis, the energy required to distort DNA (nucleosome sliding intermediate), and the sequence-dependent energy landscape of DNA wrapped around the histone core (see Methods). The energy from ATP, here estimated to be ∼20 k_B_T (34), is much higher than the other two factors. However, the driving force of remodeling from ATP at the two SHL2 sites is equivalent; therefore, the other two terms – the energy of DNA distortion and sequence-dependent wrapping – dominate to determine the final nucleosome positions. That is, if at one SHL2 site the energy required to distort DNA prior to sliding is high, nucleosome sliding will preferentially take place at the other SHL2, thus shifting DNA in the opposite direction.

**Figure 9.**
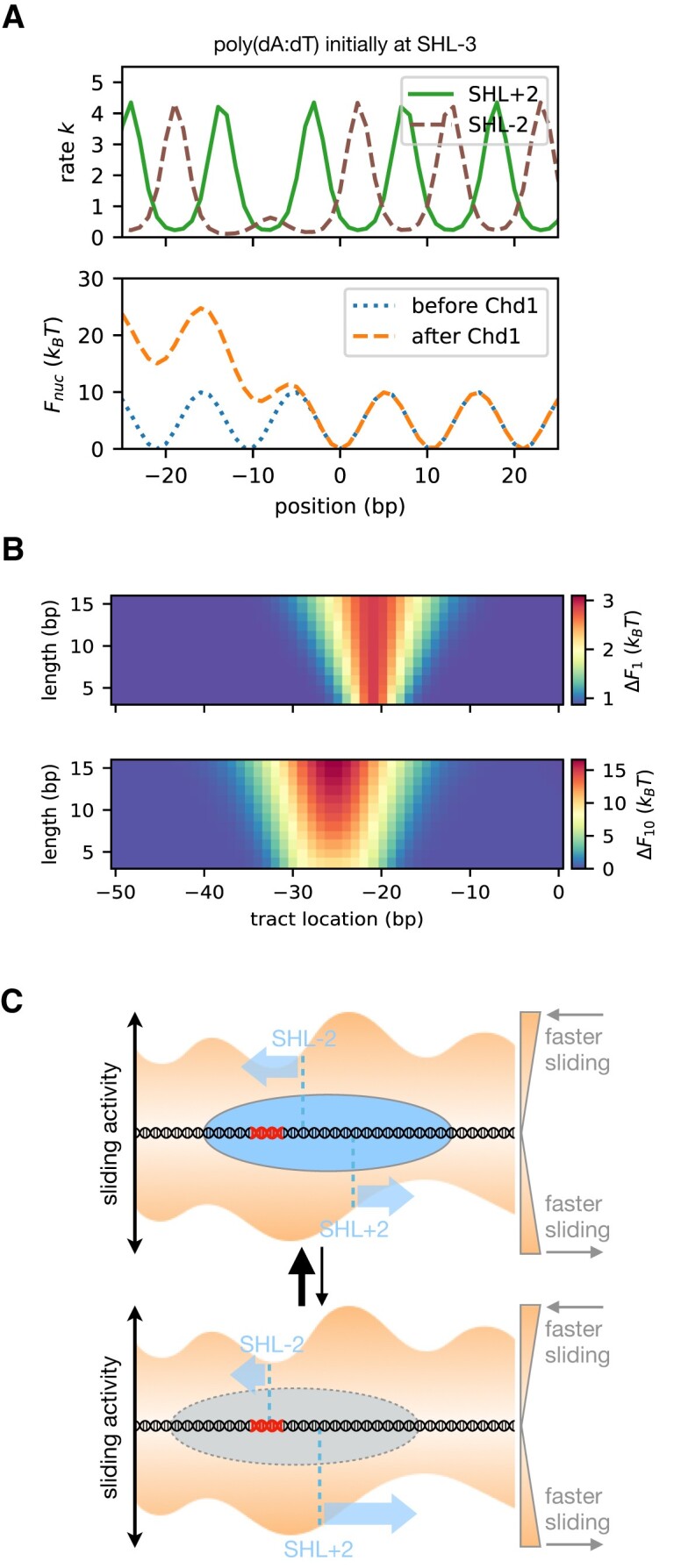
A kinetic model of nucleosome remodeling by Chd1. (**A**) On the top, the rate of nucleosome sliding due to Chd1 binding at either SHL+2 (green, solid line) or SHL-2 (brown, dashed line) as a function of nucleosome position when a ∼10 bp poly(dA:dT) tract is initially located at −30 bp from dyad (SHL-3). On the bottom, the original 601 nucleosome free energy *F*(*i*) (dotted, blue line), and the effective nucleosome free energy after remodeling by Chd1 *F*_eff_(*i*) (dashed, orange line). (**B**) The effective free energy difference due to Chd1 remodeling by 1 bp (top) or by 10 bp (bottom) as a function of poly(dA:dT) tract initial location x (horizontal axis) and length L (vertical). The energy of sliding by 1 bp mainly takes into account the difference in the cost of defects at SHL+/-2 in the initial nucleosome configuration, whereas sliding by 10 bp takes into account this bias over the course of the entire 10 bp range. **(C)** Schematic model of dynamic nucleosome repositioning by Chd1, shown with nucleosome positions (ovals) overlaid on energy landscapes for forward (top) and reverse (bottom) sliding. Each SHL2 site is marked by a vertical dotted line, with the height reflecting the relative rate. Stable nucleosome positions (top) are expected where rates on both sides are similar, with the rates for sliding toward the site being faster than the rates for sliding away from it. A variety of DNA perturbations (in red) may be responsible for decreasing the rate of Chd1-driven sliding from a given side of the nucleosome, and destabilize certain nucleosome positions (bottom).

A surprising conclusion of this model is how positioning is influenced by the energy landscape of the nucleosome positioning sequence. To better match the tight wrapping of DNA around the histone core, strong positioning sequences have a periodic nature, with DNA sequence motifs that favor narrow minor grooves (inside of wrap) alternating with those that favor wider minor grooves (outside of wrap). Strong positioning sequences therefore produce a sinusoidal nucleosome energy landscape with ∼10 bp periodicity; shifting DNA by ∼5 bp would be most costly, as it would shift sequences favoring narrow grooves to the outside and vice versa. As shown in Figure 9A, the rate of nucleosome sliding by Chd1 oscillates with the strong phasing of the 601 sequence. With a poly(dA:dT) tract initially located at SHL-3, the sliding rate for a remodeler at SHL-2 is strongly reduced as the tract encroaches into the SHL-2 binding site (top panel). Although the poly(dA:dT) tract does not directly inhibit remodeling until the DNA shifts ∼10 bp (when the tract will be at SHL-2), the rotational preference of the 601 positioning sequence and the bi-directional action of Chd1 collectively inhibit sliding in the negative direction to reach the next stable nucleosome position at –10 bp (bottom panel). When the cost of a barrier such as a poly(dA:dT) tract is considered in 1 bp steps, the inhibitory effect on nucleosome sliding is localized at SHL-2 (Figure 9B, top panel). However, the cost of the barrier becomes amplified and expanded in the final effective free energy landscape in combination with a strong nucleosome positioning sequence, because the preferred nucleosomal DNA phasing only occurs every ∼10 bp (Figure 9B, bottom panel). Thus, the requirement for cumulative ∼10 bp sliding events, which is imposed by the sinusoidal landscape of positioning sequences, broadens and extends the effect of a barrier up to SHL-3.

In the model, although barriers (such as poly(dA:dT) tracts) initially have no effect on remodeling when located at SHL-3, they slow down sliding after a ∼10 bp shift to SHL-2. The instability of a ∼10 bp shift due to a barrier located at SHL-3 (outside the Chd1 binding site) can be understood as resulting from the competition between the activities of the remodeler at the two SHL2 binding sites: after a ∼10 bp shift, the barrier will be located at SHL-2, slowing further sliding by Chd1 binding there, but the remodeler can still bind and act at SHL+2 on the opposite side, sliding DNA so that the barrier is pushed back to its original position. Our model, consistent with our biochemical findings, supports the idea that nucleosomes preferentially populate away from positions where remodeling activity differs greatly at the two SHL2 sites. Instead, nucleosomes are expected to favor sites where remodeling activity is similar at both SHL2 sites (Figure 9C). If the nucleosome is displaced from the favored site, the imbalance in the remodeling activity at the two SHL2 sites would bring it back.

## DISCUSSION

By combining experiments, MD simulations, and theory, this work reveals how nucleosome sliding by Chd1 integrates site-specific DNA perturbations that directly affect the Chd1 ATPase motor with sequence-dependent phasing of DNA on the histone core. Although Chd1 sliding activity was most strongly blocked when localized perturbations were located at SHL+/-2, nucleosome positioning with 601 libraries showed that interfering elements (poly(dA:dT) tracts, mismatches, single-nucleotide insertions) were also found around SHL-3. Consistent with the 601 libraries, nucleosome positioning also showed displacement of interfering elements toward SHL+/-3 for the natural SWH1 sequence. These nucleosome distributions, with interfering elements outside the Chd1 binding site, can be explained by remodeler action on both sides of the nucleosome. If one side of the nucleosome has a barrier for remodeler action—whether a less accessible binding site or a higher cost of forming an intermediate structure—then remodeler action will be favored on the other side. Since action on each side shifts DNA in opposite directions on the histone core, remodeler action at one SHL2 site will push interfering sequences from the opposite SHL2 toward the neighboring SHL3. Thus, the most favored nucleosome positions are not necessarily those where, due to interfering sequences at one SHL2, action of a chromatin remodeler is low on one side. Instead, a favored nucleosome position can arise from sliding at the opposite SHL2, which shifts interfering sequences back toward SHL3 (Figure 9C).

Our model suggests that the impact of interfering elements on nucleosome positions are amplified by the preferred phasing of strong nucleosome positioning sequences. Strong nucleosome positioning sequences like the Widom 601 are predicted to have an energy landscape that oscillates with a ∼10 bp frequency (33,46). With such oscillations, shifting a DNA sequence away from its preferred phasing will be energetically uphill for ∼5 bp, whereas a return toward the preferred phasing will be downhill. When located around SHL3, an interfering element extends the uphill disadvantage from strong phasing, giving an energetic advantage to remodeling from the opposite SHL2 over a broader range. In our experiments, displacement of interfering elements toward SHL3 was observed not only with the Widom 601, but also the natural SWH1 +1 nucleosome sequence. Intrinsically, the SWH1 +1 sequence predominantly yields a single dyad position, supporting the idea that any strong positioning sequence can extend barrier effects of DNA sequences/elements that interfere with remodeler action.

Although all chromatin remodelers share a common Snf2-type ATPase motor (47), when given nucleosomes with the same sequences, different classes of remodelers produce distinctly repositioned products (48). These different responses presumably reflect how each remodeler class is uniquely able to connect action of its ATPase motor with sequence features in and around the nucleosome. The Chd1 remodeler is known to slide nucleosomes containing poly(dA:dT) tracts more slowly (18), and our work here suggests that this response occurs when the poly(dA:dT) tract overlaps with the ATPase binding site at SHL2. In particular, our MD simulations suggest that the ability of poly(dA:dT) tracts to affect Chd1 activity is likely due to the destabilization of intermediate DNA distortions necessary for sliding, a previously proposed mechanism (26,27). Thus, Chd1 appears to use its ATPase motor to directly respond to sequence-dependent properties of DNA at the SHL2 binding site. Although many remodelers also engage SHL2 with their ATPase motor, Chd1 is monomeric. In contrast, most other remodelers are multisubunit assemblies, with auxiliary subunits providing different avenues for directing ATPase-driven remodeling activity. One notable example is the INO80 remodeler, which is responsible for *in vivo* positioning of +1 nucleosomes (49). The ATPase motor of INO80, which engages nucleosomes around SHL6/7 (50,51) and hexasomes around SHL2/3 (52,53), appears to be controlled through the Arp8 module, which senses the sequence-dependent shape and mechanics of DNA > 30 bp away (54) to define the nucleosome depleted region (55,56).

This work reports that the yeast SWH1 +1 positioning sequence yields highly uniform nucleosome positions *in vitro*, and thus is an excellent substrate for studying nucleosome repositioning. For both SWH1 +1 and Widom 601 sequences, nucleosome positions were most disrupted by mismatches and single-nucleotide insertions around the dyad. The importance of the histone-DNA contacts at the nucleosome dyad agrees with previous experiments that identified the dyad as the most energetically important (57). Intrinsic nucleosome positioning therefore relies on sequence elements in different locations around the nucleosome from those that guide remodeler action. For the case of SWH1 +1, the intrinsically preferred position was largely maintained after nucleosome sliding by Chd1, suggesting that in this case, both SHL2 sites have sequences that are relatively unfavorable for nucleosome sliding. Beyond the +1 nucleosome, positions of nucleosomes further into gene bodies have been shown to correlate with sites of higher DNA flexibility (55), which are known to favor nucleosomes (17,37,58,59). An exciting question for future studies will be exploring how nucleosome positions with other natural DNA sequences may coordinate intrinsic preferences for histone positioning with symmetry of remodeler action.

## Supplementary Material

gkad738_Supplemental_files

## Data Availability

Sequencing data has been deposited in the GEO database with accession number GSE198440.

## Notes

Present address: Sangwoo Park, Dana-Farber Cancer Institute and Harvard Medical School, Boston, MA 02215, USA.

Present address: Taekjip Ha, Program in Cellular and Molecular Medicine, Boston Children’s Hospital, Boston, MA 02115, USA; Department of Pediatrics, Harvard Medical School, Boston, MA 02115, USA.
