# Supplementary material for: Bi-directional nucleosome sliding by the Chd1 chromatin remodeler integrates intrinsic sequence-dependent and ATP-dependent nucleosome positioning
Source: Nucleic Acids Res. 2023 Sep 20;51(19):10326–43. doi: 10.1093/nar/gkad738 (PMC10602870; doi:10.1093/nar/gkad738)
Supplement: gkad738_Supplemental_files [file gkad738_supplemental_files.zip › Park-SupplementaryFiguresplusLegends-2023july28a.pdf]

SUPPLEMENTARY FIGURES

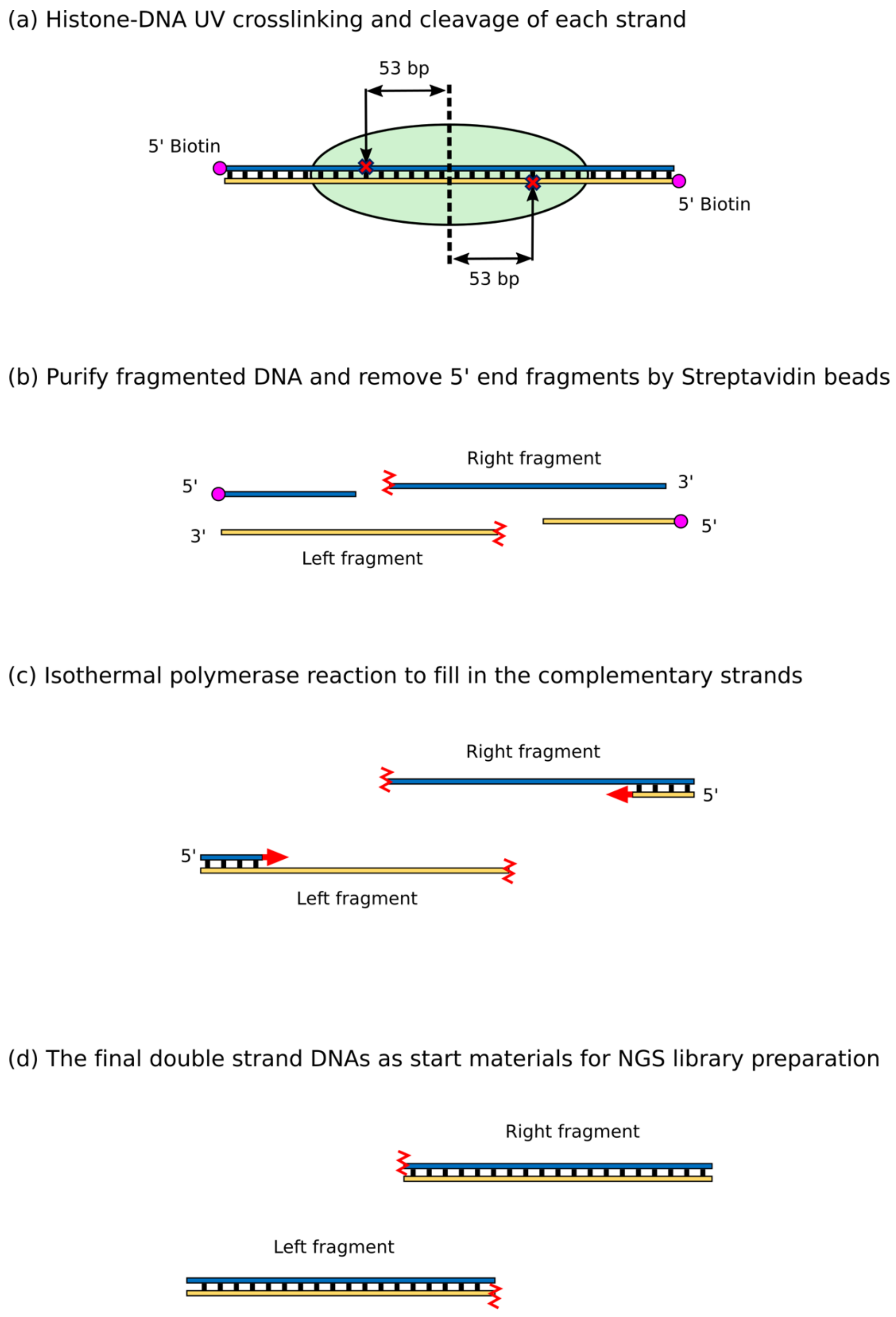

Supplementary Figure S1. Overview of key steps in the slide-seq protocol.

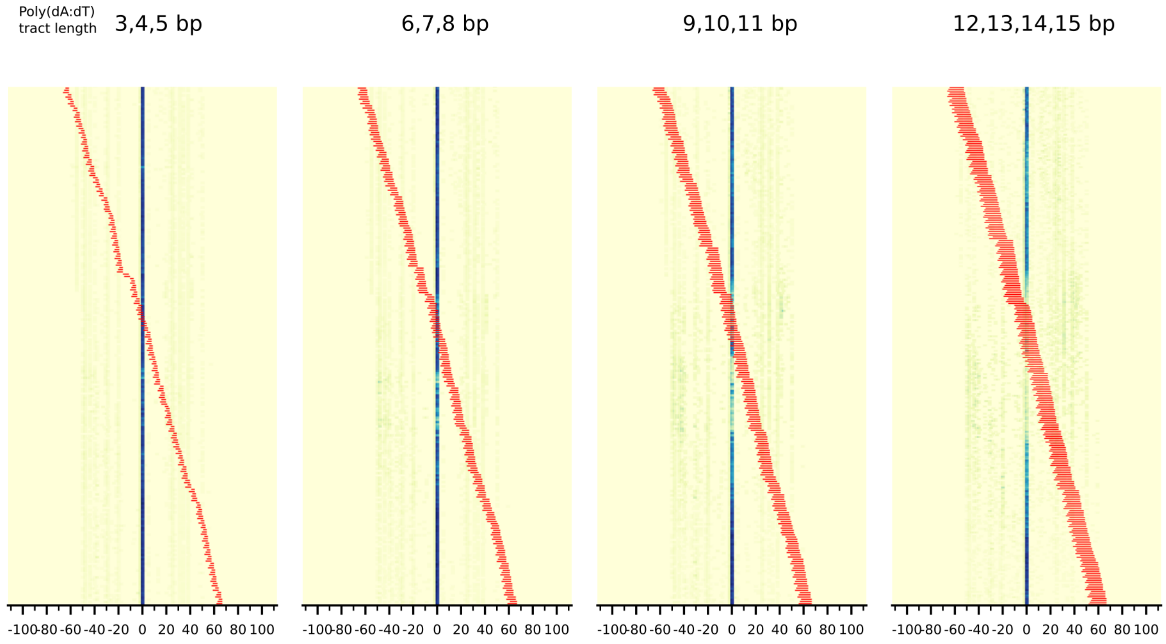

Supplementary Figure S2. Heatmaps for estimated nucleosome positioning signals on the 601 poly(dA:dT) library before sliding.

The sequences were grouped according to the lengths and locations of poly(dA:dT) tracts (red).

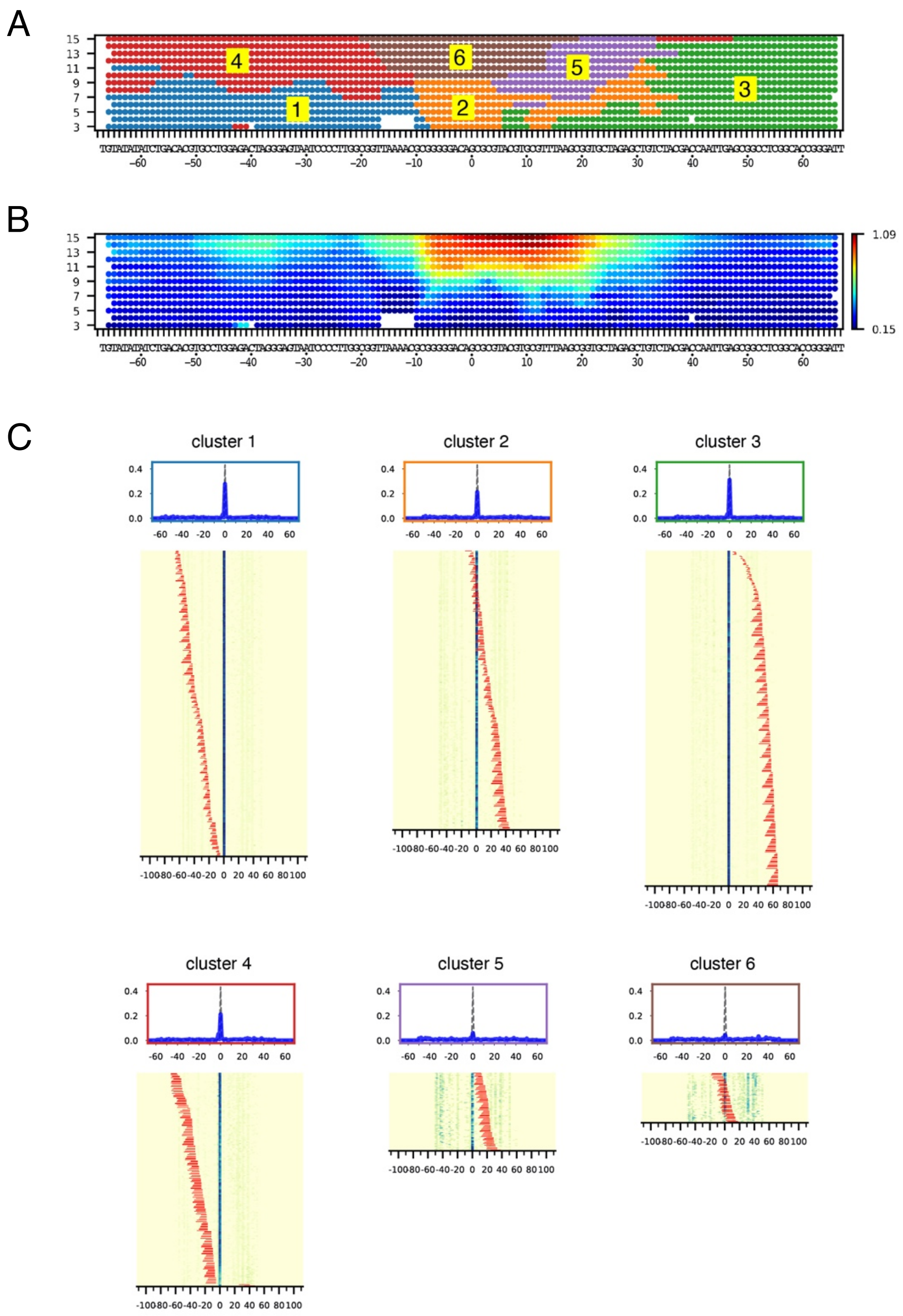

Supplementary Figure S3. Clustering analysis of the nucleosome positioning on the 601 poly(dA:dT) library before sliding by Chd1.

(A) Based on the similarity of nucleosome positions and perturbation locations, poly(dA:dT) library data was clustered into 6 groups. (B) A KL-divergence heatmap shows the most sensitive area in the Widom 601 DNA by perturbations. (C) Dyad positions for each poly(dA:dT) tract are shown as heatmaps according to each cluster. Red bars indicate the length and position of each tract.

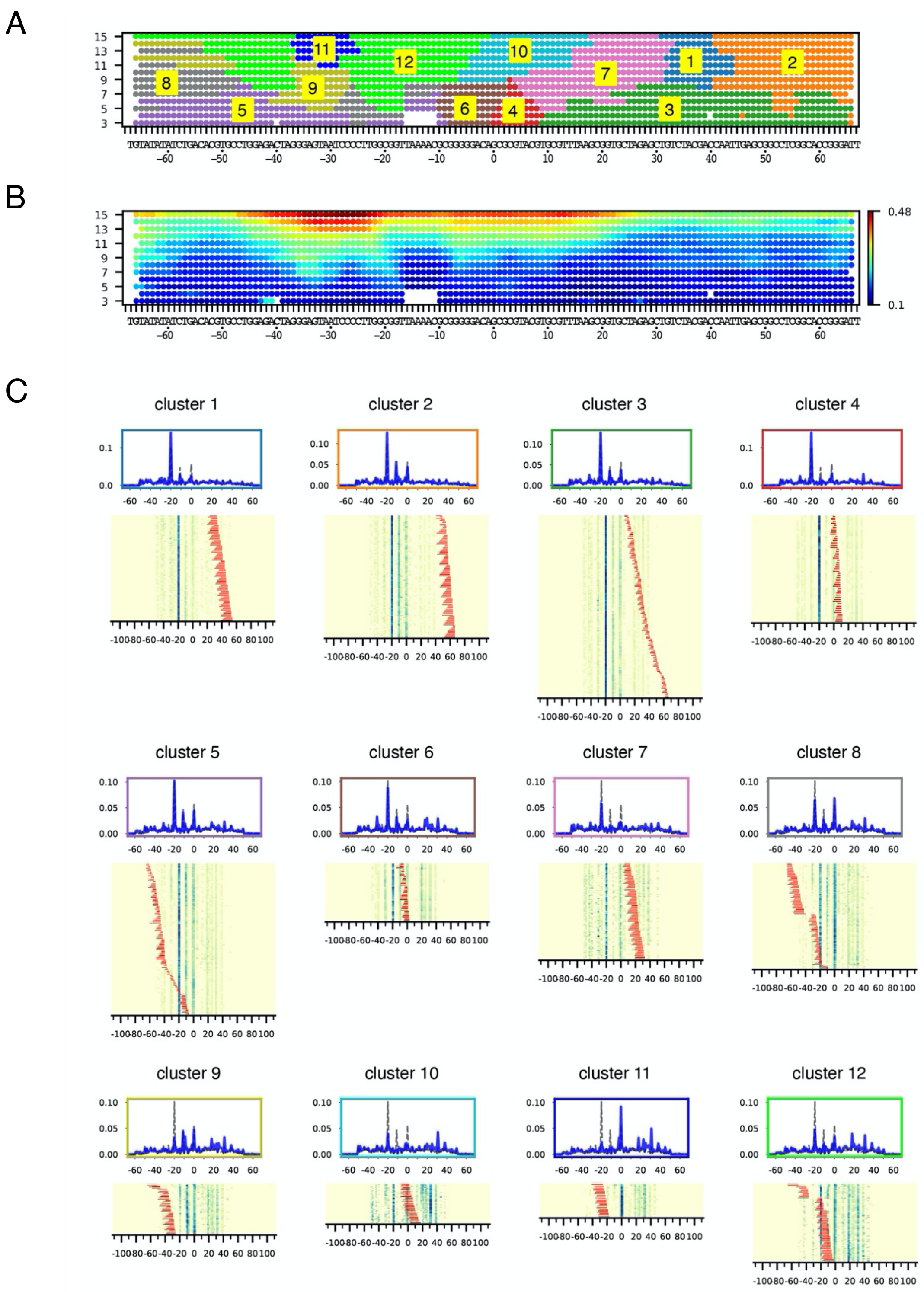

Supplementary Figure S4. Clustering analysis of the 601 poly(dA:dT) library based on nucleosome positions after sliding by Chd1.

(A) The poly(dA:dT) library was clustered into 12 groups after sliding by Chd1. (B) A KL-divergence heatmap shows the positions and lengths of poly(dA:dT) tracts that most strongly altered the dyad pattern after Chd1 sliding. (C) Heatmaps of dyad positions grouped according to each cluster.

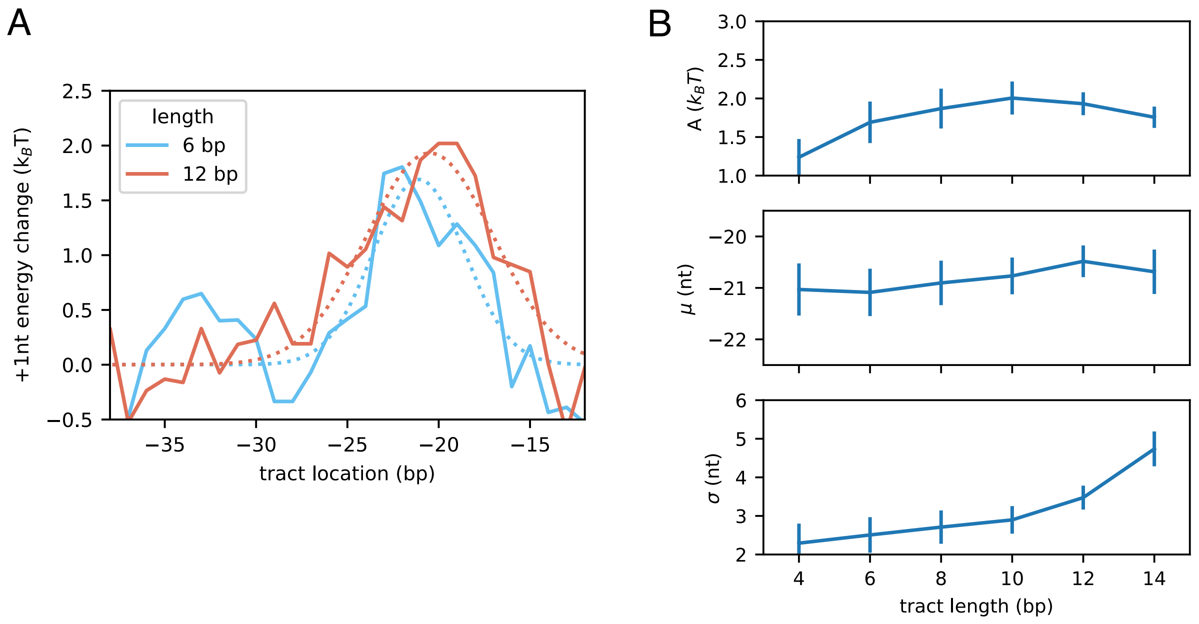

Supplementary Figure S5. Dependence of defect energies with poly(dA:dT) tracts.

(A) Gaussian fits (dashed lines) to the +1 nt defect free energies (solids lines) relative to the original 601 cost as a function of poly(dA:dT) tract location k for tract lengths of 6 bp and 12 bp: $\Delta F_{d+/-2}(k) = A \exp(-(k-\mu)^2/2\sigma^2)$. (B) Summary of the Gaussian fits as a function of the poly(dA:dT) tract length, showing the Gaussian height A (top), mean μ (center) and standard deviation σ (bottom).

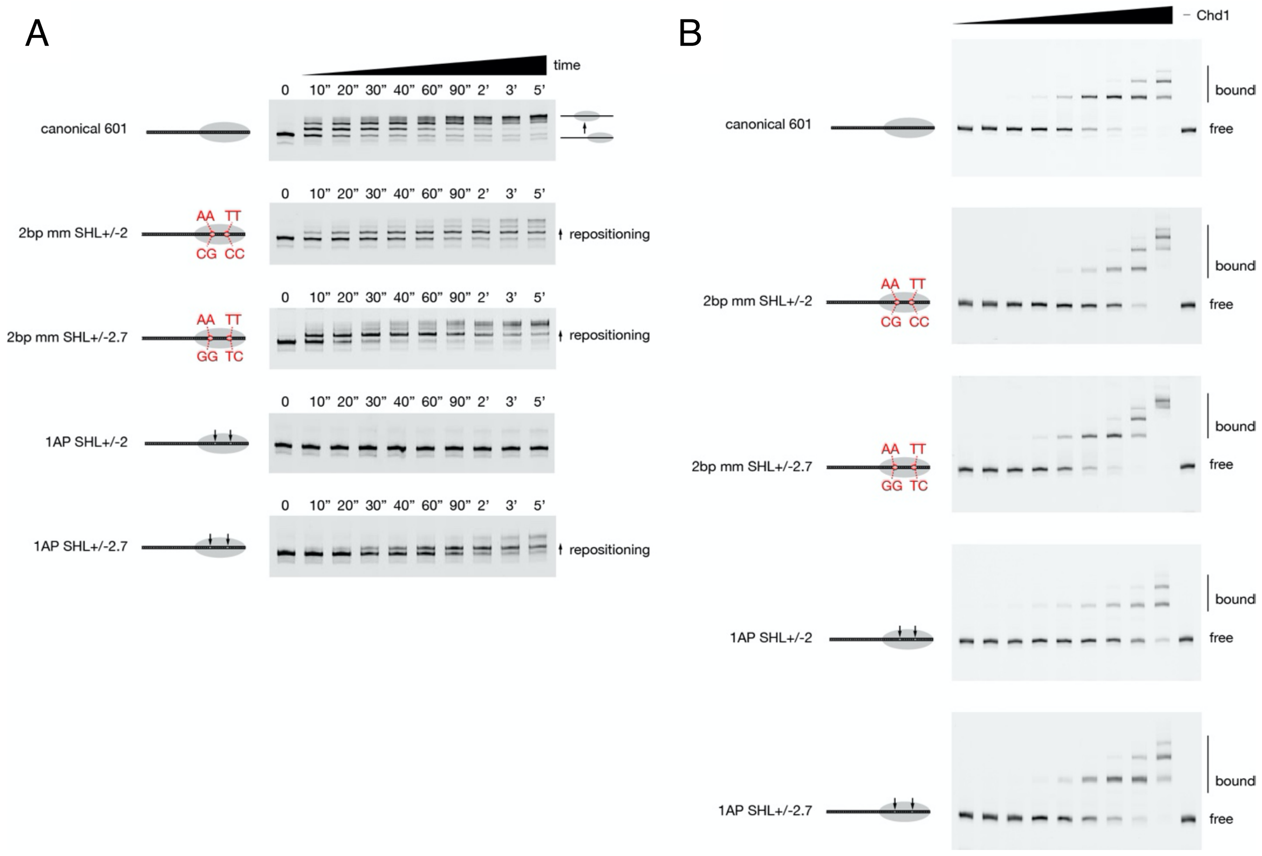

Supplementary Figure S6. Effects of DNA perturbations on nucleosome sliding and binding by Chd1.

(A) Representative native acrylamide gels for nucleosome sliding reactions. Sliding experiments contained 50 nM nucleosome, 100 nM Chd1, and 2 mM ATP. In each case, flanking linker DNA (80 bp) was only on one side, and perturbations were generated on both sides of the nucleosome, as indicated. Nucleosome centering is indicated by slower migration of nucleosomes.

(B) Nucleosome binding reactions, using the same substrates as shown in (A). For each experiment, Chd1 was titrated in the following concentrations: 12.8 pM, 64 pM, 320 pM, 1.6 nM, 8 nM, 40 nM, 200 nM, 1 μM, 2 μM. Reactions also contained 1 μg/μl salmon sperm DNA and 2 mM AMP-PNP.

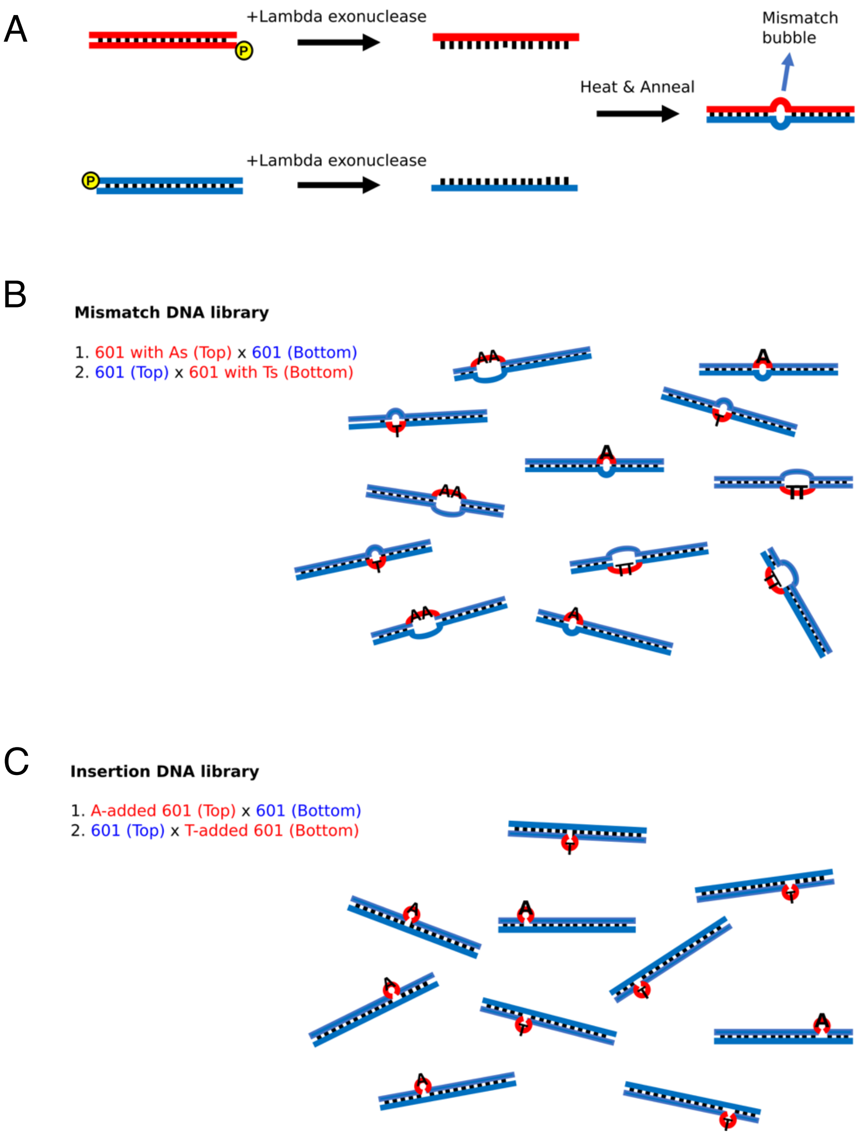

Supplementary Figure S7. Overview of the strategy for generating the mismatch and single-insertion DNA library.

(A) To generate mismatches or single-nucleotide insertions, the two strands of the duplex were produced in two separate reactions (red and blue). Lambda exonuclease, which has preferred activity toward strands with phosphorylated 5' ends, was then added to selectively eliminate one strand. When the original templates differ at defined positions, annealing the resulting single strands produced duplexes containing mismatches or insertions. (B) The 601 mismatch library is the mixture of two mismatch types: Adenine (A) substitutions in the top 601 strand paired with the original 601 bottom strand, and Thymine (T) substitutions in the bottom 601 strand paired with the original 601 top strand. (C) The 601 insertion library is a mixture of two insertion types: Adenine (A) insertions in the top 601 strand paired with the original 601 bottom strand, and Thymine (T) insertions in the bottom 601 strand paired with the original 601 top strand.

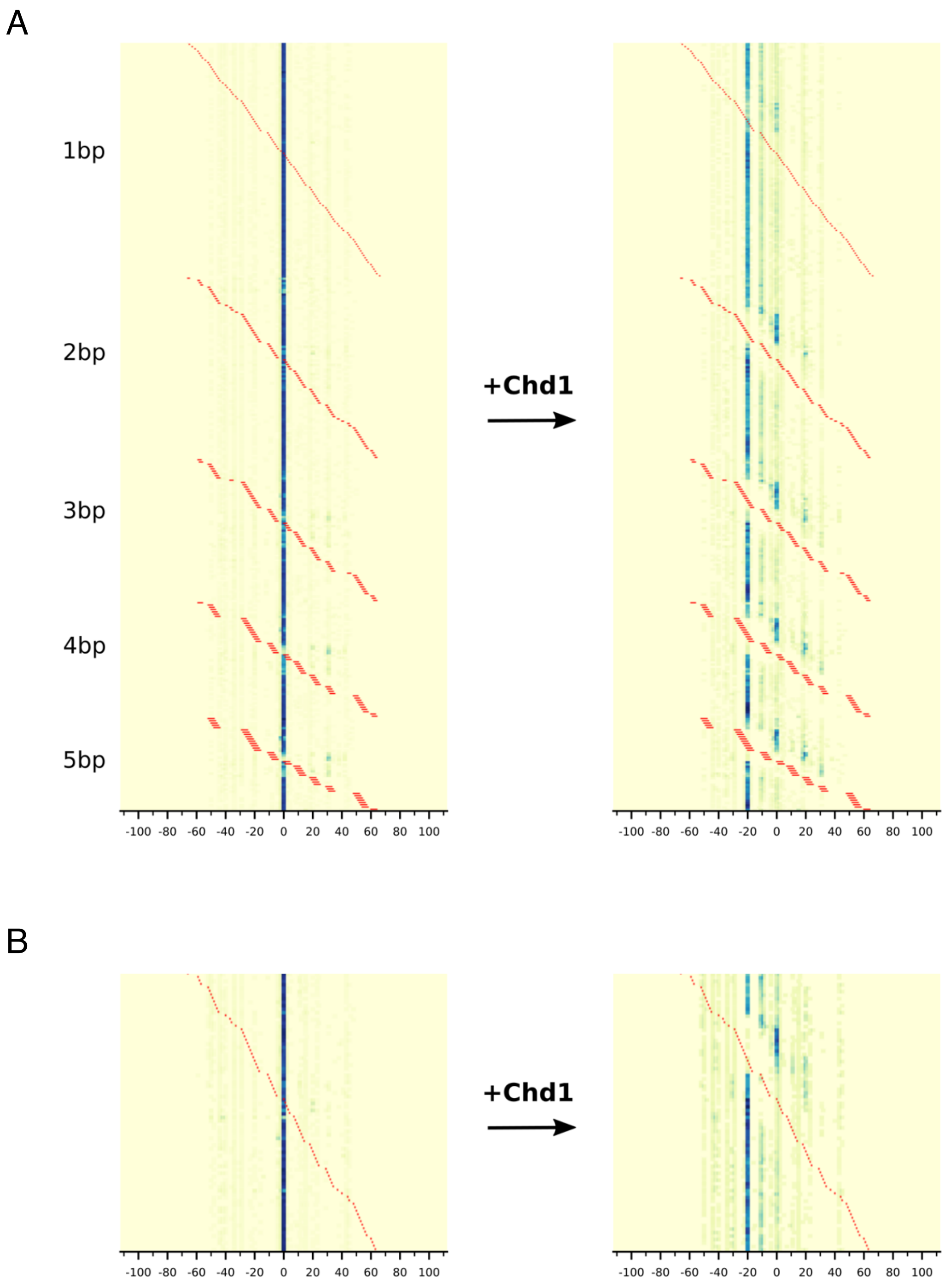

Supplementary Figure S8. Heatmaps of dyad positions for 601 mismatch and insertion libraries, before and after sliding by Chd1.

(A) Heatmaps of the 601 mismatch library, grouped by mismatch position and length. (B) Heatmaps of the 601 single-insertion library, ordered by position. The location of mismatches and insertions are indicated by red bars.

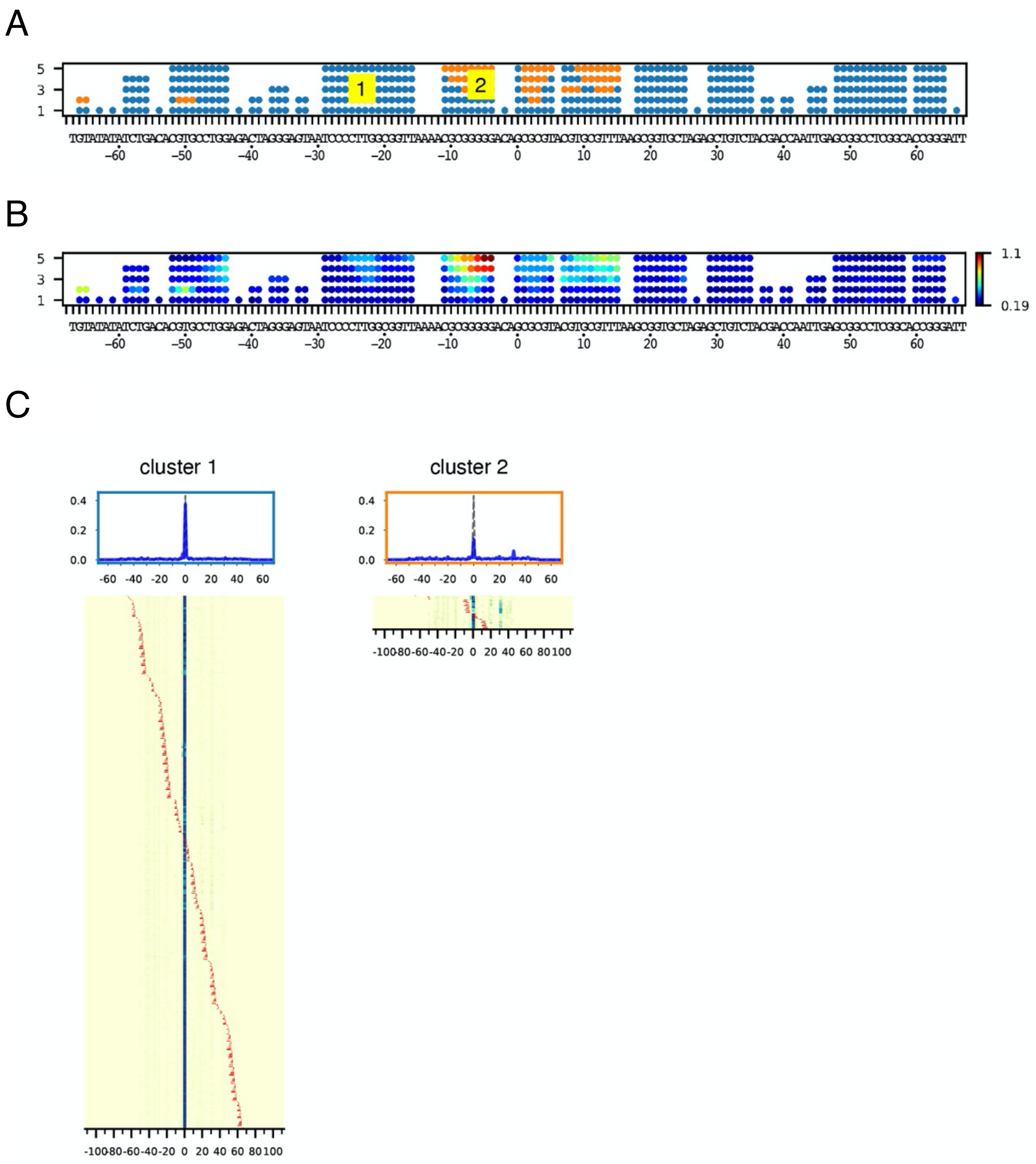

Supplementary Figure S9. Clustering of 601 mismatch library, before sliding by Chd1.

(A) Based on the similarity of dyad positions and distributions, the 601 mismatch library data was clustered into 2 groups. (B) A KL-divergence heatmap shows the most sensitive area in the Widom 601 DNA by perturbations. (C) Dyad positions for each mismatch are shown as heatmaps according to each cluster. Red bars indicate the length and position of mismatches.

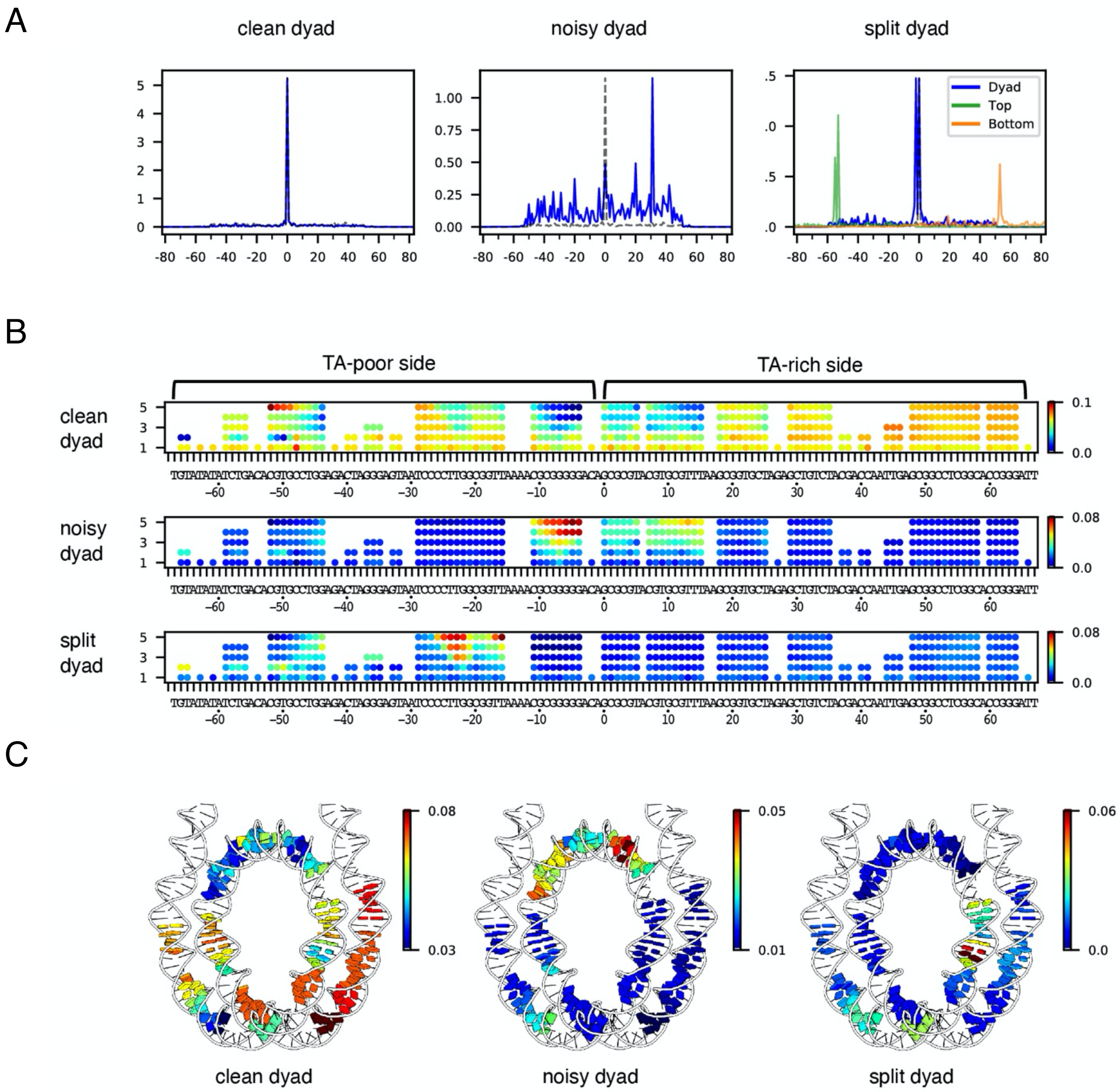

Supplementary Figure S10. NMF analysis for nucleosome positioning on the 601 mismatch library before sliding by Chd1.

(A) Through NMF analysis, all nucleosome positioning data was linearly decomposed into three basis patterns: clean dyad, noisy dyad, and split dyad. Calculated dyad positions are shown in blue, and sites of H2B(S53C) cross-linking are shown in orange and green. The canonical 601 dyad position is indicated by a dotted line. The y-axes are $x10^3$ reads. (B) For each basis pattern, the corresponding NMF scores were mapped onto the Widom 601 sequence using the color scale as indicated. The y-axes indicate the number of mismatches (C) The NMF scores for 3 bp mismatches were mapped onto a nucleosome structure (6WZ5).

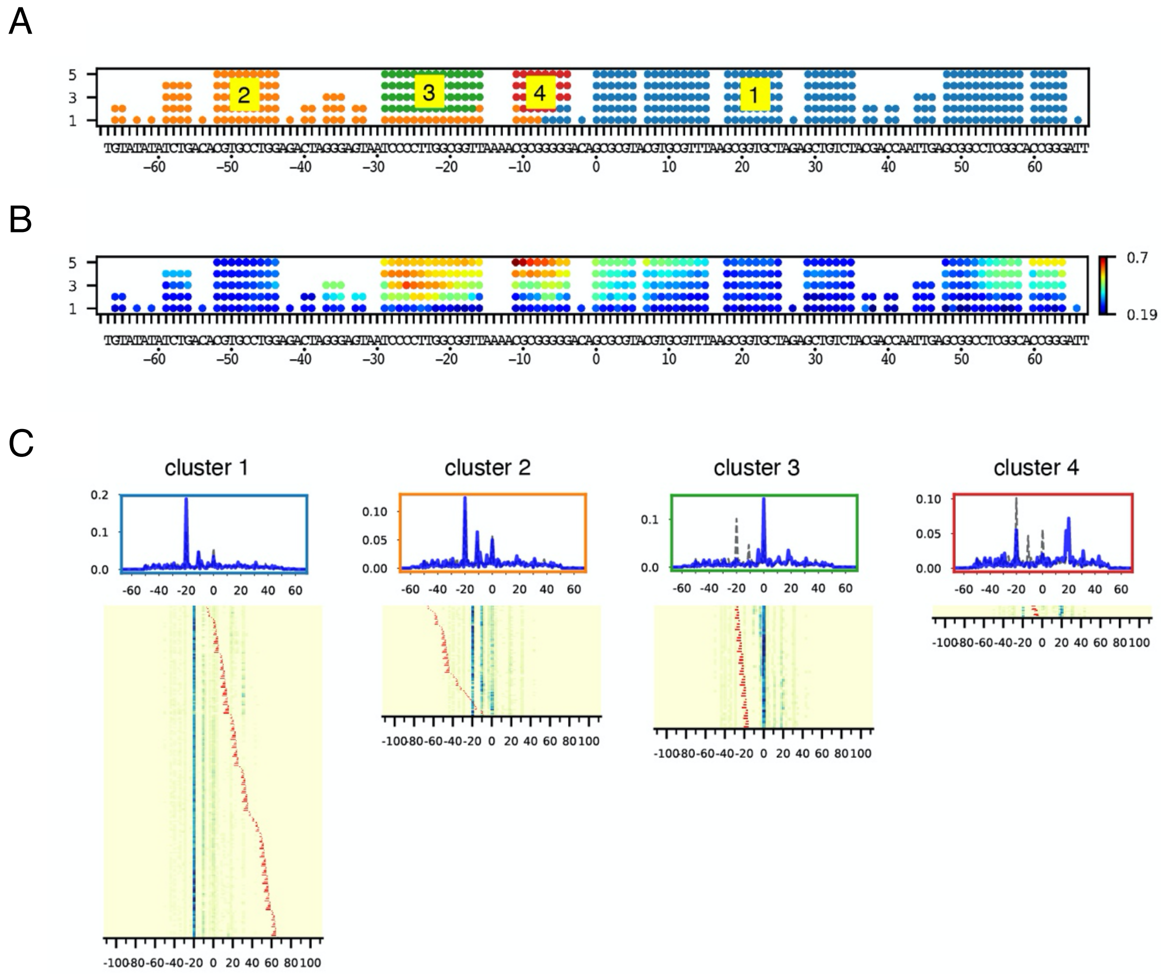

Supplementary Figure S11. Clustering analysis of the 601 mismatch library after sliding by Chd1.

(A) The mismatch library was clustered into 4 groups after sliding by Chd1. (B) A KL-divergence heatmap shows the positions and lengths of mismatches that most strongly altered the dyad pattern after Chd1 sliding. Y-axes indicate number of mismatches. (C) Heatmaps of dyad positions grouped according to each cluster. In top panels, y-axes are x10^3 reads

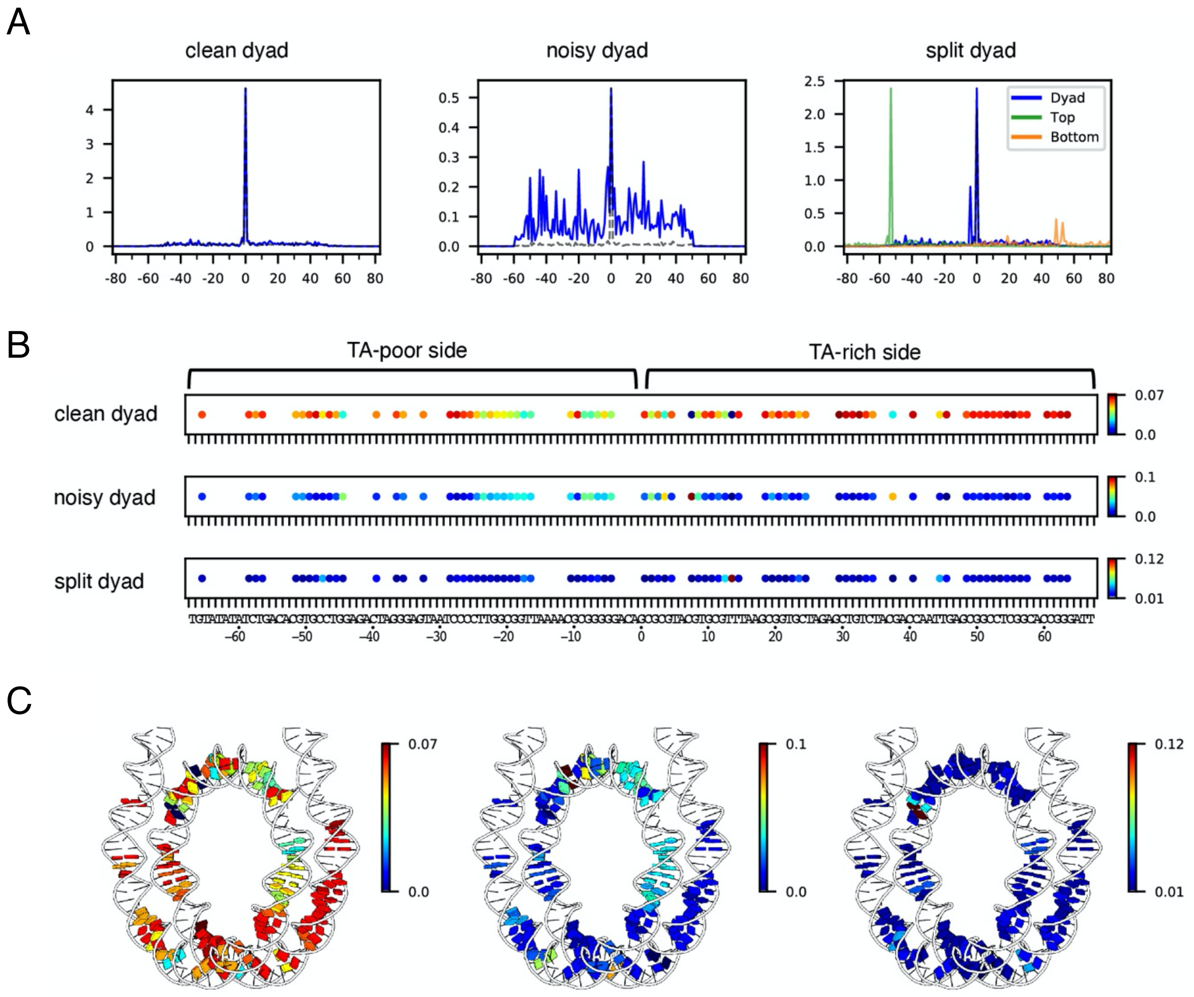

Supplementary Figure S12. NMF analysis for nucleosome positioning on insertion 601 library before sliding by Chd1.

(A) Through NMF analysis, nucleosome positioning data was li nearly decomposed into three basis patterns: clean dyad, noisy dyad, and split dyad. Calculated dyad positions are shown in blue, and sites of H2B(S53C) cross-linking are shown in orange and green. The canonical 601 dyad position is indicated by a dotted line. The y-axes are x10^3 reads. (B) For each basis pattern, the corresponding NMF scores were mapped onto the Widom 601 sequence using the color scale as indicated. (C) The NMF scores were mapped onto a nucleosome structure (6WZ5).

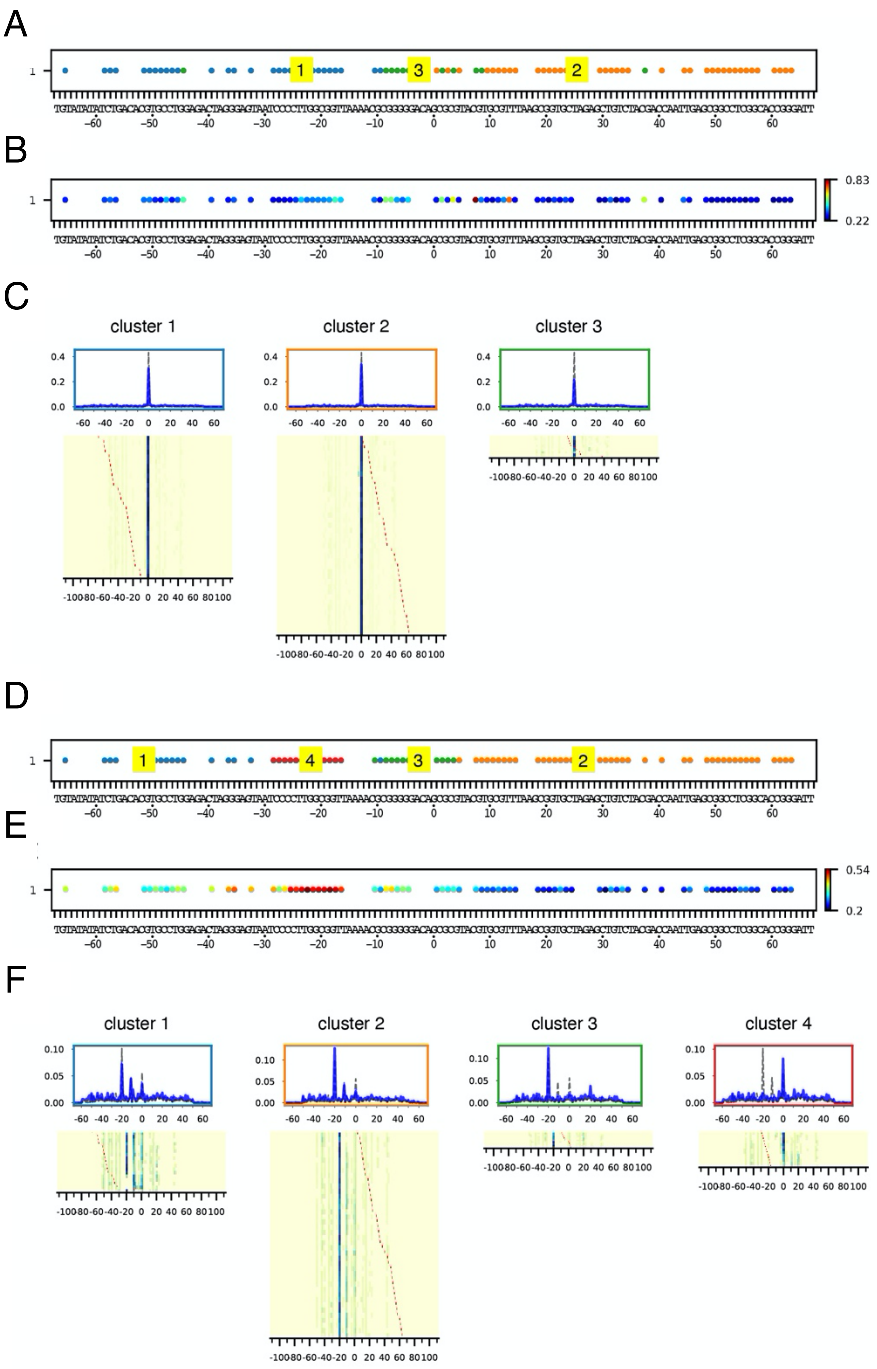

Supplementary Figure S13. Clustering analysis of the 601 insertion library before and after sliding by Chd1.

Through Spectral clustering analysis, the data were grouped and mapped on the Widom 601 before sliding (A) and after sliding (D) by Chd1. KL-divergence maps are show before sliding (B) and after sliding (E). For each cluster, the average positioning signal and data heatmap is shown for before sliding (C) and after sliding (F) by Chd1. The y-axes are x10^3 reads.

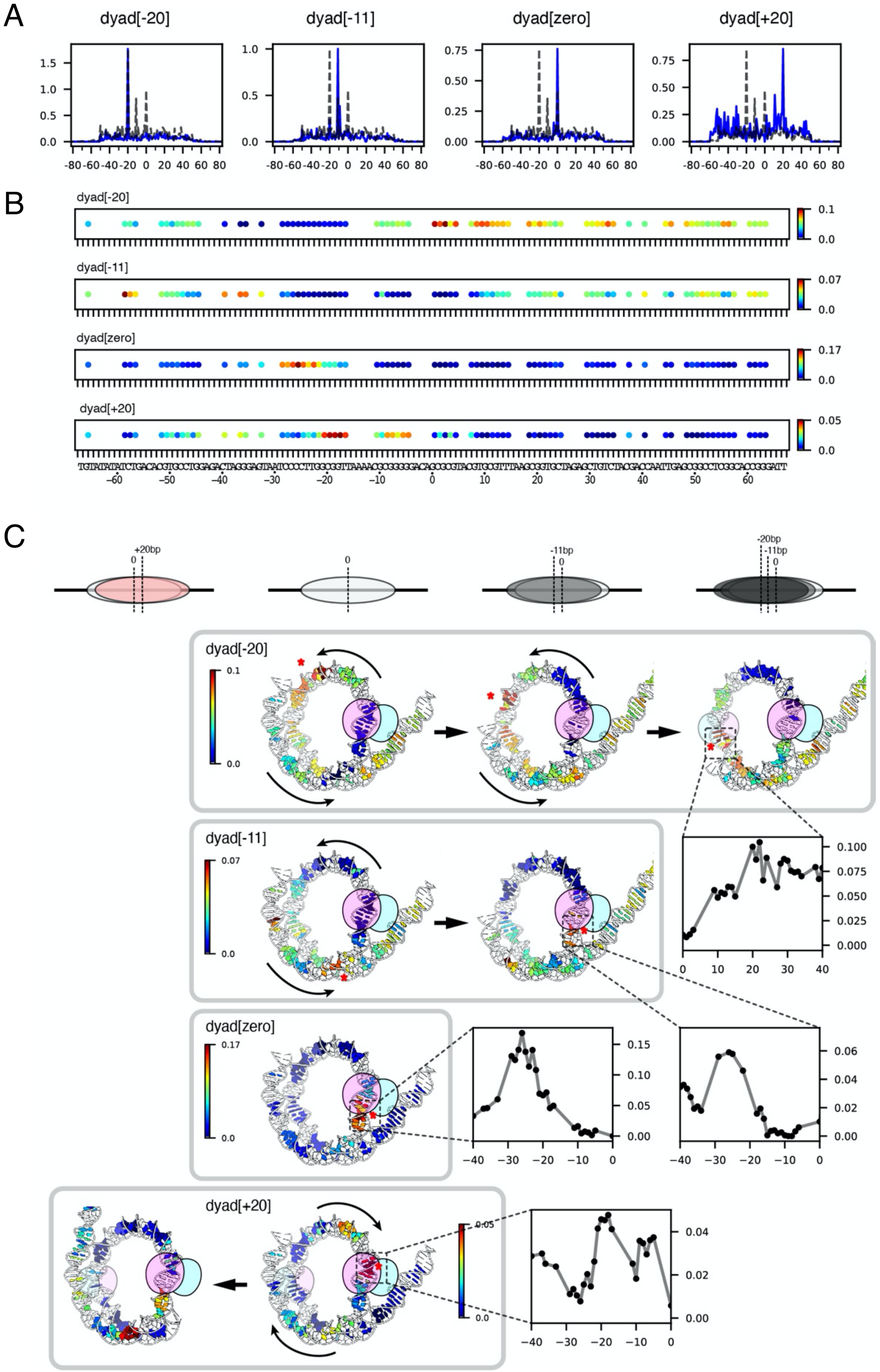

Supplementary Figure S14. Single-nucleotide insertions alter the distribution of 601 nucleosomes repositioned by Chd1.

(A) Four basis dyad patterns observed after sliding by Chd1, which were used for NMF scoring (blue). The distribution of the canonical 601 is shown with dotted lines. The y-axes are x10^3 reads. (B) NMF scores mapped onto the Widom 601 DNA sequence, using the color scale as indicated, based on the dyad[-20], dyad[-11], dyad[zero], and dyad[+20] patterns. (C) NMF scores mapped onto a Chd1-nucleosome complex (7TN2) at different translational positions, as shown in Figure 4. Asterisks highlight prevalent insertion sites (high NMF scores), and where these sites would be repositioned after nucleosome sliding.

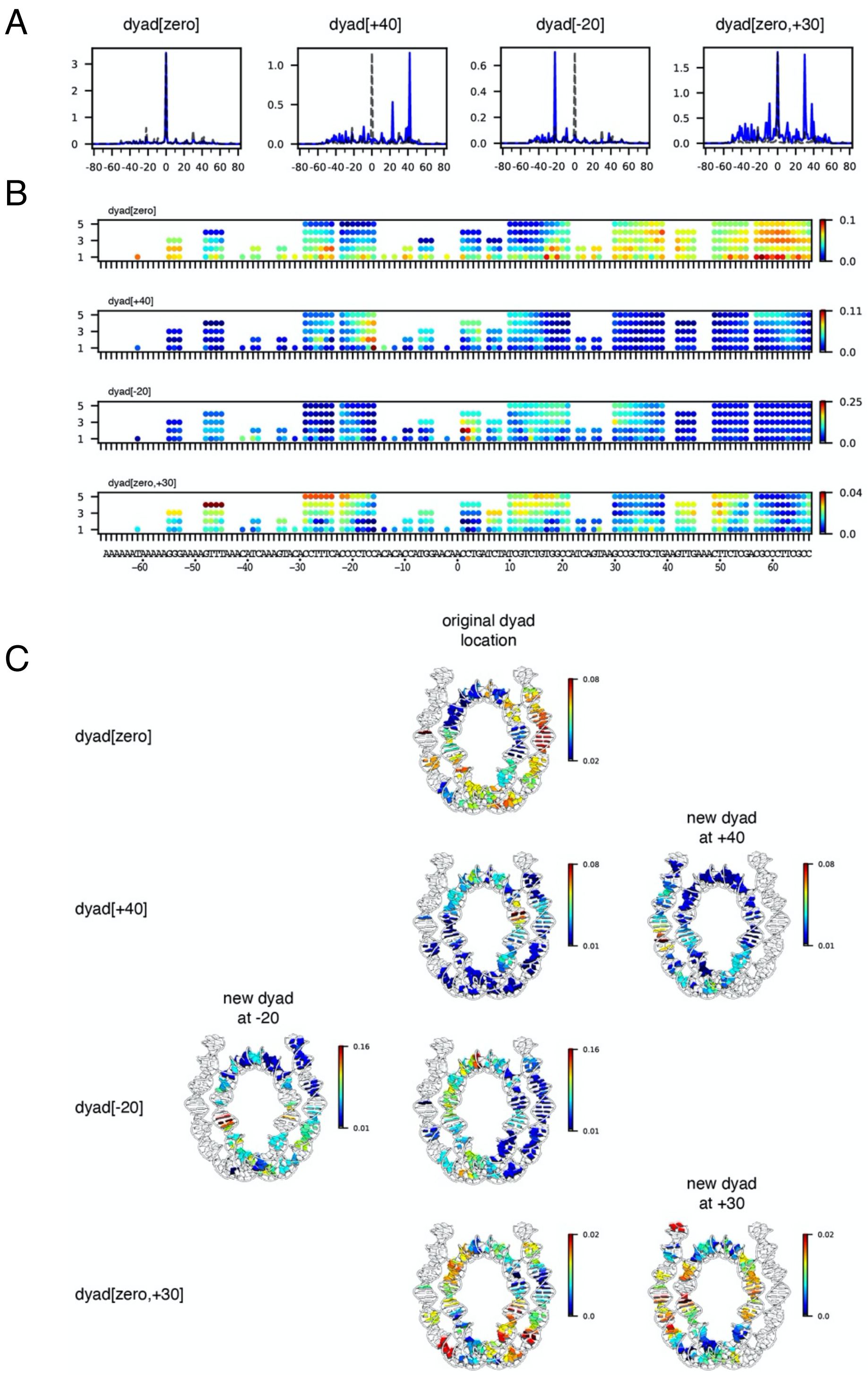

Supplementary Figure S15. NMF analysis for nucleosome positioning on the SWH1 +1 mismatch library before sliding by Chd1.

(A) Through NMF analysis, all nucleosome positioning data is linearly decomposed into four basis patterns. The canonical SWH1 +1 dyad position is indicated by a dotted line. For each plot, the y-axis indicates number of reads ($x10^3$) and the x-axis is the nucleotide position relative to the dyad. (B) For

each basis pattern, the corresponding NMF scores were mapped onto the SWH1 +1 sequence using the color scale as indicated. (C) The NMF scores were mapped onto a nucleosome structure (6WZ5). For off-center positions, the sequence has been shifted so that the dyad matches with the major observed nucleosome position (+40, -20, and +30).

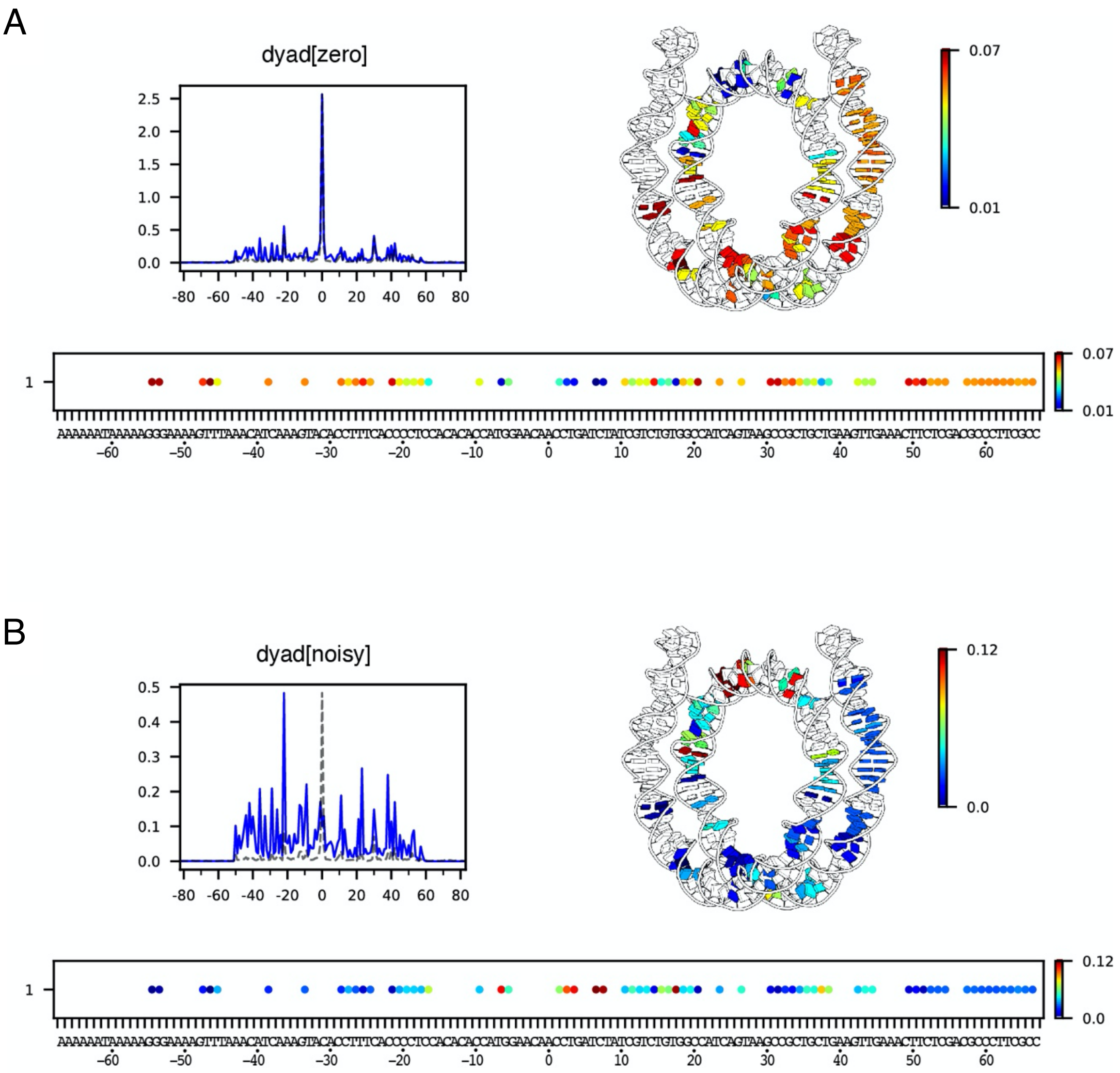

Supplementary Figure S16. NMF analysis for nucleosome positioning on the SWH1 +1 insertion library before sliding by Chd1.

Through NMF analysis, all nucleosome positioning data is linearly decomposed into two basis patterns, shown in (A) and (B). For each, the canonical SWH1 +1 dyad position is indicated by a dotted line. The y-axes are x10^3 reads. NMF scores were mapped onto the SWH1 +1 sequence and a nucleosome structure (6WZ5).

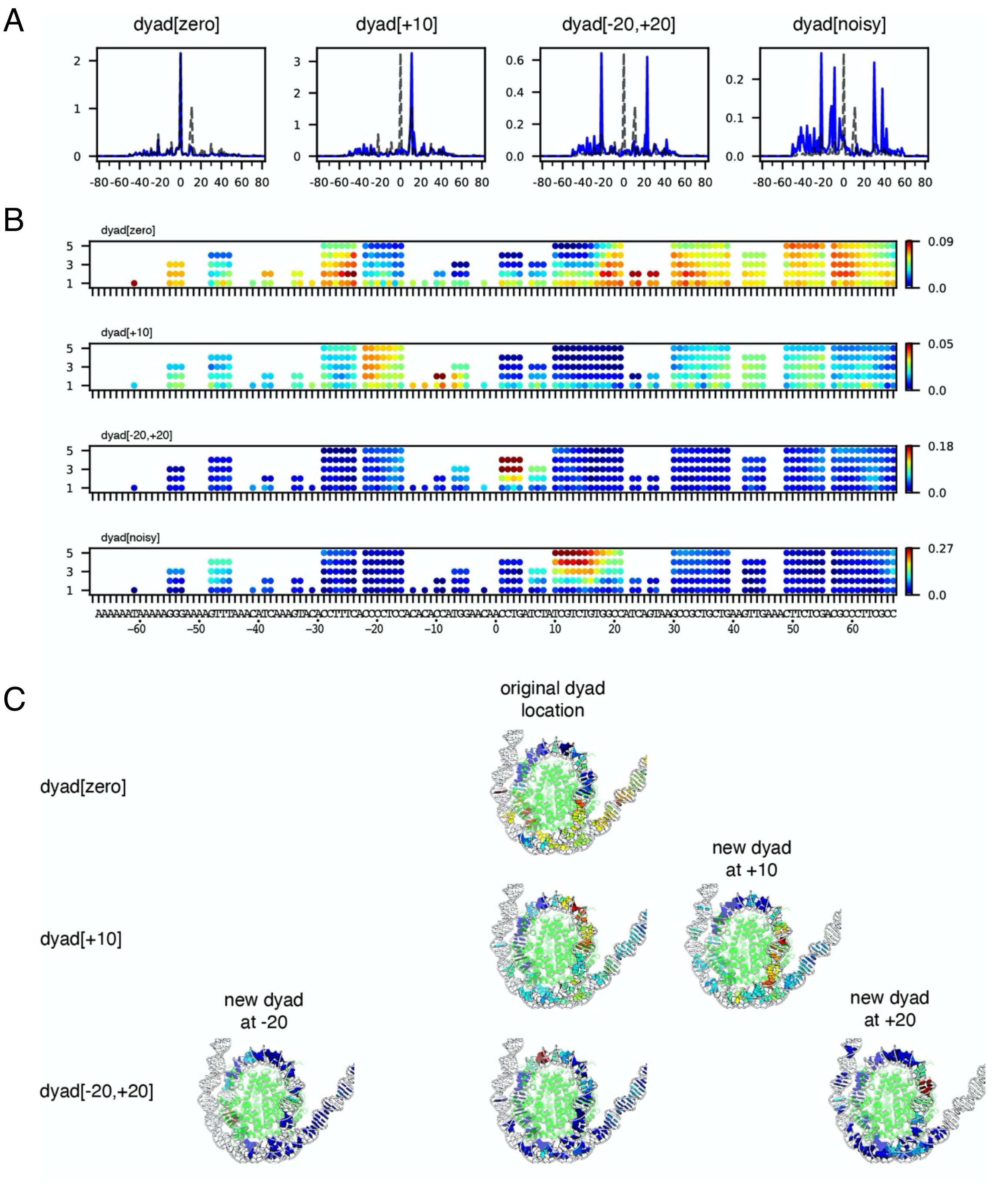

Supplementary Figure S17. NMF analysis for nucleosome positioning on the SWH1 +1 mismatch library after sliding by Chd1.

(A) Through NMF analysis, all nucleosome positioning data was linearly decomposed into four basis patterns. The canonical SWH1 +1 dyad position is indicated by a dotted line. The y-axes are x10^3 reads. (B) For each basis pattern, the corresponding NMF scores were mapped onto the SWH1 +1 sequence. (C) The NMF scores were mapped onto a Chd1-nucleosome structure (7TN2). For off-center positions, the sequence has been shifted so that the dyad matches with the major observed nucleosome position.

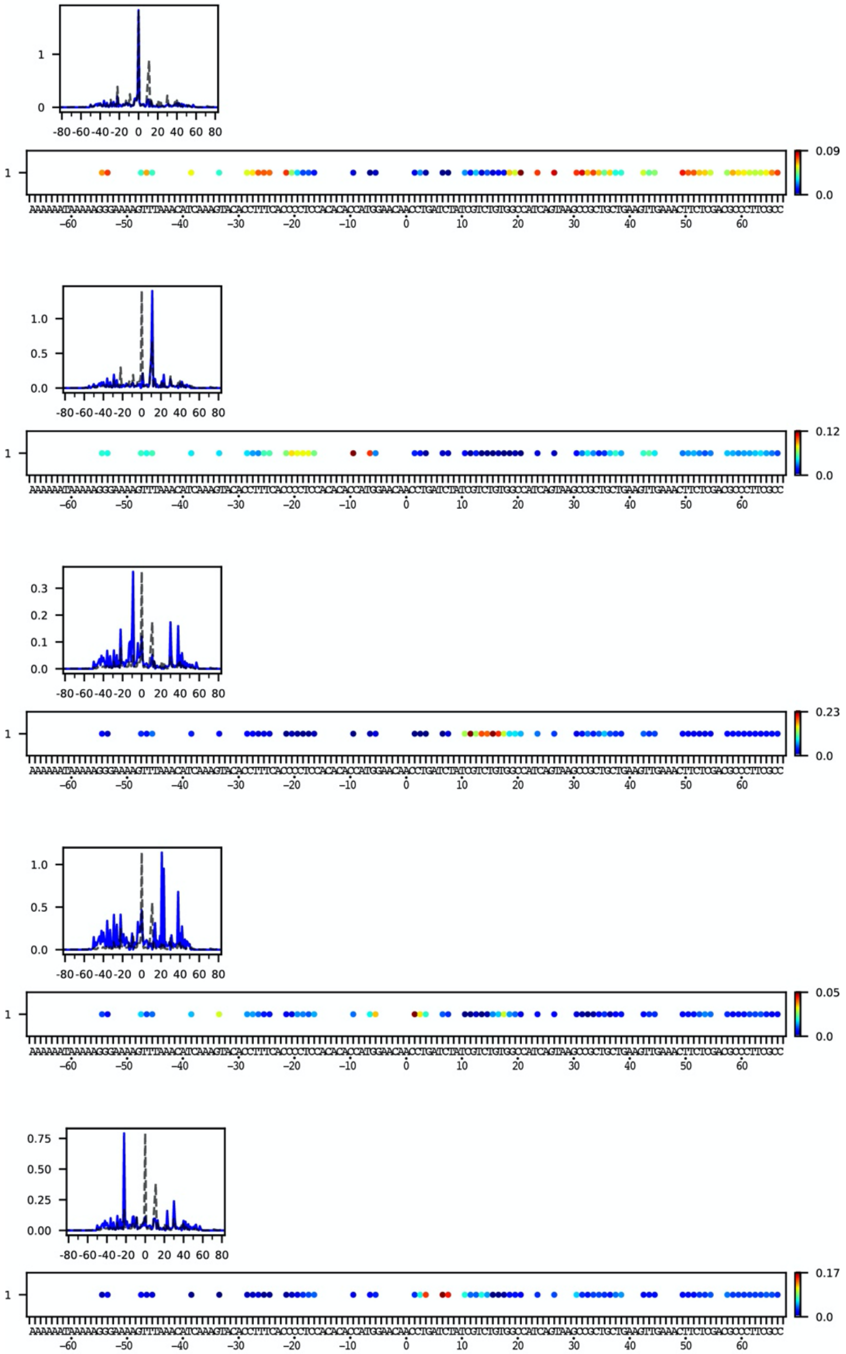

Supplementary Figure S18. Single-nucleotide insertions alter the distribution of SWH1 +1 nucleosomes repositioned by Chd1.

Five basis dyad patterns observed after sliding by Chd1, which were used for NMF scoring (blue). The distribution of the unmodified SWH1 +1 dyads is shown with dotted lines. The y-axes are x10^3 reads.
