# Supplementary material for: Bi-directional nucleosome sliding by the Chd1 chromatin remodeler integrates intrinsic sequence-dependent and ATP-dependent nucleosome positioning
Source: Nucleic Acids Res. 2023 Sep 20;51(19):10326–43. doi: 10.1093/nar/gkad738 (PMC10602870; doi:10.1093/nar/gkad738)
Supplement: gkad738_Supplemental_files [file gkad738_supplemental_files.zip › SupplementaryTable1-DNAinformation-2023july28a.pdf]

Supplementary Table 1. DNA sequences used in this study

DNA

Original 601 sequence

top strand (linker DNA, dyad)

5’ ATCCGACTGG CACCGGCAAG GTCGCTGTTC AATACATGCA CAGGATGTATATATCTGACACGTGCCTGGAGACTAGGGAGTAATCCCCTTGGCGGTTAAAACGCGGGGGACA G
CGCGTACGTGCGTTTAAGCGGTGCTAGAGCTGTCTACGACCAATTGAGCGGCCTCGGCACCGGGATTCTCCA GGGCGGCCGC GTATAGGGTC CATCACATAA GGGATGAACT 3’

bottom strand (linker DNA, dyad)

3’ TAGGCTGACC GTGGCCGTTC CAGCGACAAG TTATGTACGT GTCCTACATATATAGACTGTGCACGGACCTCTGATCCCTCATTAGGGGAACCGCCAATTTTGCGCCCCCTGT C
GCGCATGCACGCAAATTCGCCACGATCTCGACAGATGCTGGTTAACTCGCCGGAGCCGTGGCCCTAAGAGGT CCCGCCGGCG CATATCCCAG GTAGTGTATT CCCTACTTGA 5’

601-based libraries – backbone template

top strand (red are changes from original linkers)

5’ ATCCGACTGGCACCGGCAAGGTCGCTGT TCGCCACATGCG CAGGATGTATATATCTGACACGTGCCTGGAGACTAGGGAGTAATCCCCTTGGCGGTTAAAACGCGGGGGACA G
CGCGTACGTGCGTTTAAGCGGTGCTAGAGCTGTCTACGACCAATTGAGCGGCCTCGGCACCGGGATTCTCCA GGGCGTCCTCGT ATAGGGTCCATCACATAAGGGATGAACT 3’

primers for library amplification

Primer1: 5'- ATCCGACTGGCACCGGCAAGGTCGCTGT TCGCCACATGCG -3'

Primer2: 5'- AGTTCATCCCTTATGTGATGGACCCTAT ACGAGGACGCCC -3'

Primer1: 5’-/5Biosg//i6-FAMK/ ATCCGACTGGCACCGGCAAGGTCGCTGT TCGCCACATGCG -3’

Primer2: 5’-/5Biosg//iCy5/ AGTTCATCCCTTATGTGATGGACCCTAT ACGAGGACGCCC -3’

SWH1(+1)-based libraries – backbone template

top strand (linkers), 5’ to 3’

ATCCGACTGGCACCGGCAAGGTCGCTGTTCGCCACATGCG
AAAAAAAAAAATAAAAAGGGAAAAGTTTAAACATCAAAGTACACCTTTCACCCCTCCACACACCATGGAACA A CCTGATCTATCGTCTGTGGCCATCAGTAAGCCGCTGCTGAAGTTGAAACTTCTCGACGCCCTTCGCCAGGGA
GGGCGTCCTCGTATAGGGTCCATCACATAAGGGATGAACT

Supplementary Table 1. DNA sequences used in this study

0N80 601 mismatch and AP constructs

WT control, top strand (linker, SHL+/-2 and SHL+/-2.7 sites underlined)
5' CGGTACCCGG GGATCCTCTA GAGTGGGAGC TCGGAACACT ATCCGACTGG CACCGGCAAG GTCGCTGTTC AATACATGCA
CAGGATGTATATATCTGACACGTGCCTGGAGACTAGGGAGTAATCCCCTTGGCGGTTAAAACGCGGGGGACA G CGCGTACGTGCGTTTAAGCGGTGCTAGAGCTGTCTACGACCAATTGAGCGGCCTCGGCACCGGGATTCTCCA3'

2bp mismatch SHL+/-2 (AA/TT)
CAGGATGTATATATCTGACACGTGCCTGGAGACTAGGGAGTAATCCCCTTG**AA**GGTTAAAACGCGGGGGACA G CGCGTACGTGCGTTTAAGC**TT**TGCTAGAGCTGTCTACGACCAATTGAGCGGCCTCGGCACCGGGATTCTCCA

2bp mismatch SHL+/-2.7 (AA/TT)
CAGGATGTATATATCTGACACGTGCCTGGAGACTAGGGAGTAATC**AA**CTTGGCGGTTAAAACGCGGGGGACA G CGCGTACGTGCGTTTAAGCGGTGCT**TT**AGCTGTCTACGACCAATTGAGCGGCCTCGGCACCGGGATTCTCCA

AP construct designs

*1AP SHL+/-2 (**x**), top strand (5′ to 3′)*
CAGGATGTATATATCTGACACGTGCCTGGAGACTAGGGAGTAATCCCCTTGG**x**GGTTAAAACGCGGGGGACA G CGCGTACGTGCGTTTAAGC**x**GTGCTAGAGCTGTCTACGACCAATTGAGCGGCCTCGGCACCGGGATTCTCCA

*1AP SHL+/-2 (**x**), bottom strand (5′ to 3′)*
TGGAGAATCCCGGTGCCGAGGCCGCTCAATTGGTCGTAGACAGCTCTAGCAC**x**GCTTAAACGCACGTACGCG C TGTCCCCCGCGTTTTAACC**x**CCAAGGGGATTACTCCCTAGTCTCCAGGCACGTGTCAGATATATACATCCTG

*1AP SHL+/-2.7 (**x**), top strand (5′ to 3′)*
CAGGATGTATATATCTGACACGTGCCTGGAGACTAGGGAGTAATC**x**CCTTGGCGGTTAAAACGCGGGGGACA G CGCGTACGTGCGTTTAAGCGGTGCTA**x**AGCTGTCTACGACCAATTGAGCGGCCTCGGCACCGGGATTCTCCA

*1AP SHL+/-2.7 (**x**), bottom strand (5′ to 3′)*
TGGAGAATCCCGGTGCCGAGGCCGCTCAATTGGTCGTAGACAGCT**x**TAGCACCGCTTAAACGCACGTACGCG C TGTCCCCCGCGTTTTAACCGCCAAGG**x**GATTACTCCCTAGTCTCCAGGCACGTGTCAGATATATACATCCTG

AP oligos

Top Left adapter (5′ to 3′)
/5Cy3/ CGGTACCCGG GGATCCTCTA GAGTGGGAGC TCGGAACACT ATCCGACTGG CACCGGCAAG GTCGCTGTTC AATACATGCA CAGGATGTATAT

Top Right adapter (5′ to 3′)
/5Phos/ CAATTGAGCG GCCTCGGCACCGGGATTCTCCA

Top Left helper (5′ to 3′)
CGTGTCAGAT ATATACATCC

Top Right helper (5′ to 3′)
CGCTCAATTG GTCGTAGACA

Top Core AP on SHL+/-2 (5′ to 3′)
/5Phos/ ATCTGACACG TGCCTGGAGA CTAGGGAGTA ATCCCCTTGG **/idSp/** GGTTAAAAC GCGGGGGACA G CGCGTACGTG CGTTTAAGC **/idSp/** GTGCTAGAGC TGTCTACGAC

Top Core AP on SHL+/-2.7 (5′ to 3′)
/5Phos/ ATCTGACACG TGCCTGGAGA CTAGGGAGTA ATC **/idSp/** CCTTGG CGGTTAAAAC GCGGGGGACA G CGCGTACGTG CGTTTAAGCG GTGCTA **/idSp/** AGC TGTCTACGAC

Bottom Left adapter (5′ to 3′)
/5Phos/ ATATACATCCTG TGCATGTATT GAACAGCGAC CTTGCCGGTG CCAGTCGGAT AGTGTTCCGA GCTCCCACTC TAGAGGATCC CCGGGTACCG

Bottom Right adapter (5′ to 3′)
/5Cy5/ TGGAGAATCCCGGTGCCGAGGC CGCTCAATTG

Bottom Left helper (5′ to 3′)
TGTCTACGAC CAATTGAGCG

Bottom Right helper (5′ to 3′)
GGATGTATAT ATCTGACACG

Bottom Core AP on SHL+/-2 (5′ to 3′)
/5Phos/ GTCGTAGACA GCTCTAGCAC **/idSp/** GCTTAAACG CACGTACGCG C TGTCCCCCGC GTTTTAACC **/idSp/** CCAAGGGGAT TACTCCCTAG TCTCCAGGCA CGTGTCAGAT

Bottom Core AP on SHL+/-2.7 (5′ to 3′)
/5Phos/ GTCGTAGACA GCT **/idSp/** TAGCAC CGCTTAAACG CACGTACGCG C TGTCCCCCGC GTTTTAACCG CCAAGG **/idSp/** GAT TACTCCCTAG TCTCCAGGCA CGTGTCAGAT
