# Supplementary figures and images for: Bi-directional nucleosome sliding by the Chd1 chromatin remodeler integrates intrinsic sequence-dependent and ATP-dependent nucleosome positioning
Source: Nucleic Acids Res. 2023 Sep 20;51(19):10326–43. doi: 10.1093/nar/gkad738 (PMC10602870; doi:10.1093/nar/gkad738)

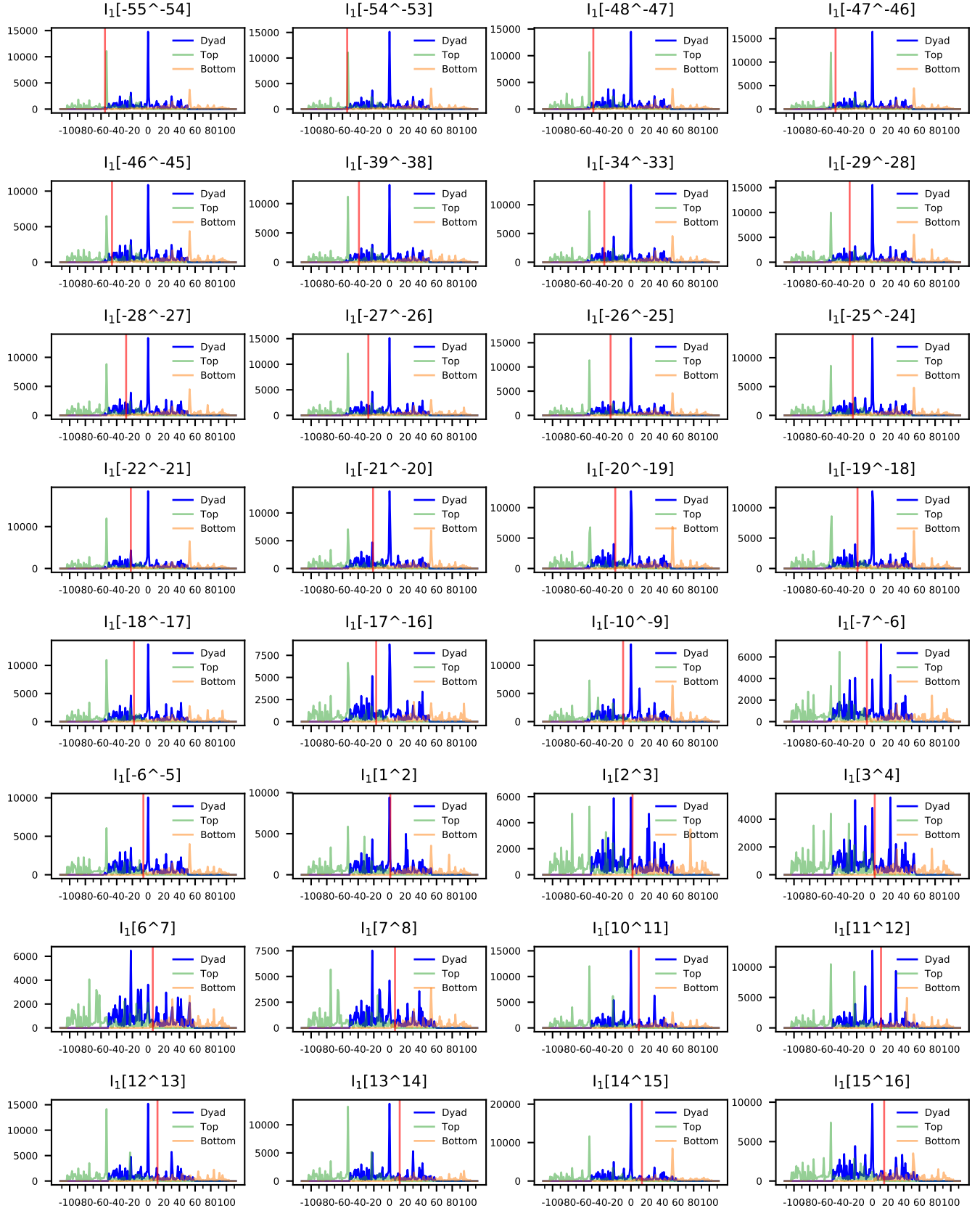

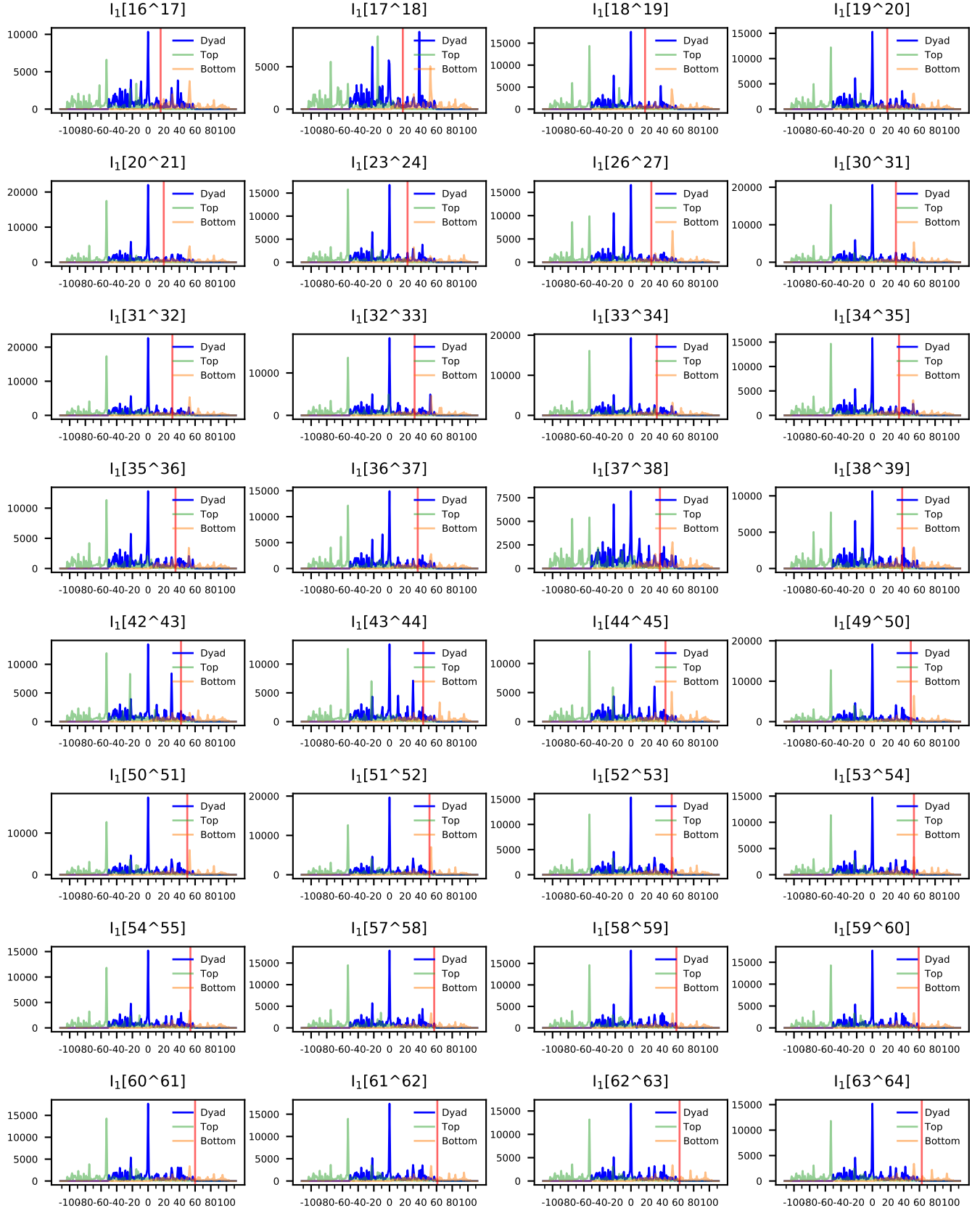

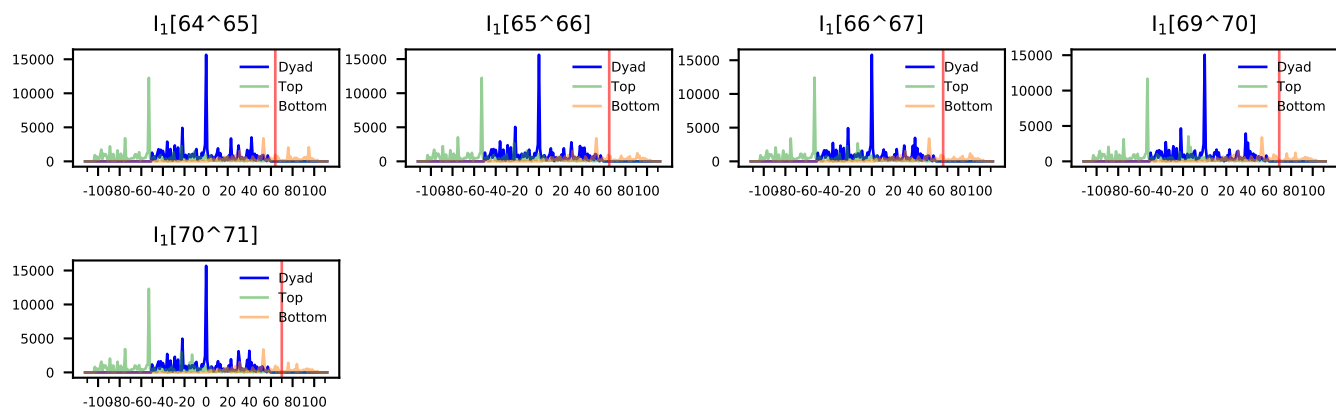

Supplement: gkad738_Supplemental_files [file gkad738_supplemental_files.zip › Supplementary Table 10 (SWH1-Signals_I_before).pdf]

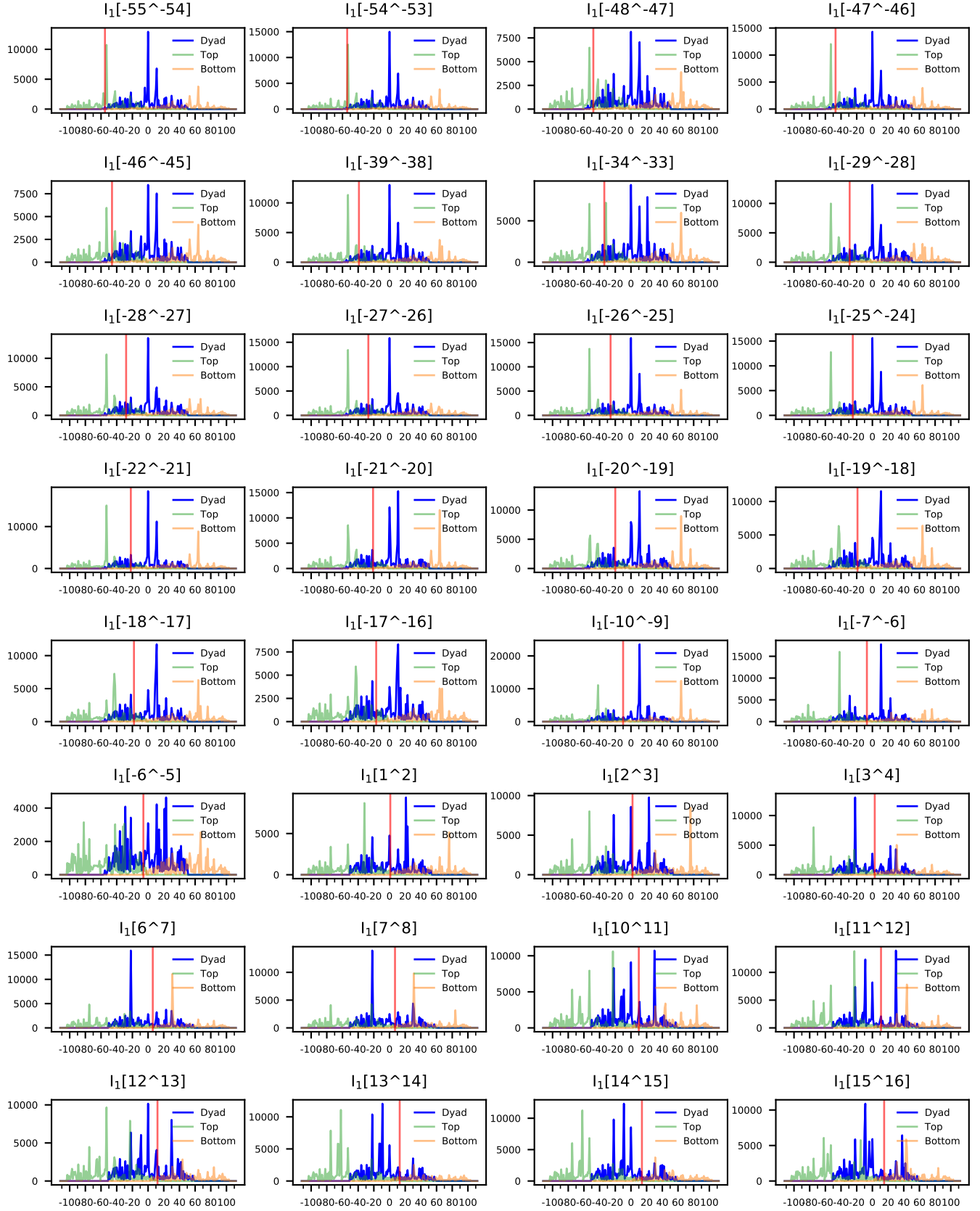

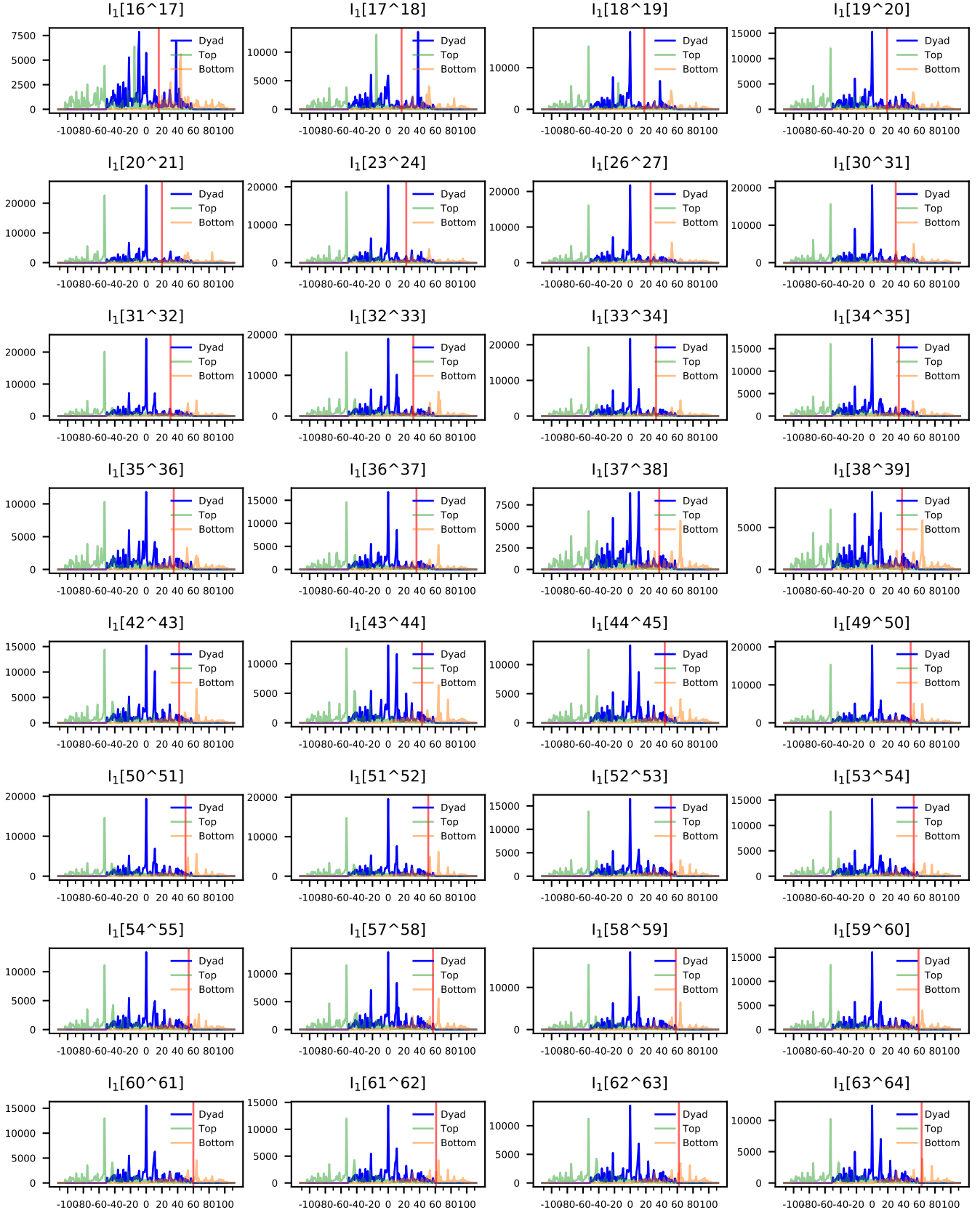

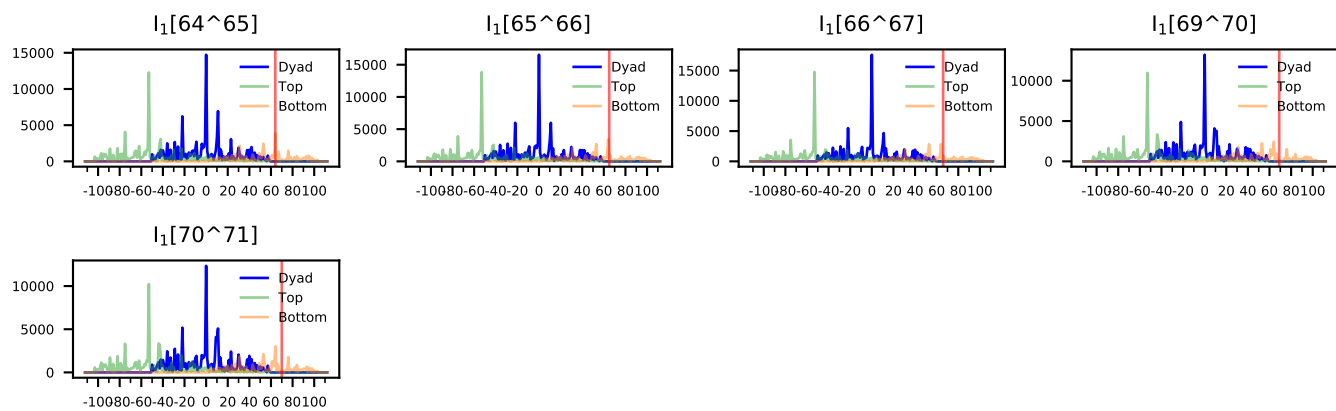

Supplement: gkad738_Supplemental_files [file gkad738_supplemental_files.zip › Supplementary Table 11 (SWH1-Signals_I_after).pdf]

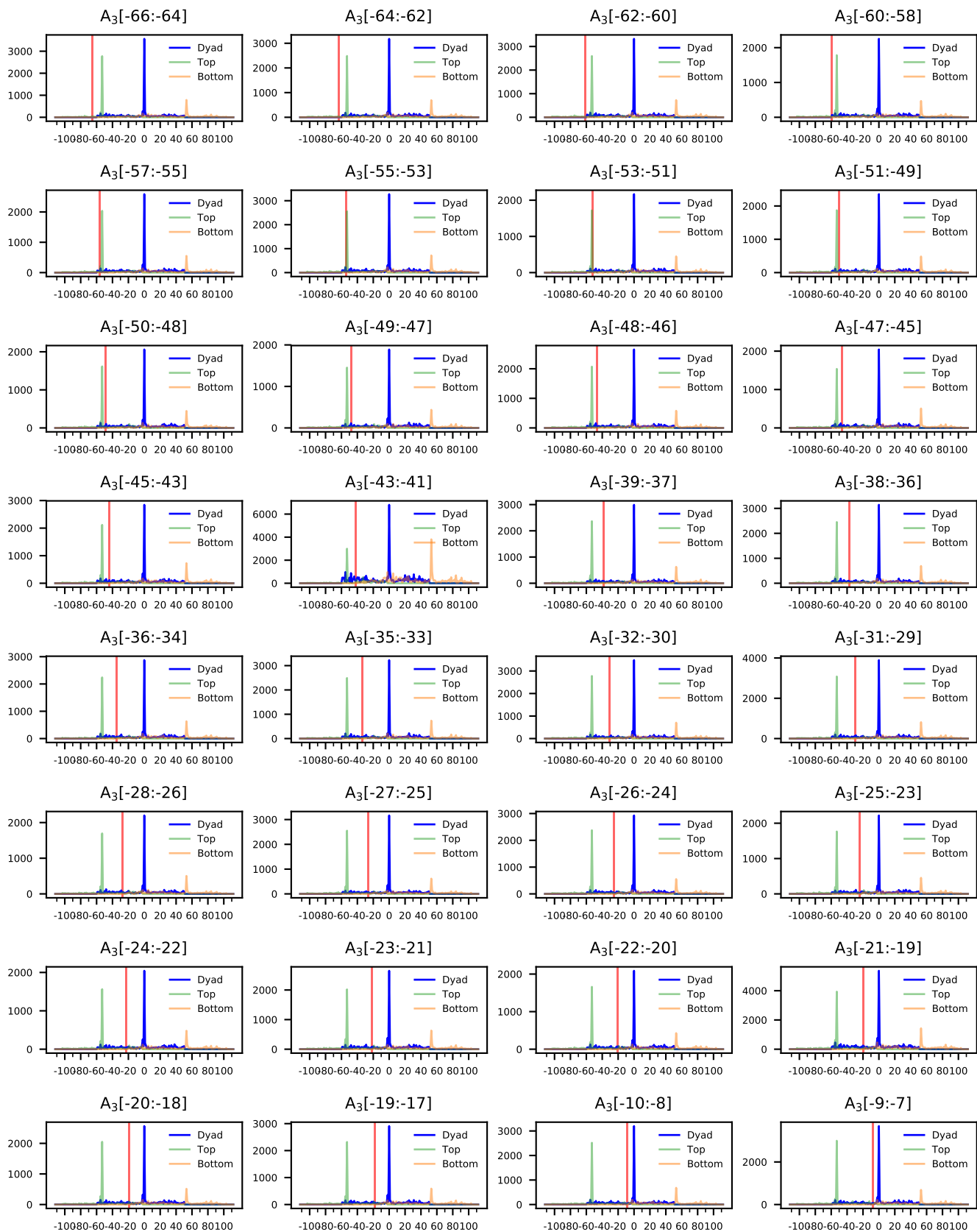

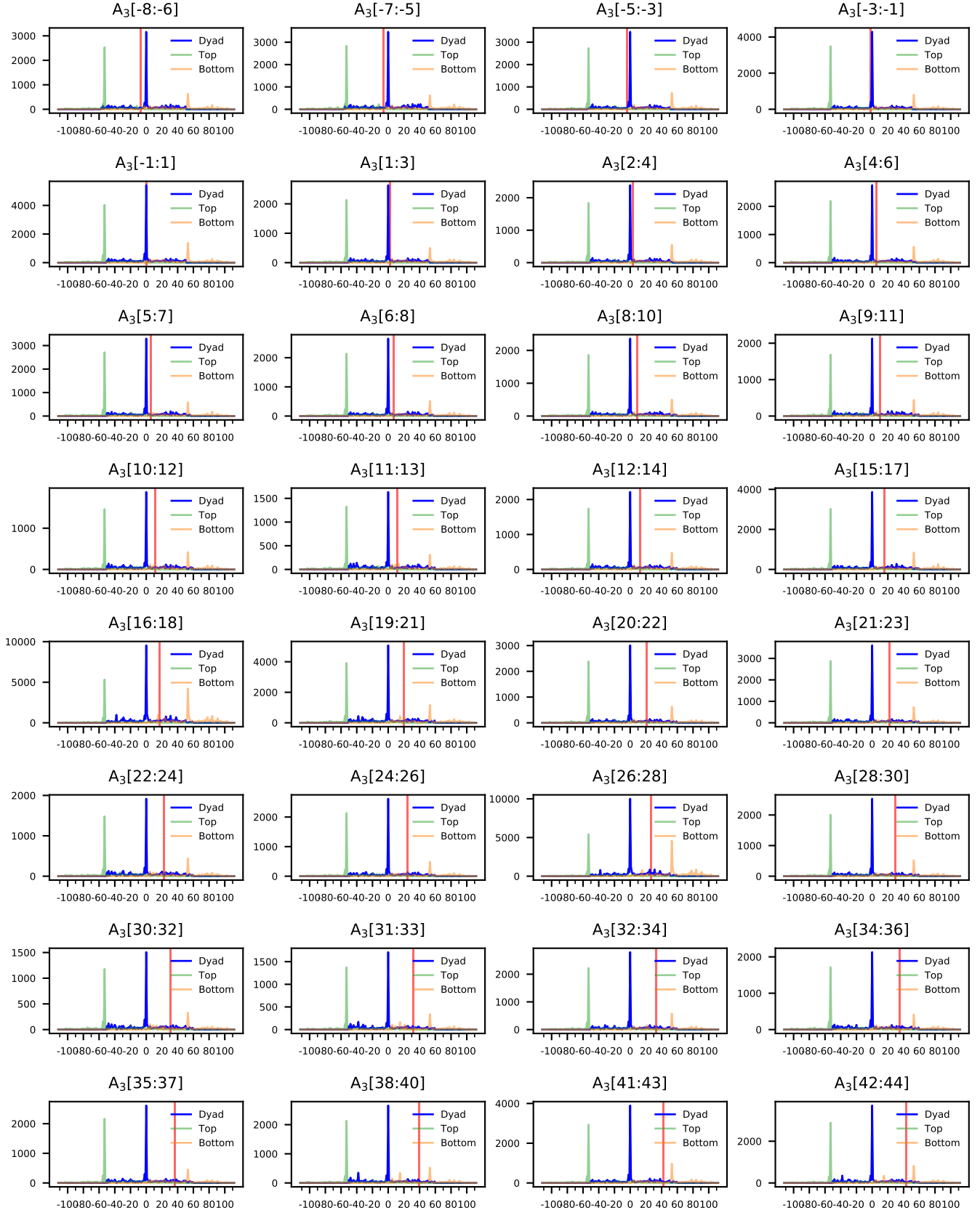

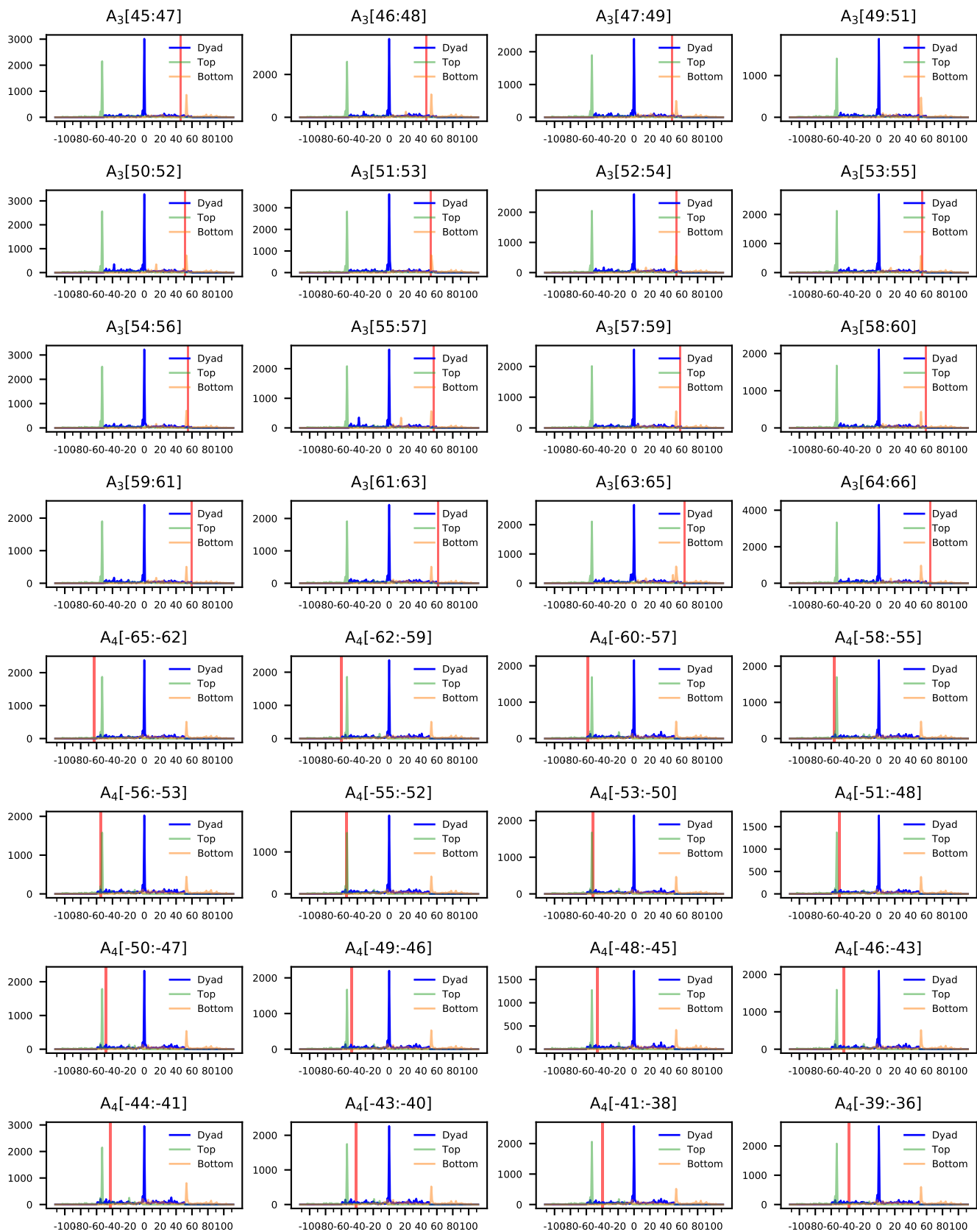

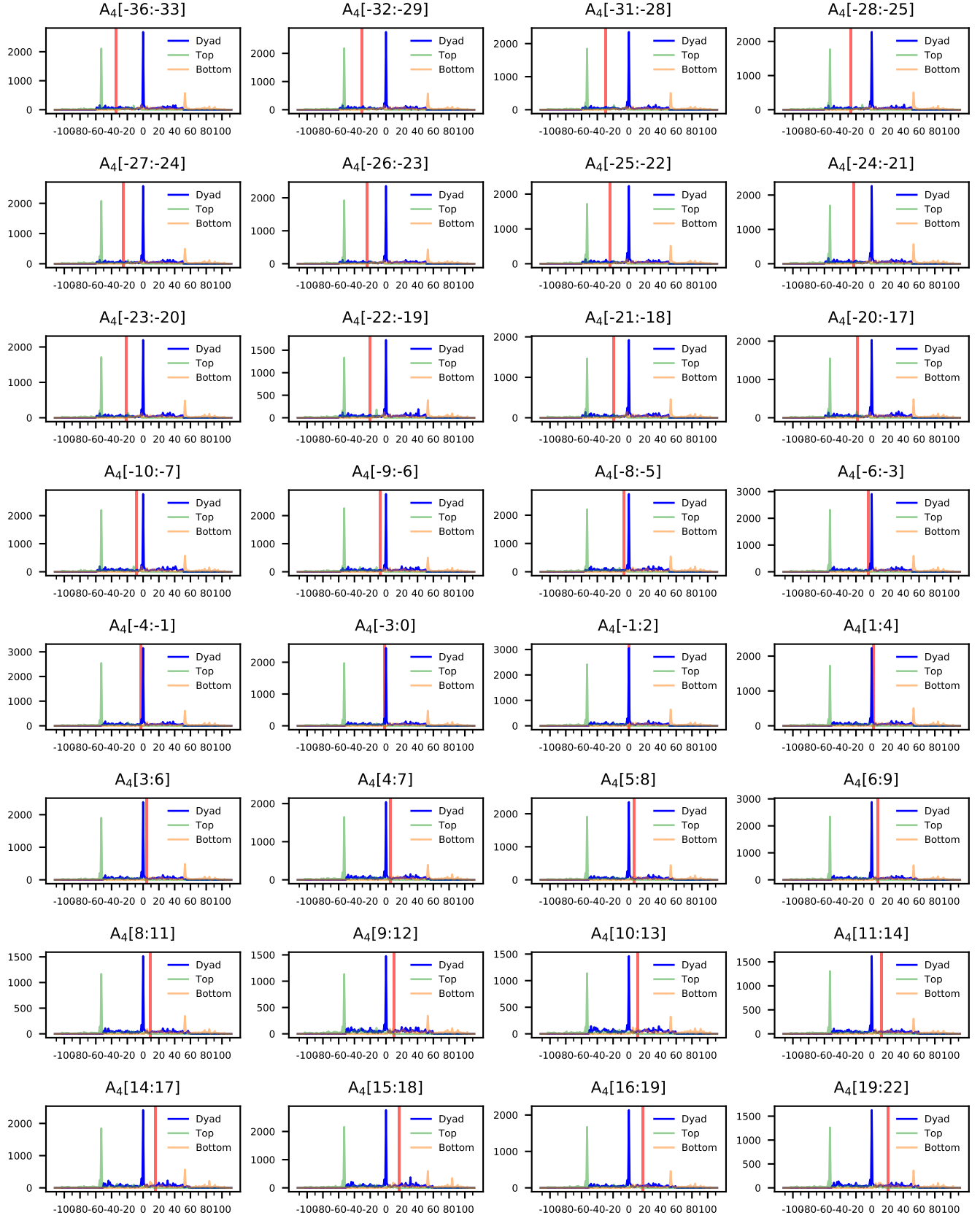

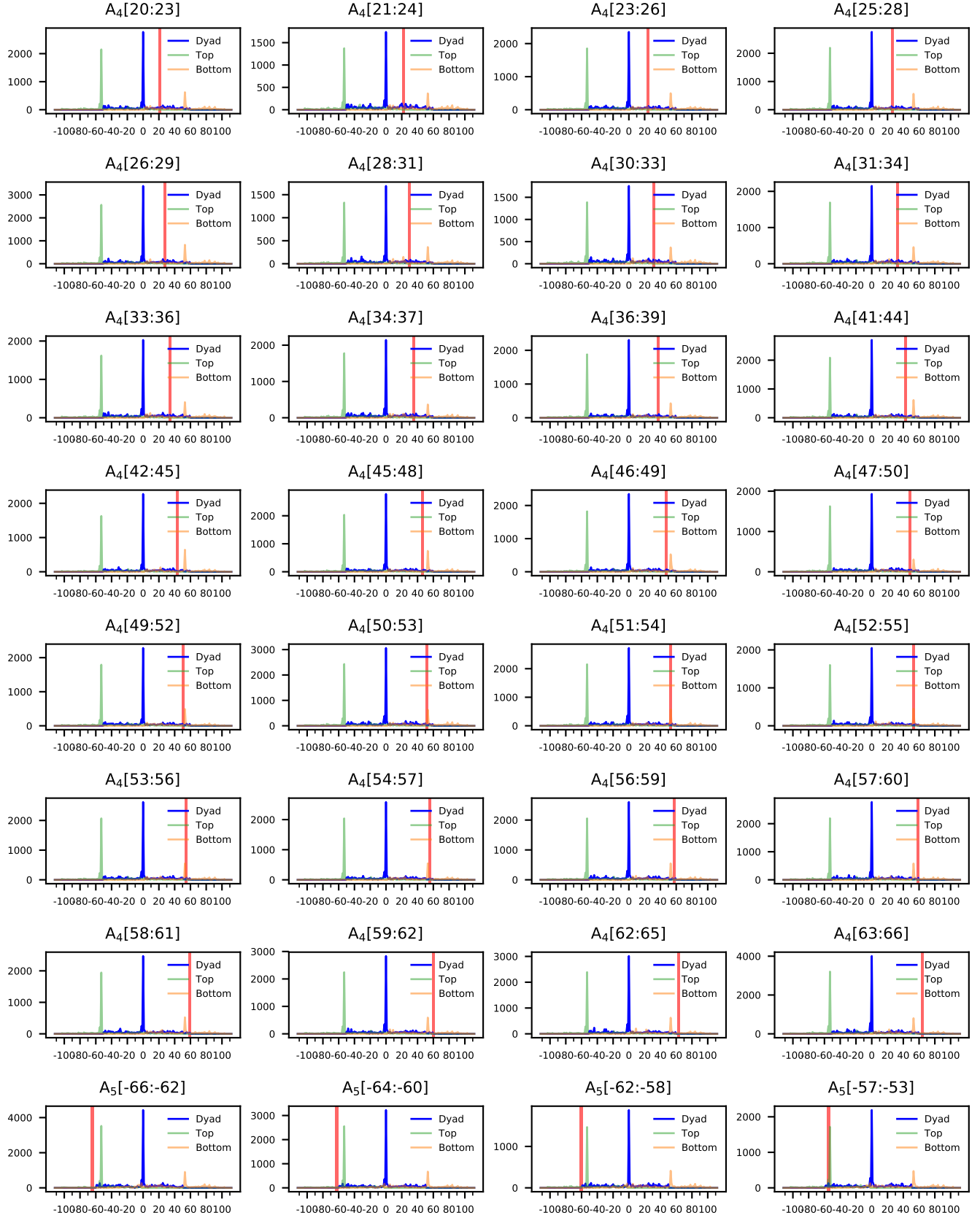

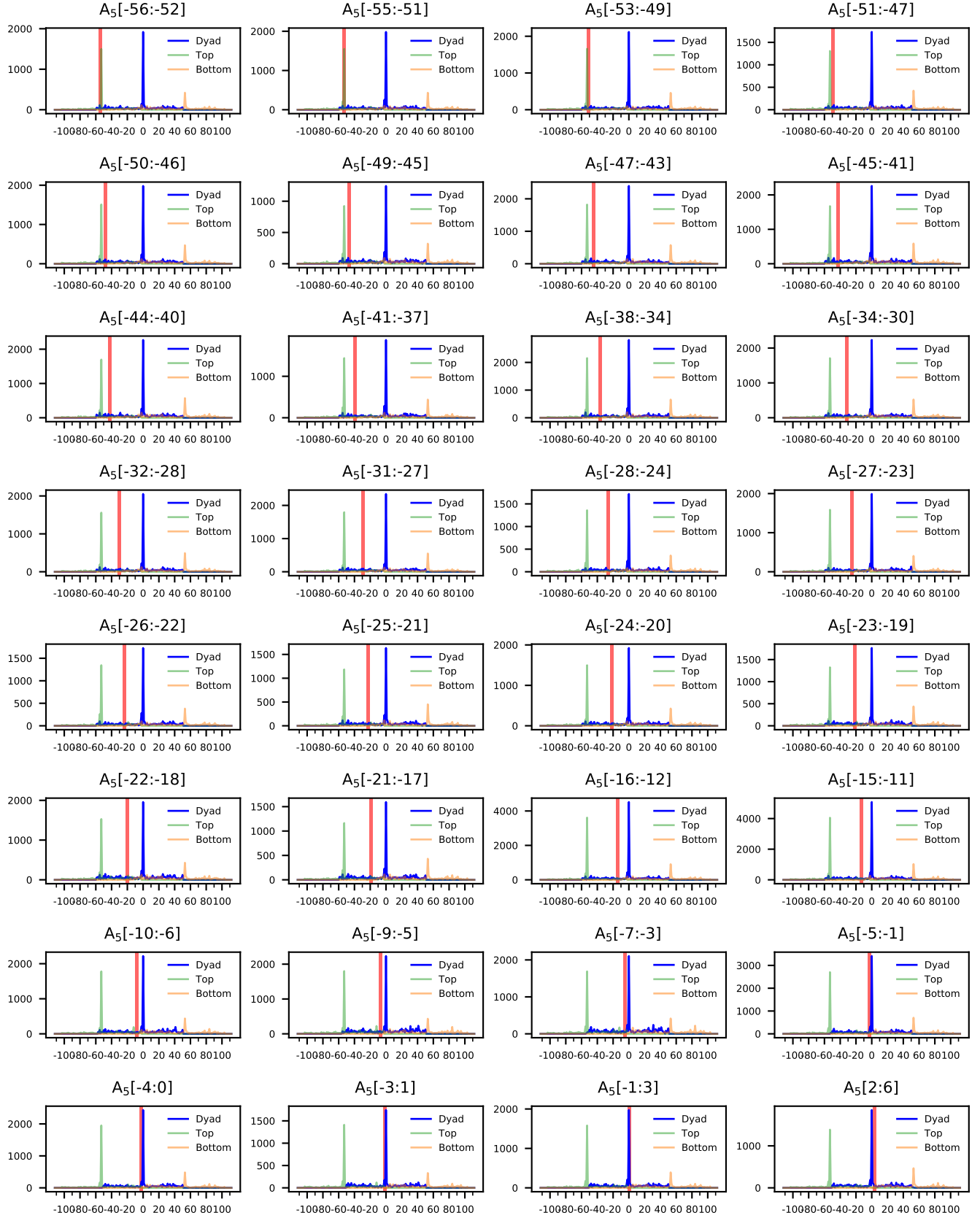

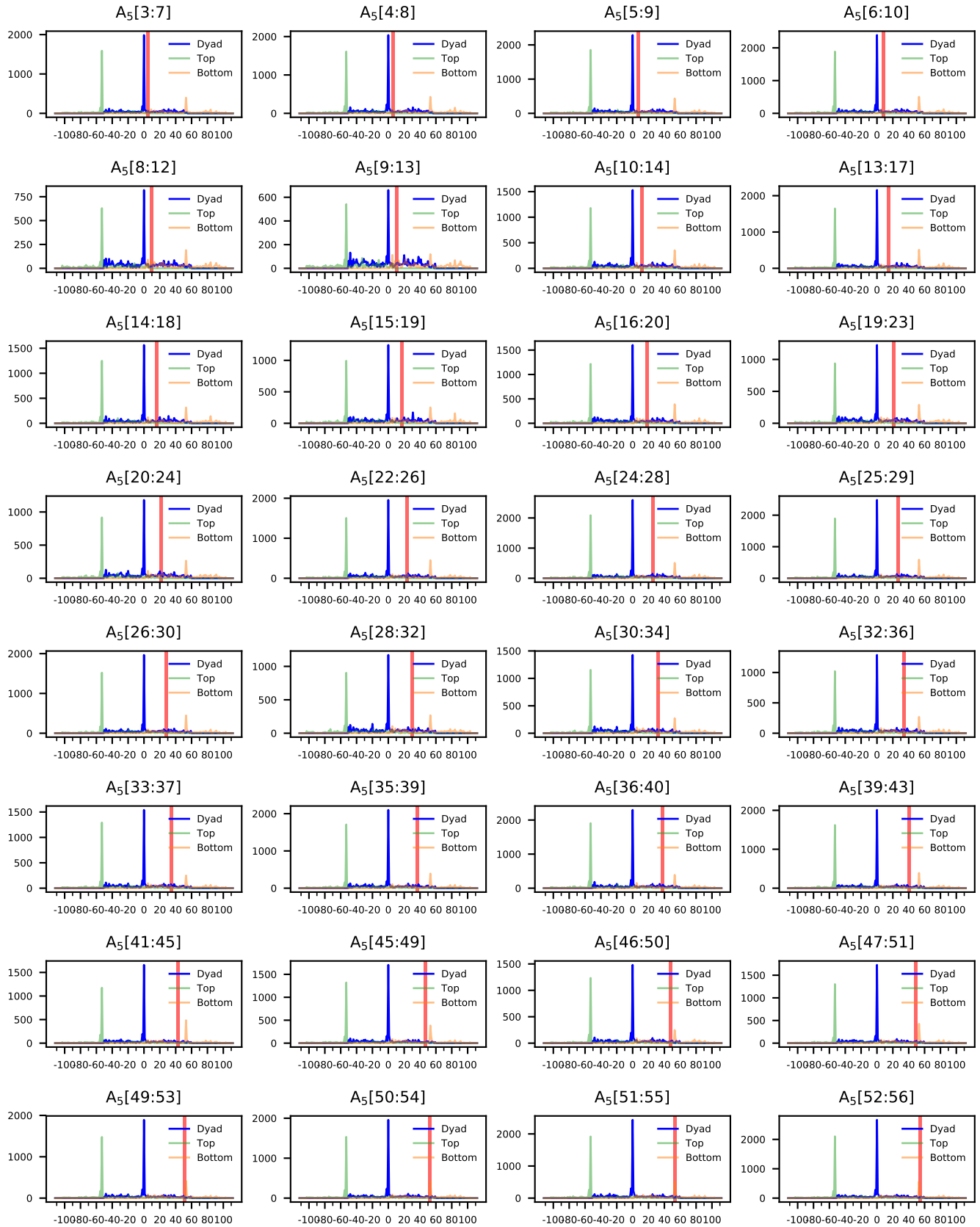

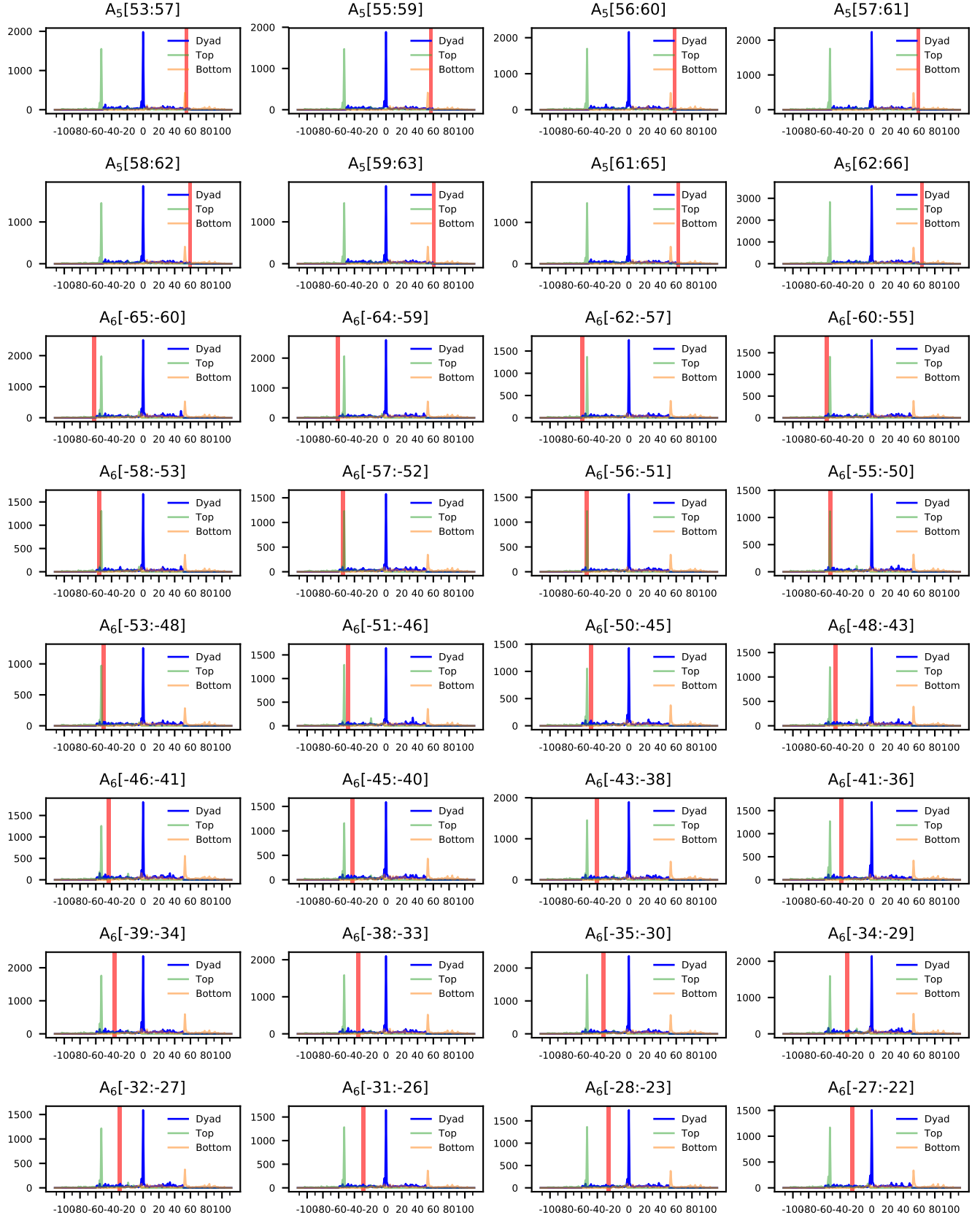

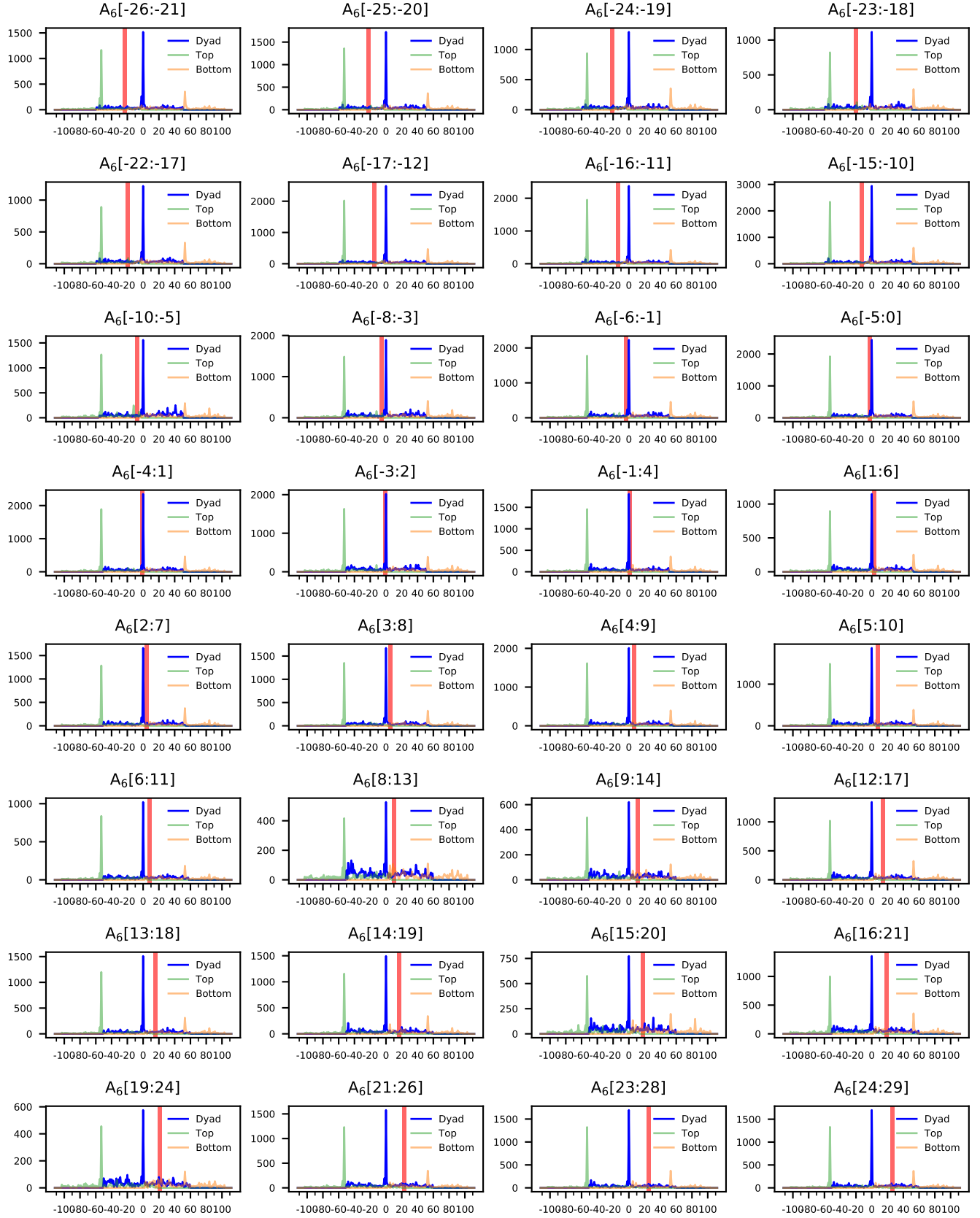

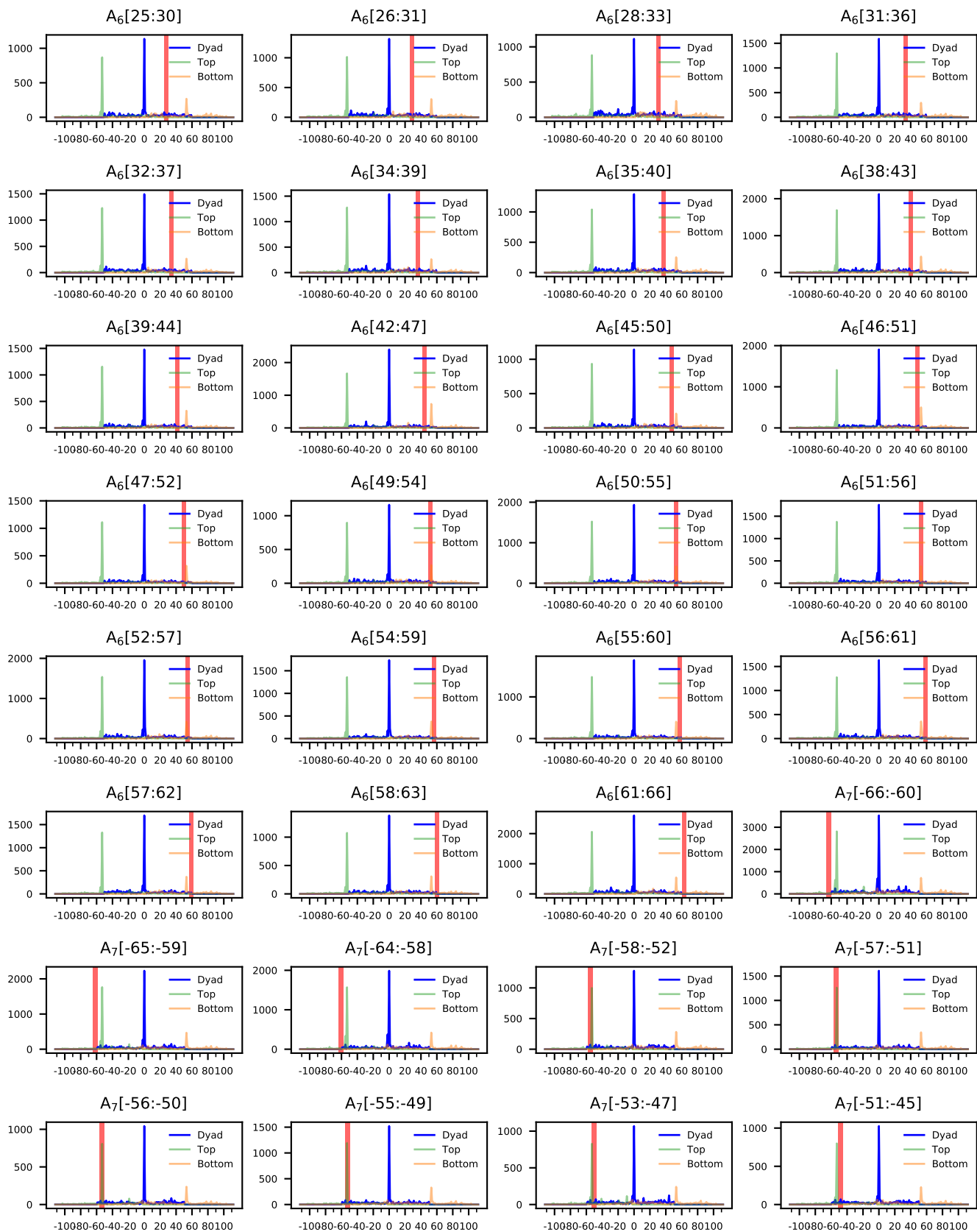

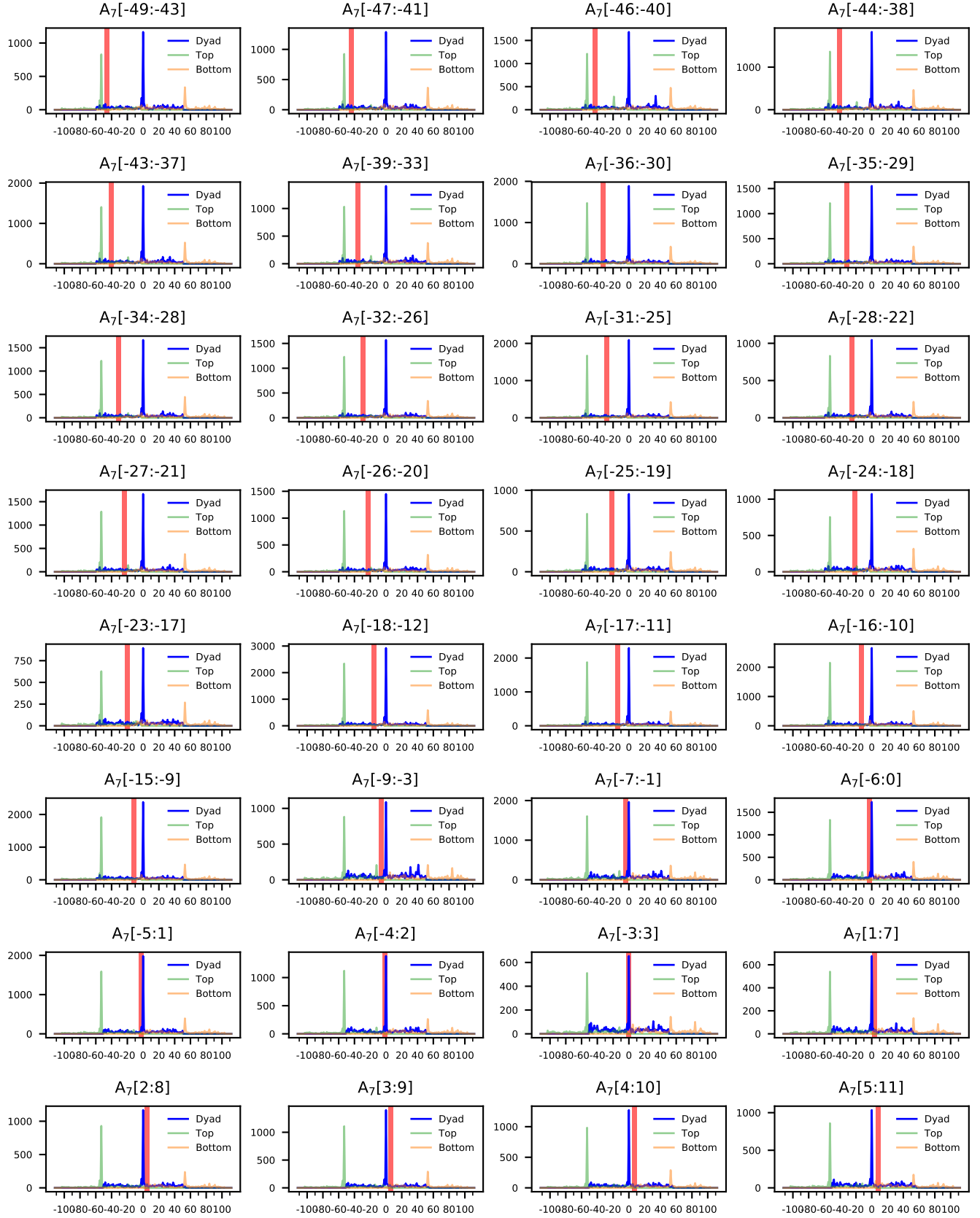

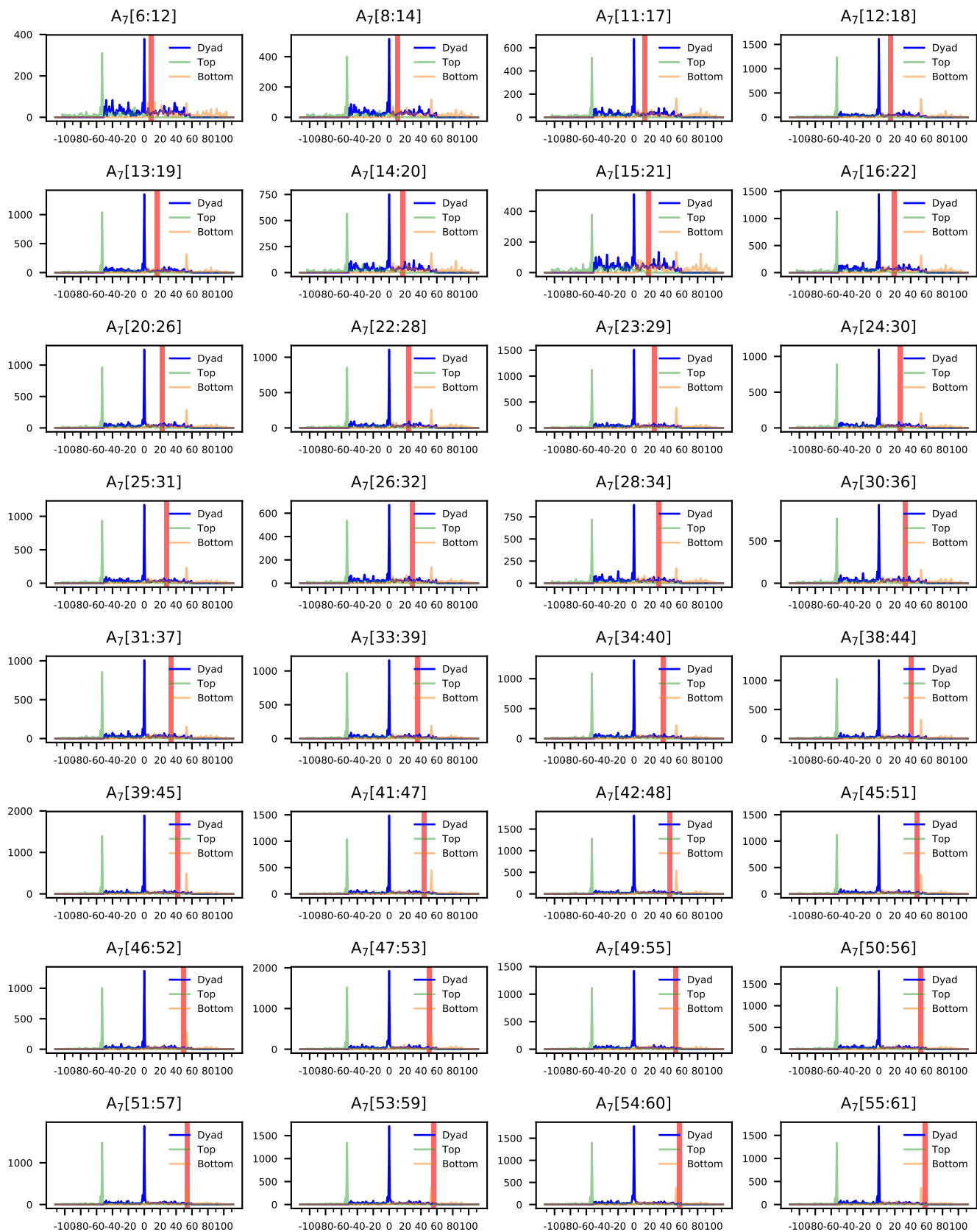

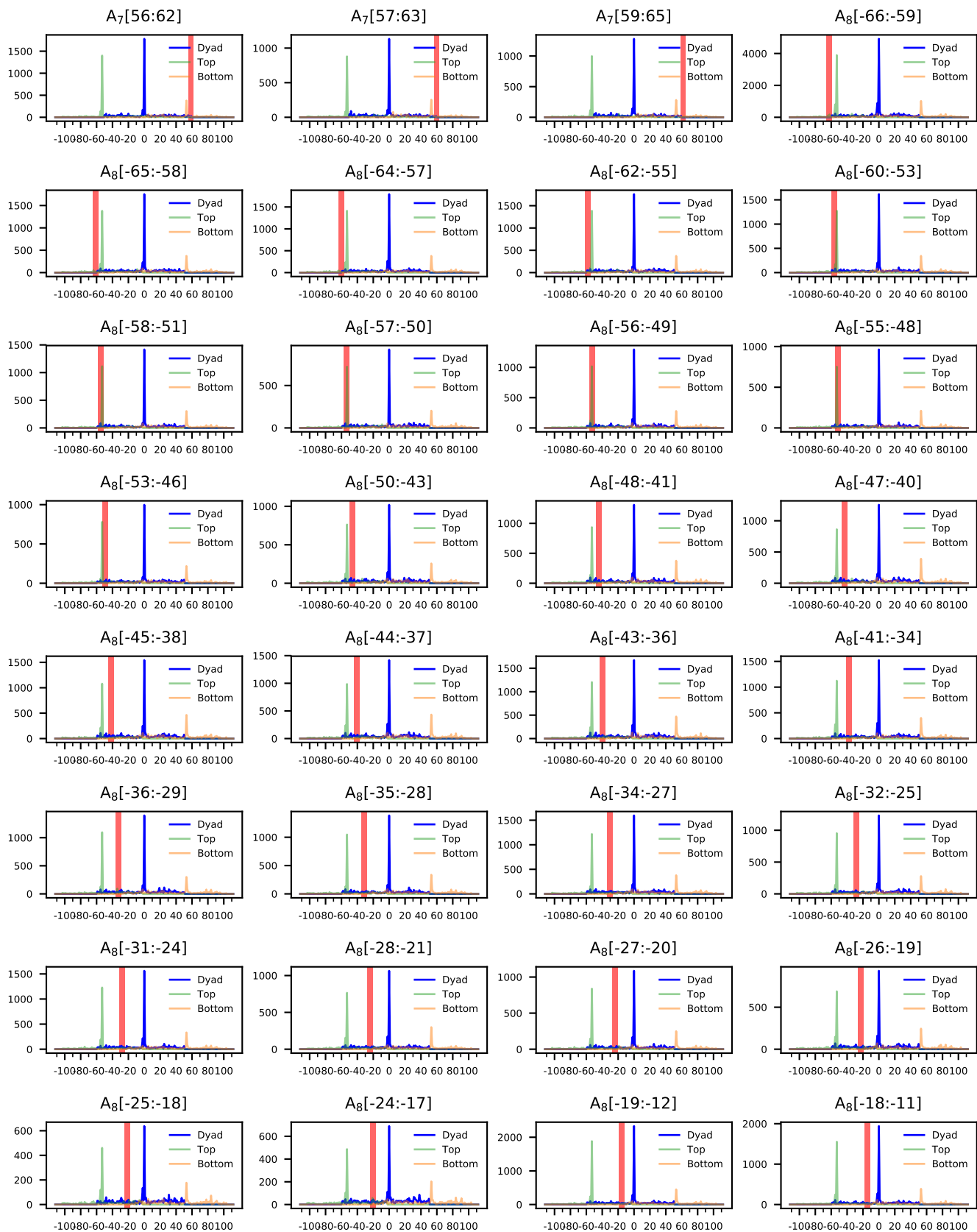

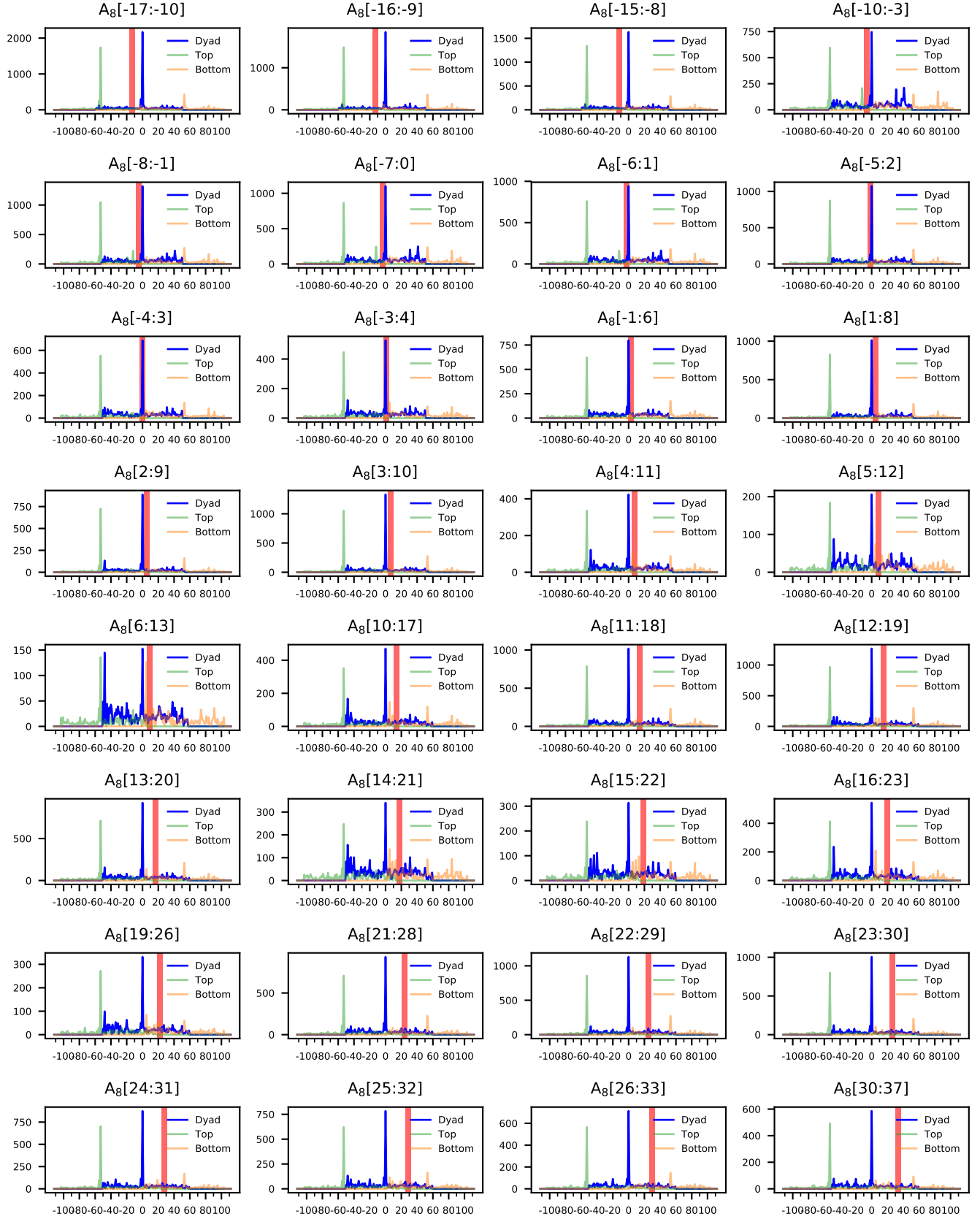

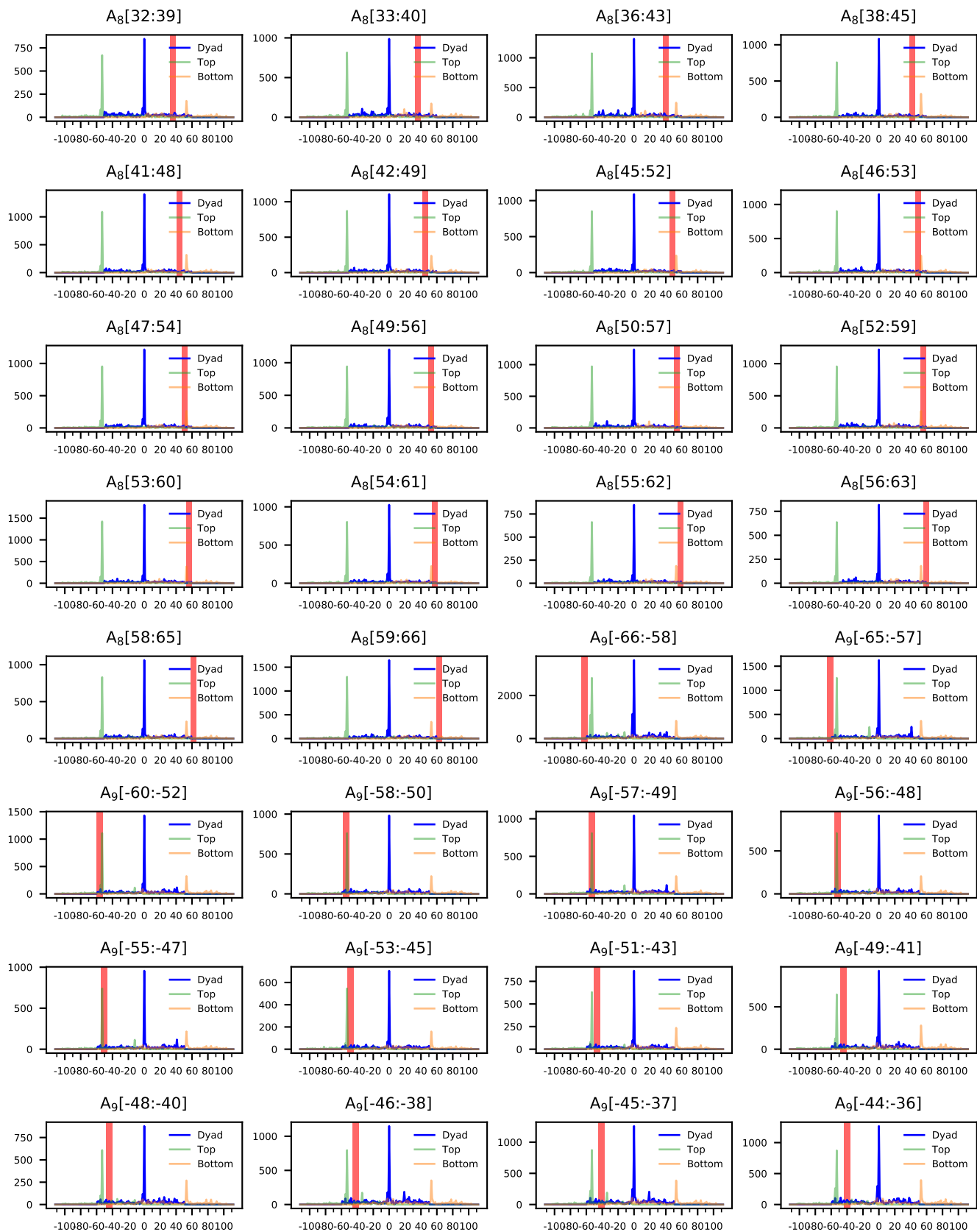

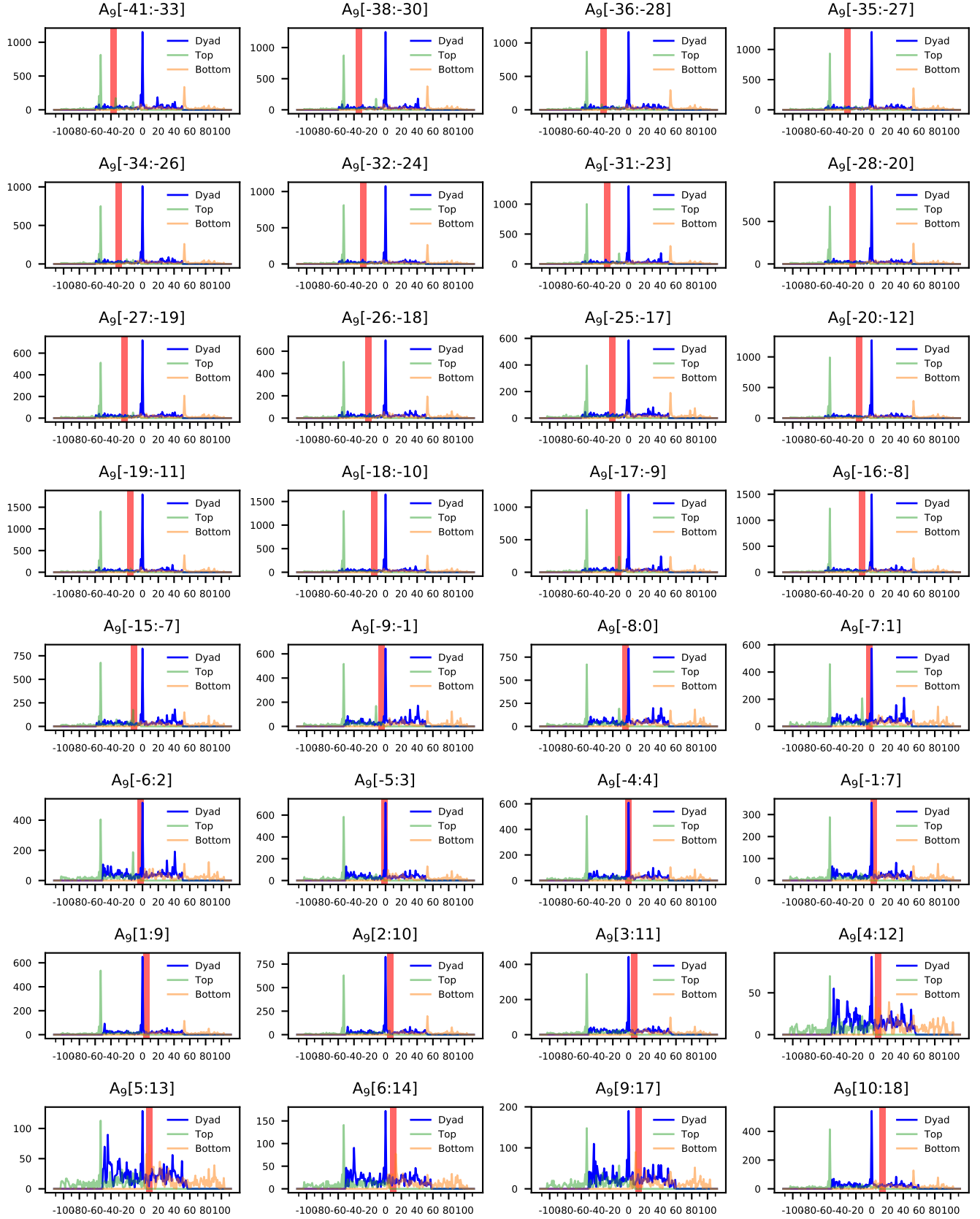

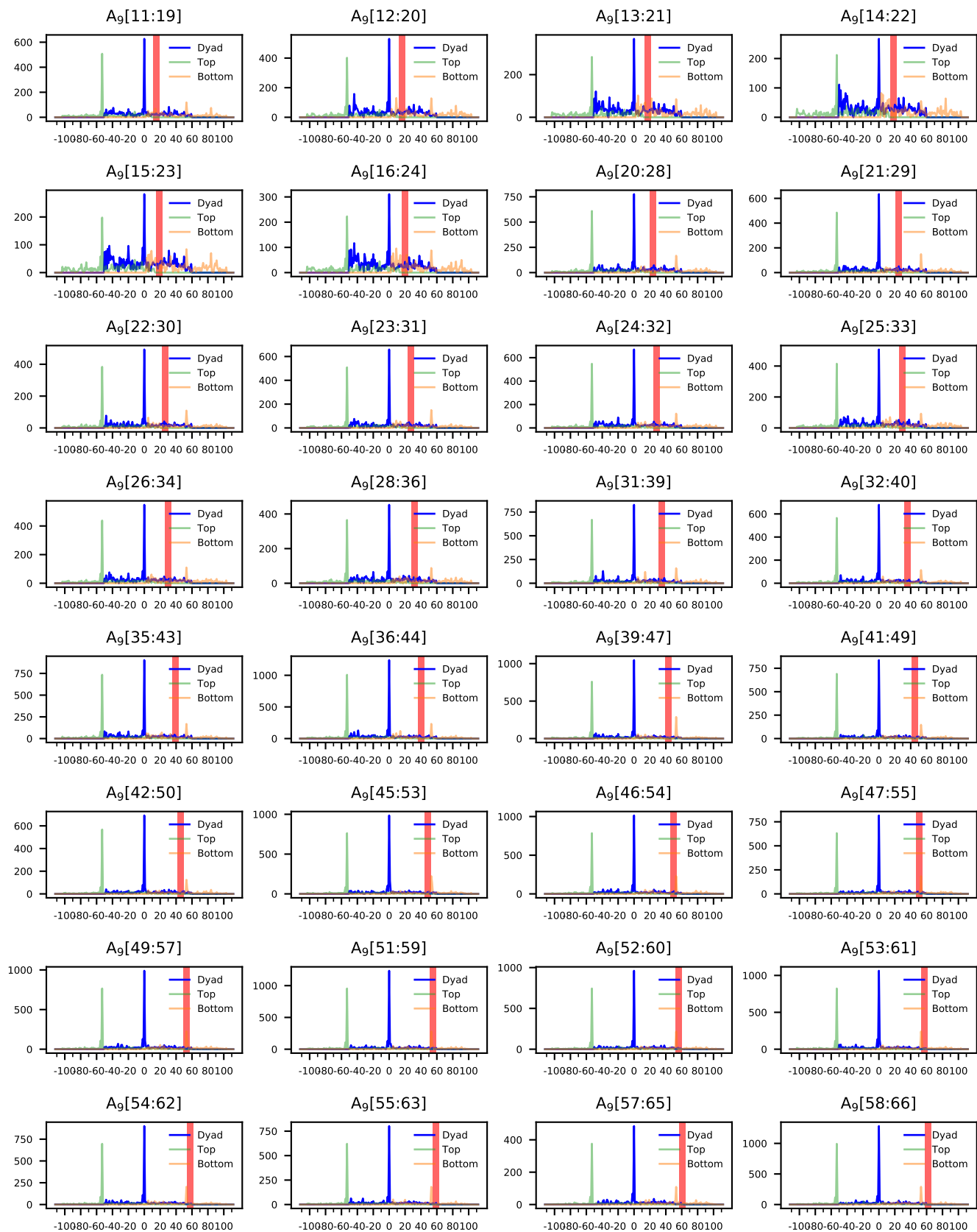

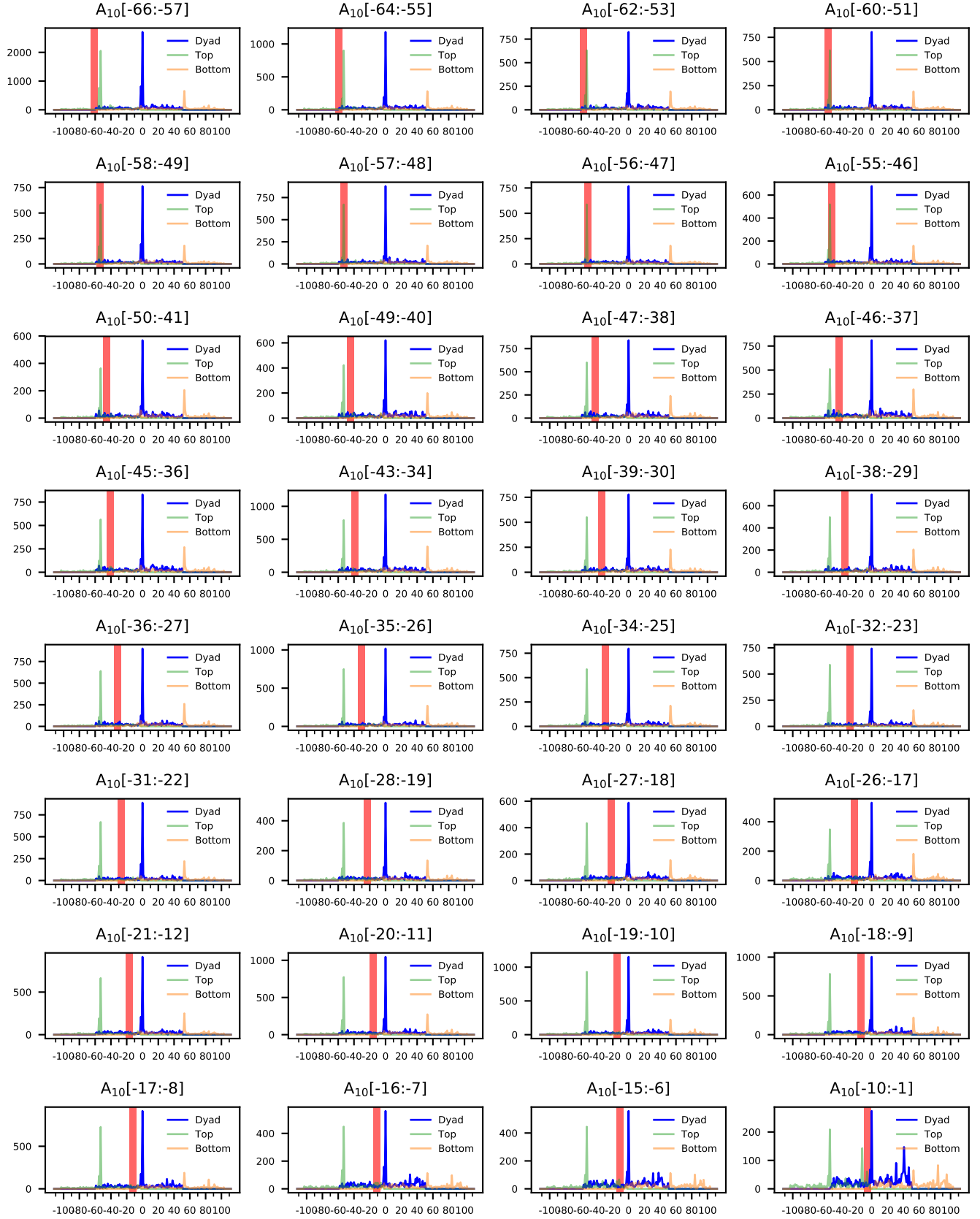

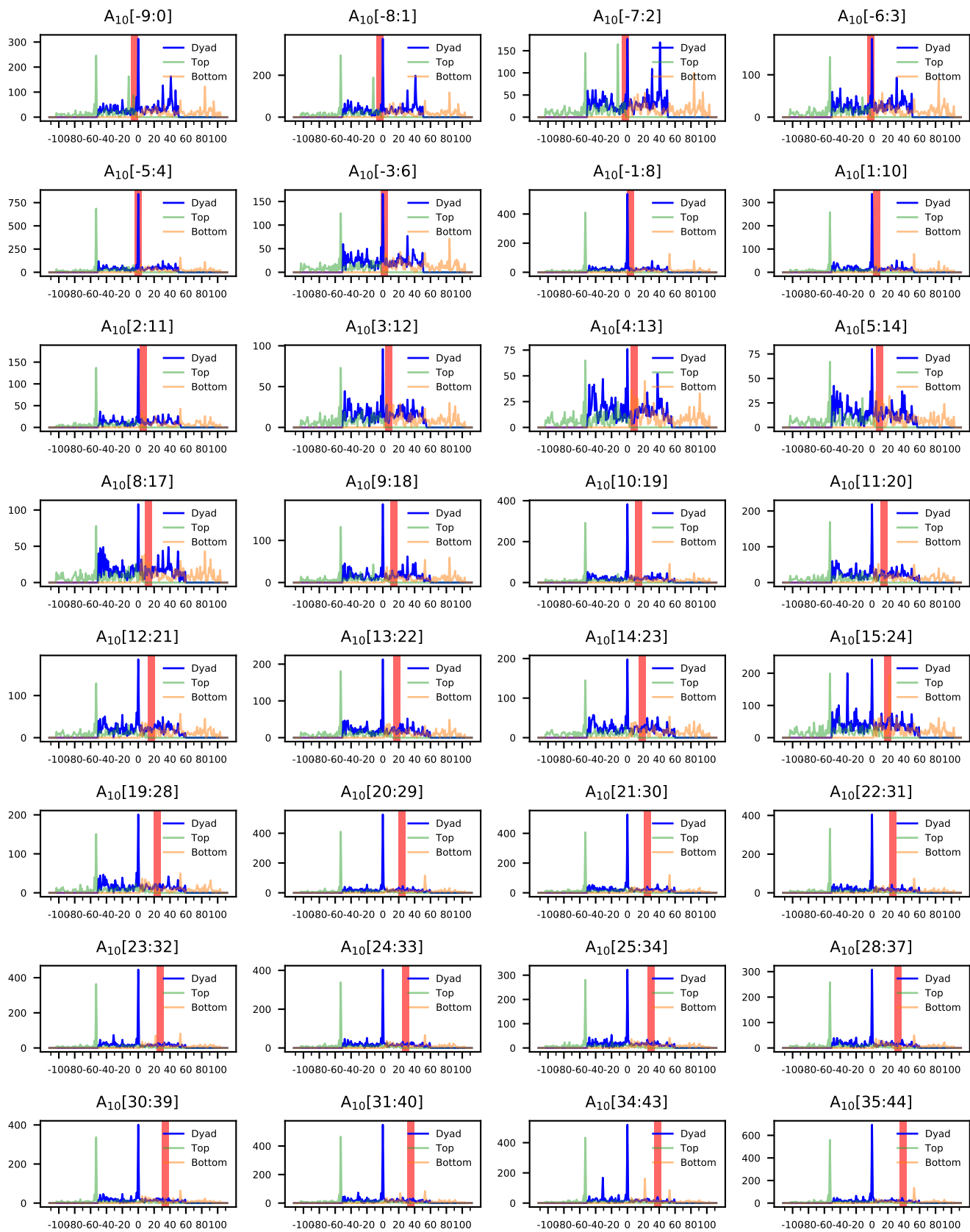

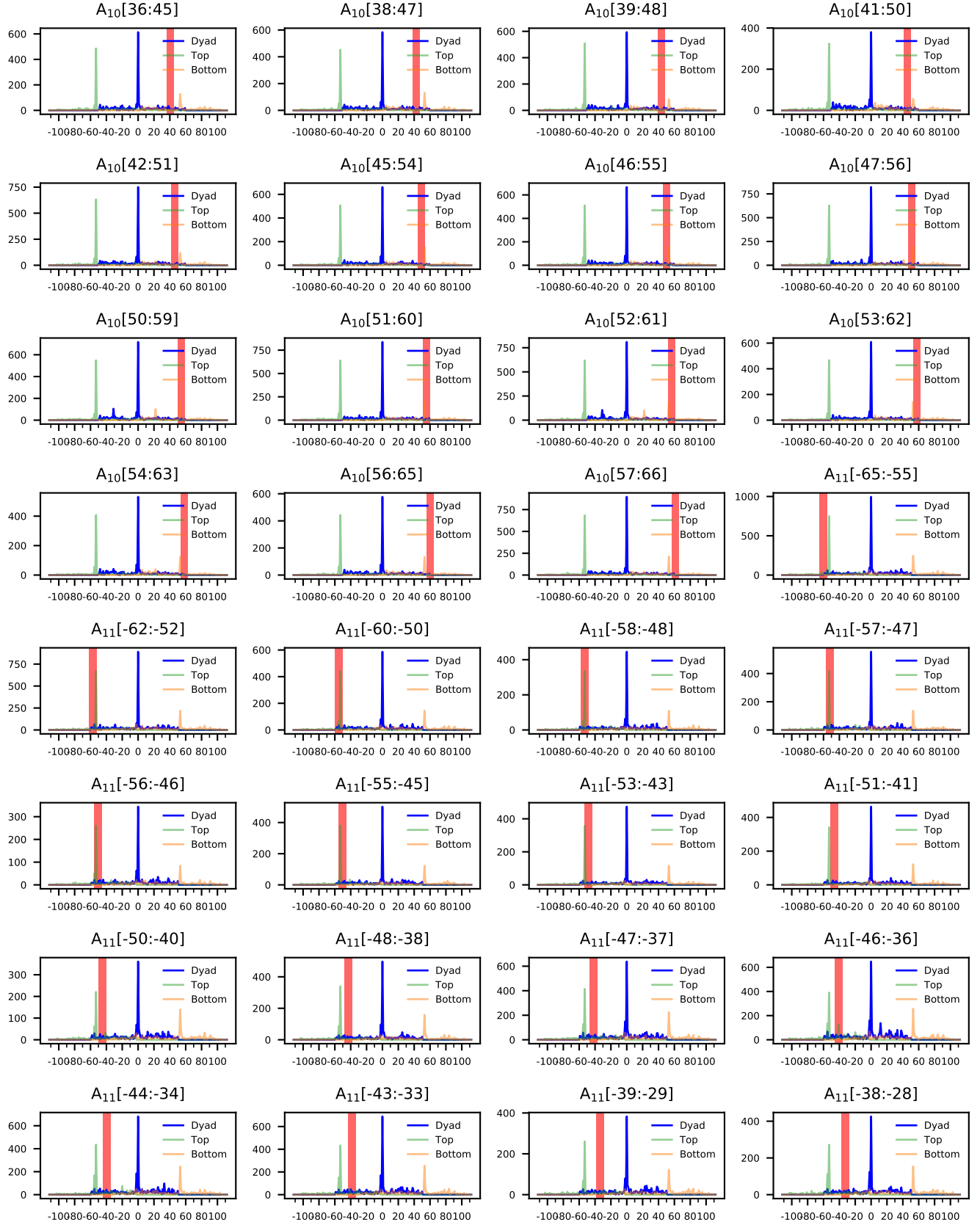

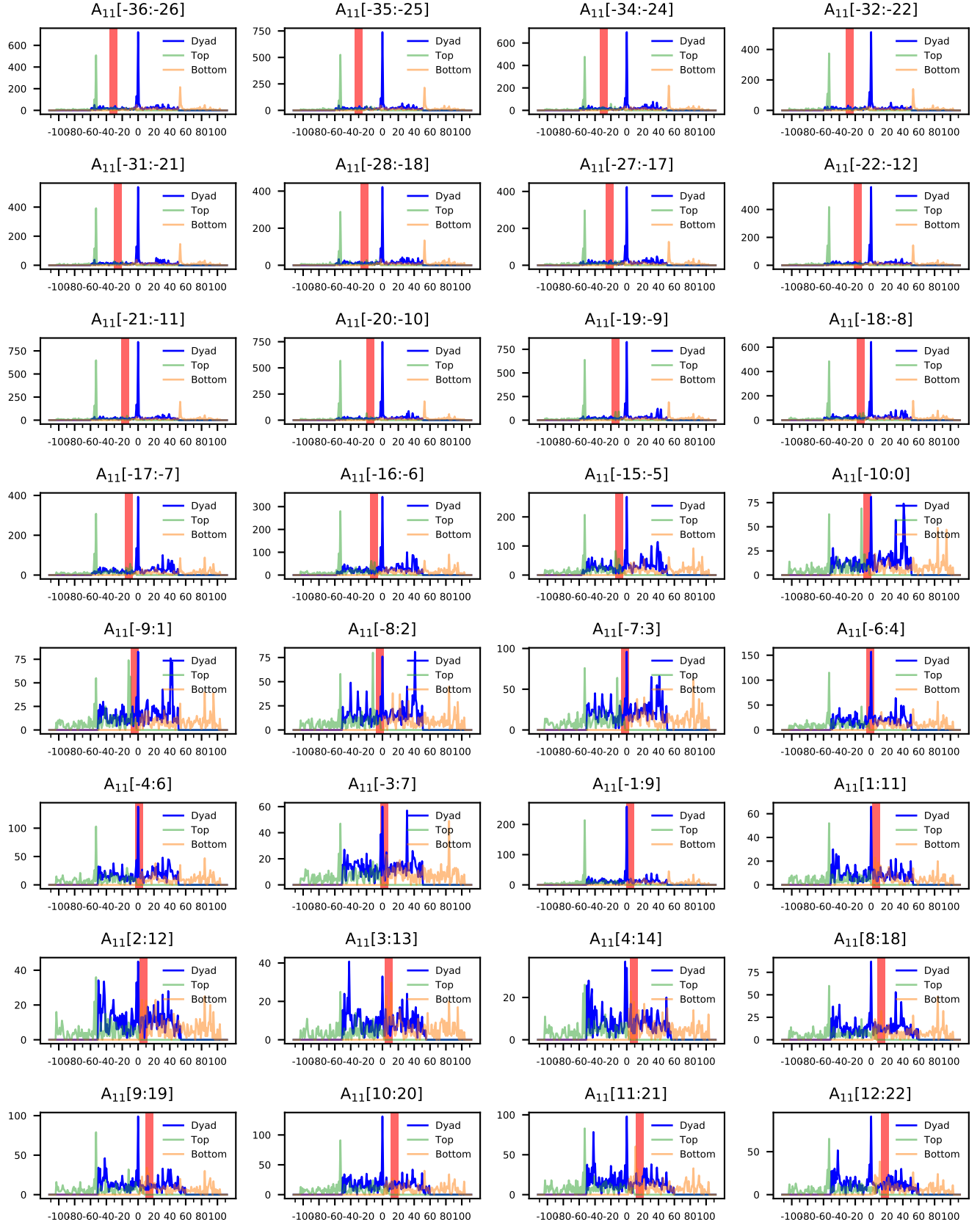

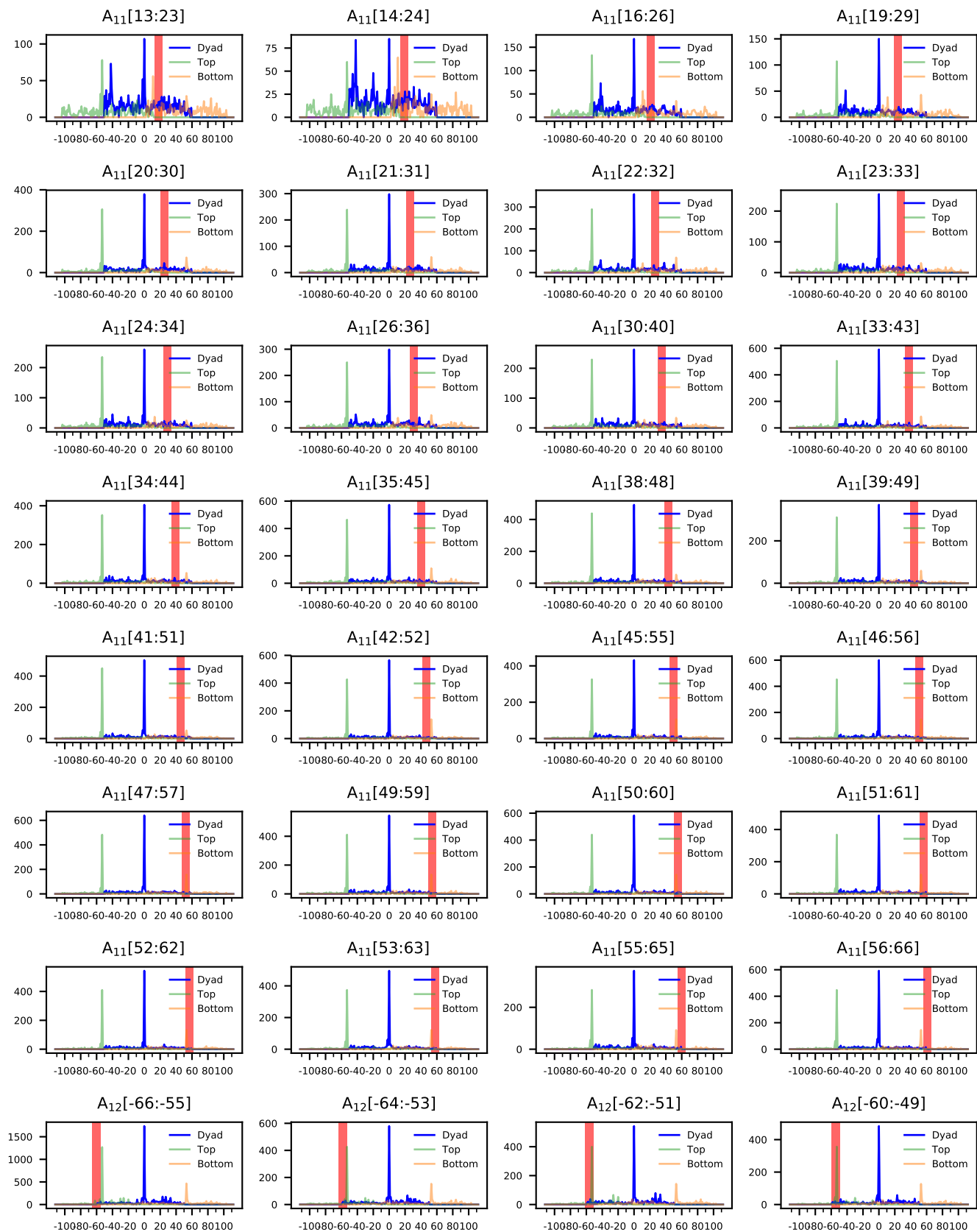

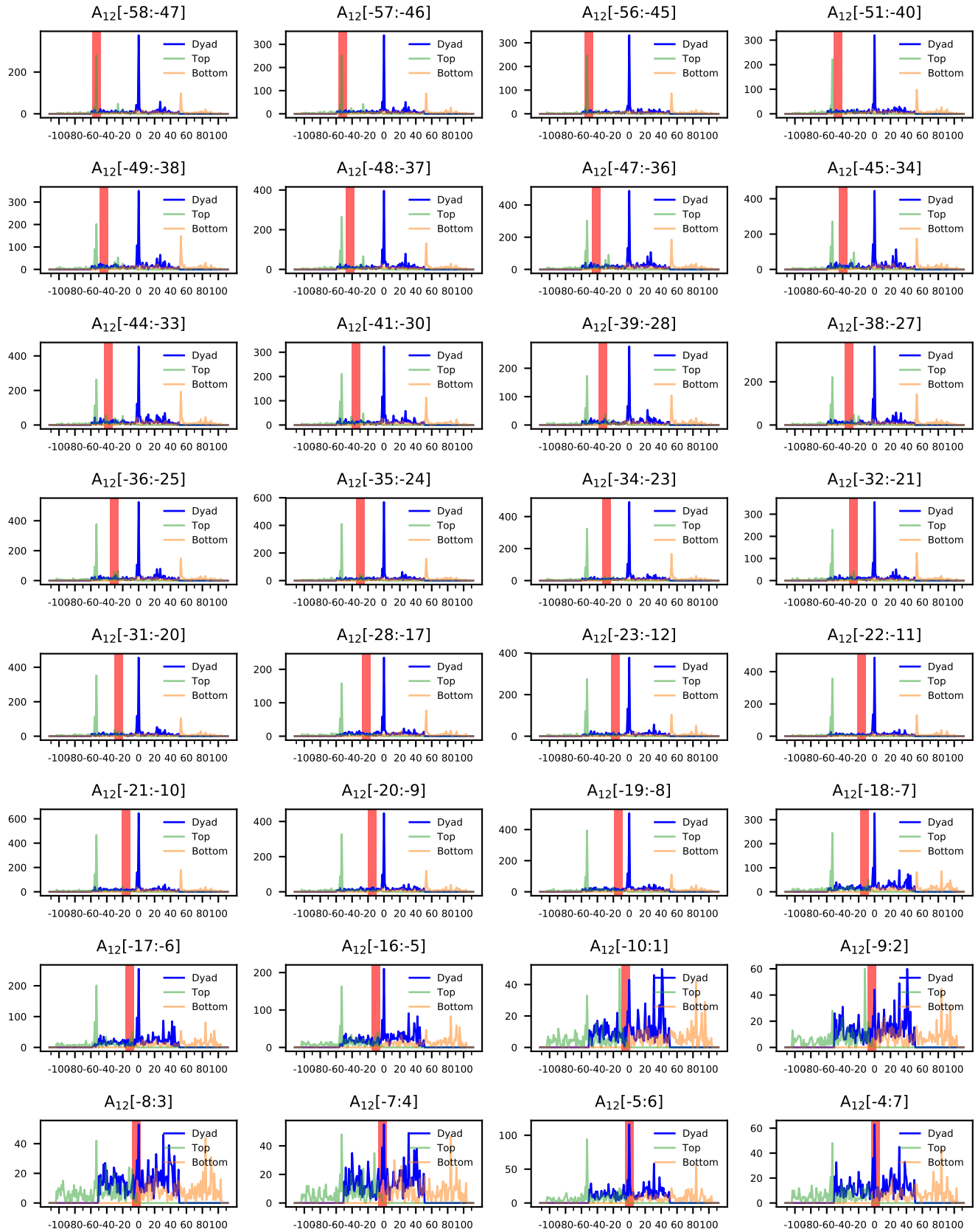

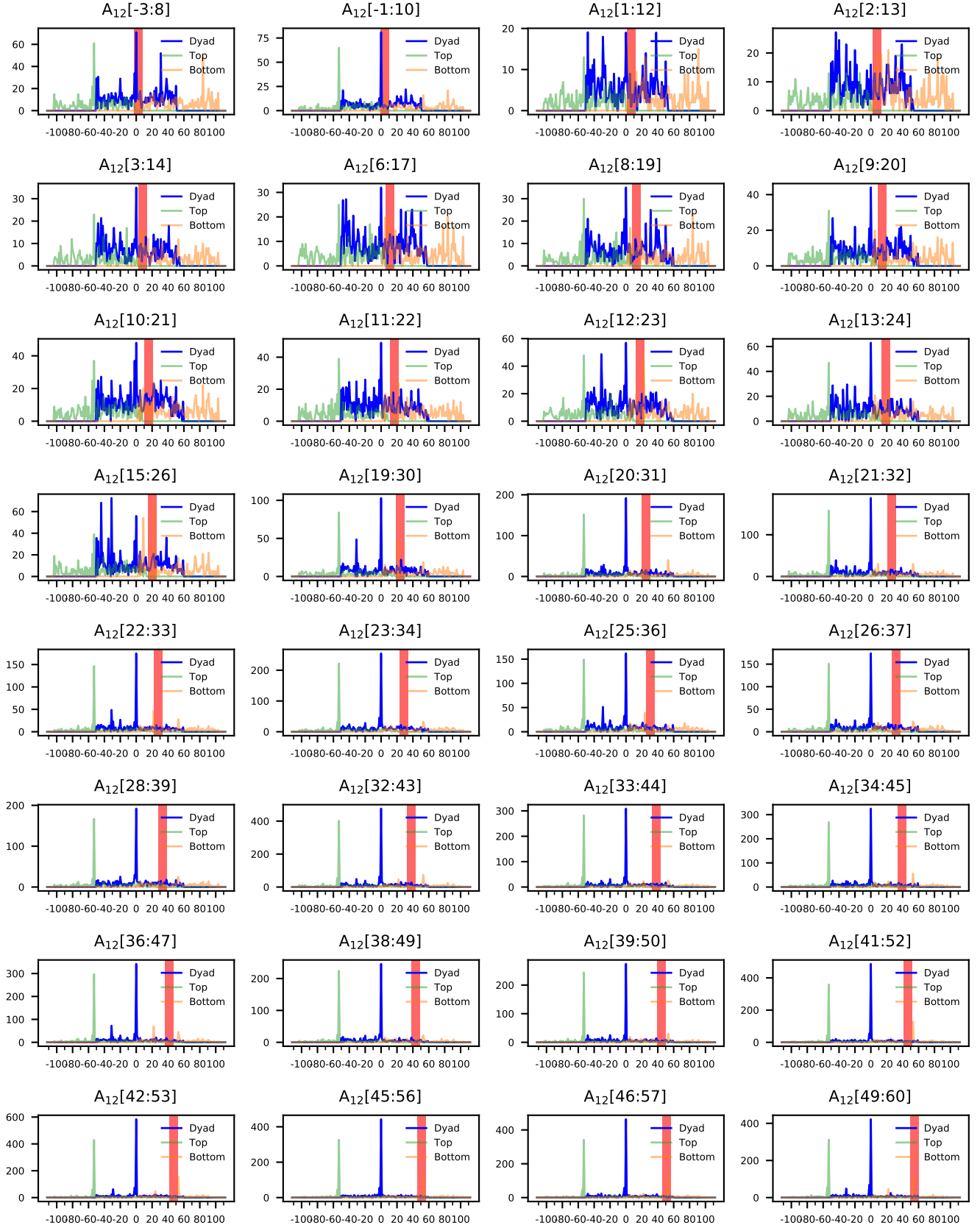

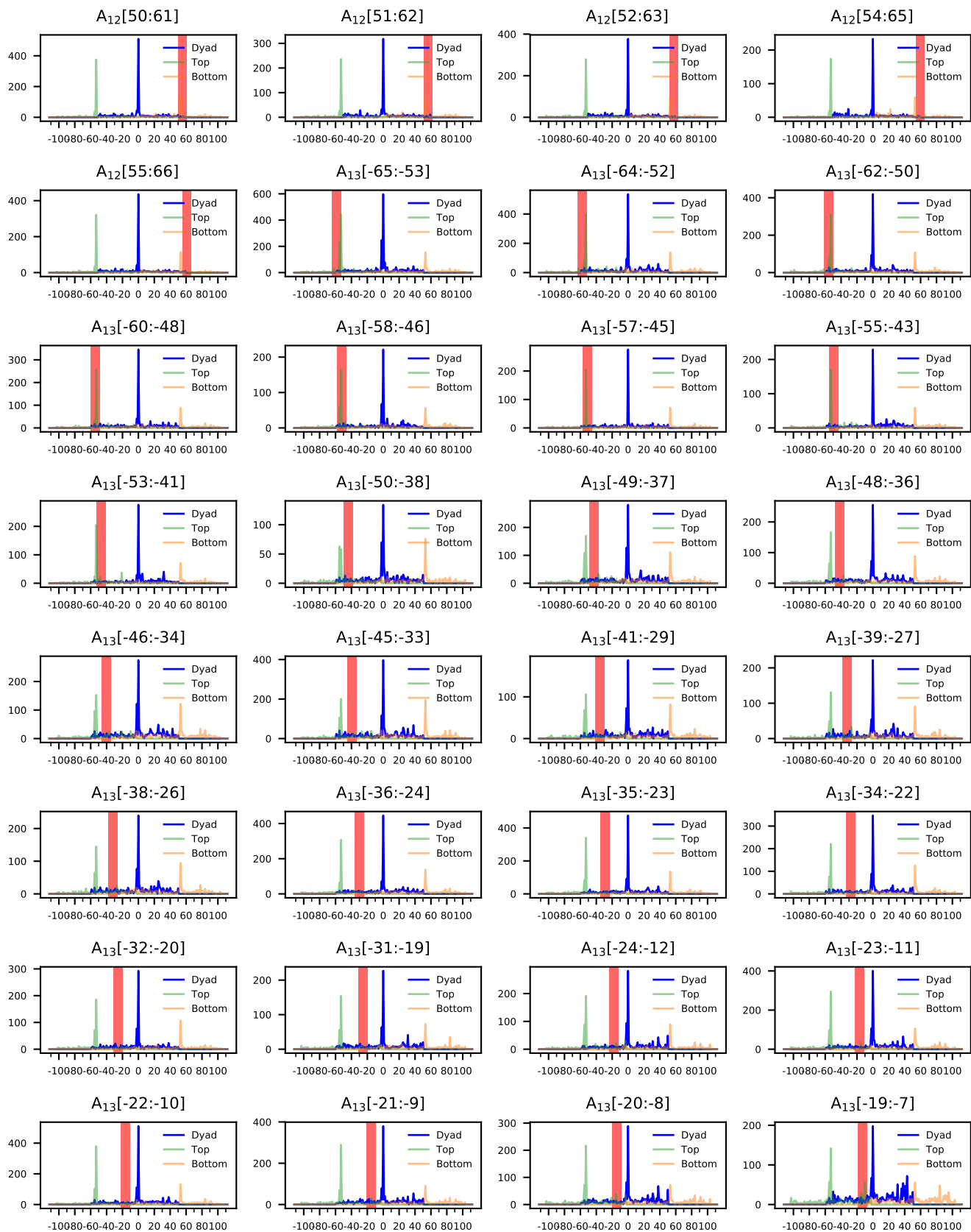

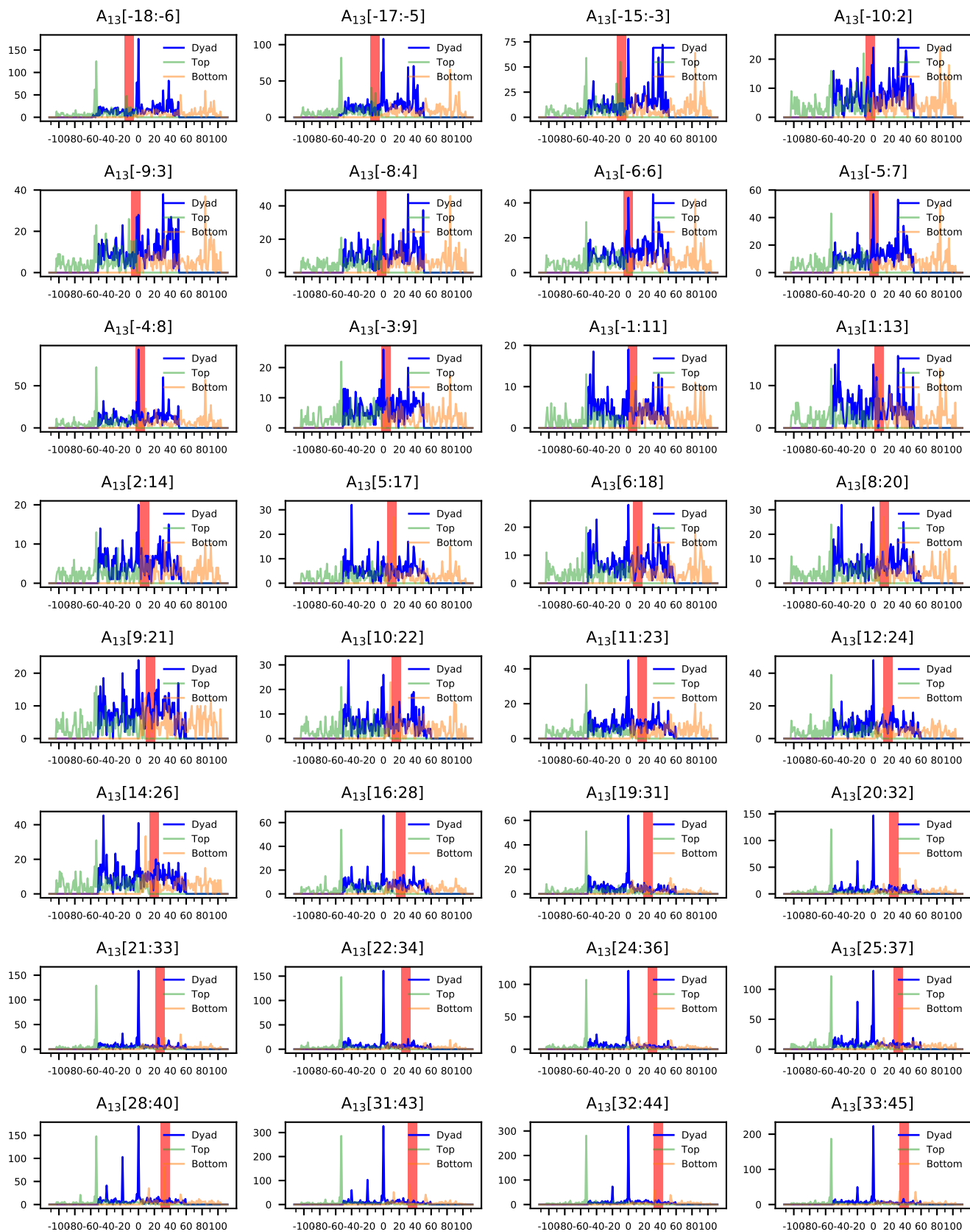

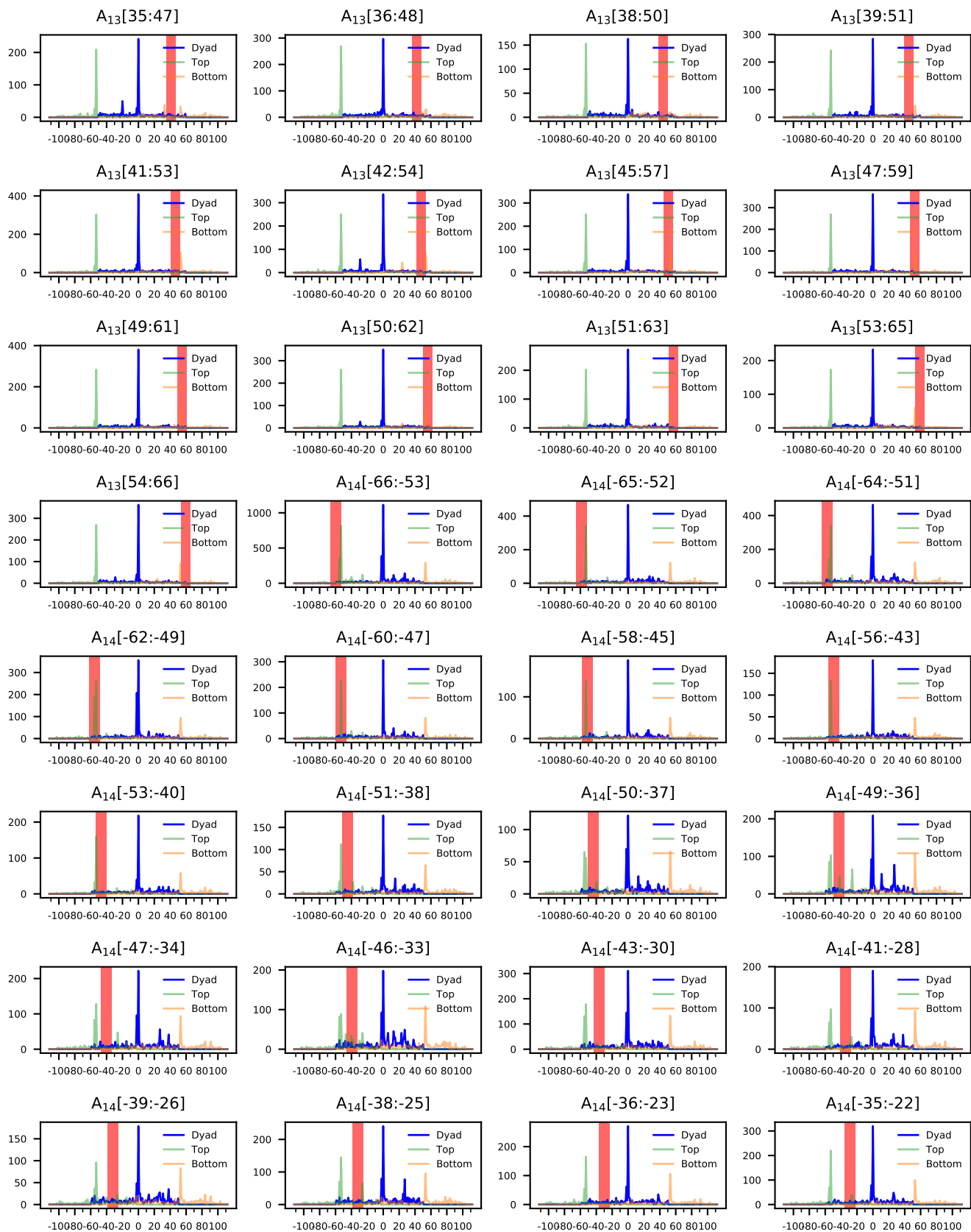

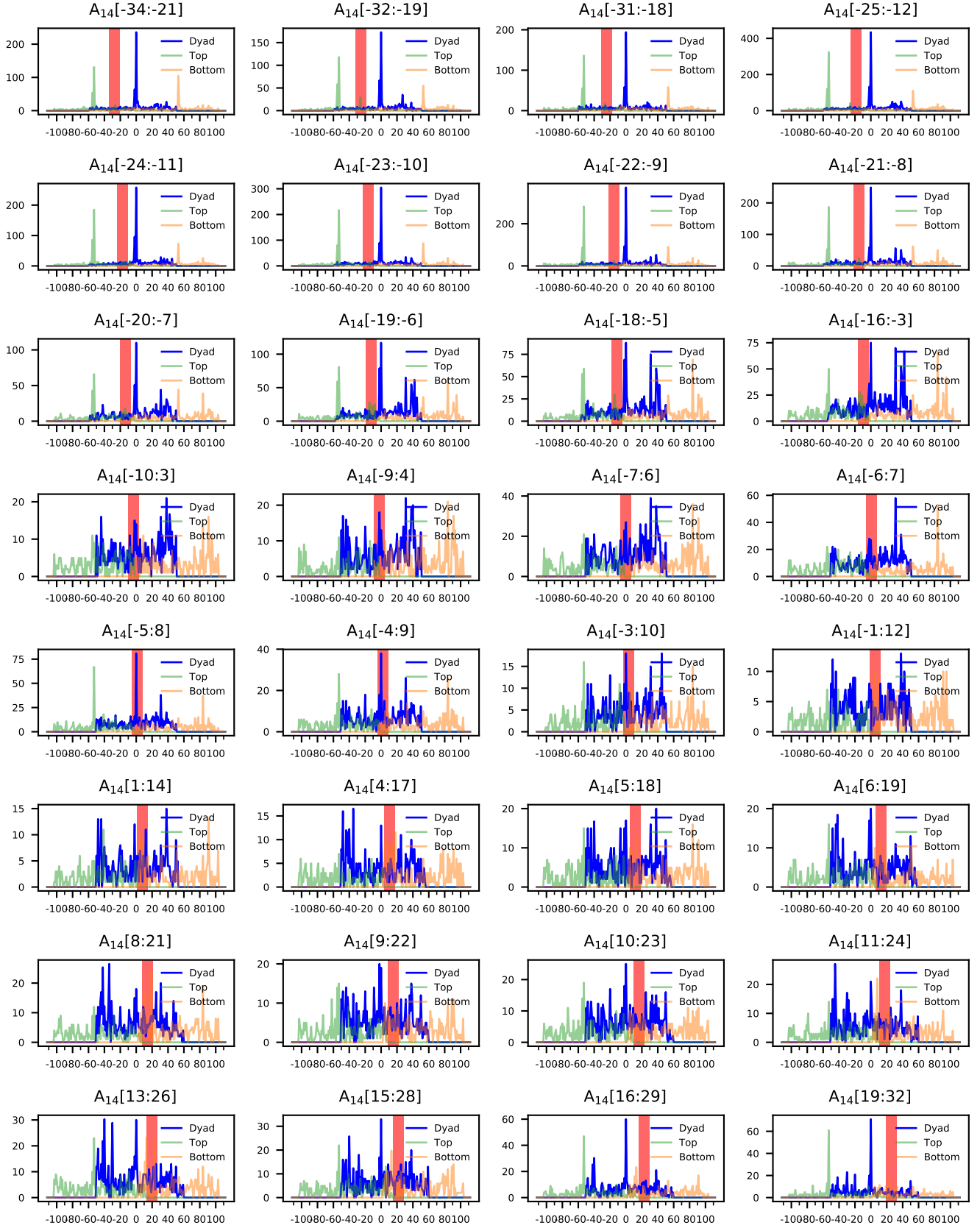

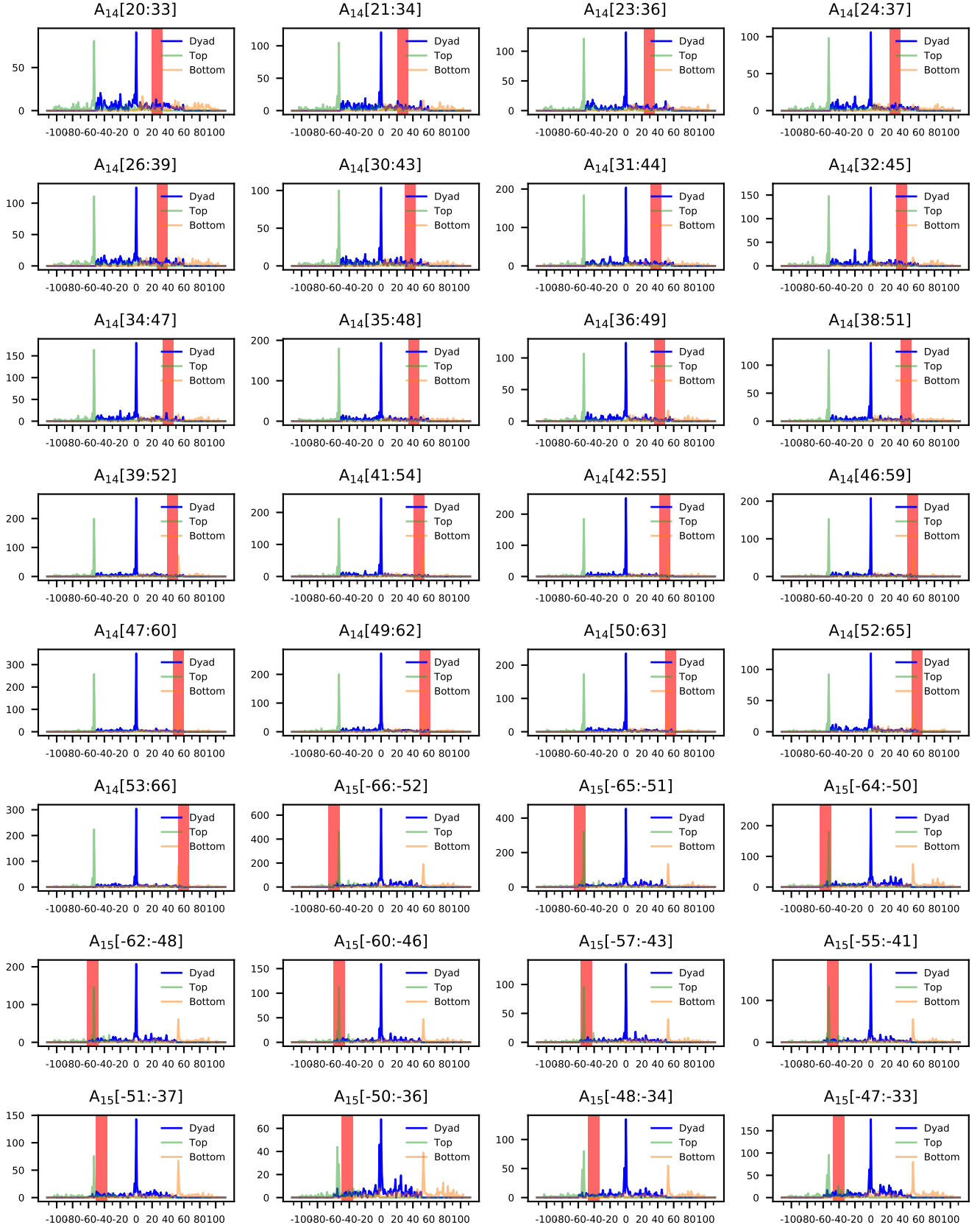

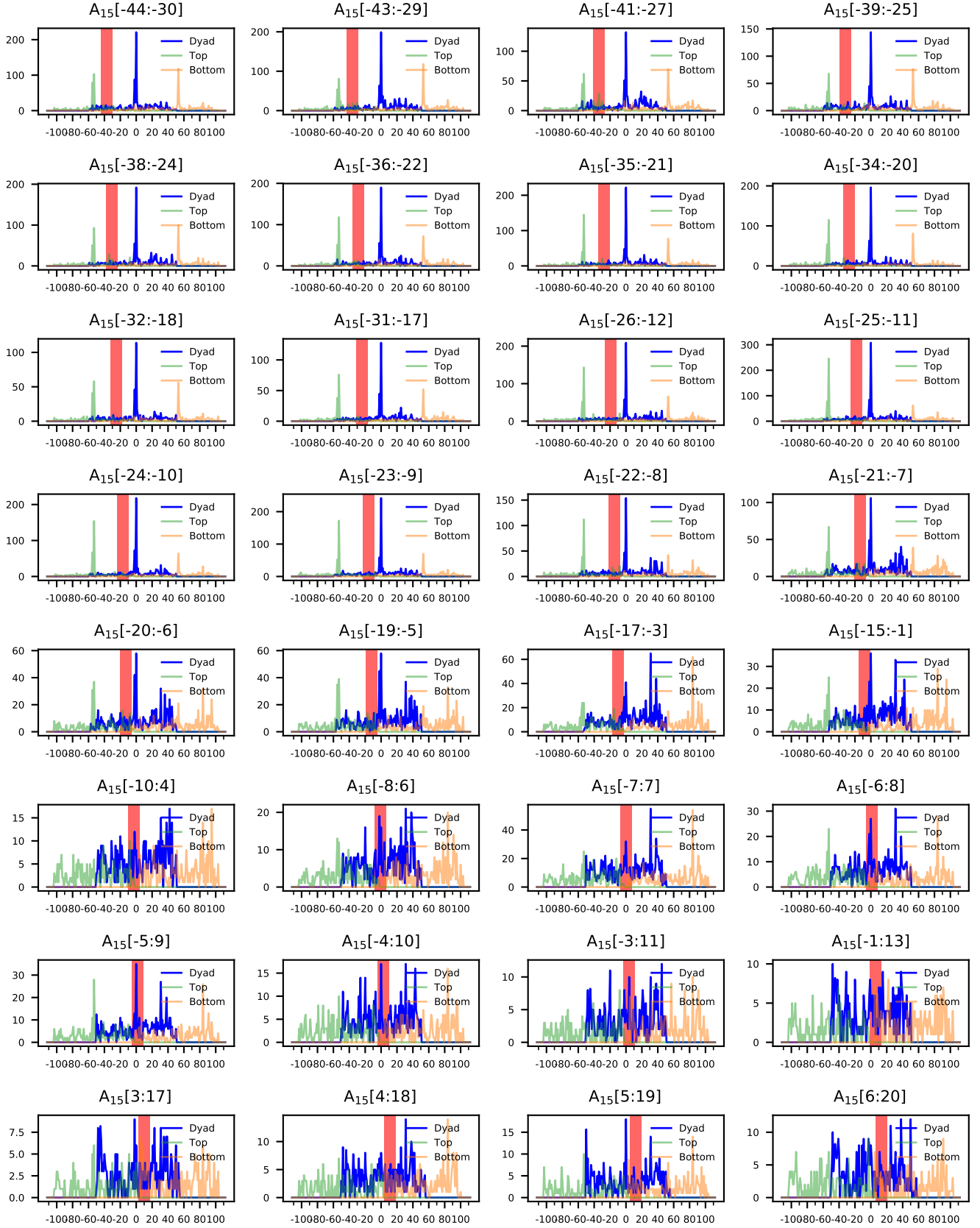

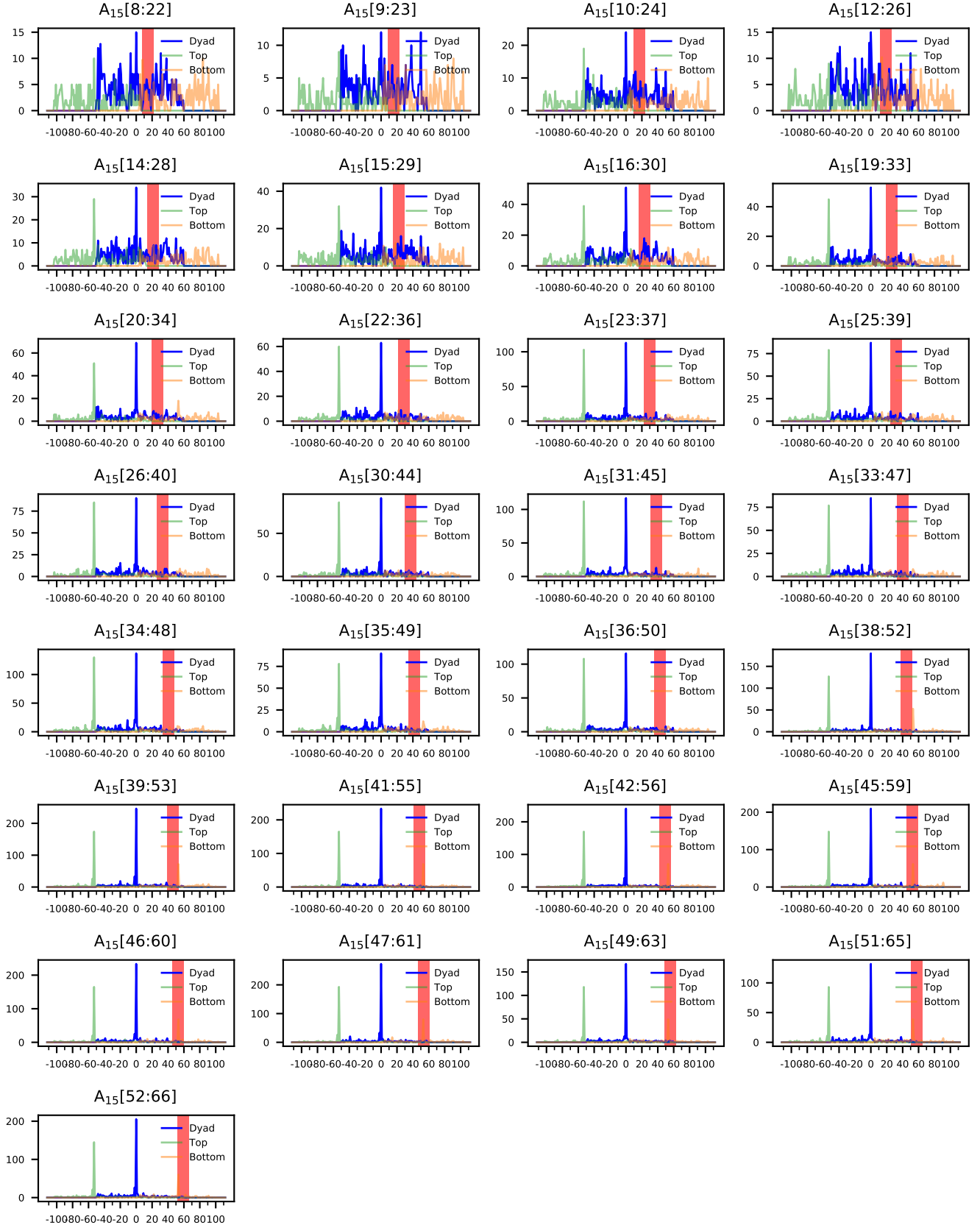

Supplement: gkad738_Supplemental_files [file gkad738_supplemental_files.zip › Supplementary Table 2 (601-Signals_A_before).pdf]

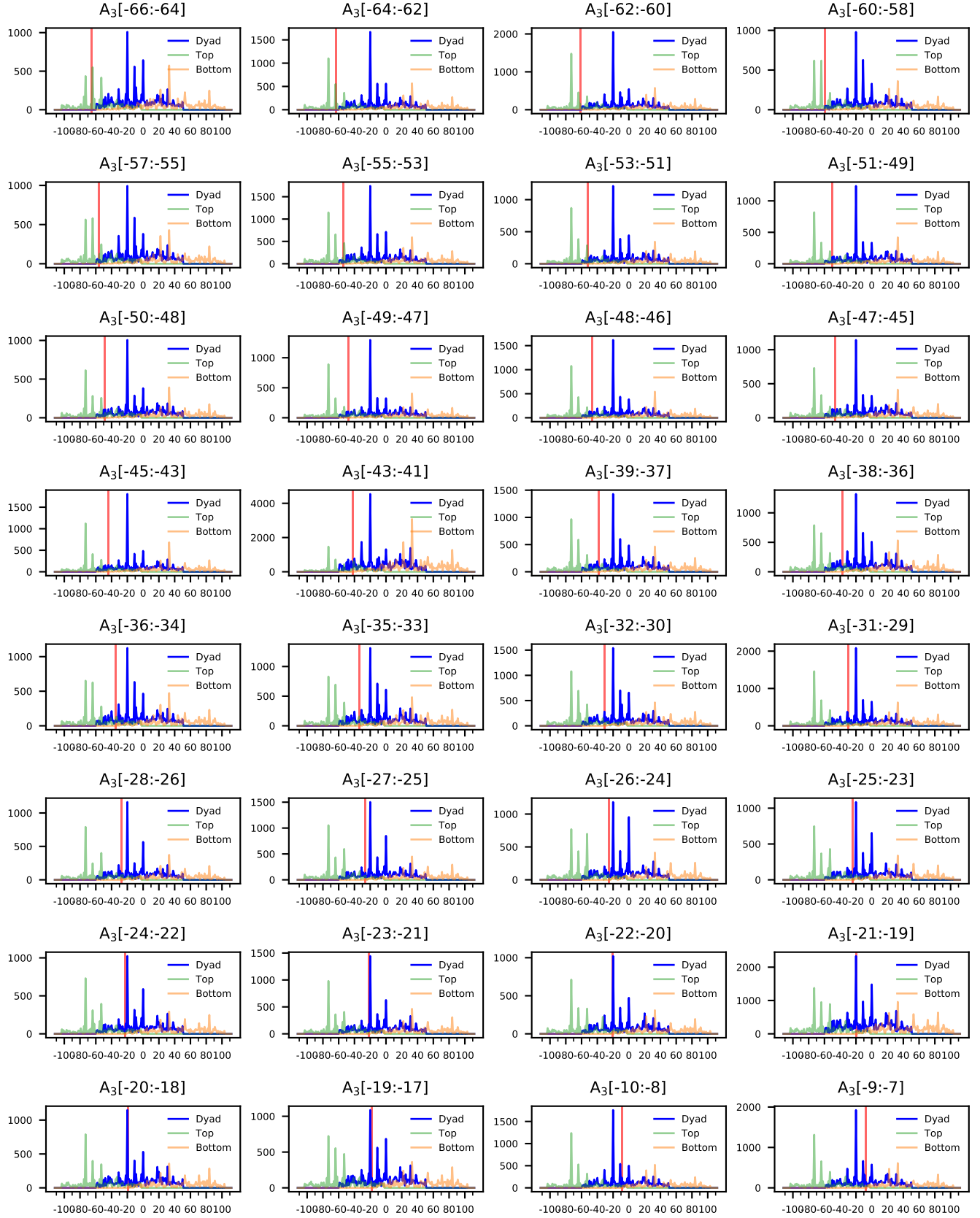

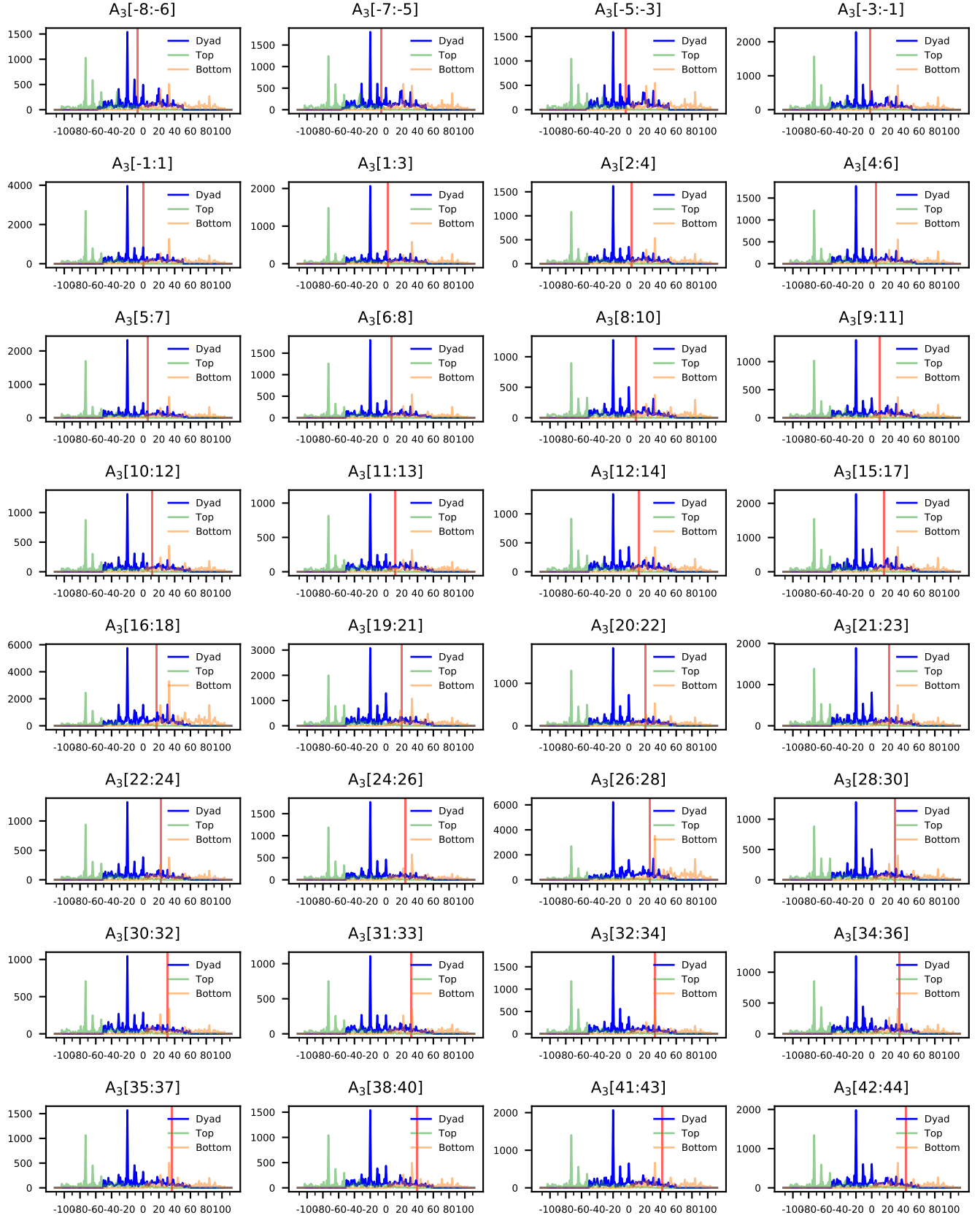

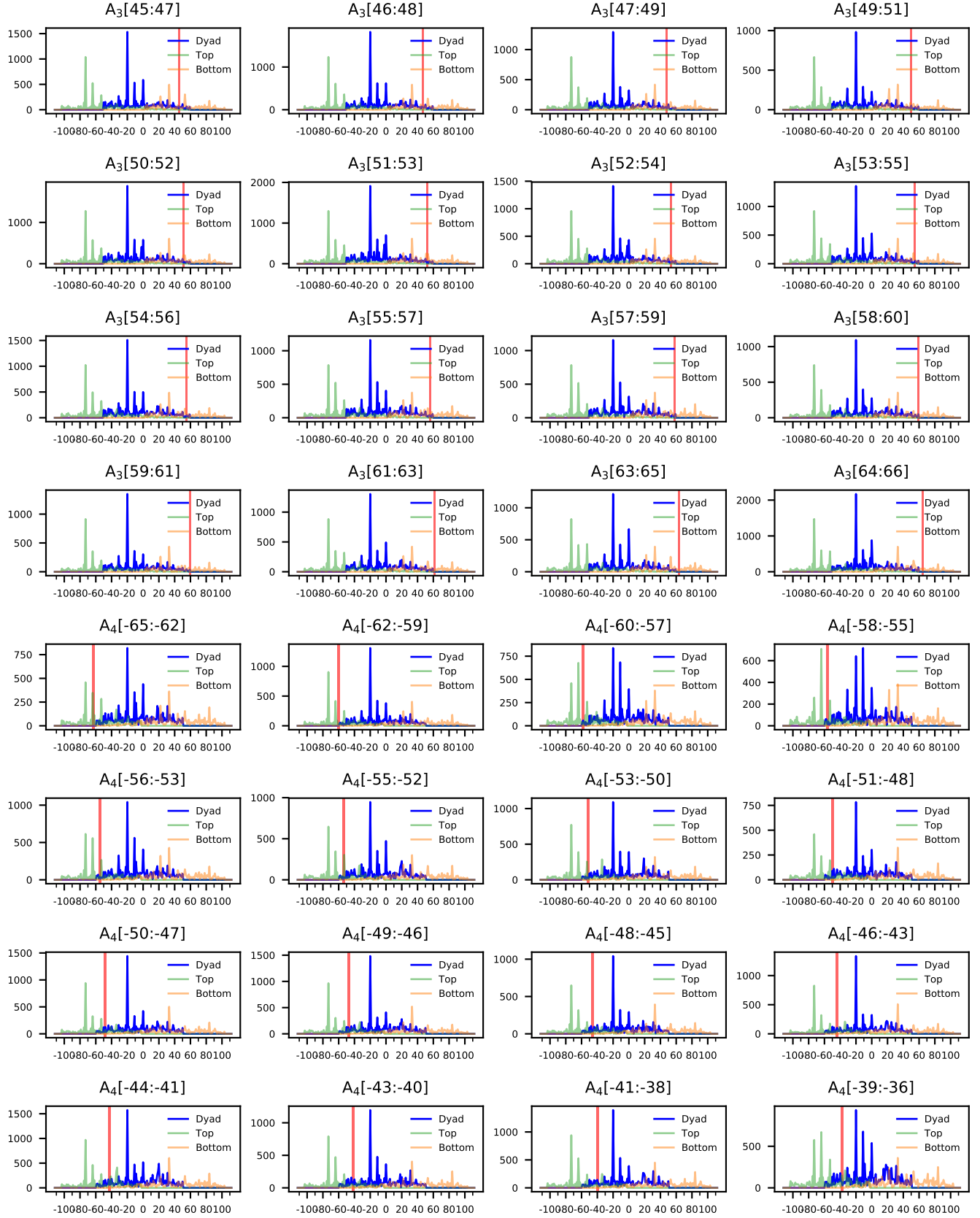

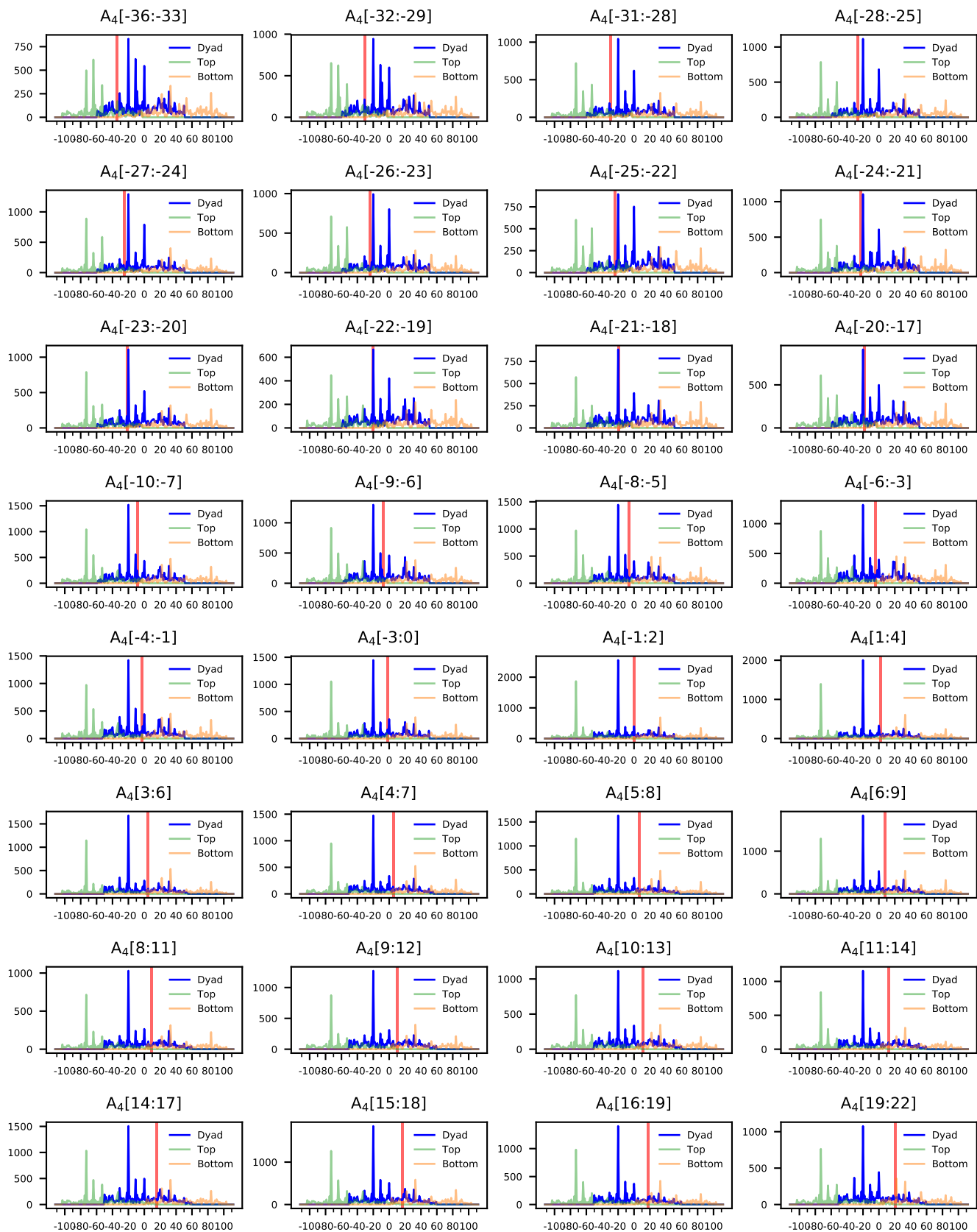

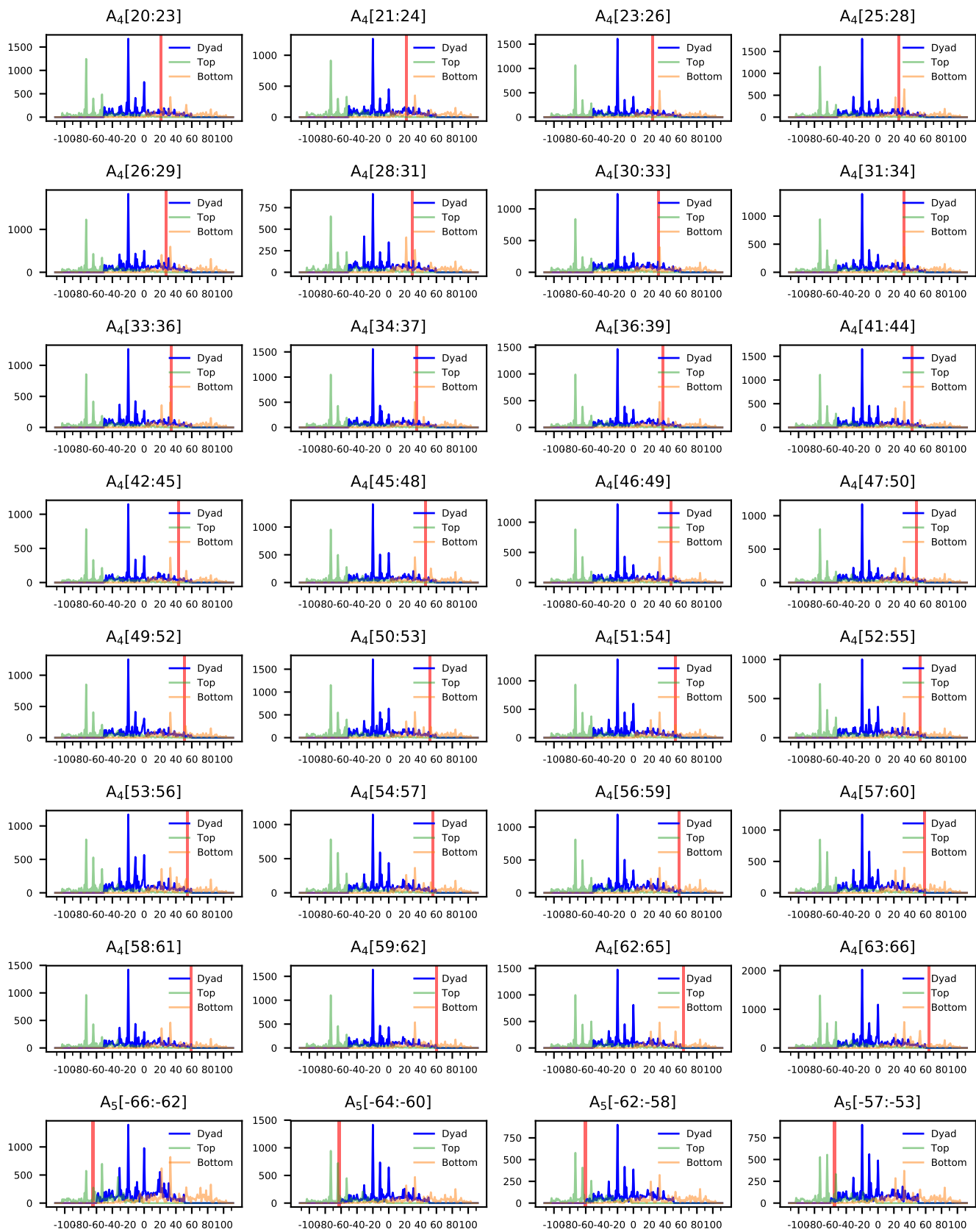

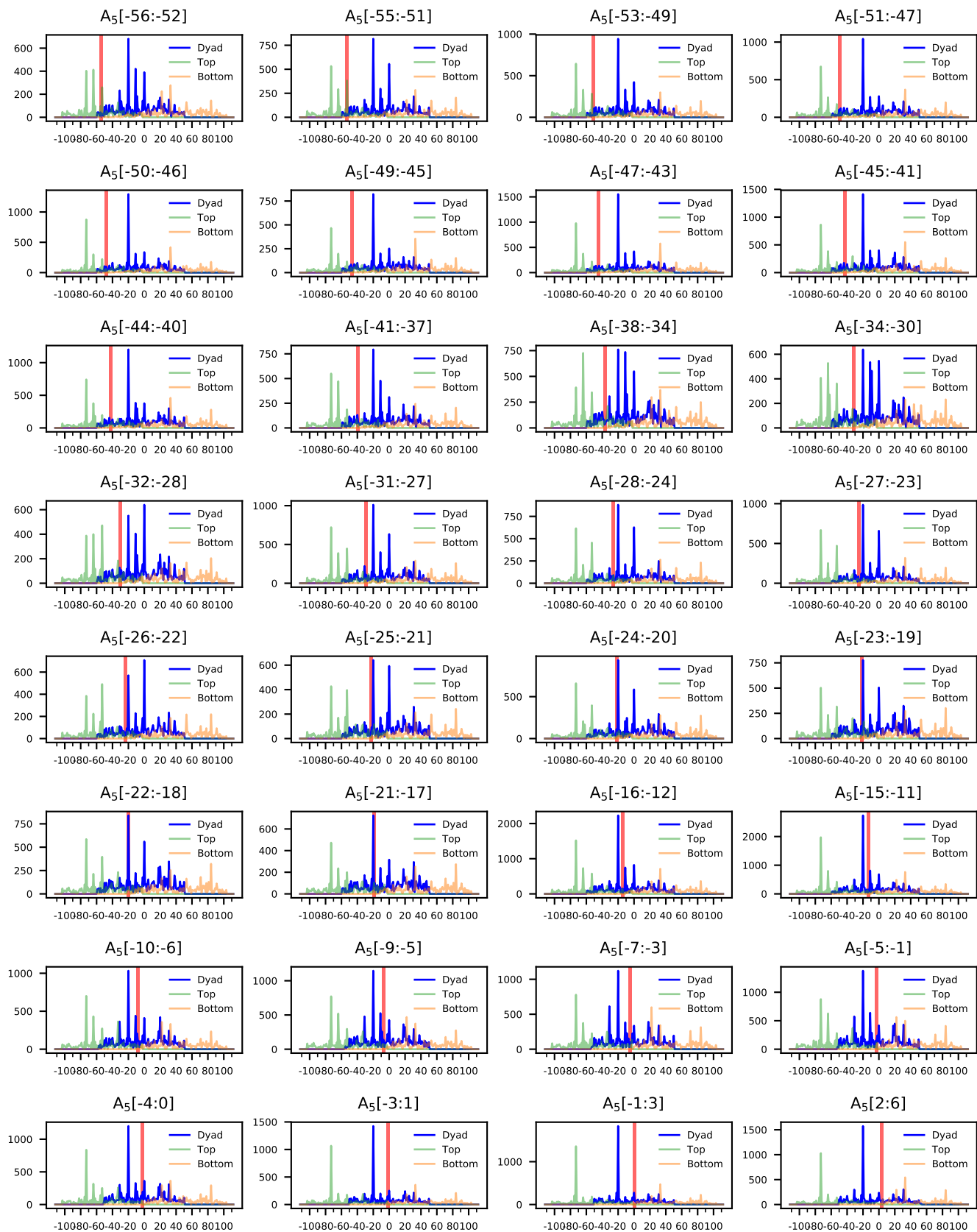

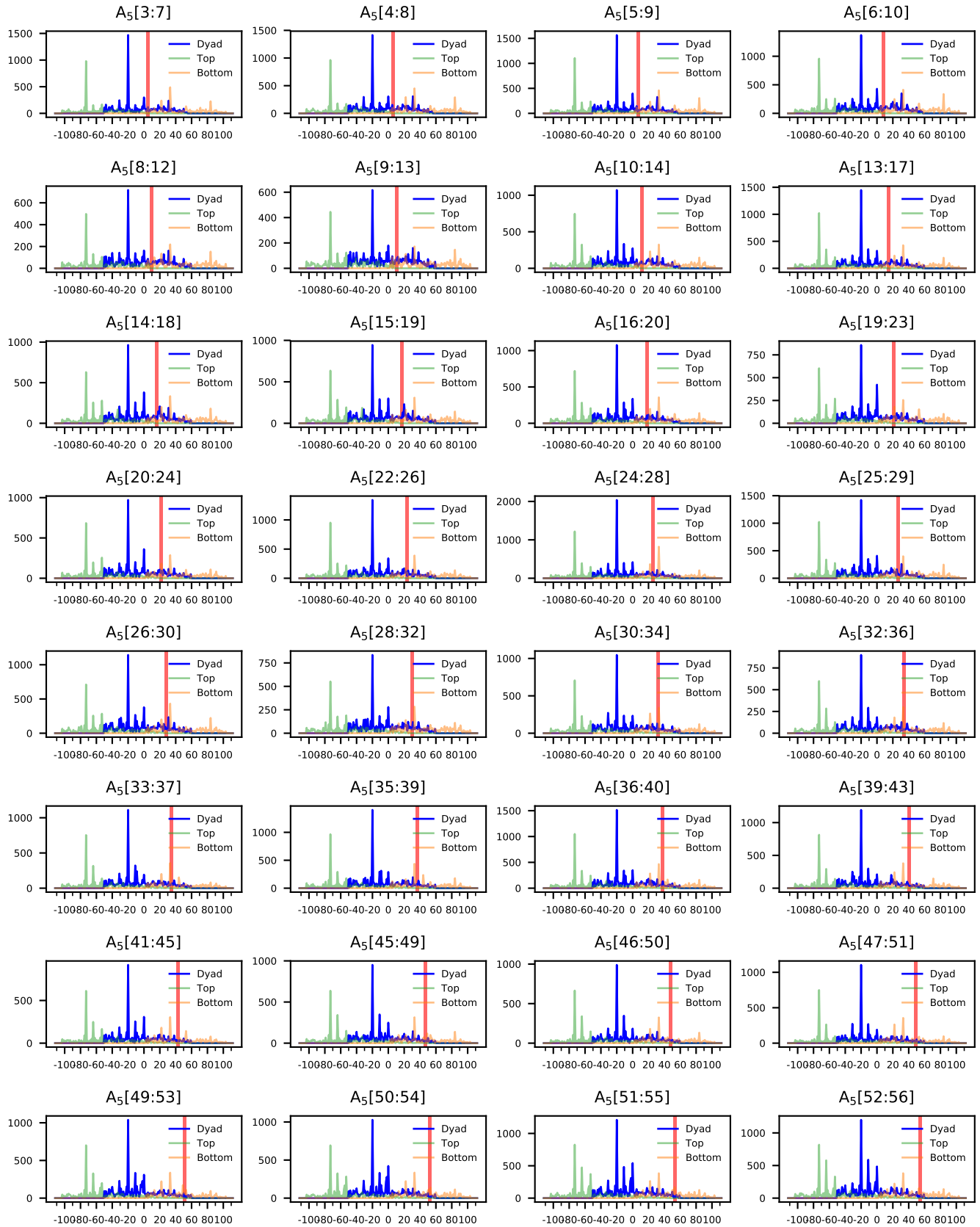

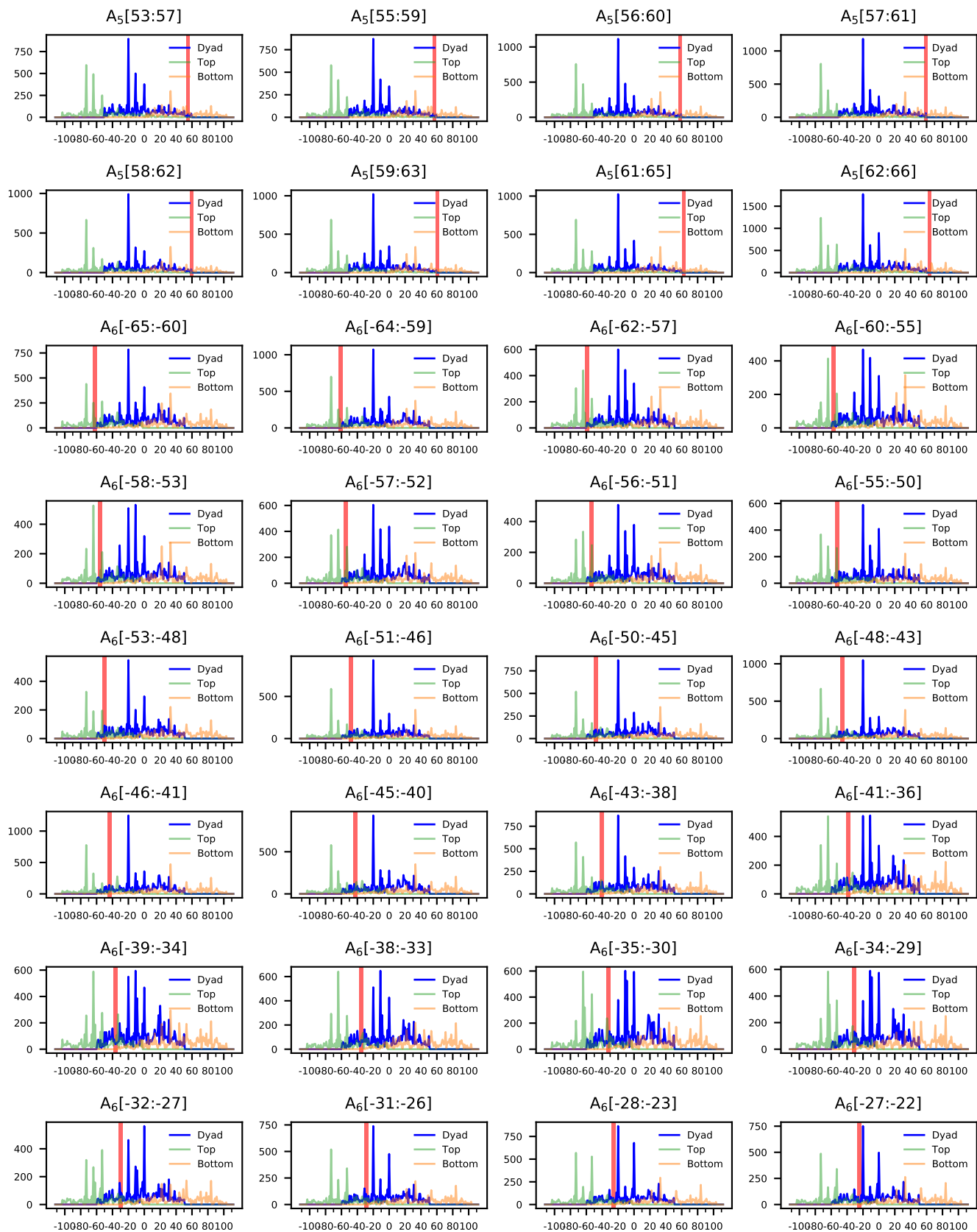

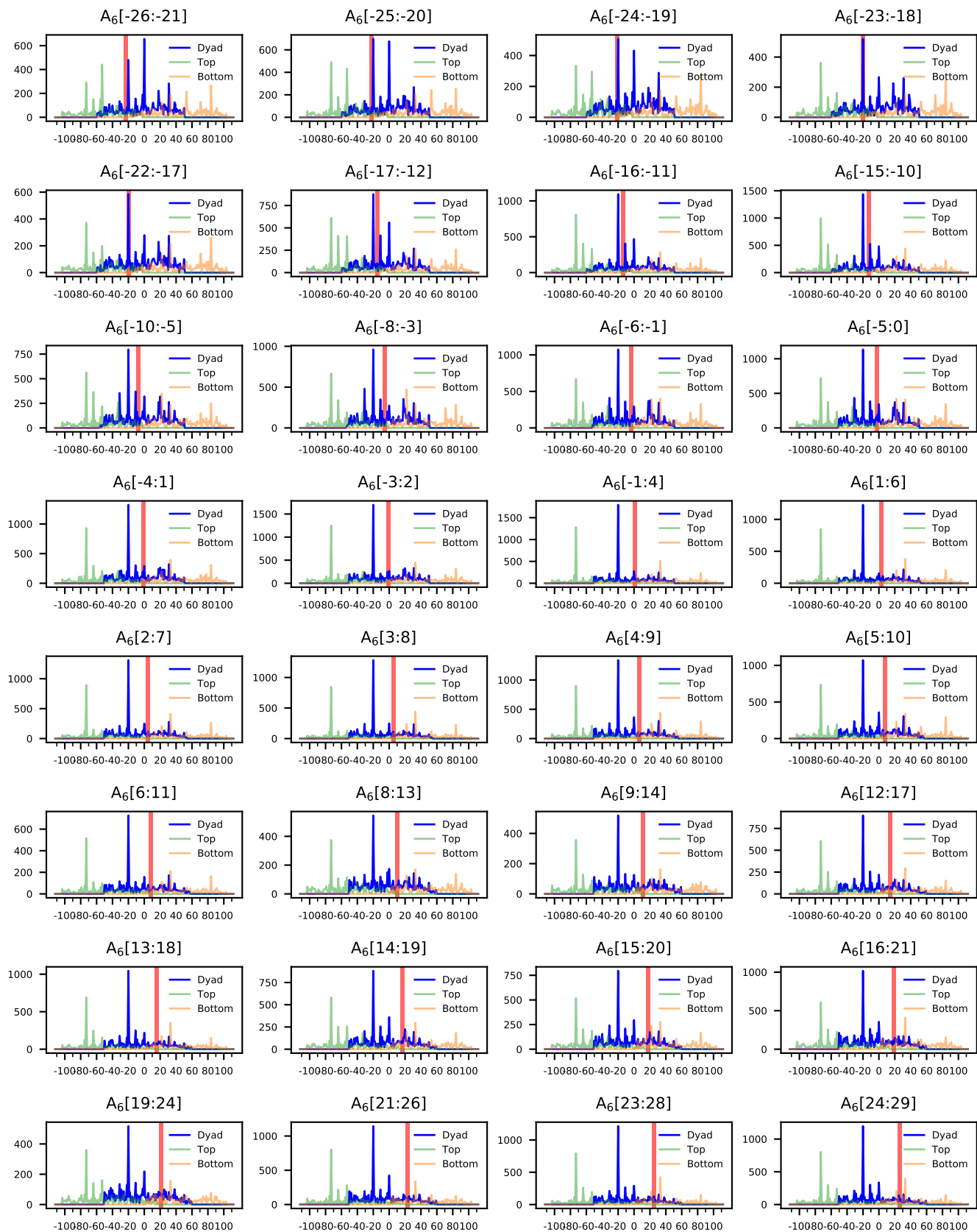

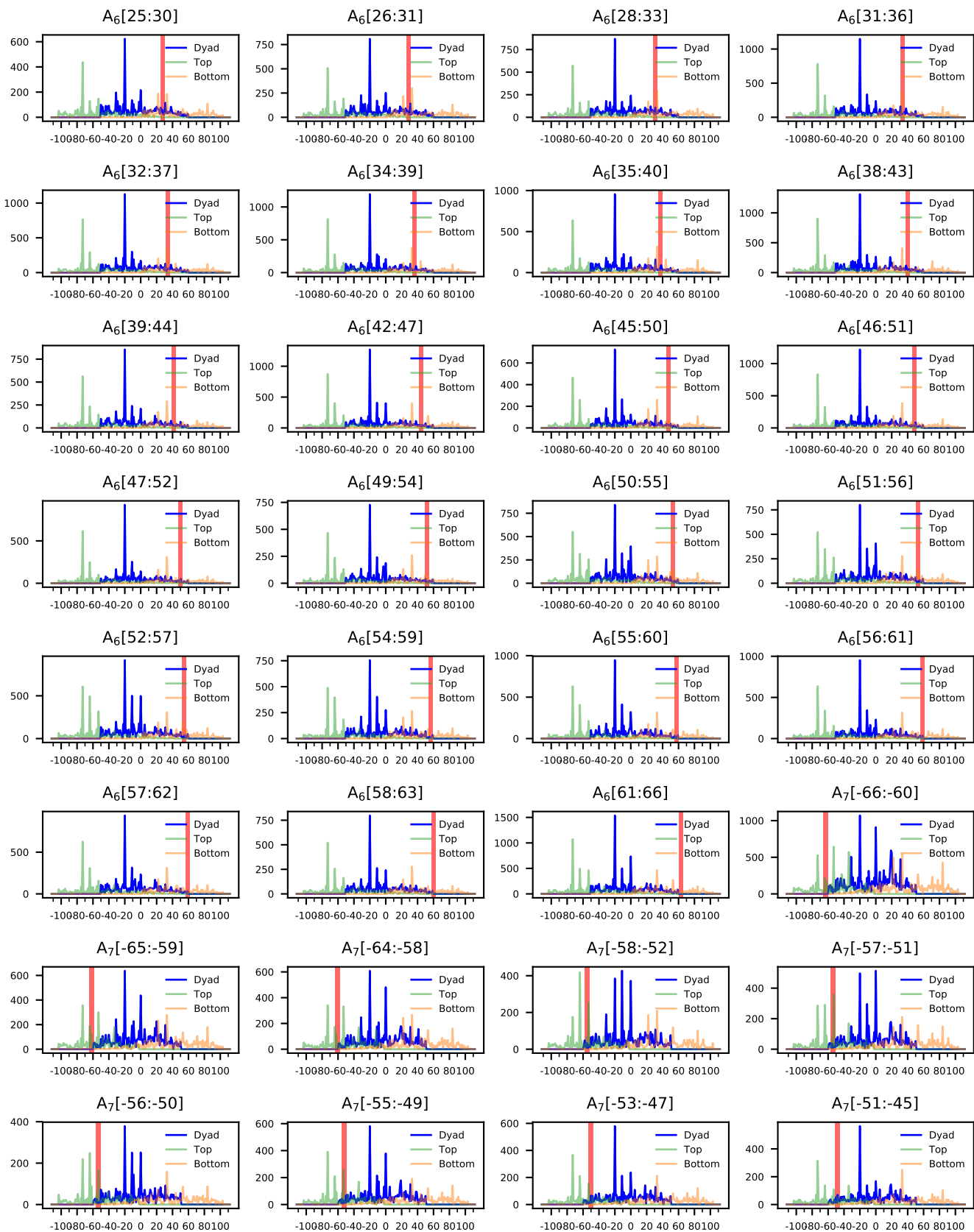

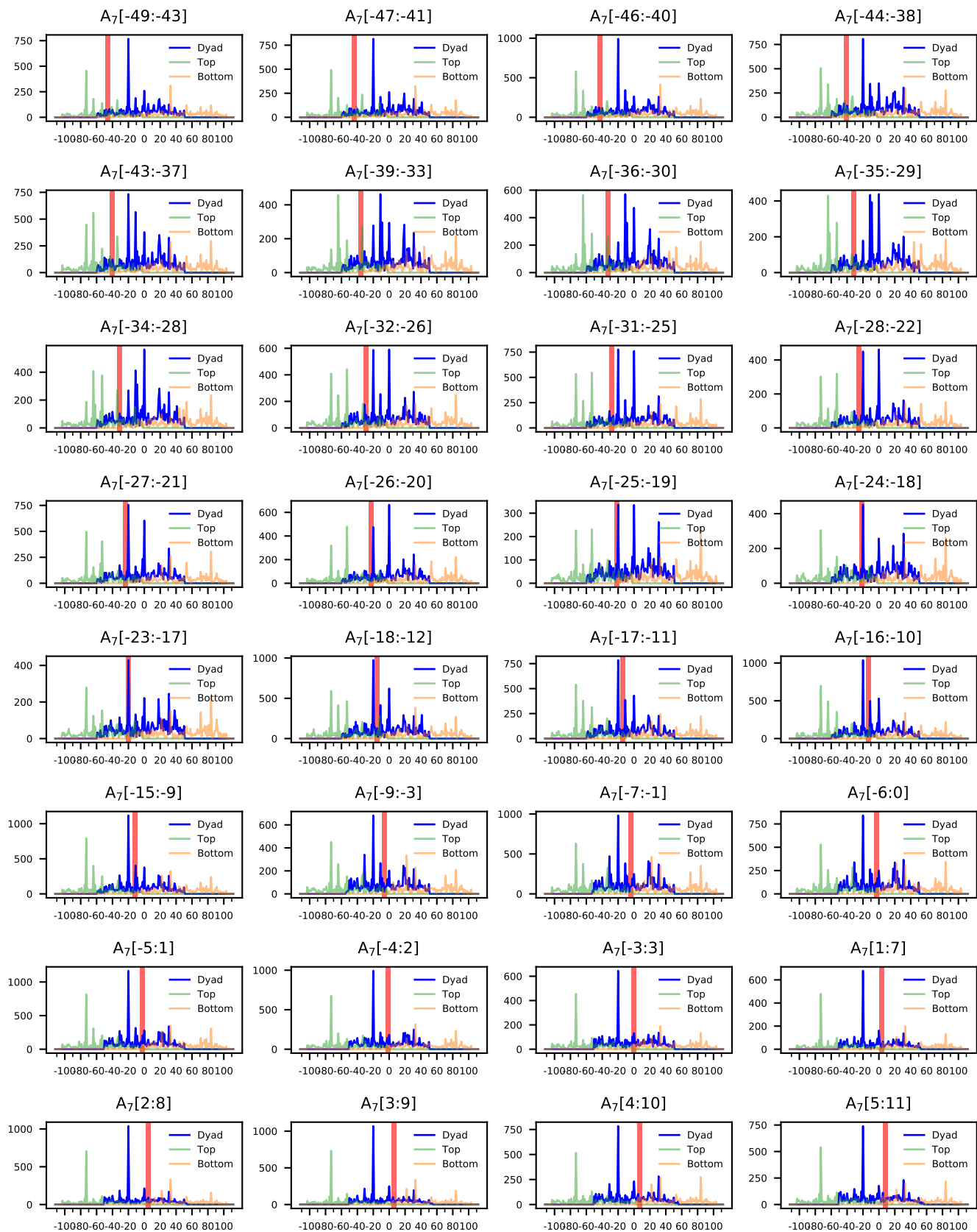

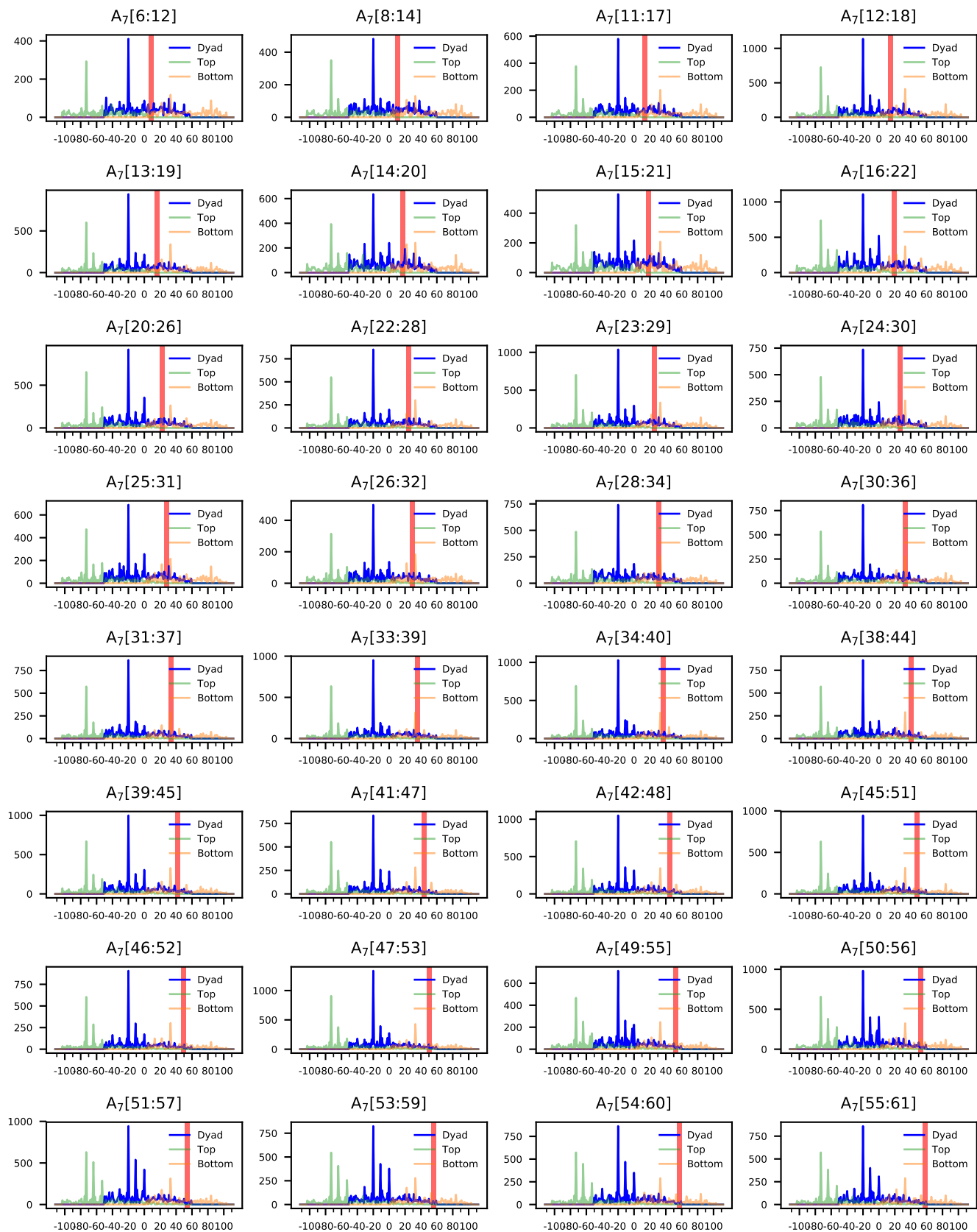

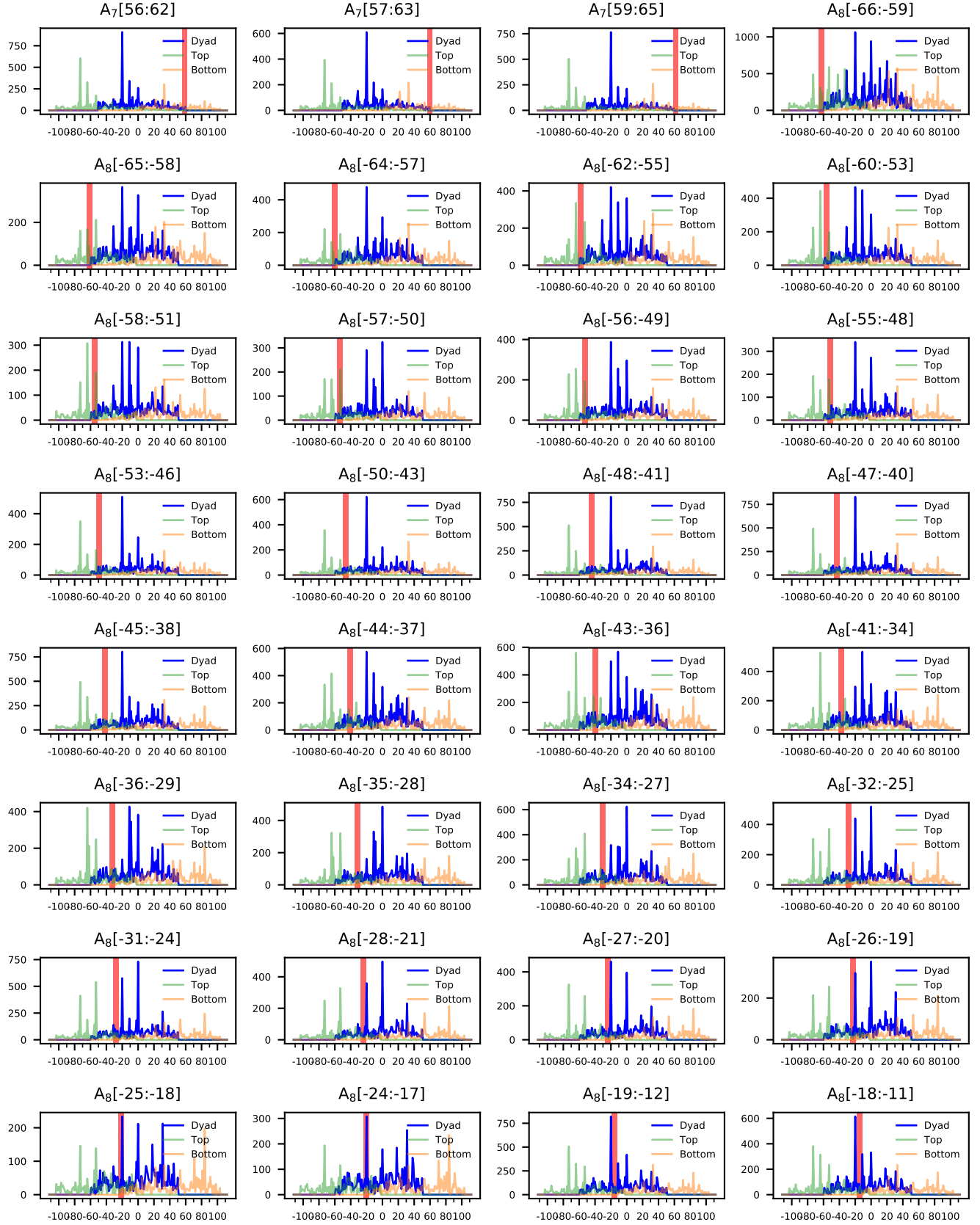

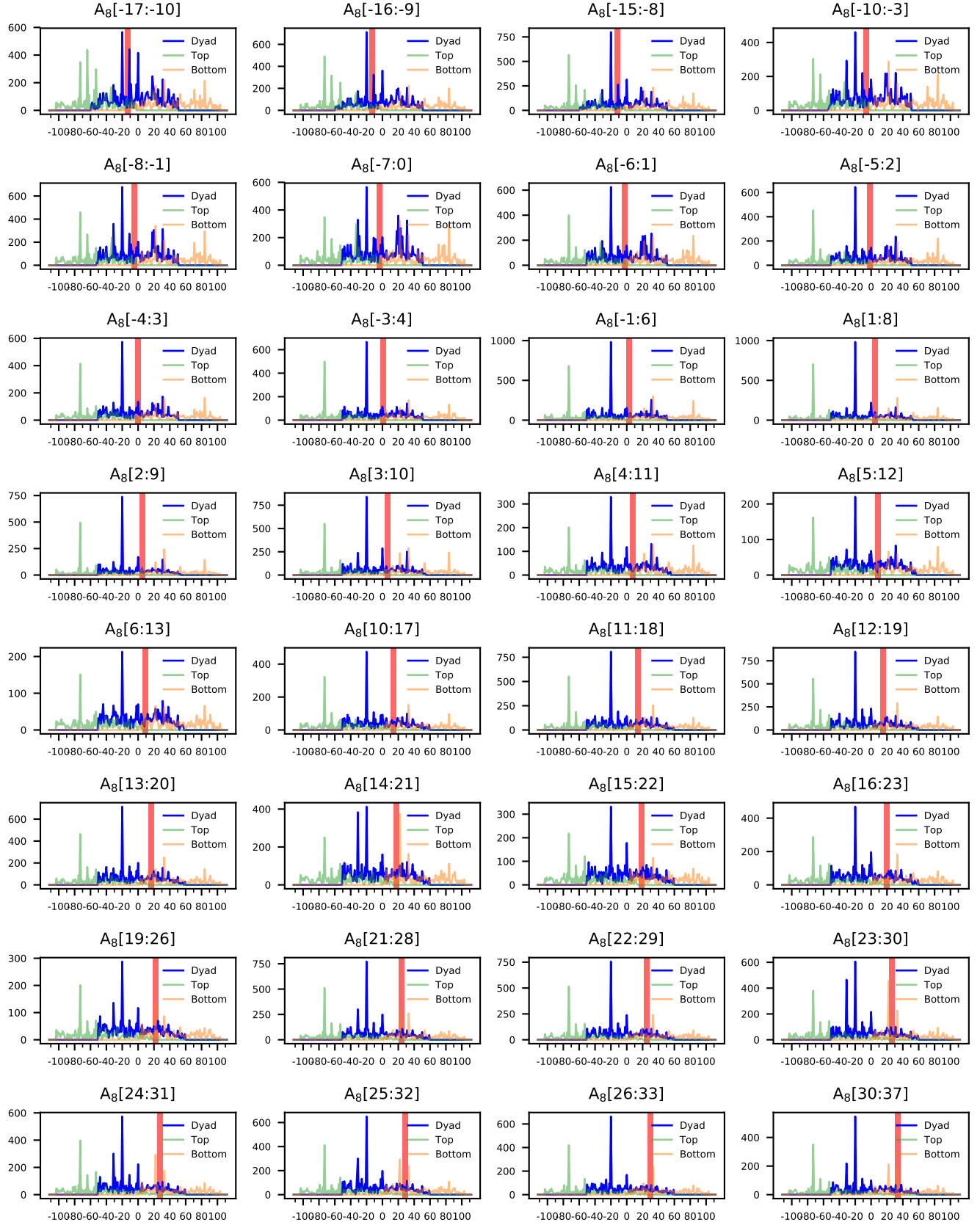

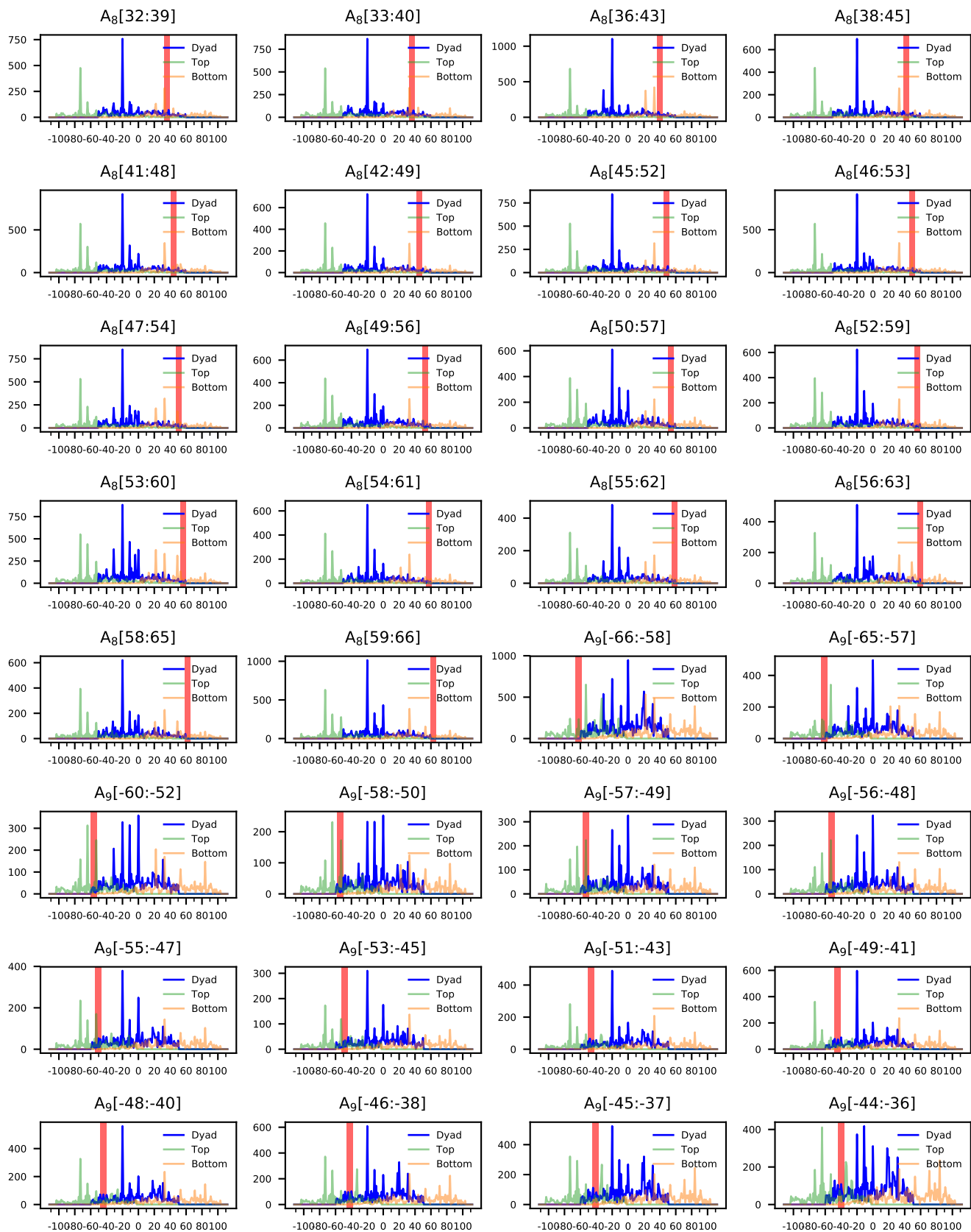

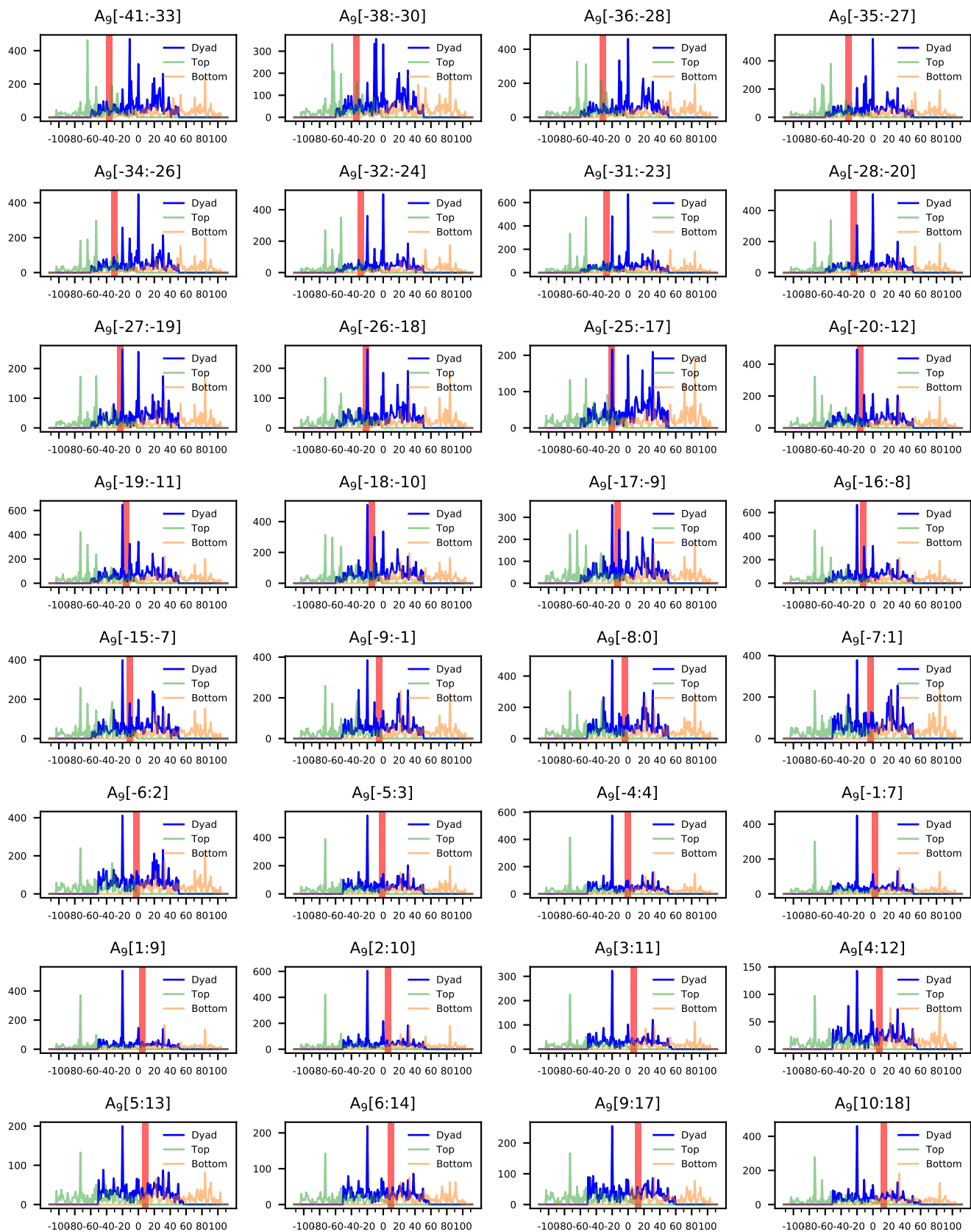

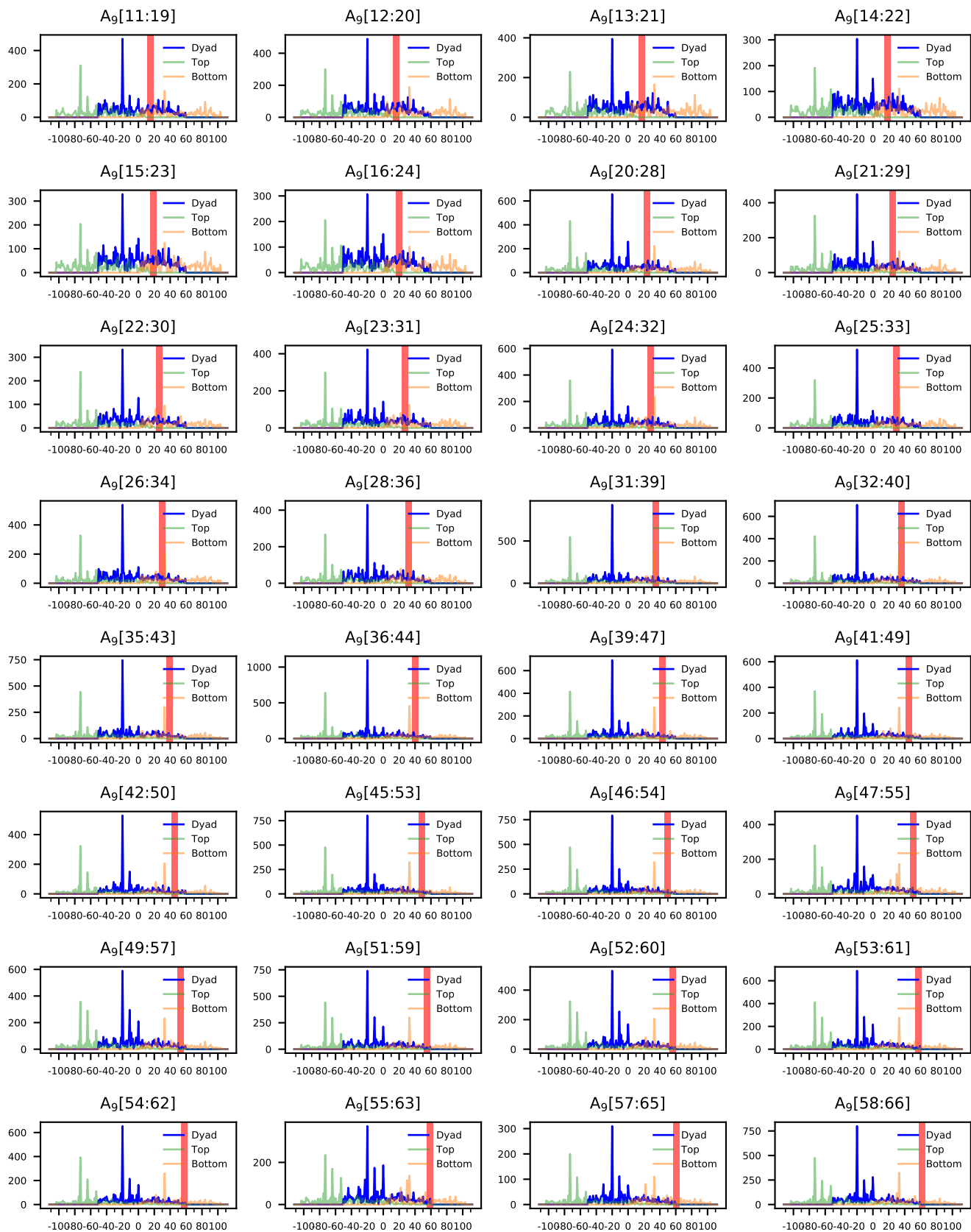

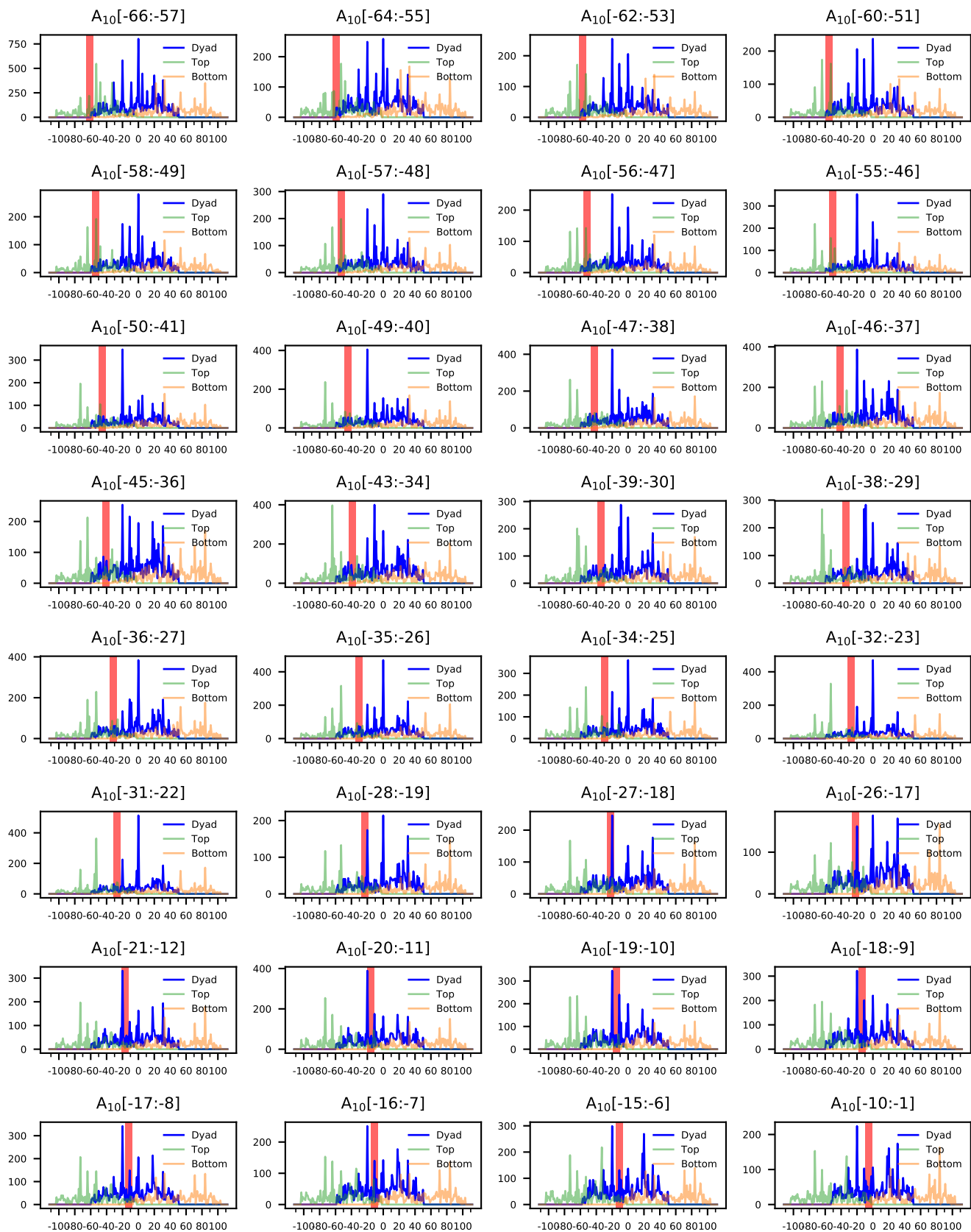

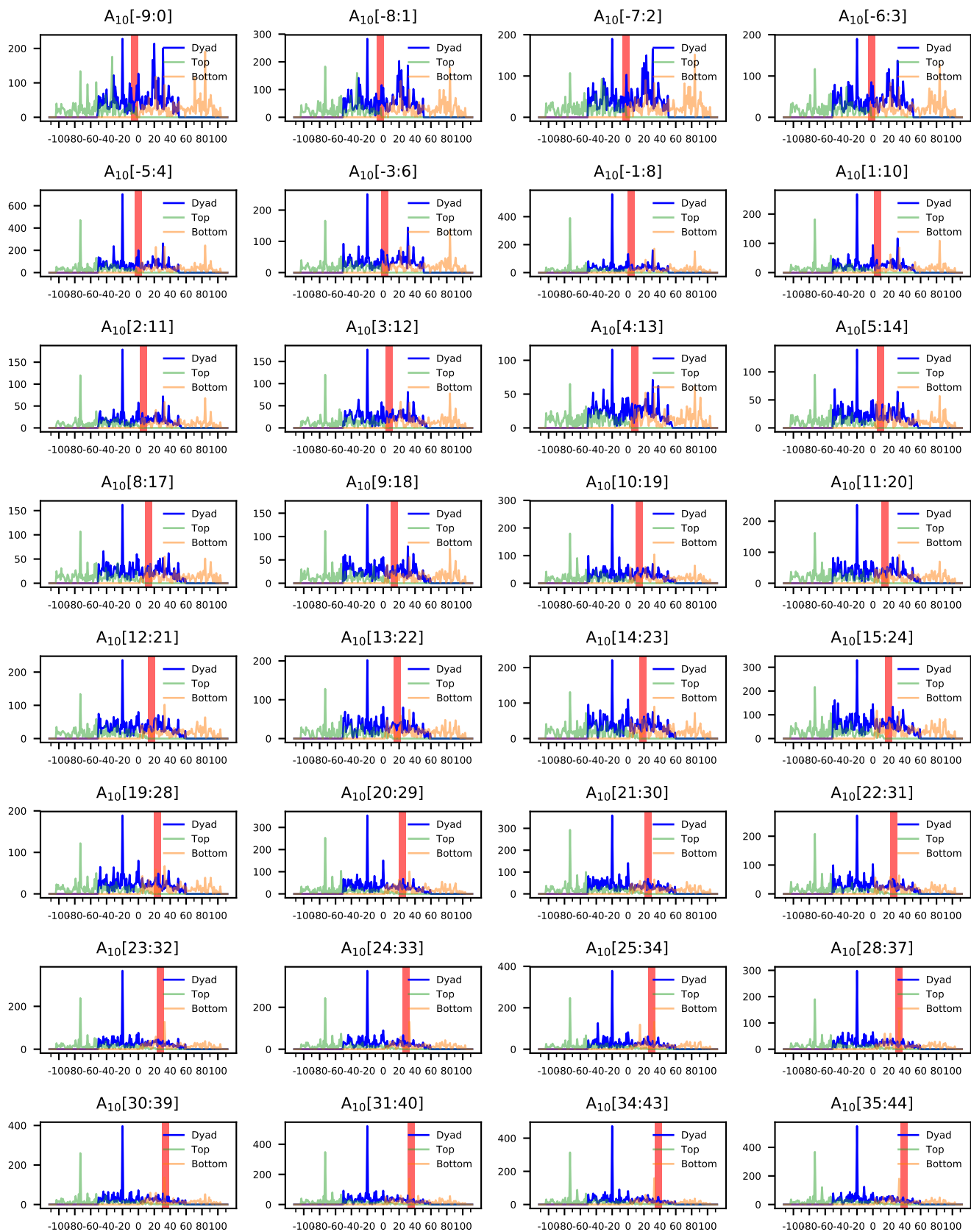

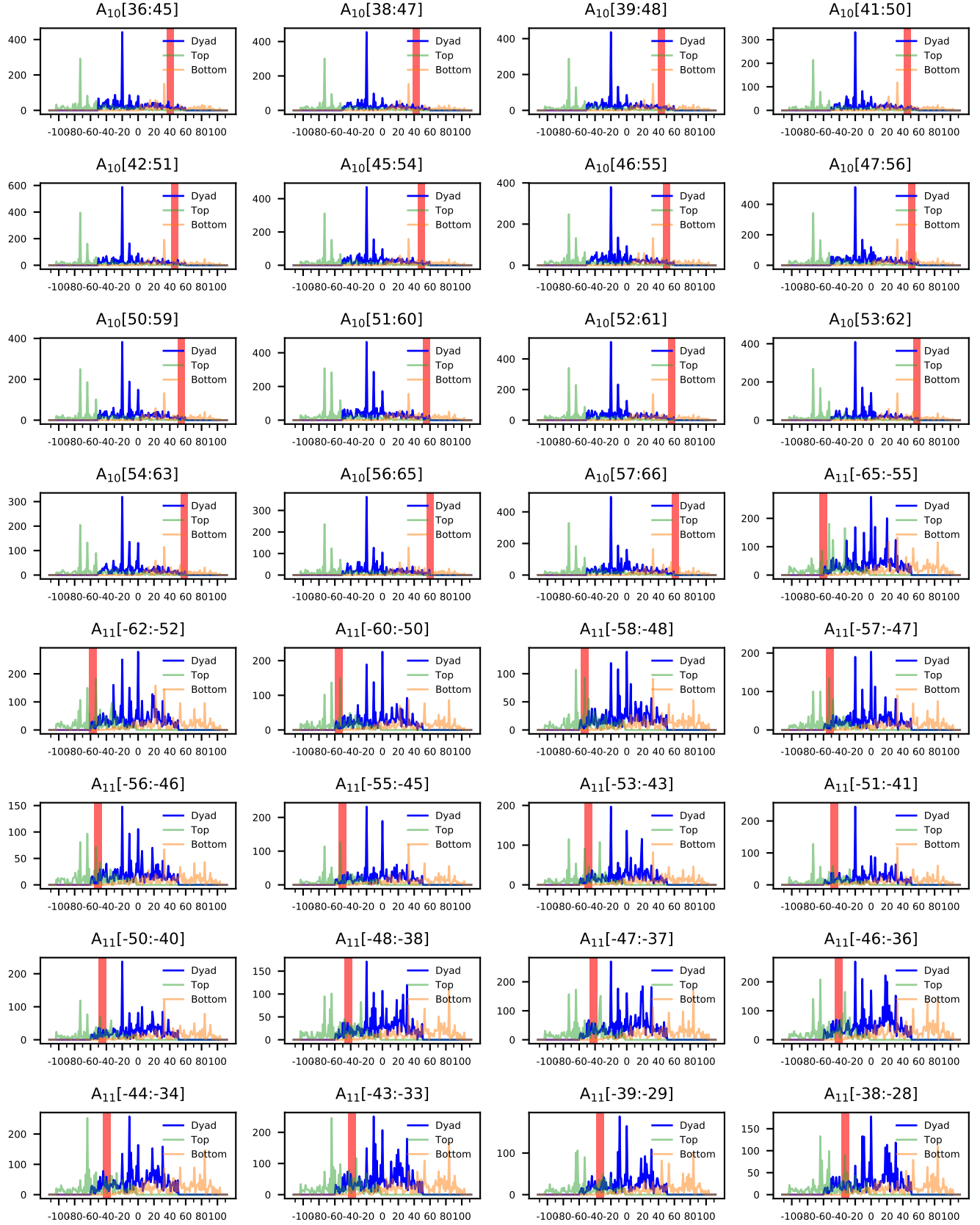

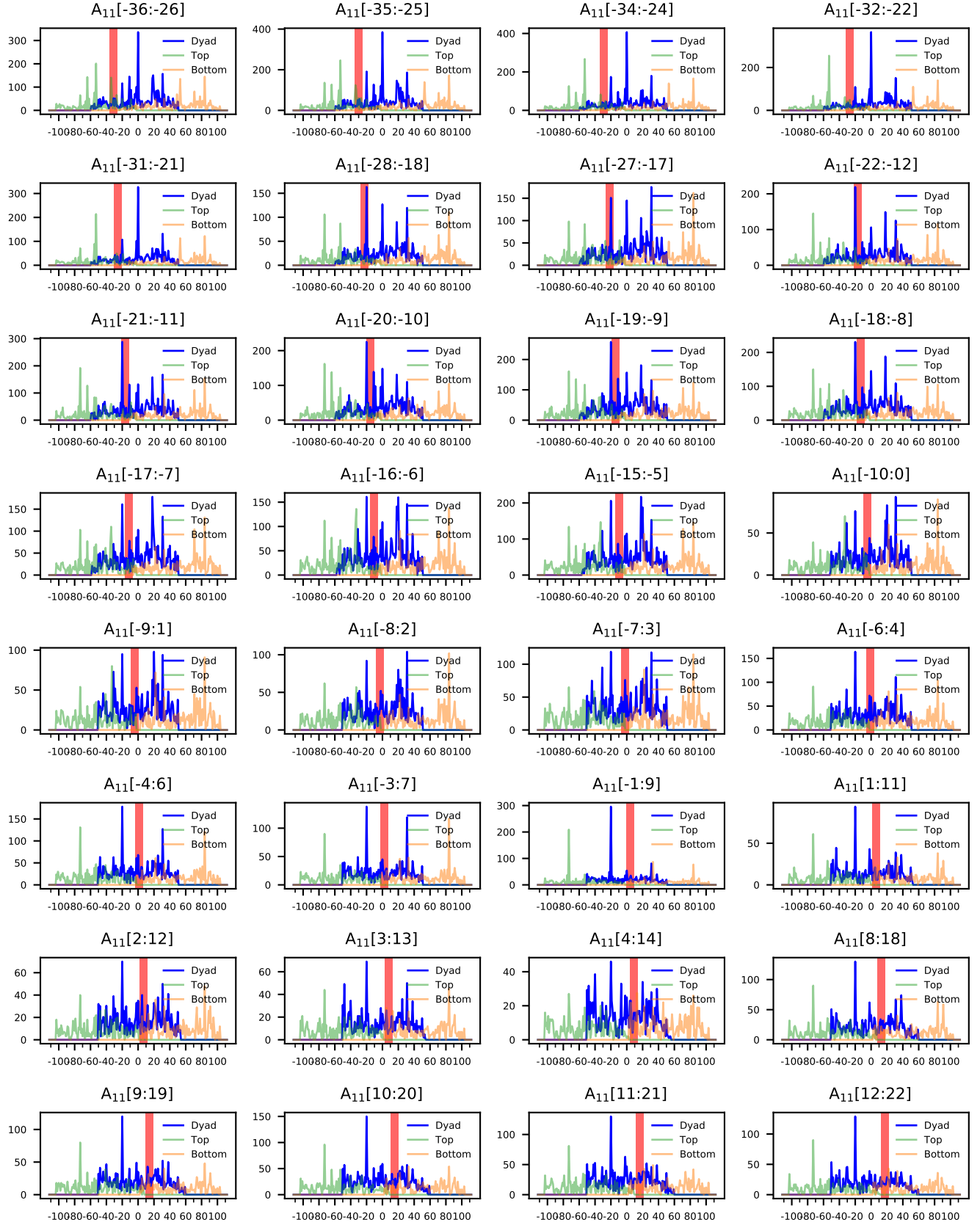

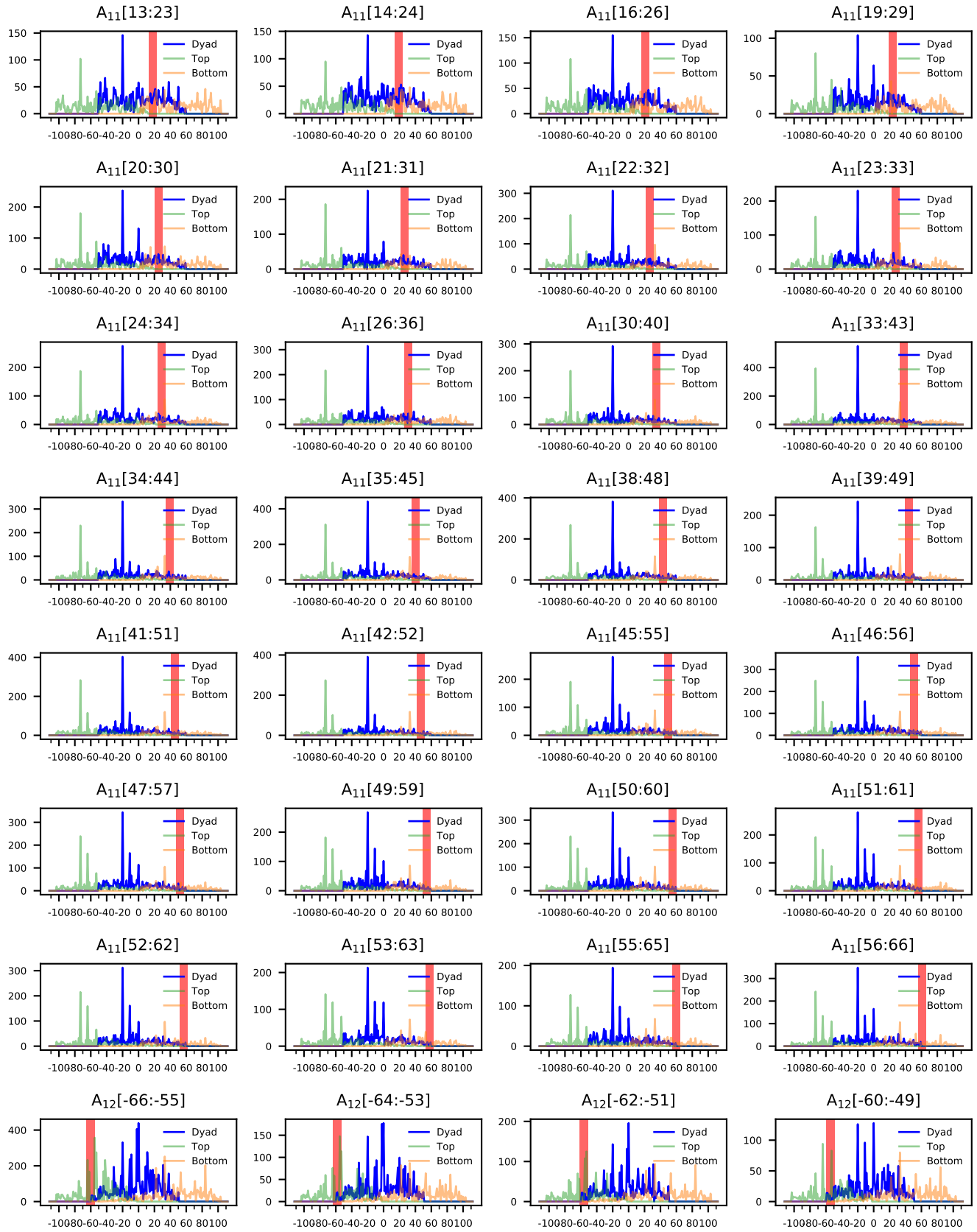

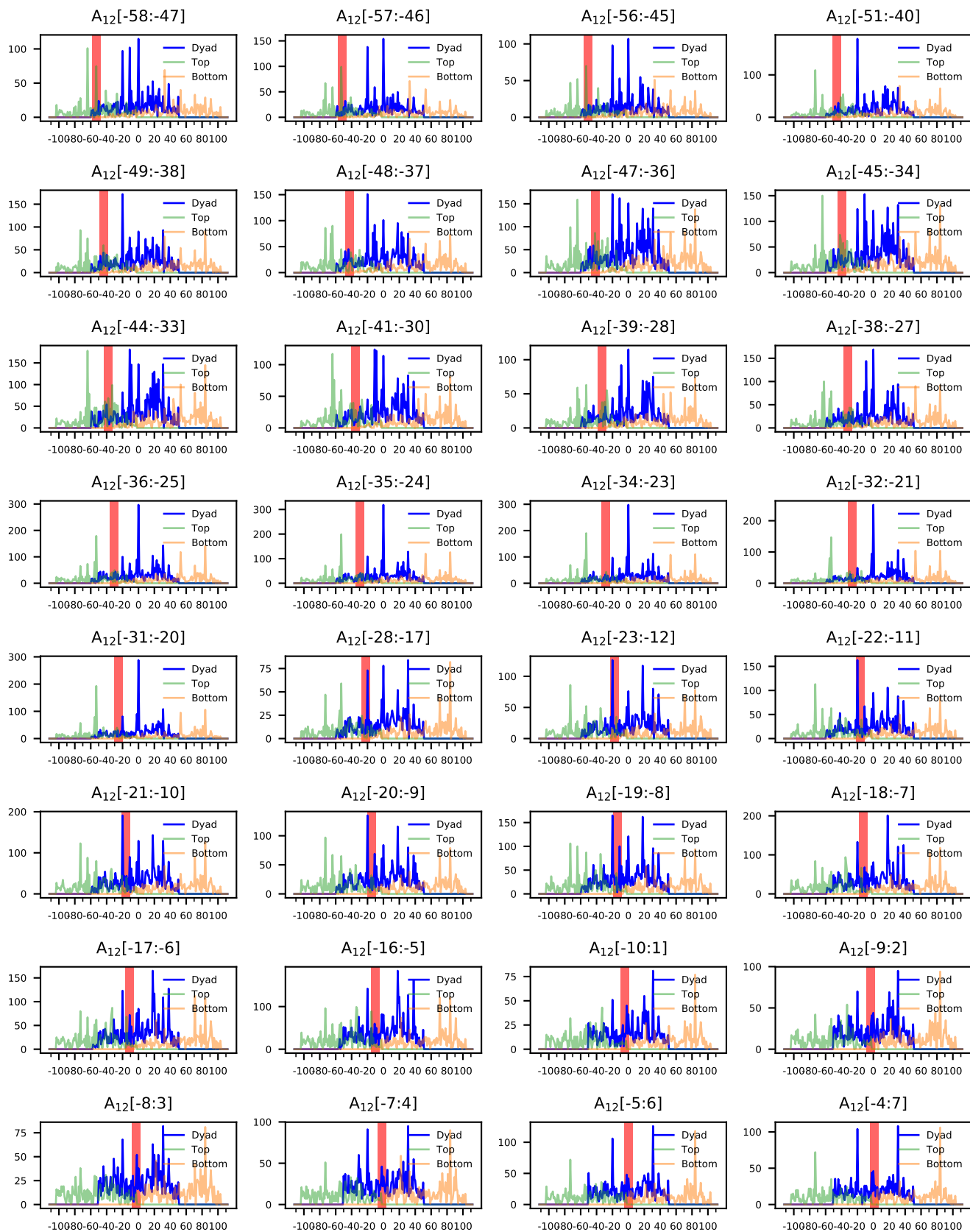

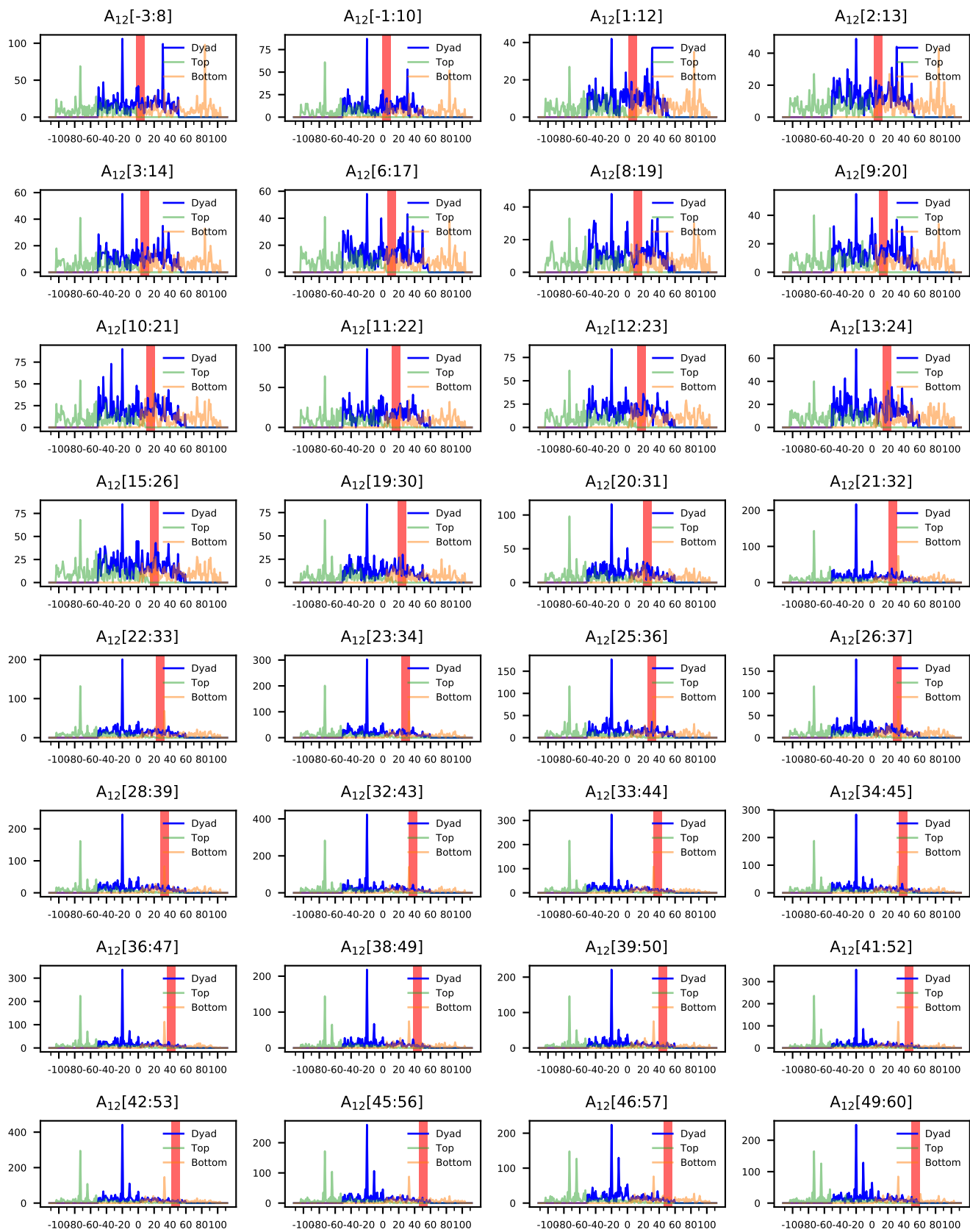

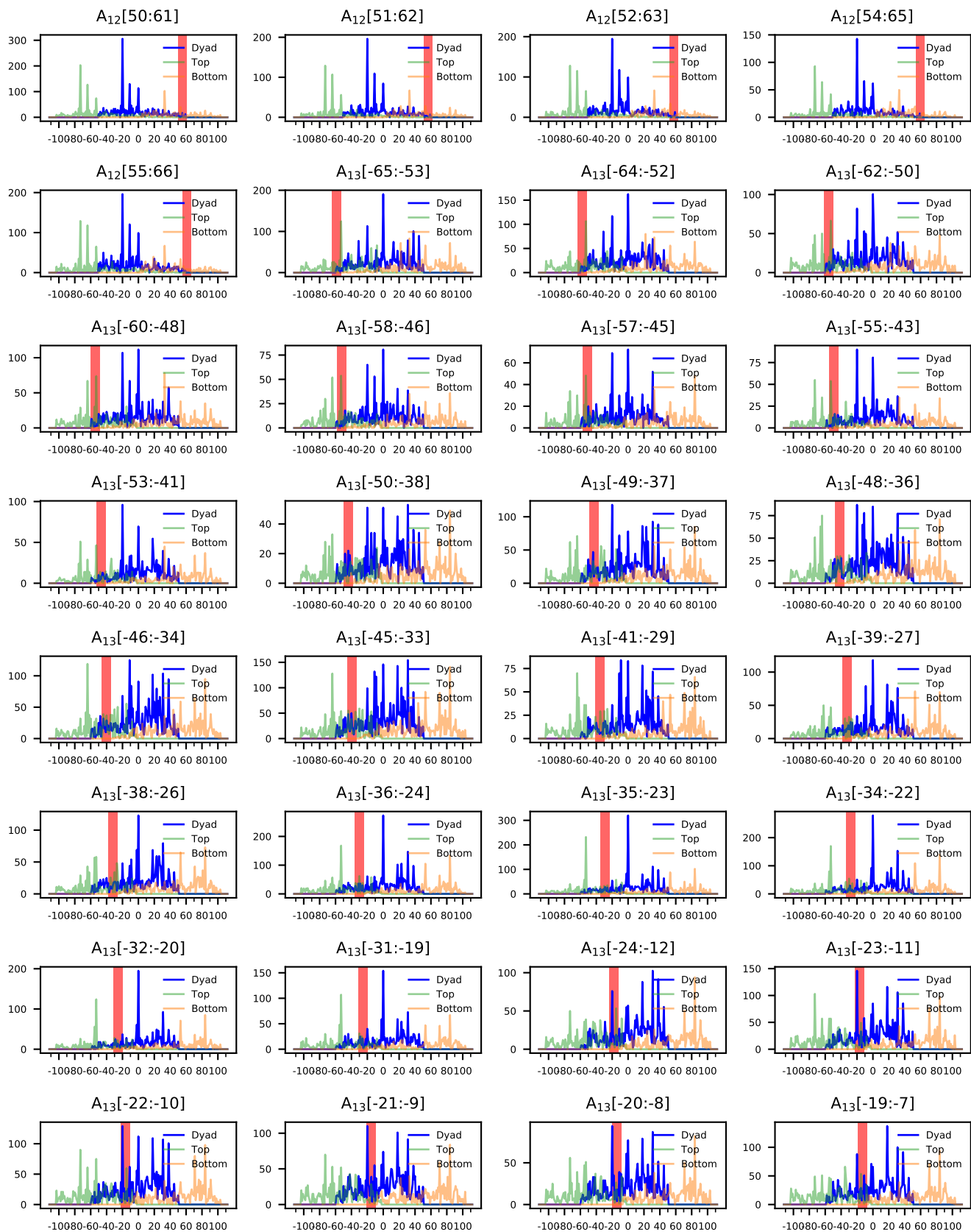

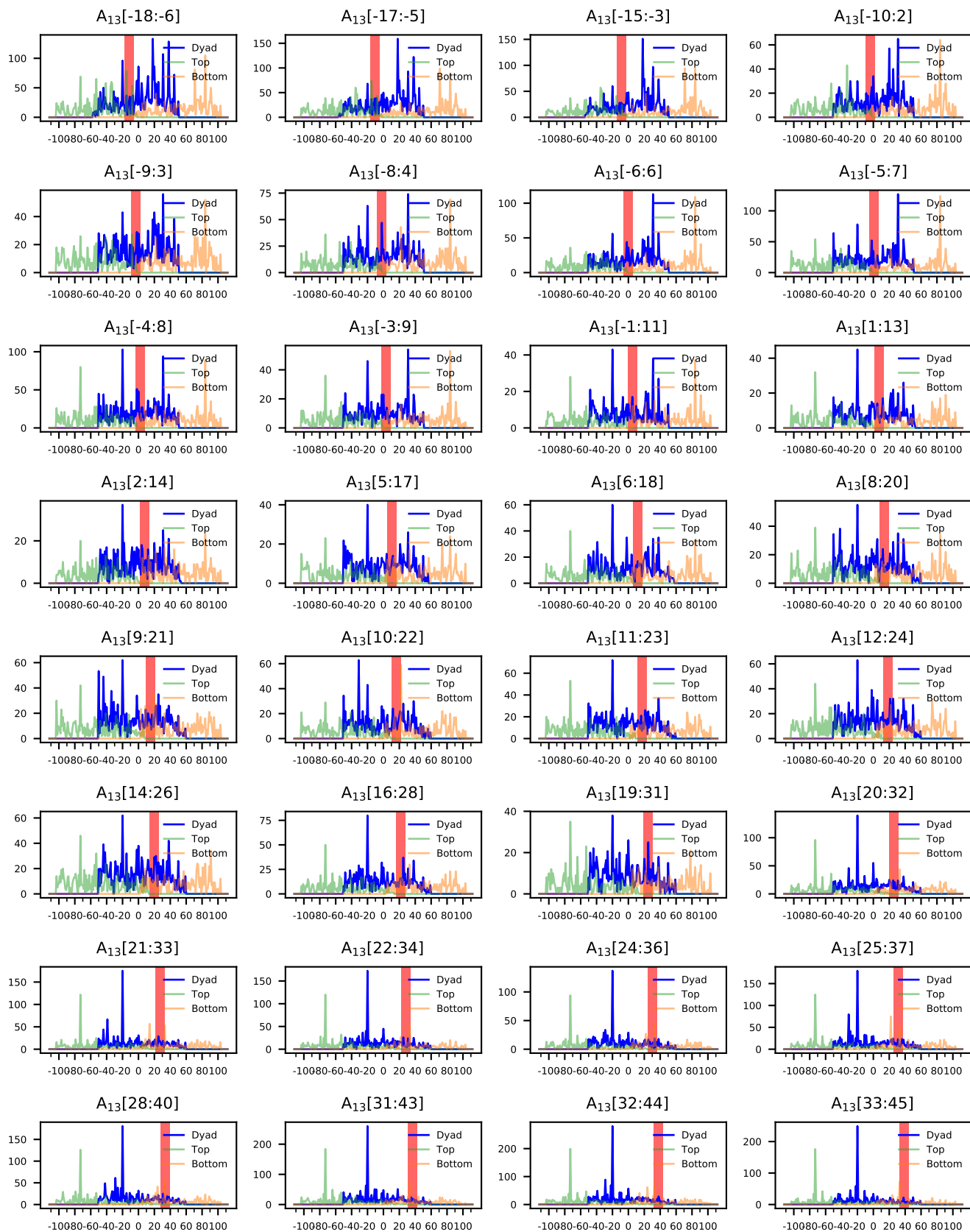

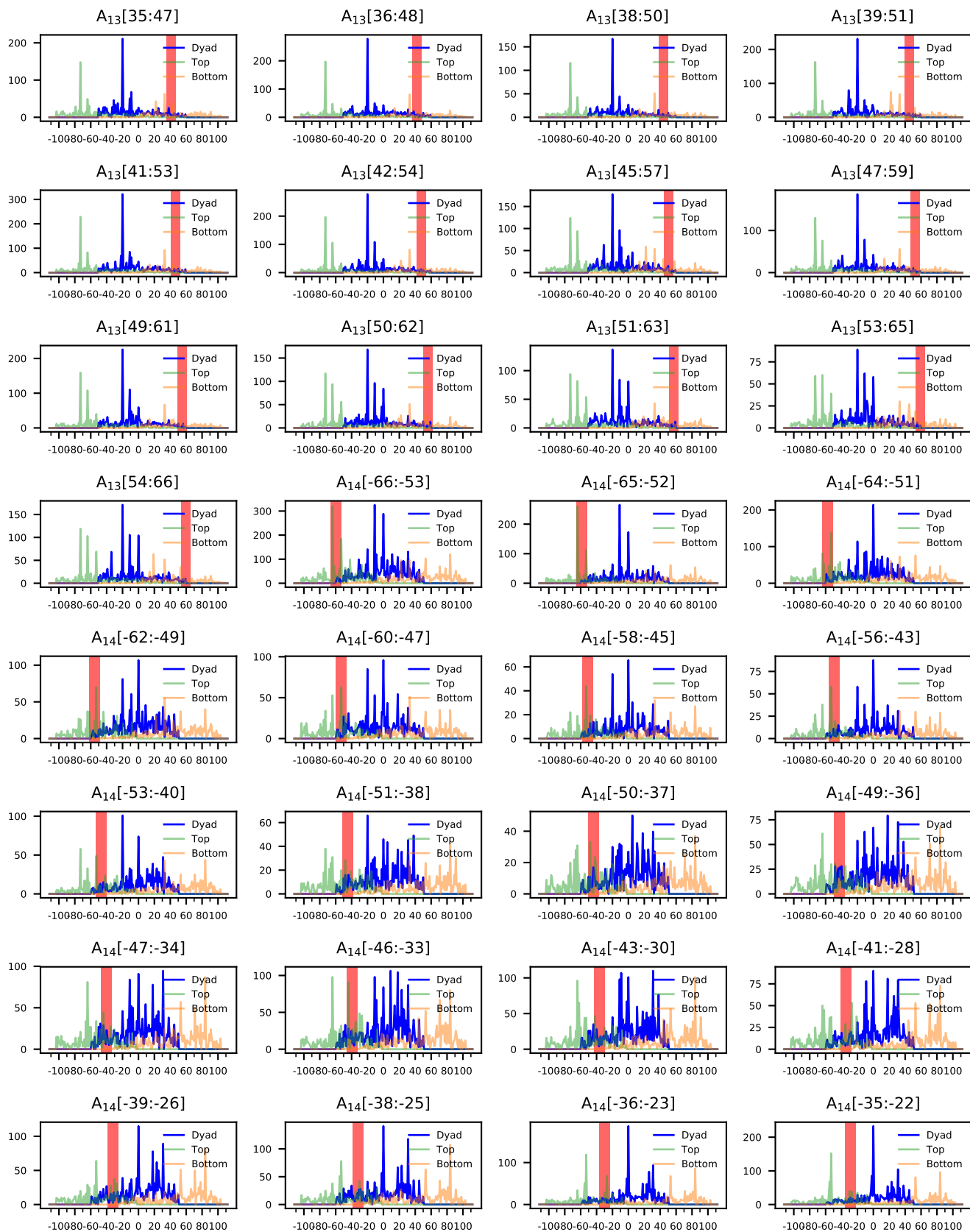

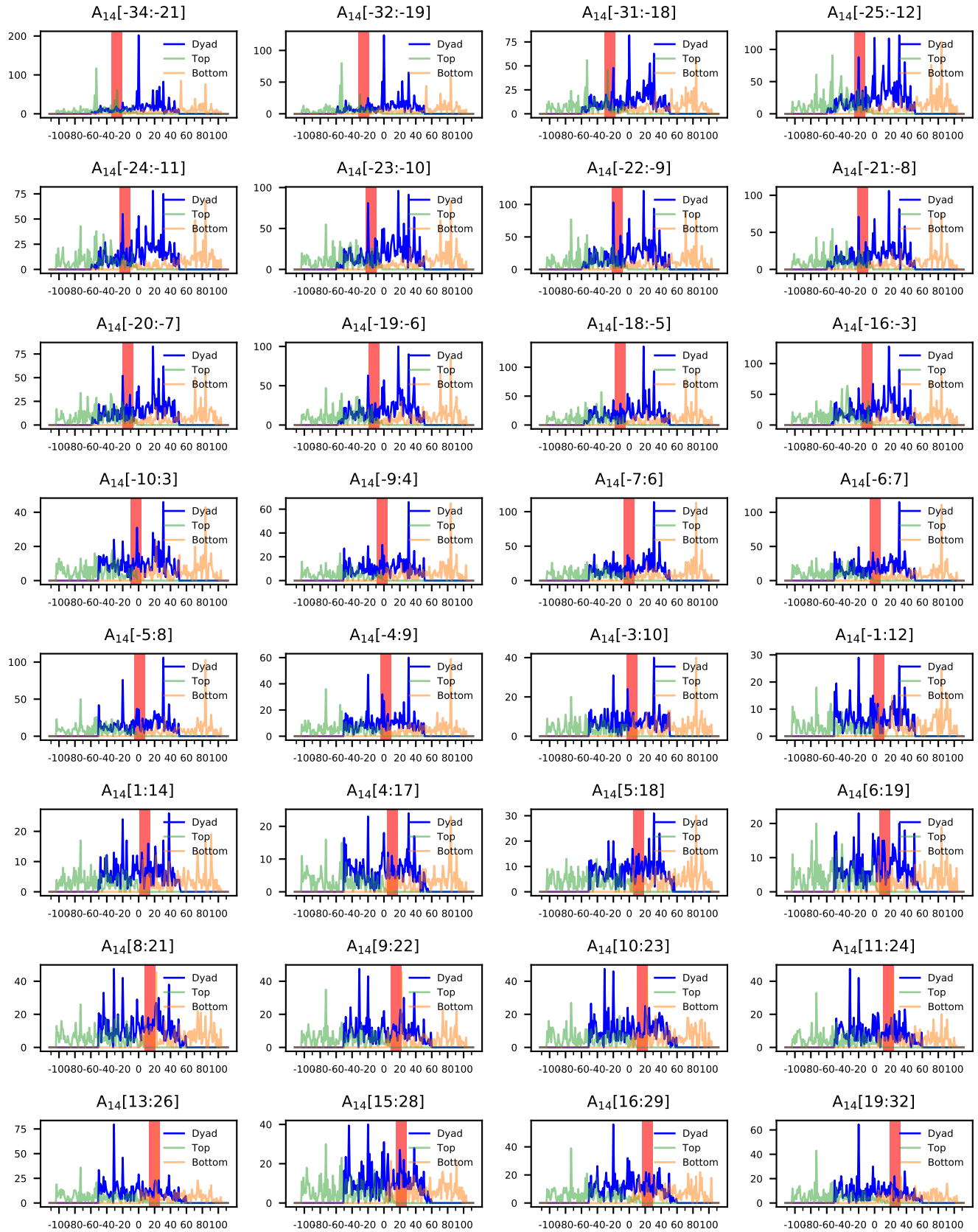

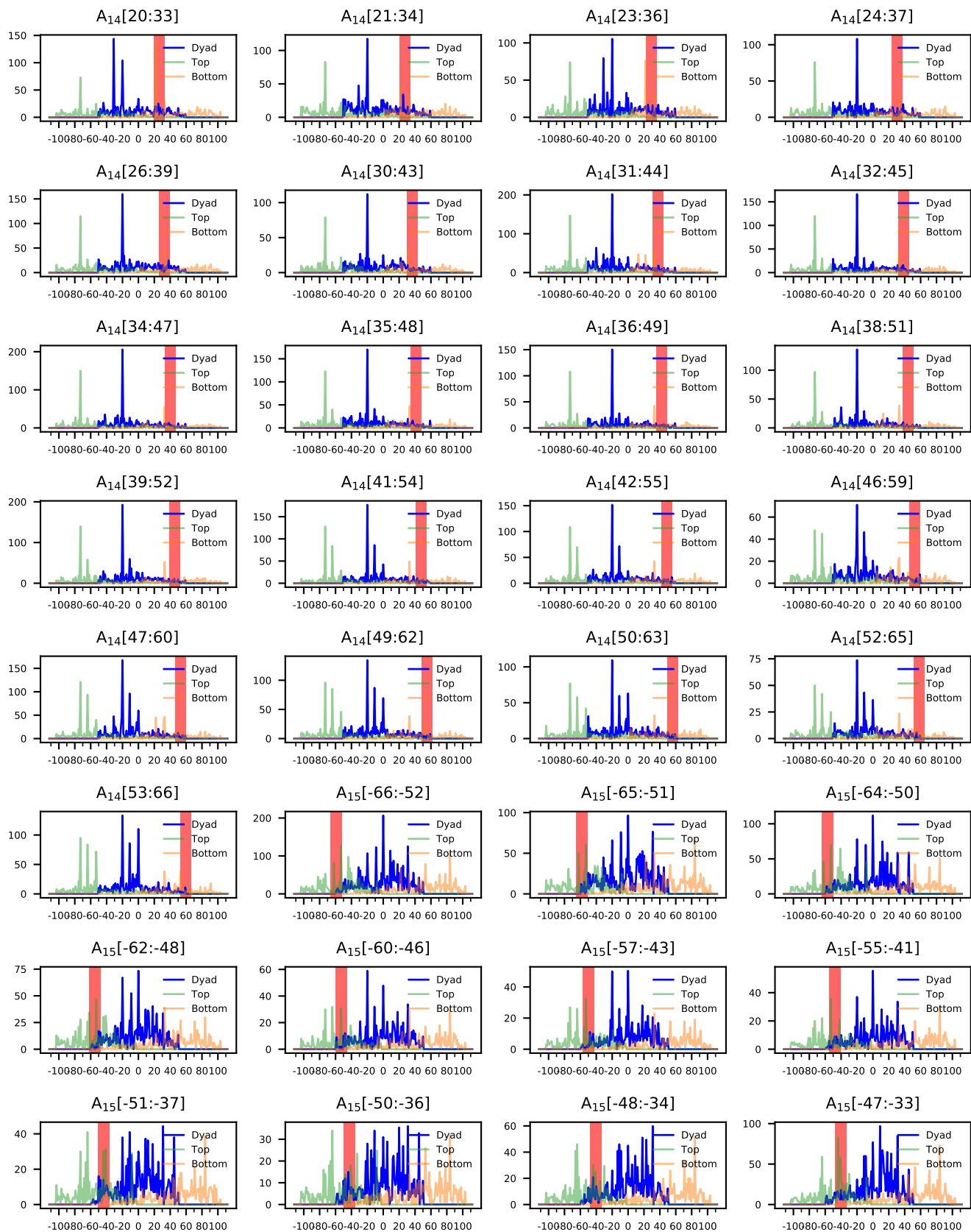

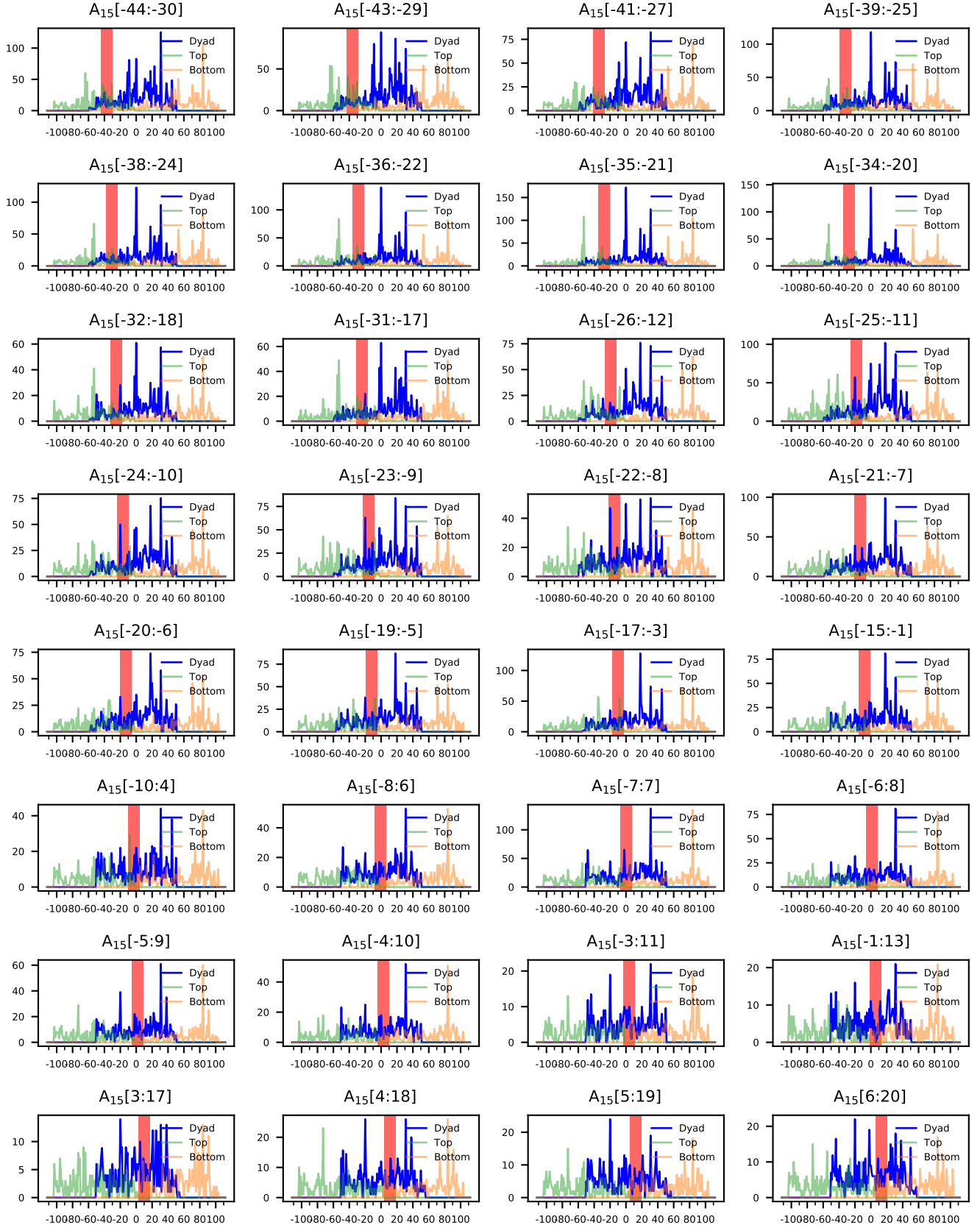

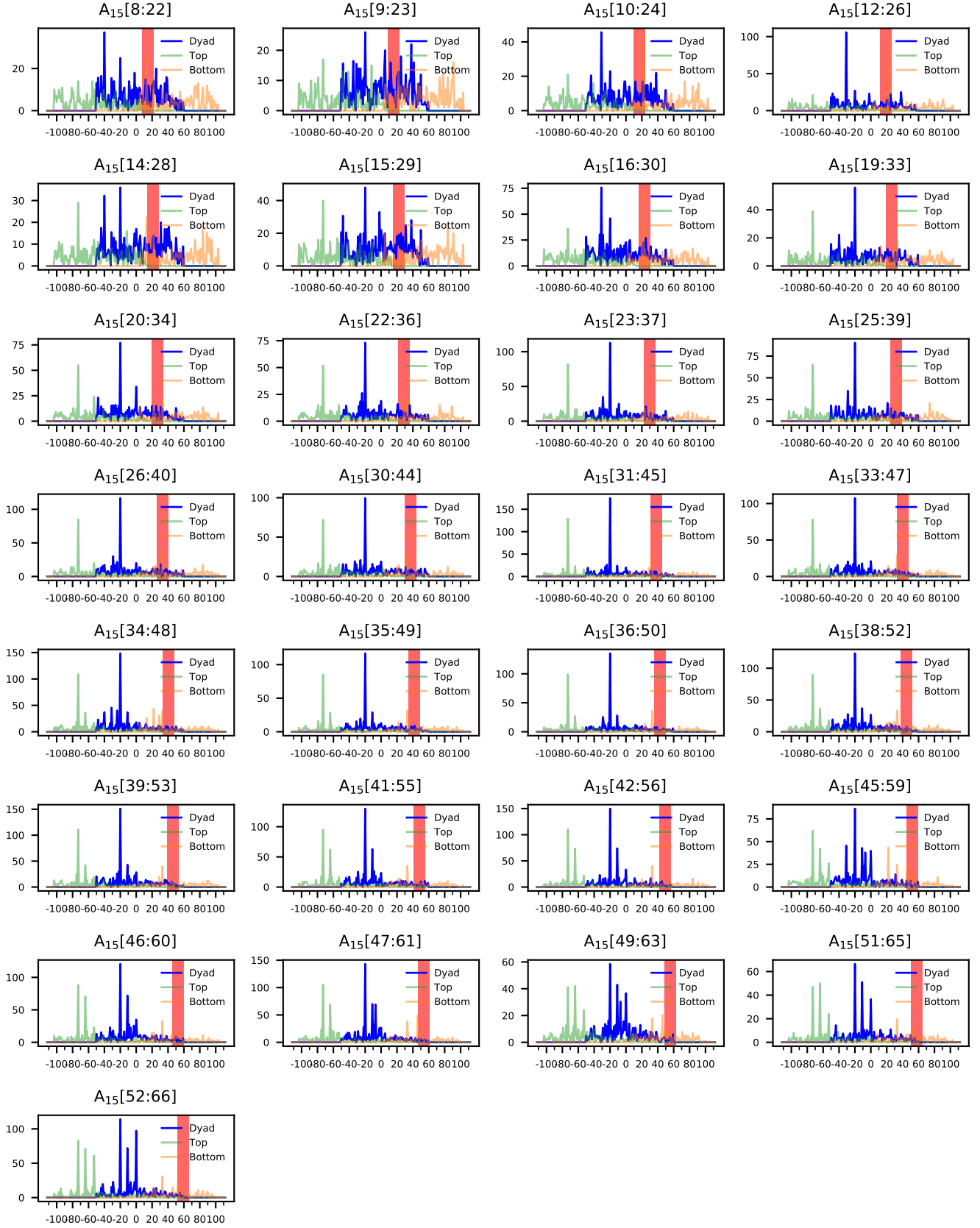

Supplement: gkad738_Supplemental_files [file gkad738_supplemental_files.zip › Supplementary Table 3 (601-Signals_A_after).pdf]

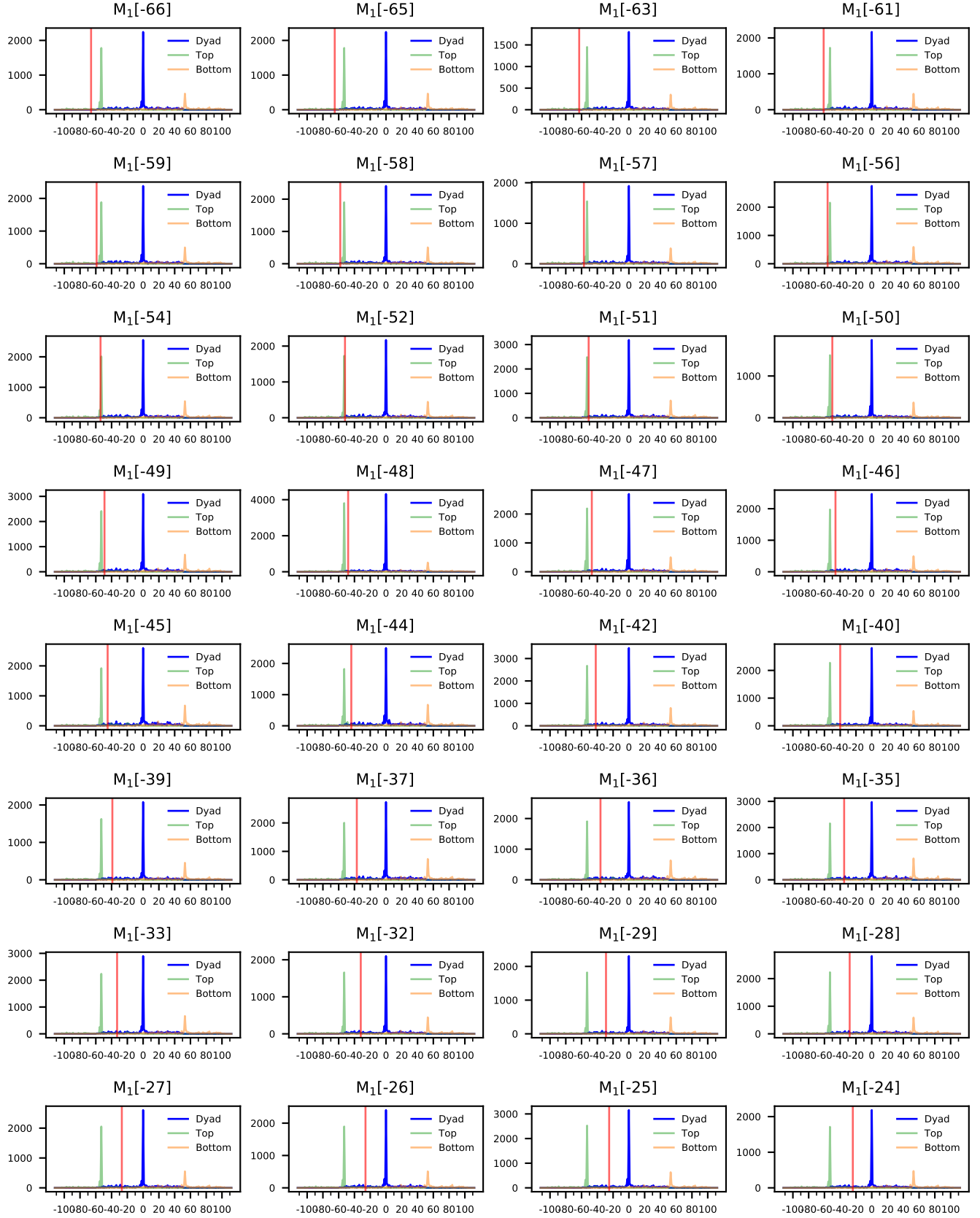

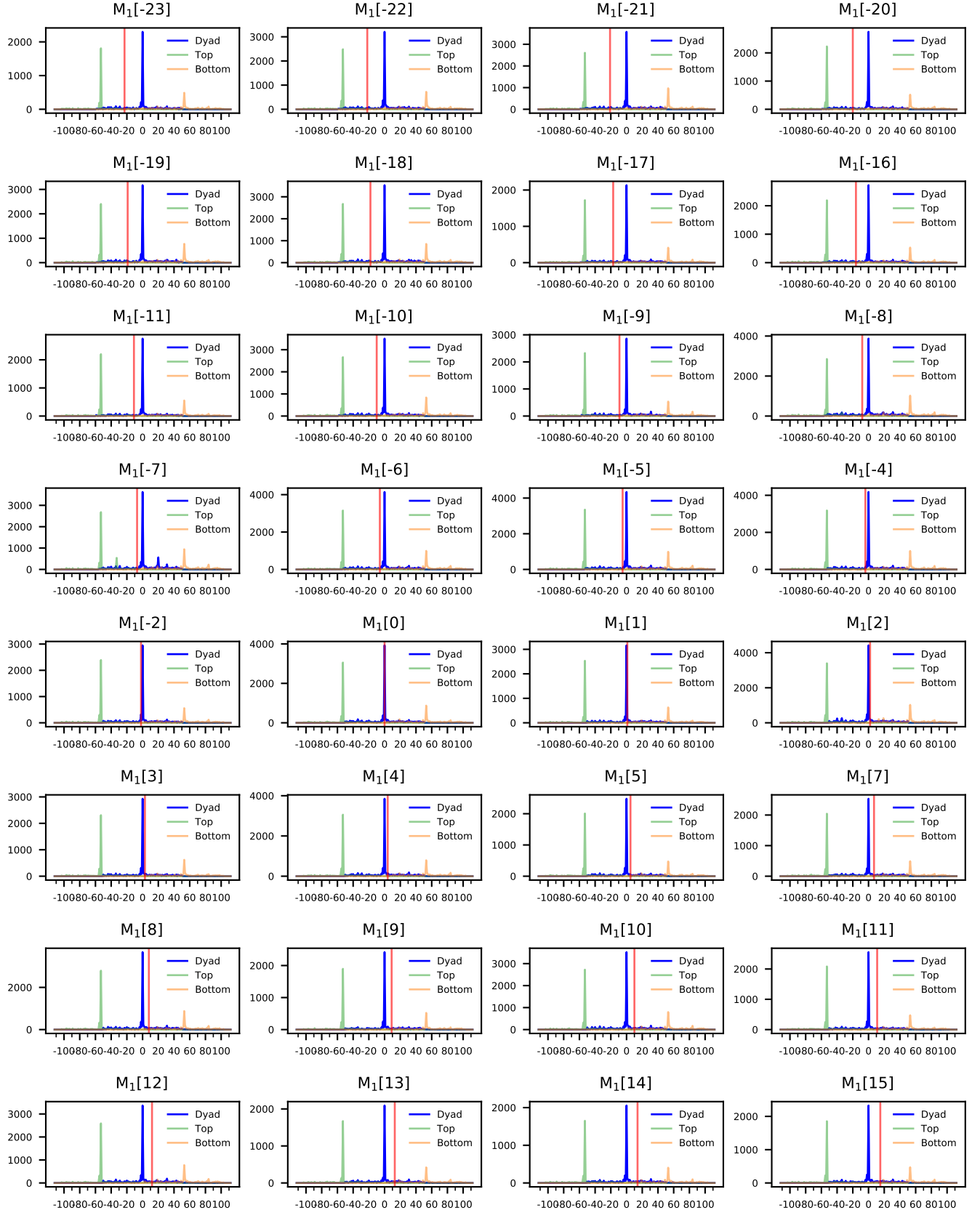

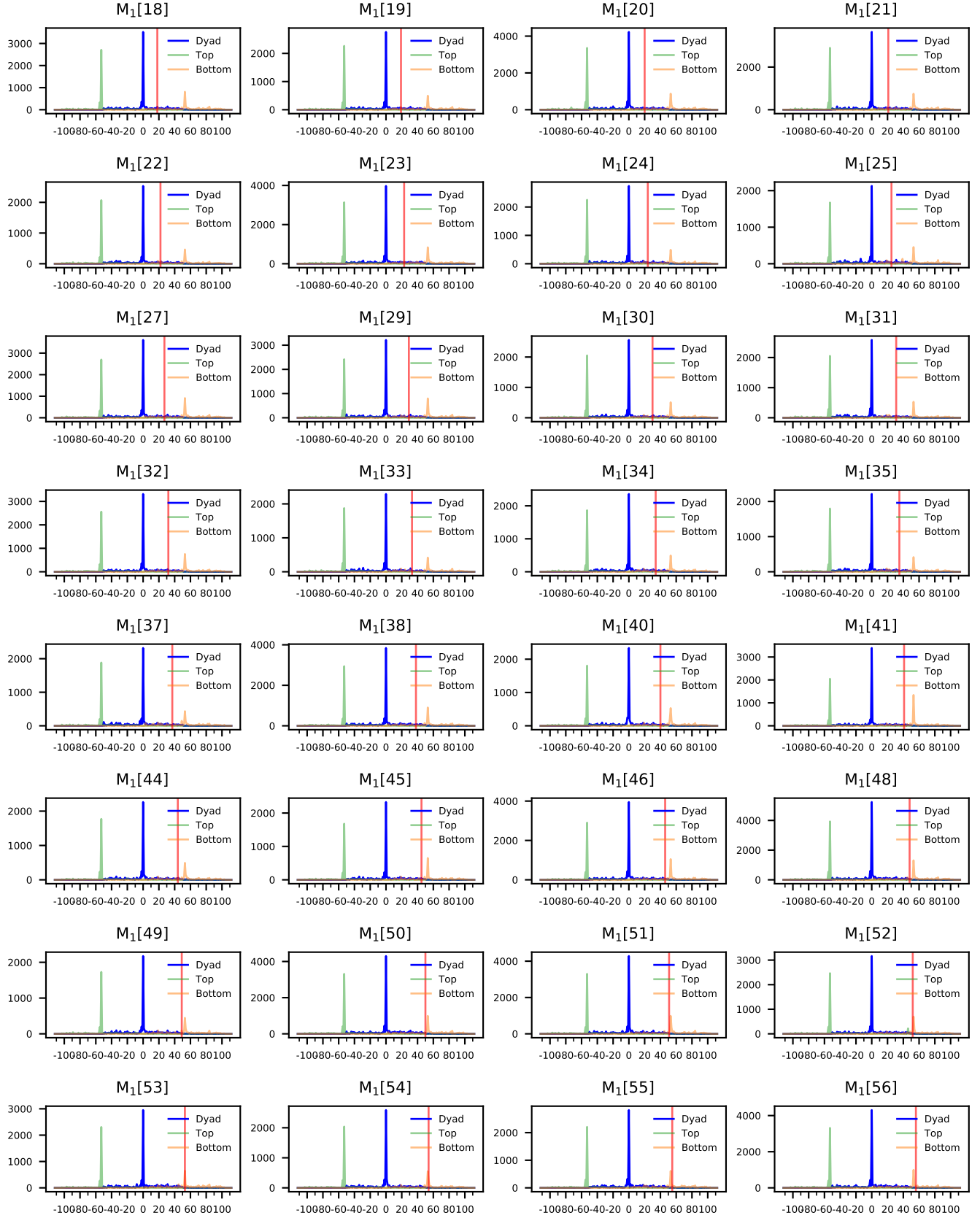

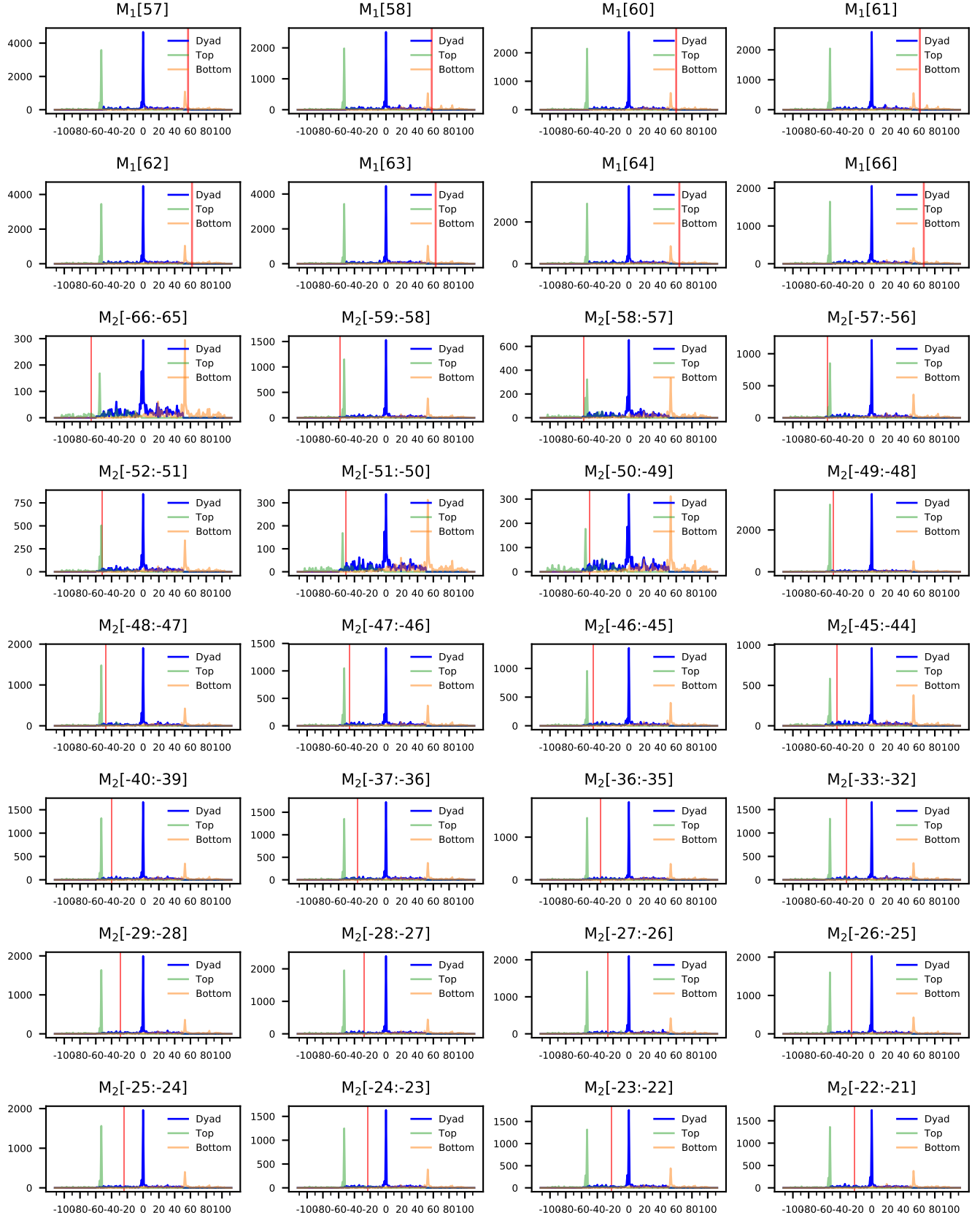

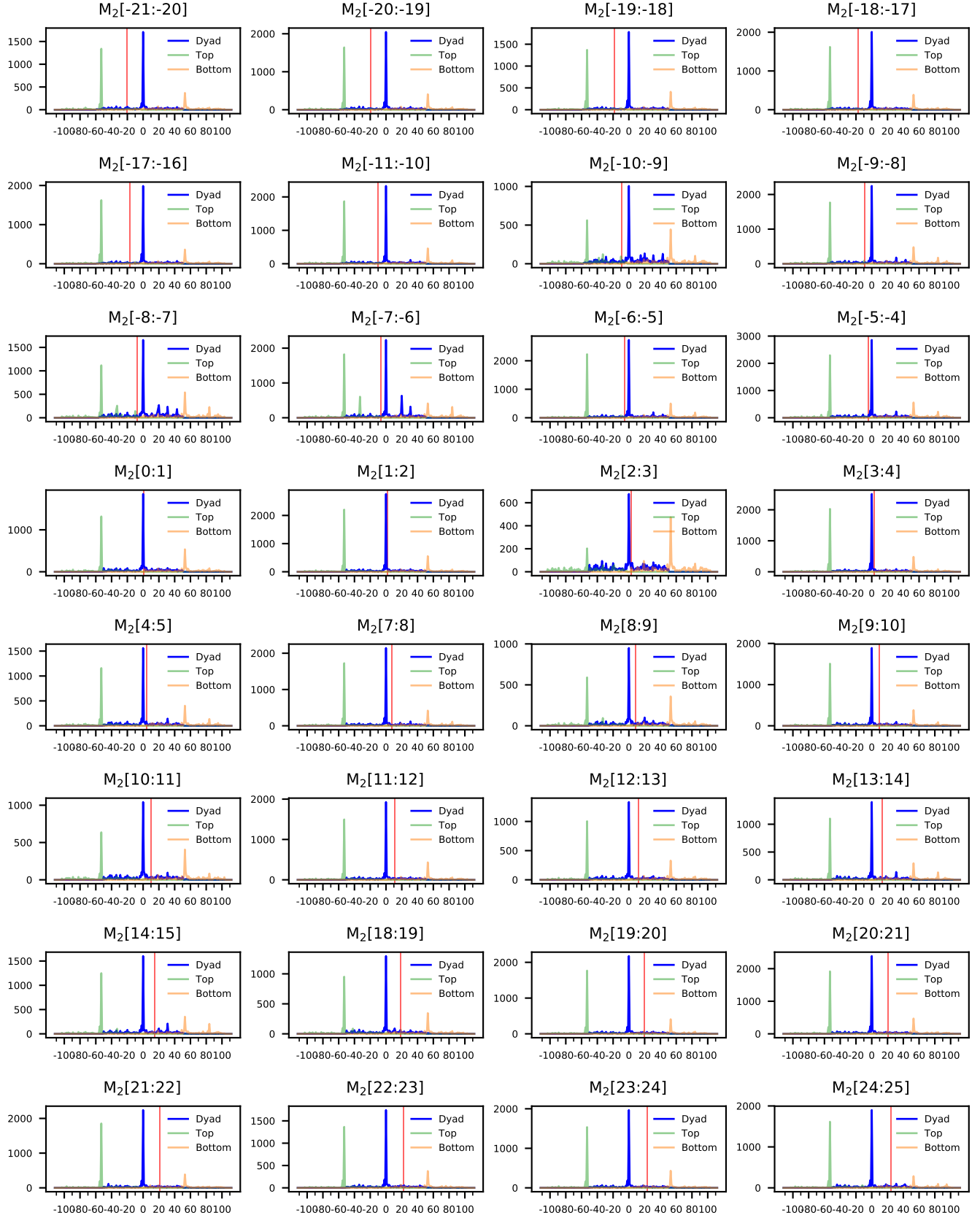

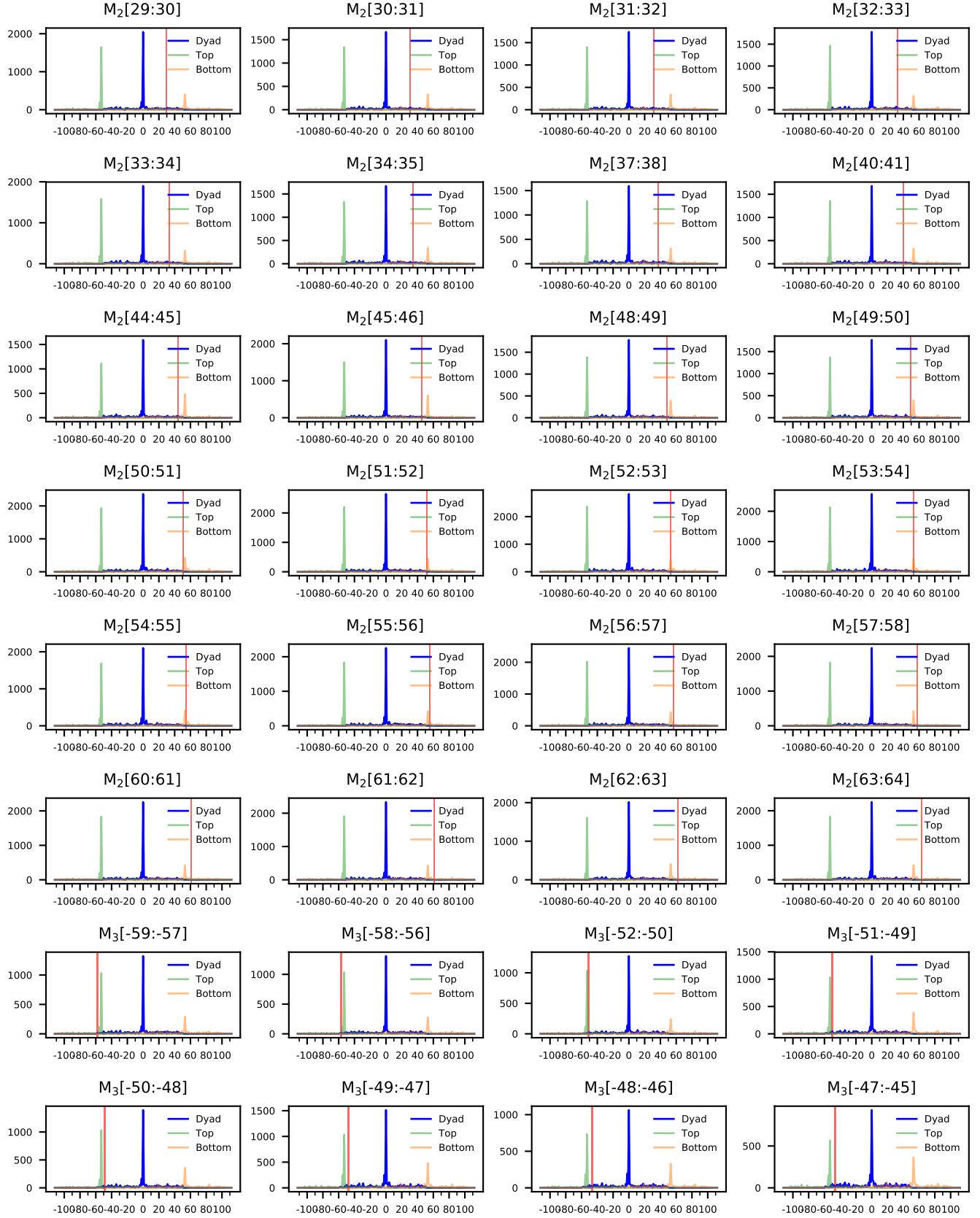

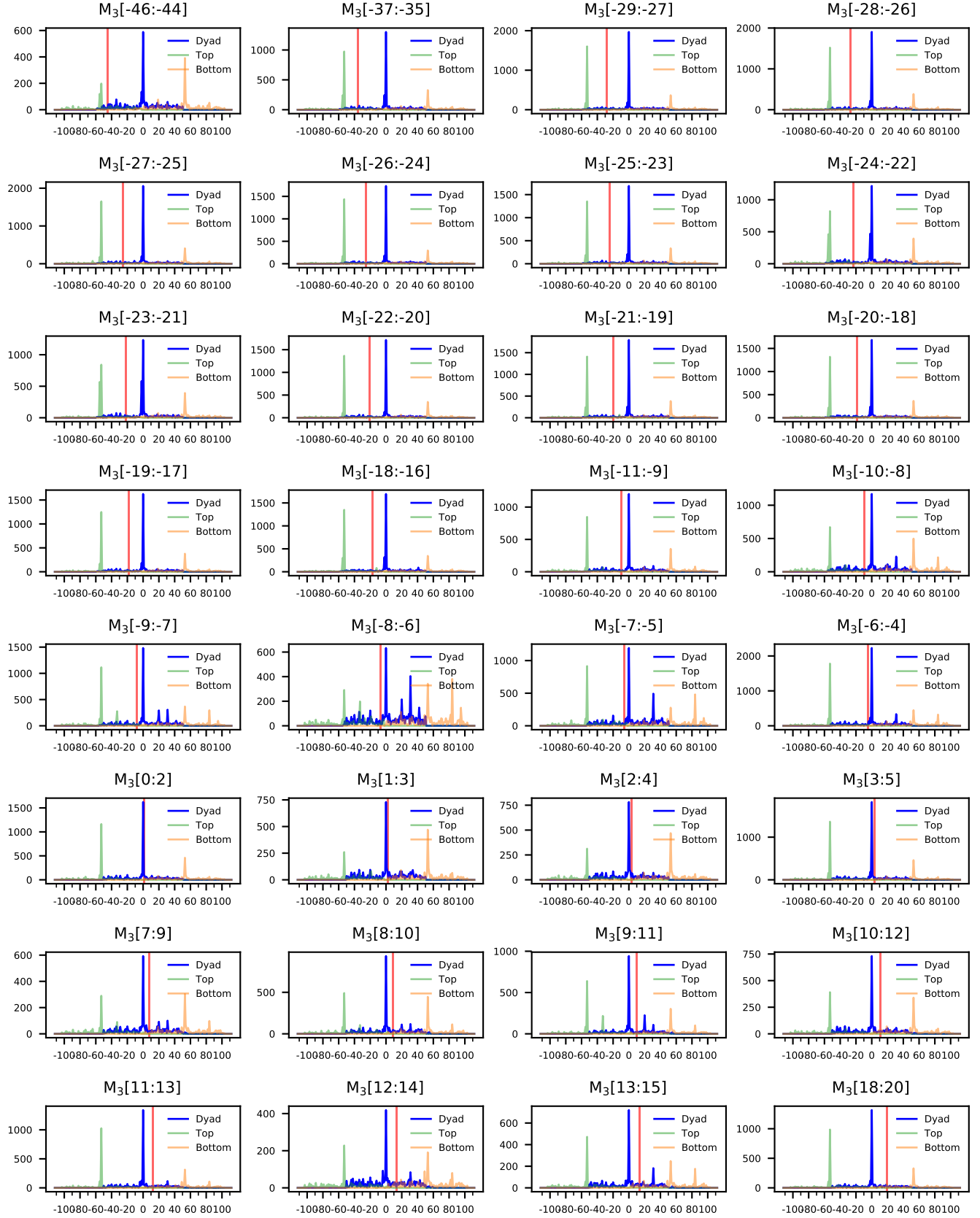

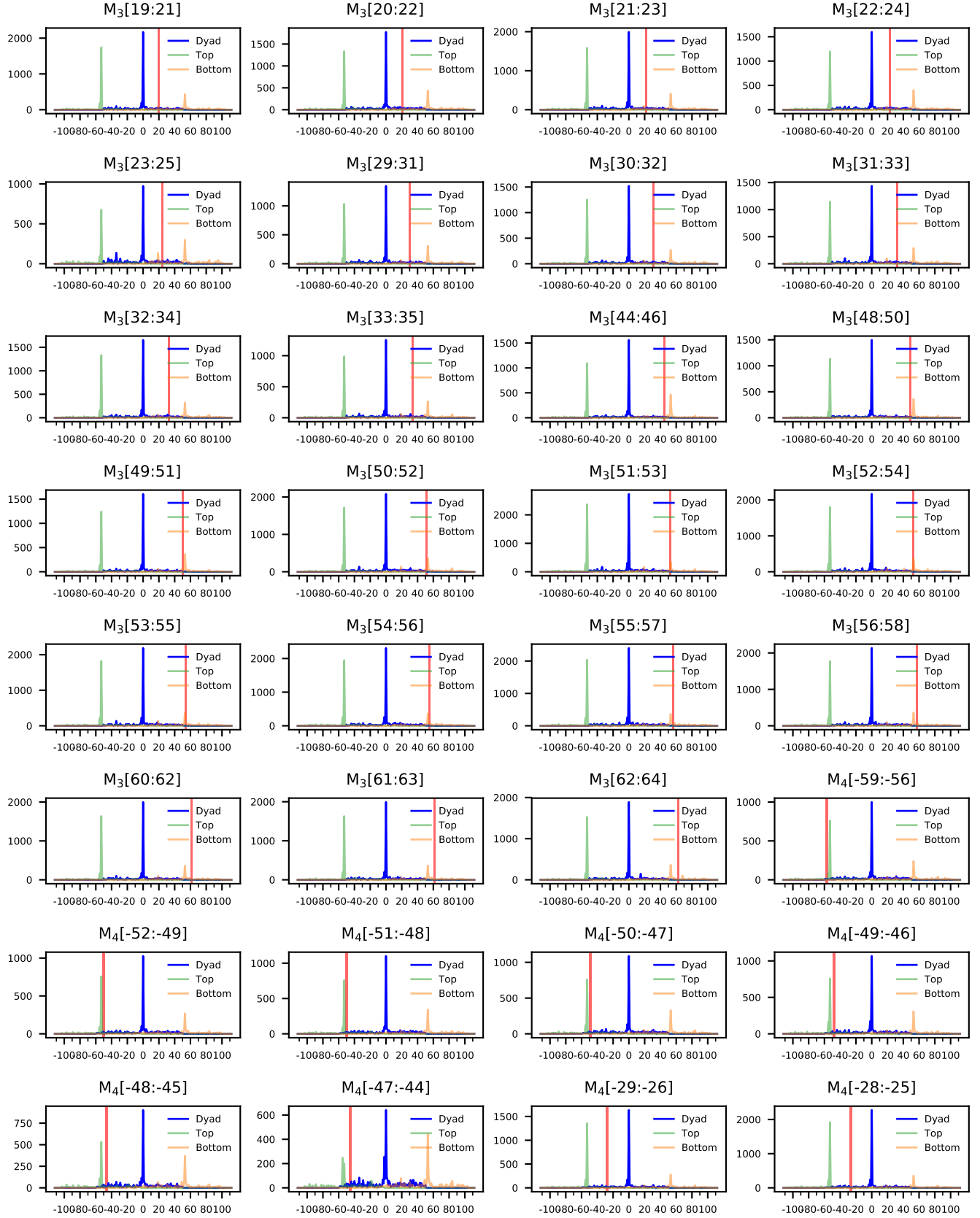

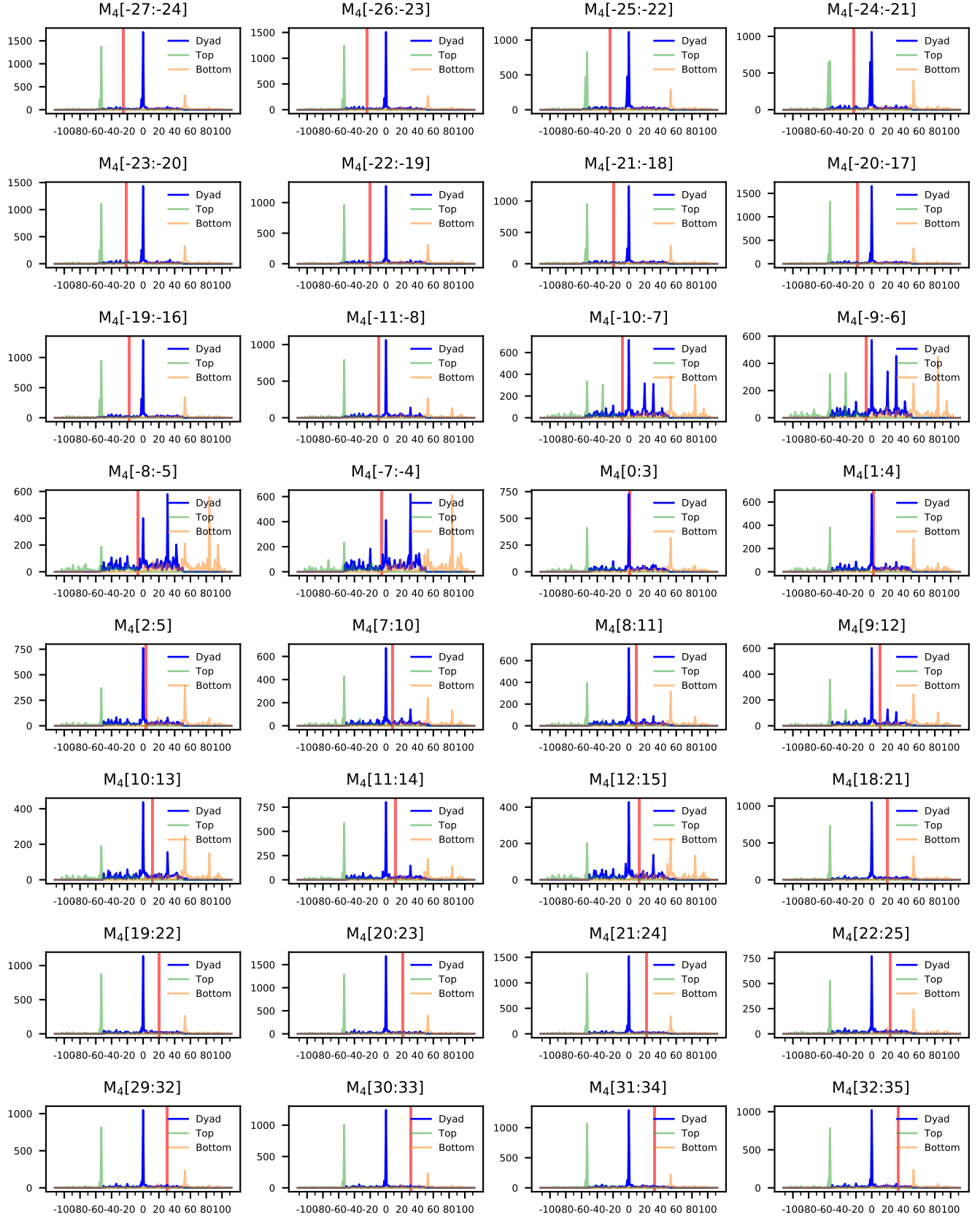

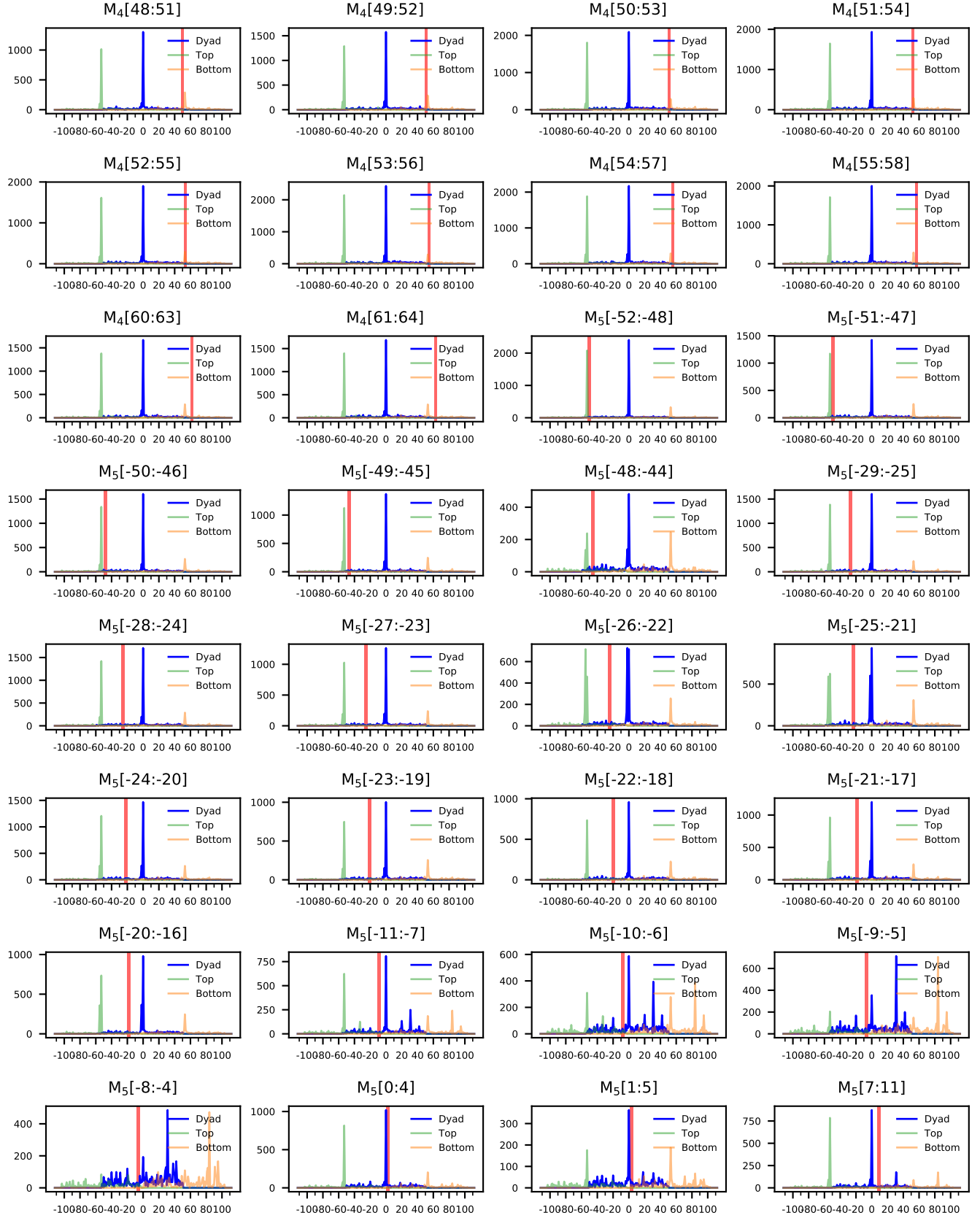

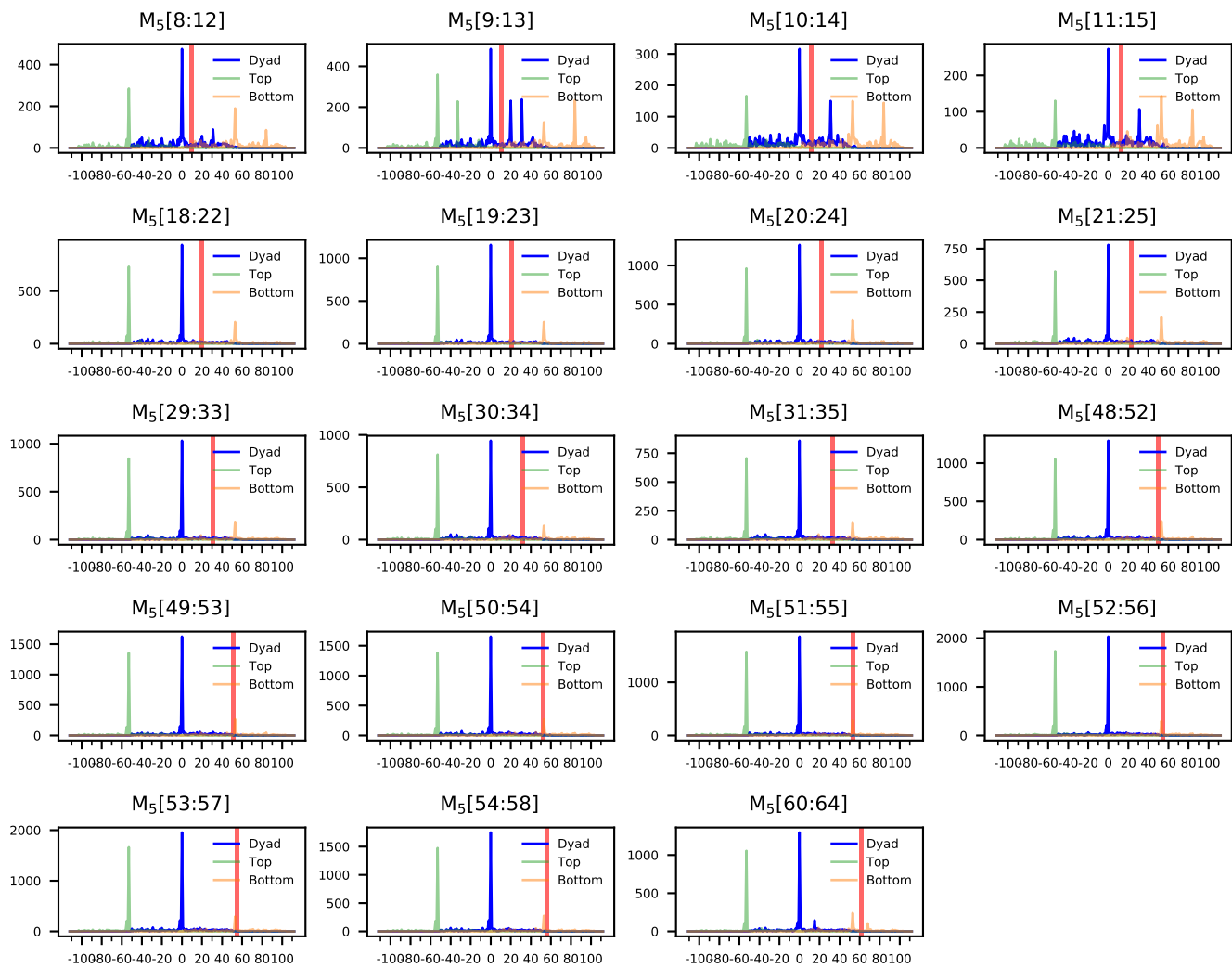

Supplement: gkad738_Supplemental_files [file gkad738_supplemental_files.zip › Supplementary Table 4 (601-Signals_M_before).pdf]

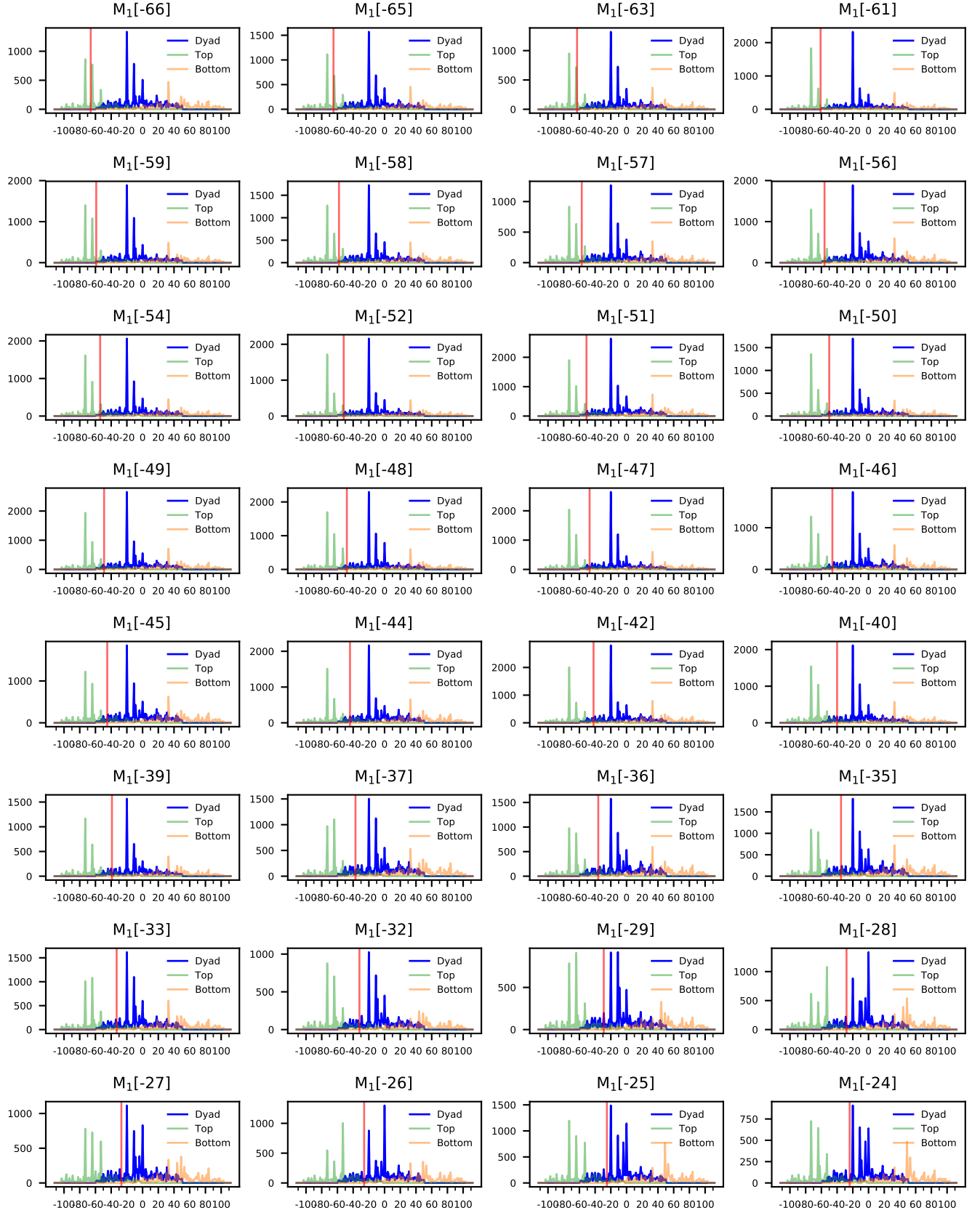

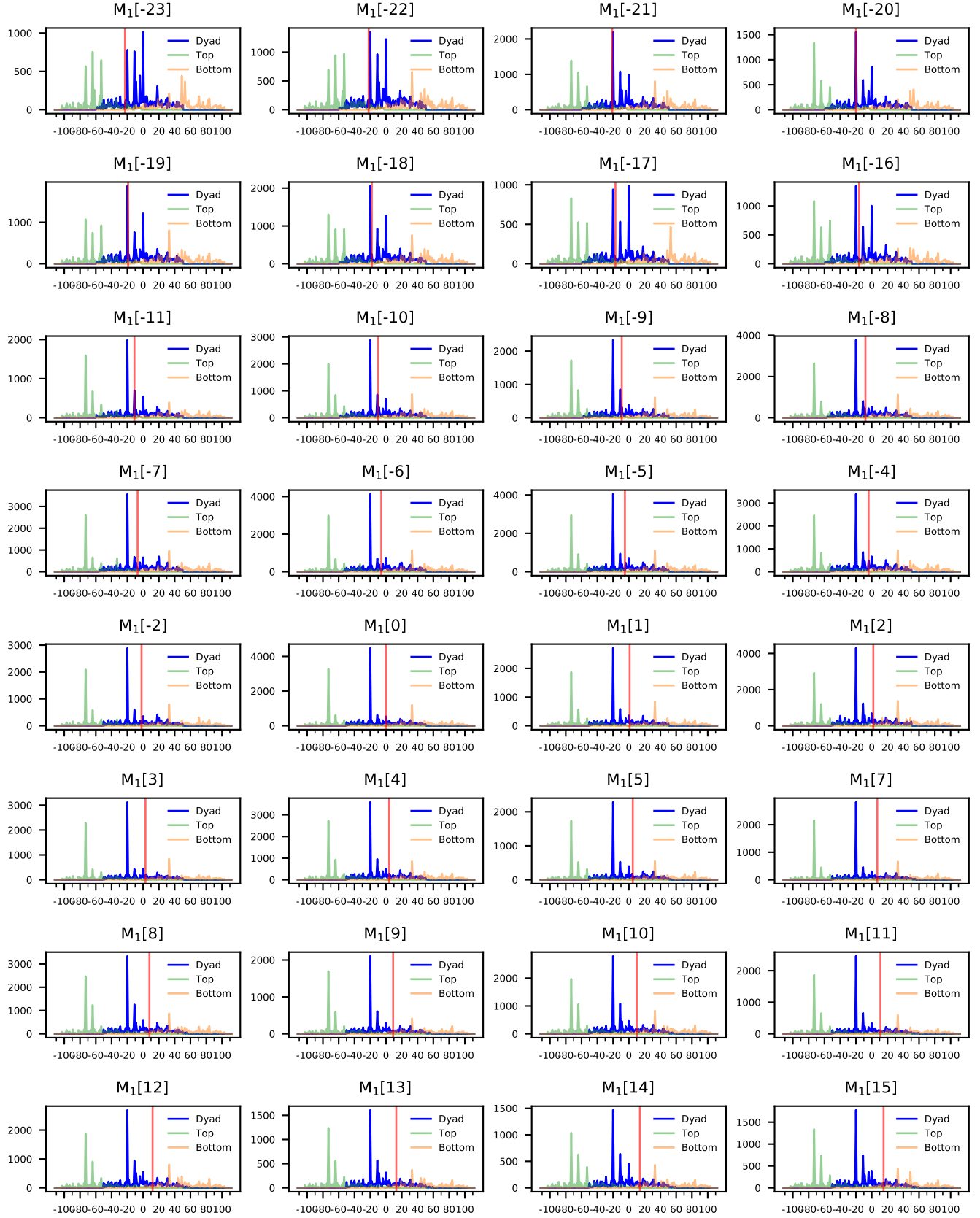

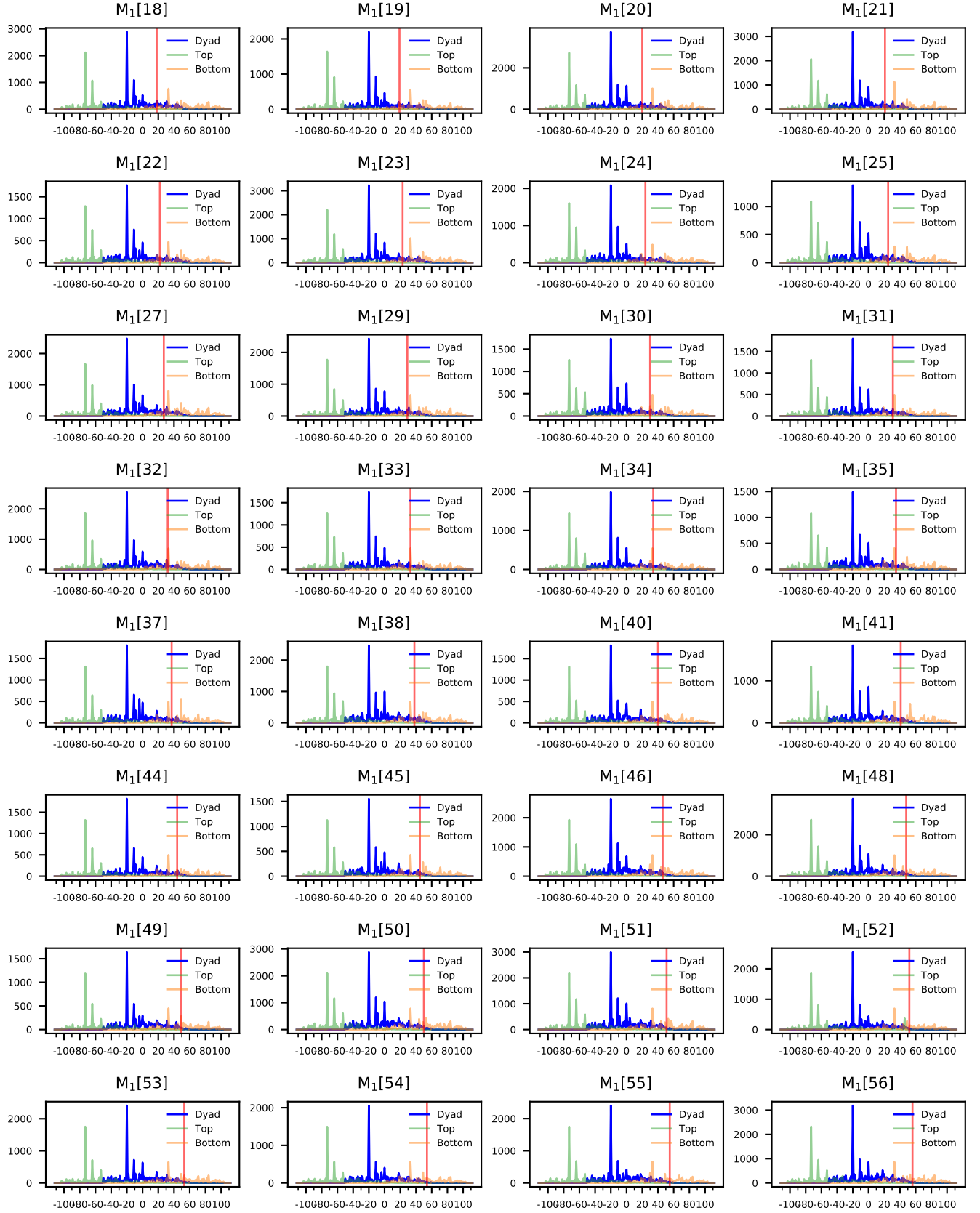

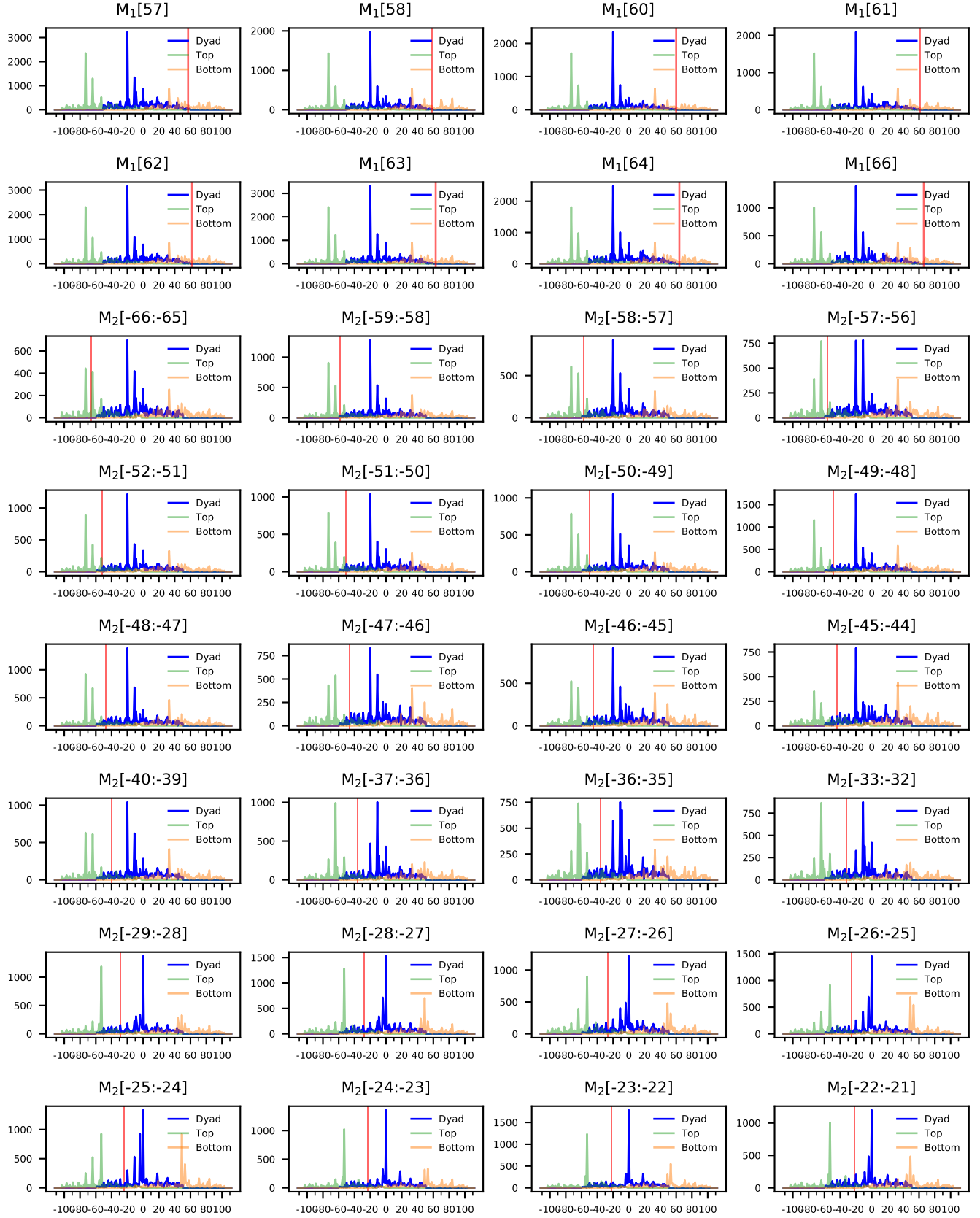

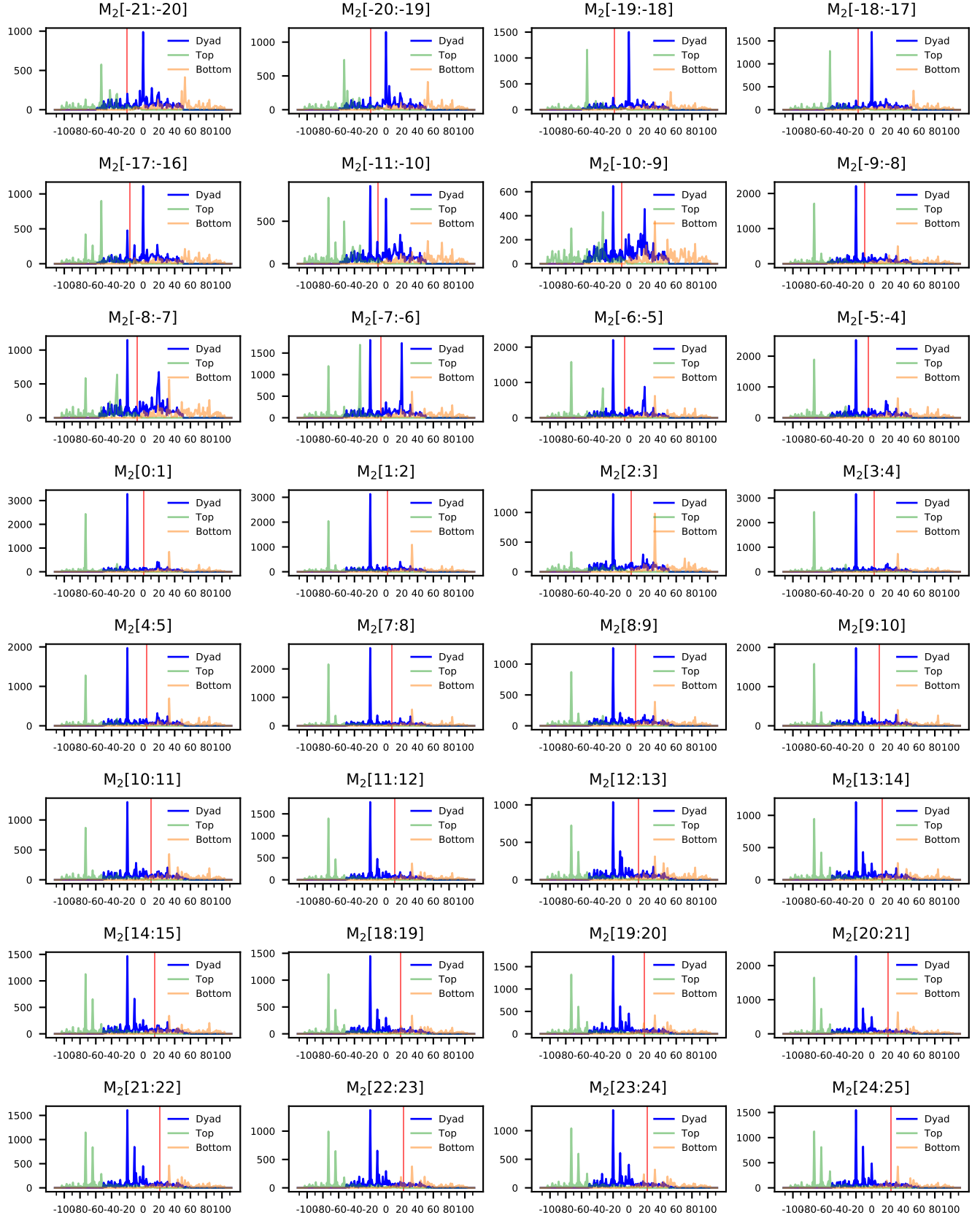

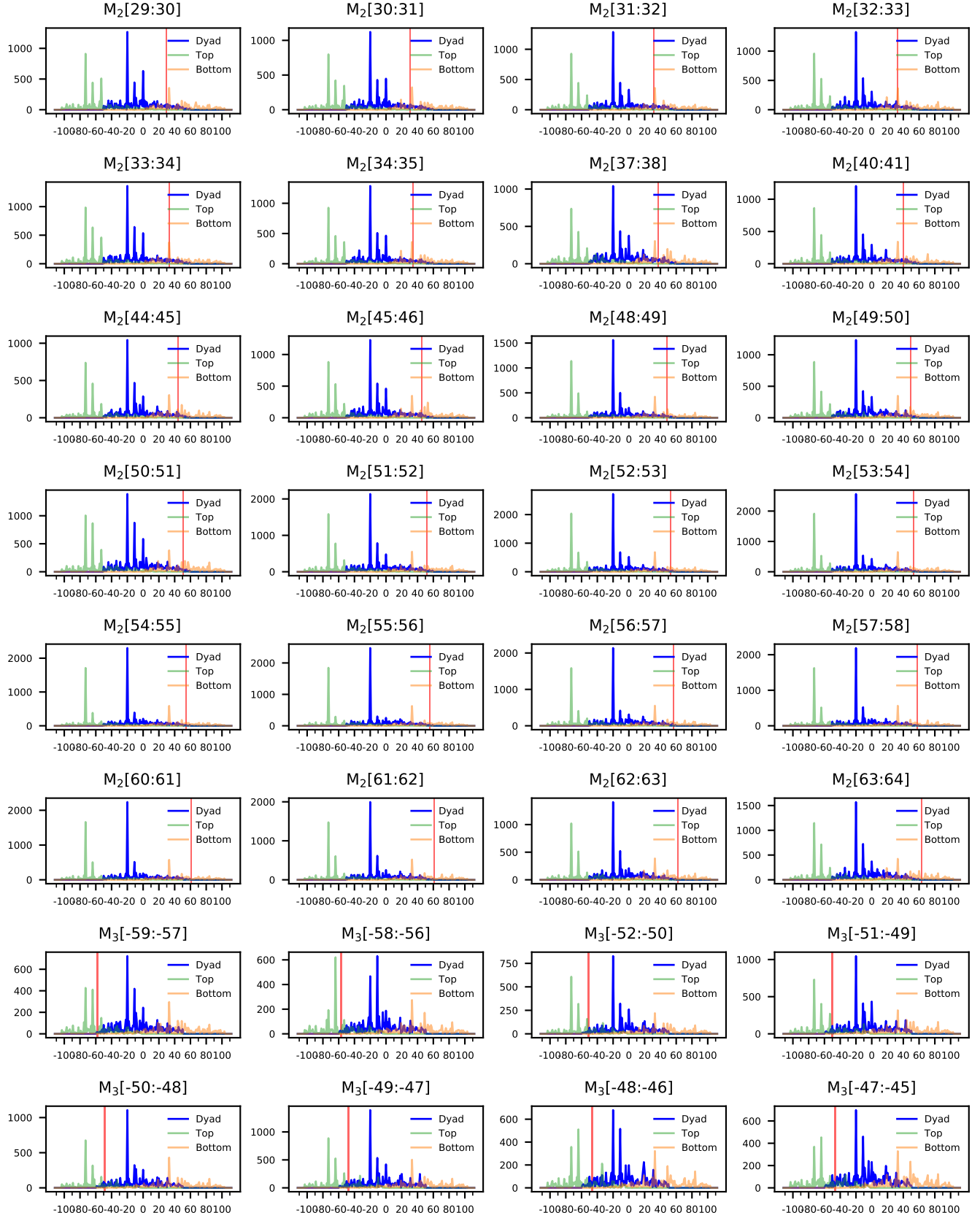

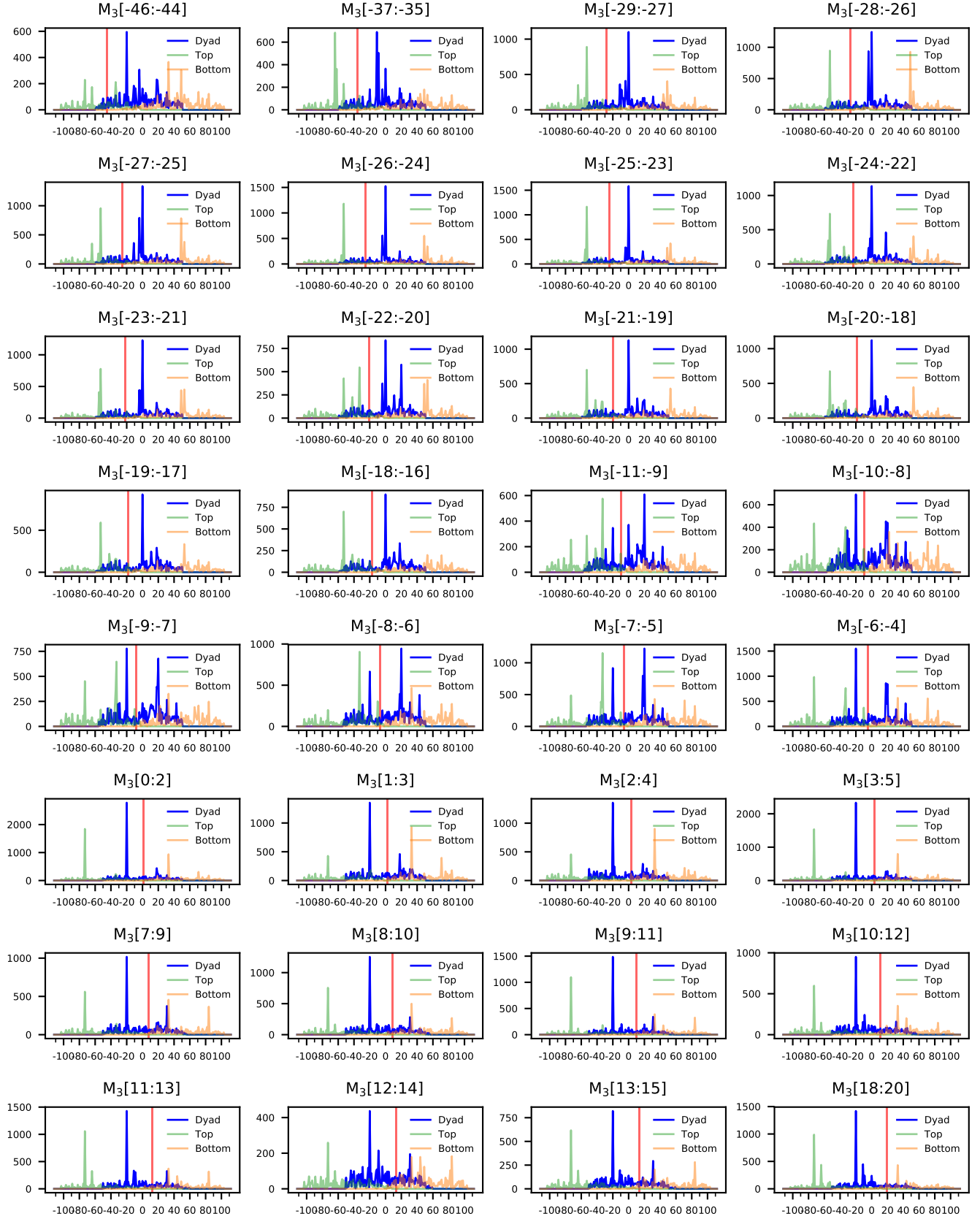

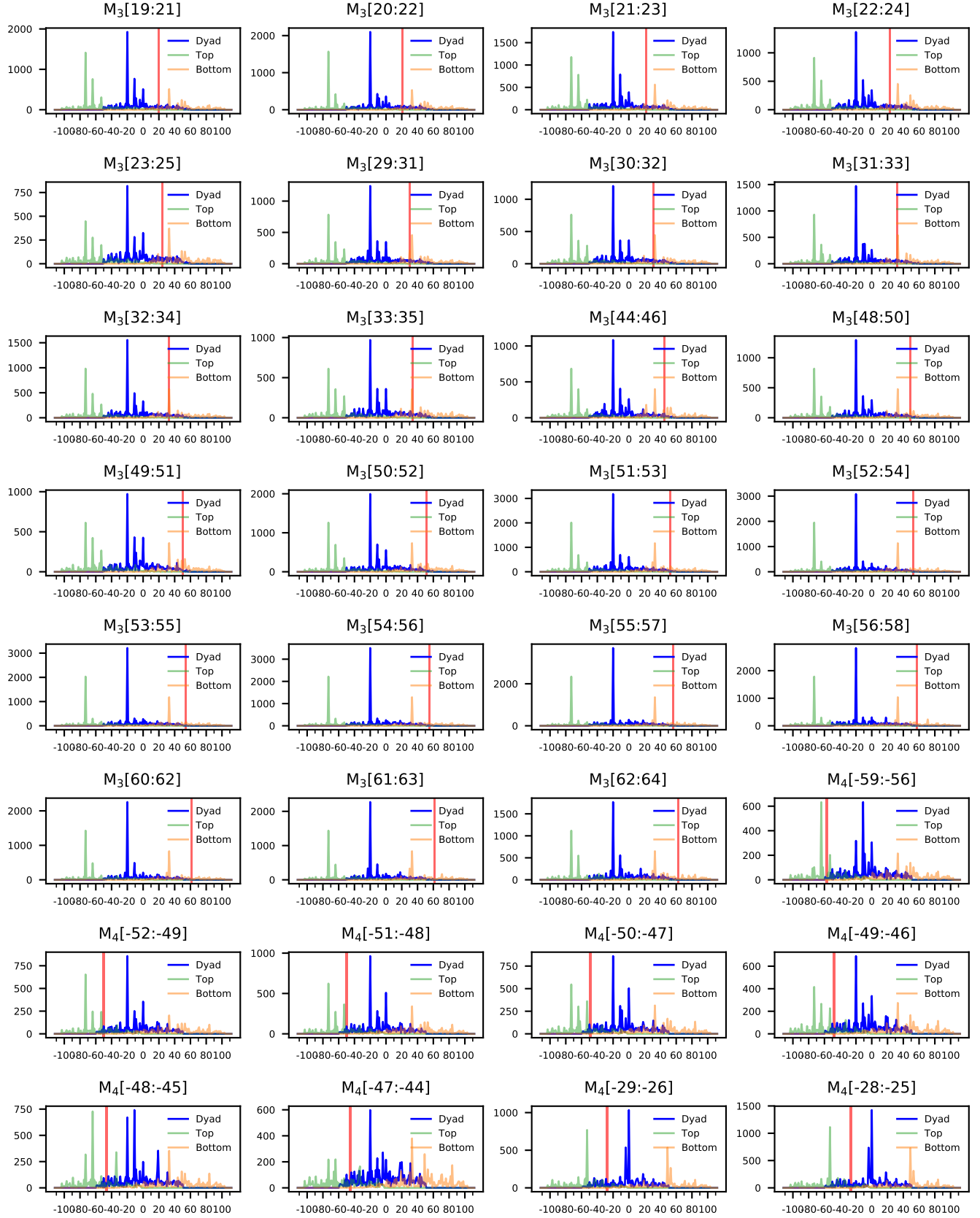

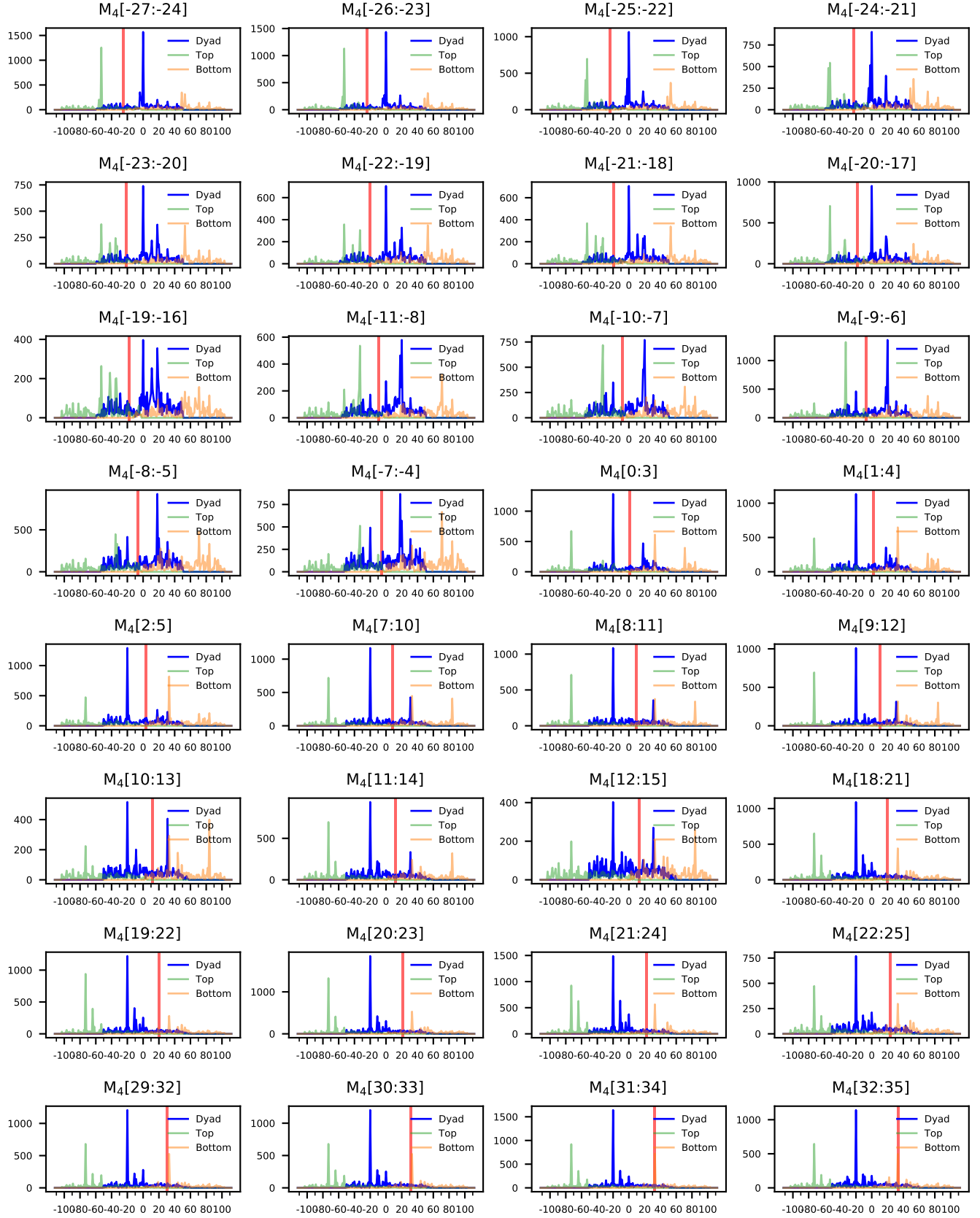

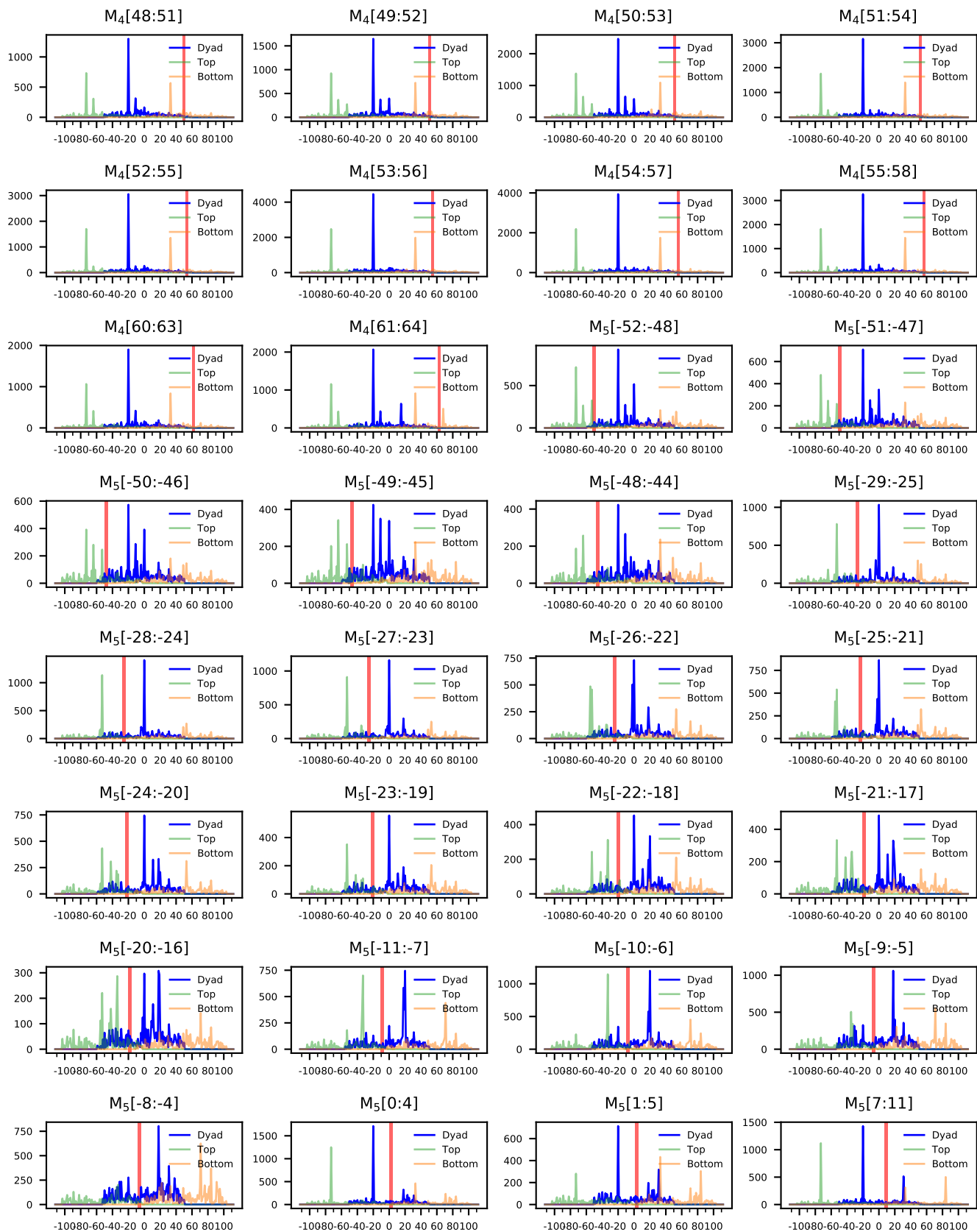

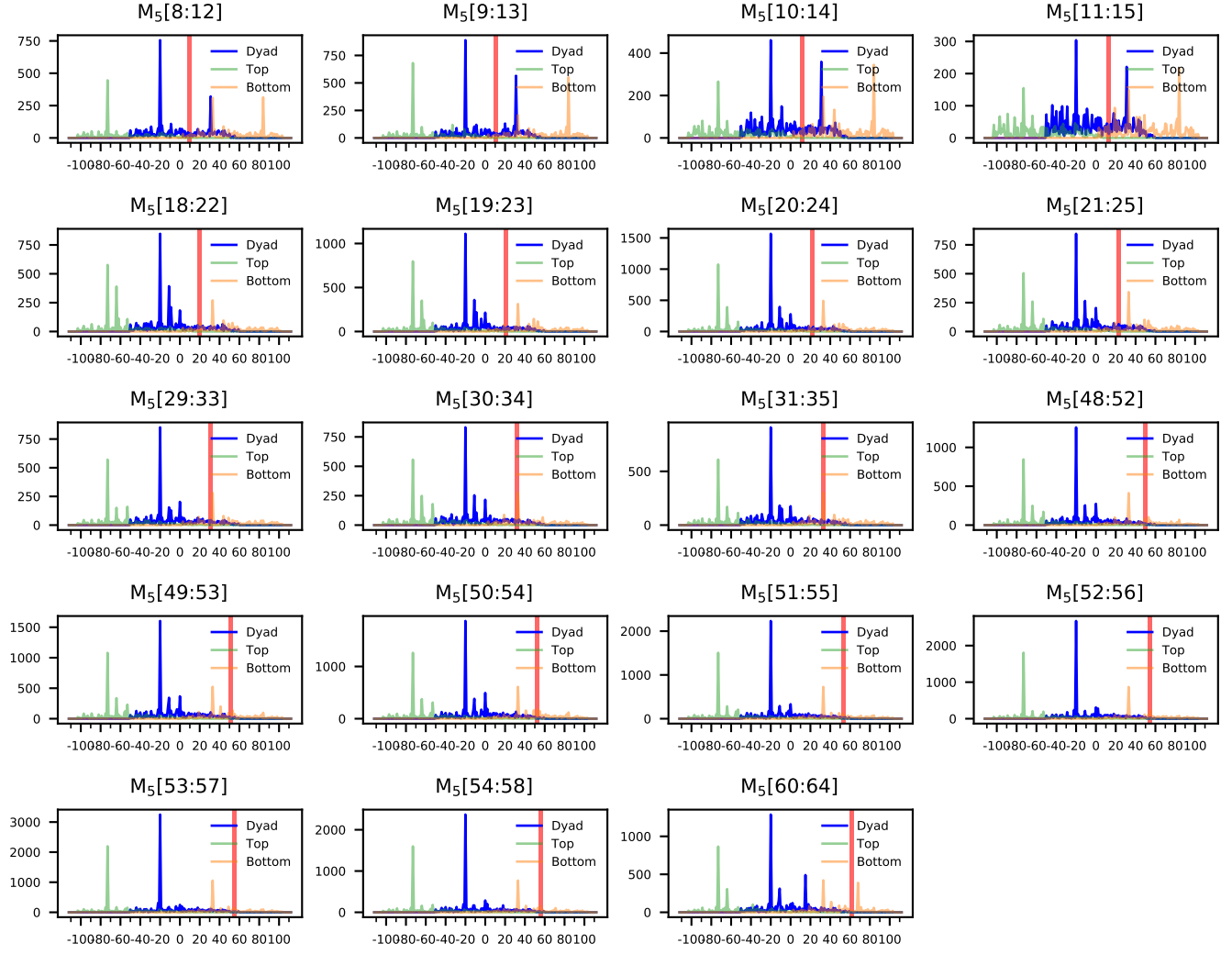

Supplement: gkad738_Supplemental_files [file gkad738_supplemental_files.zip › Supplementary Table 5 (601-Signals_M_after).pdf]

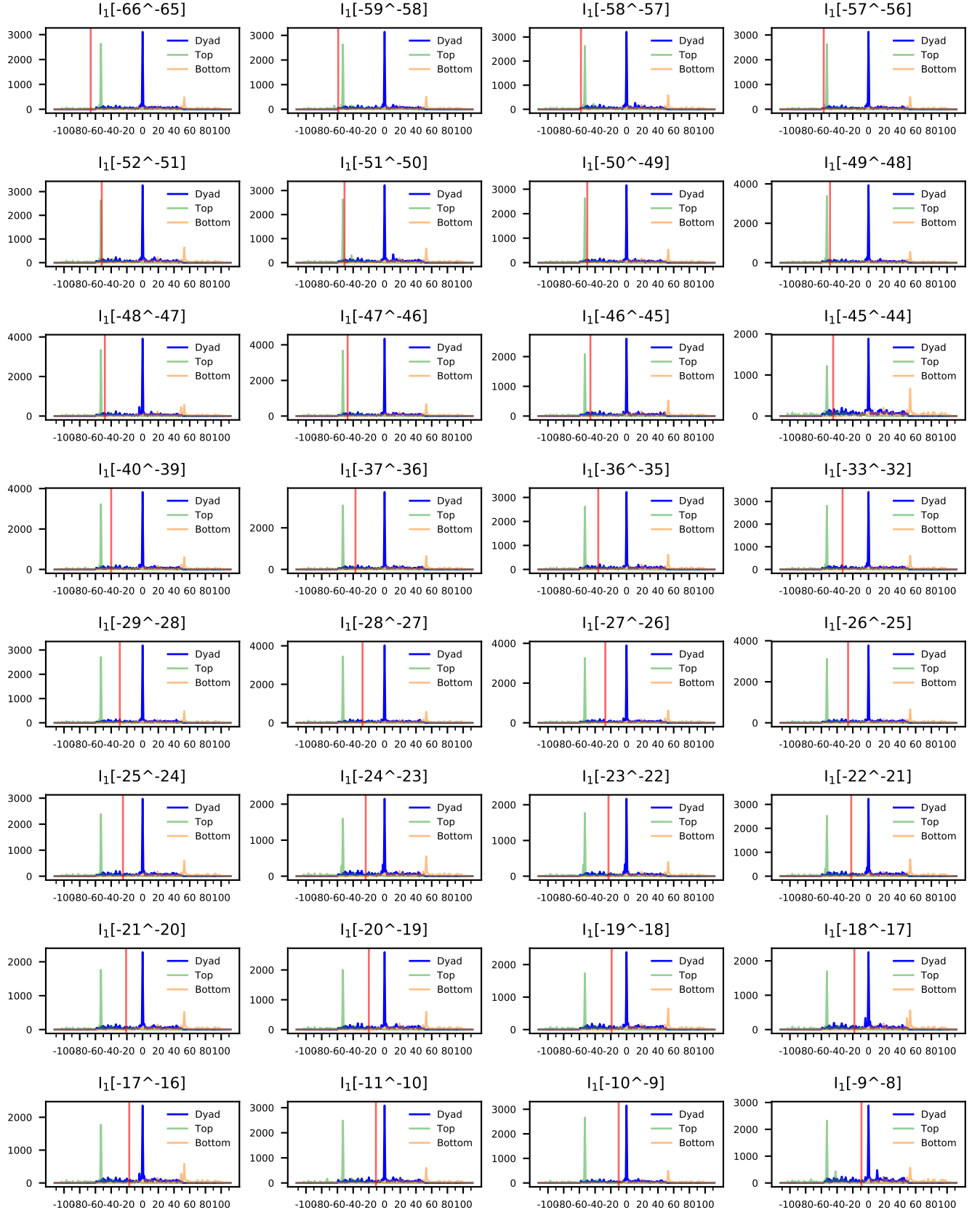

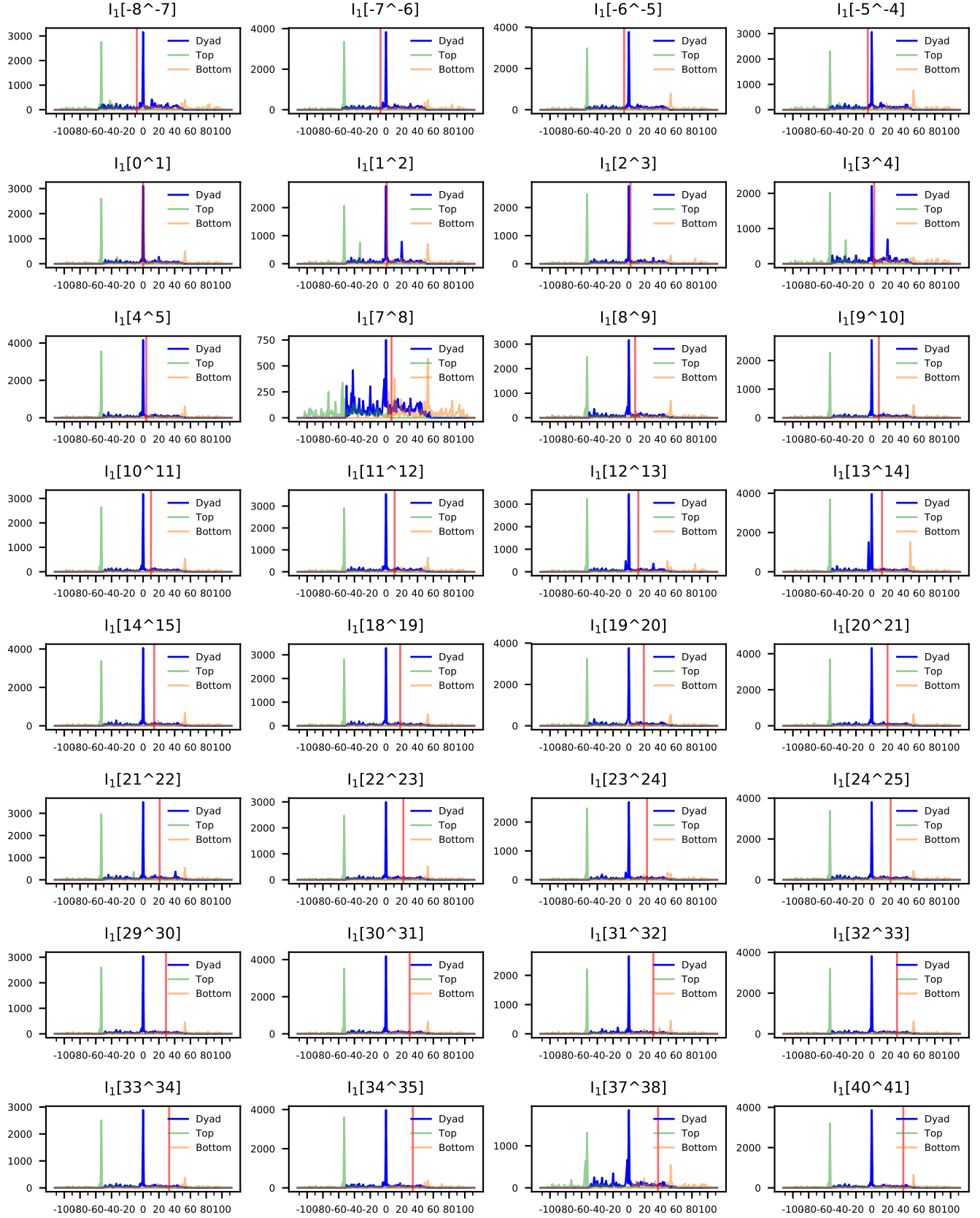

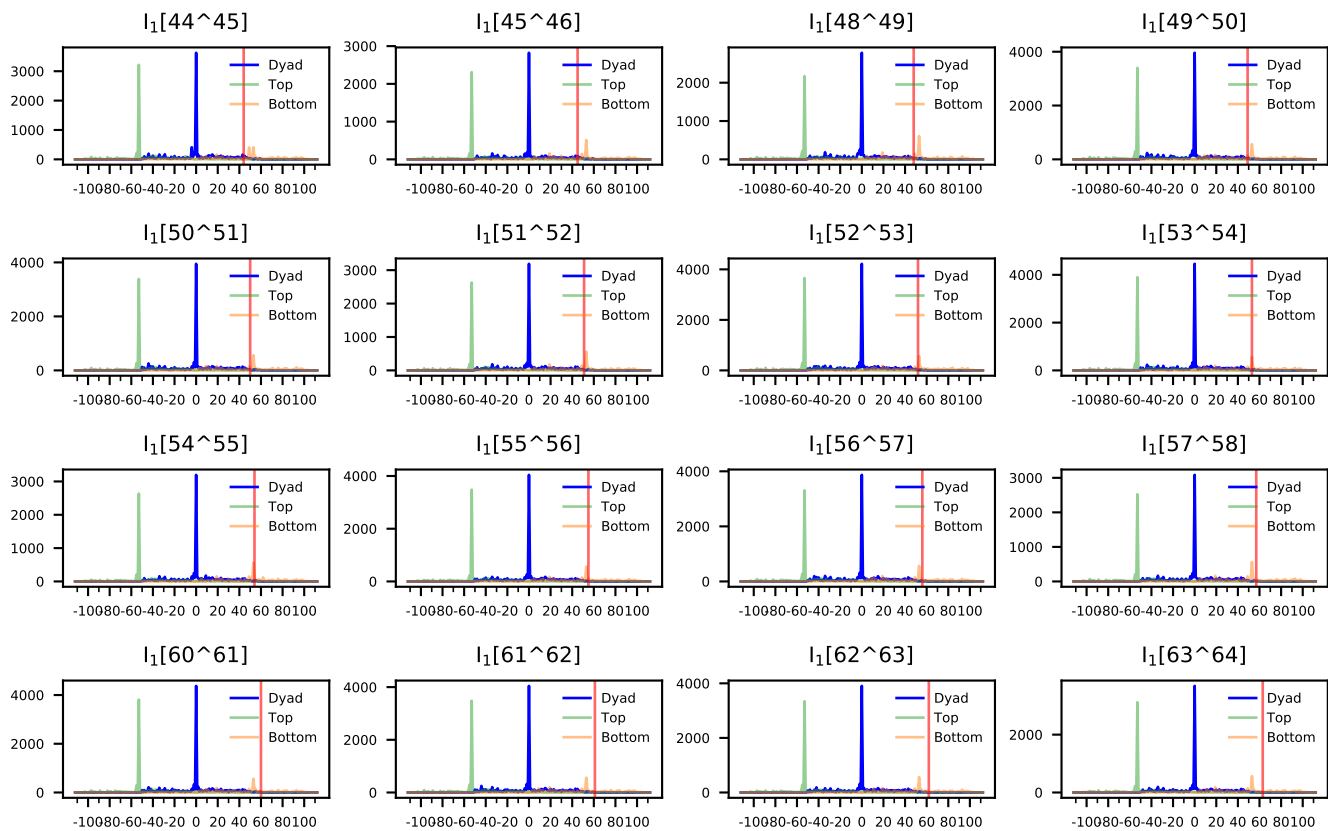

Supplement: gkad738_Supplemental_files [file gkad738_supplemental_files.zip › Supplementary Table 6 (601-Signals_I_before).pdf]

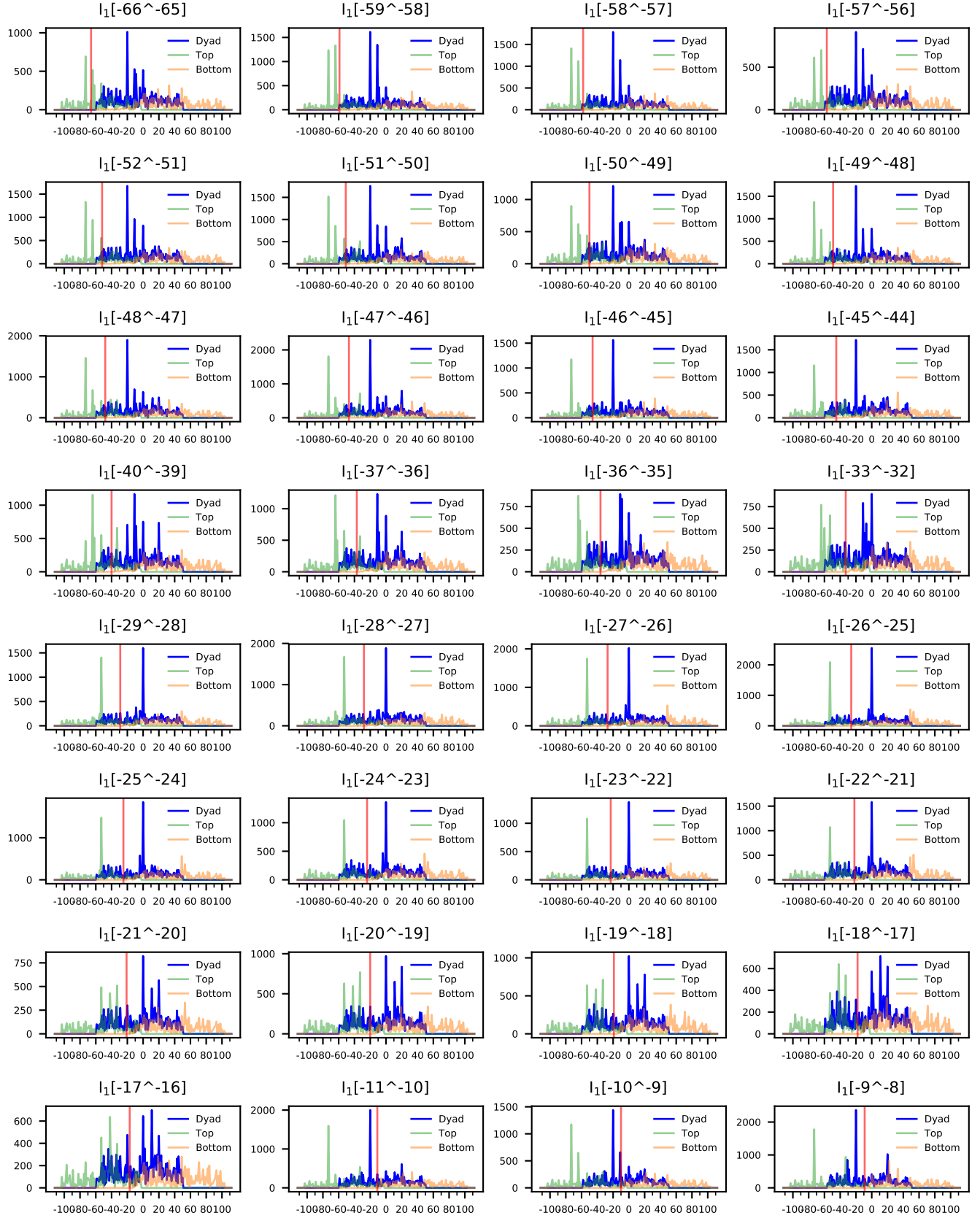

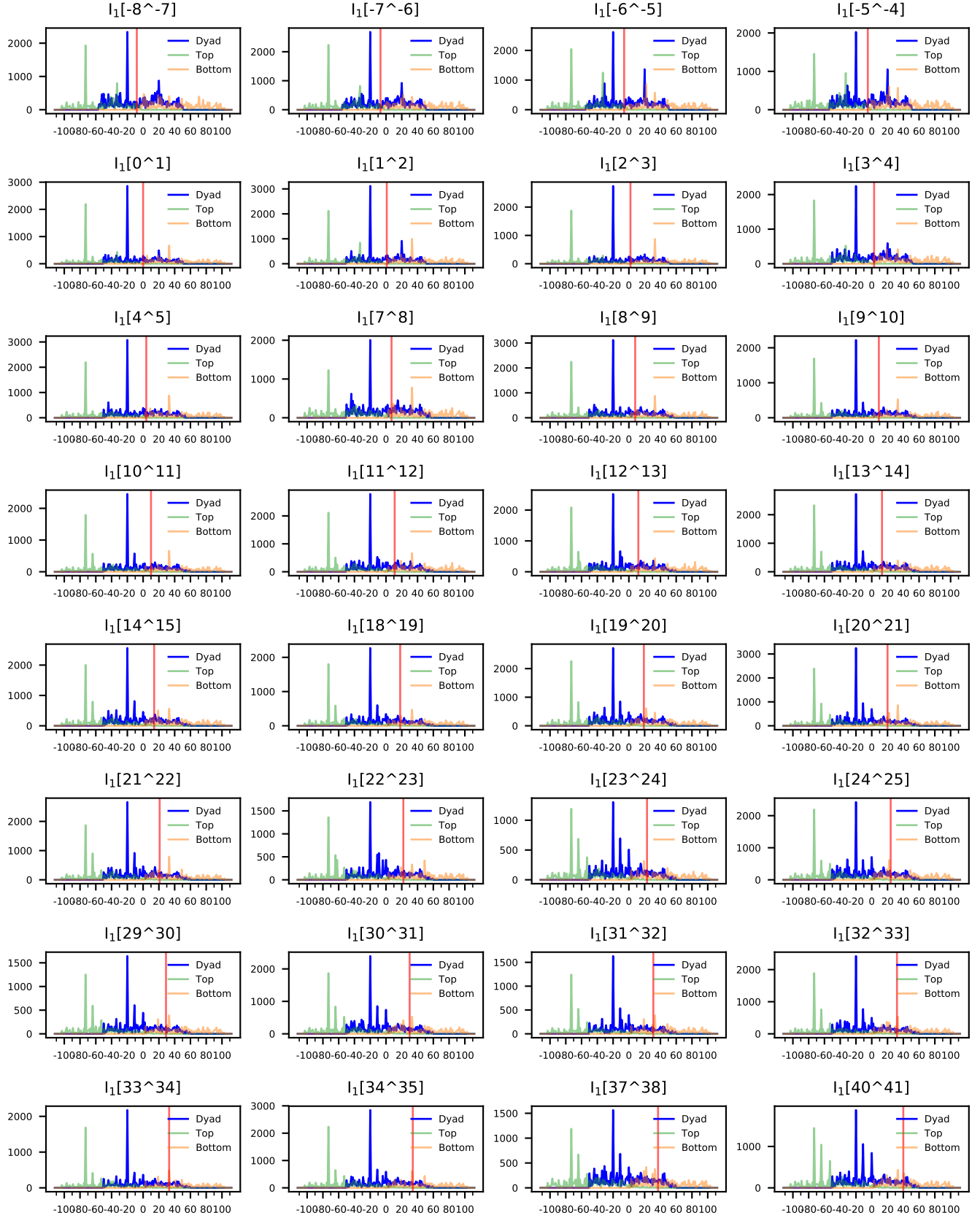

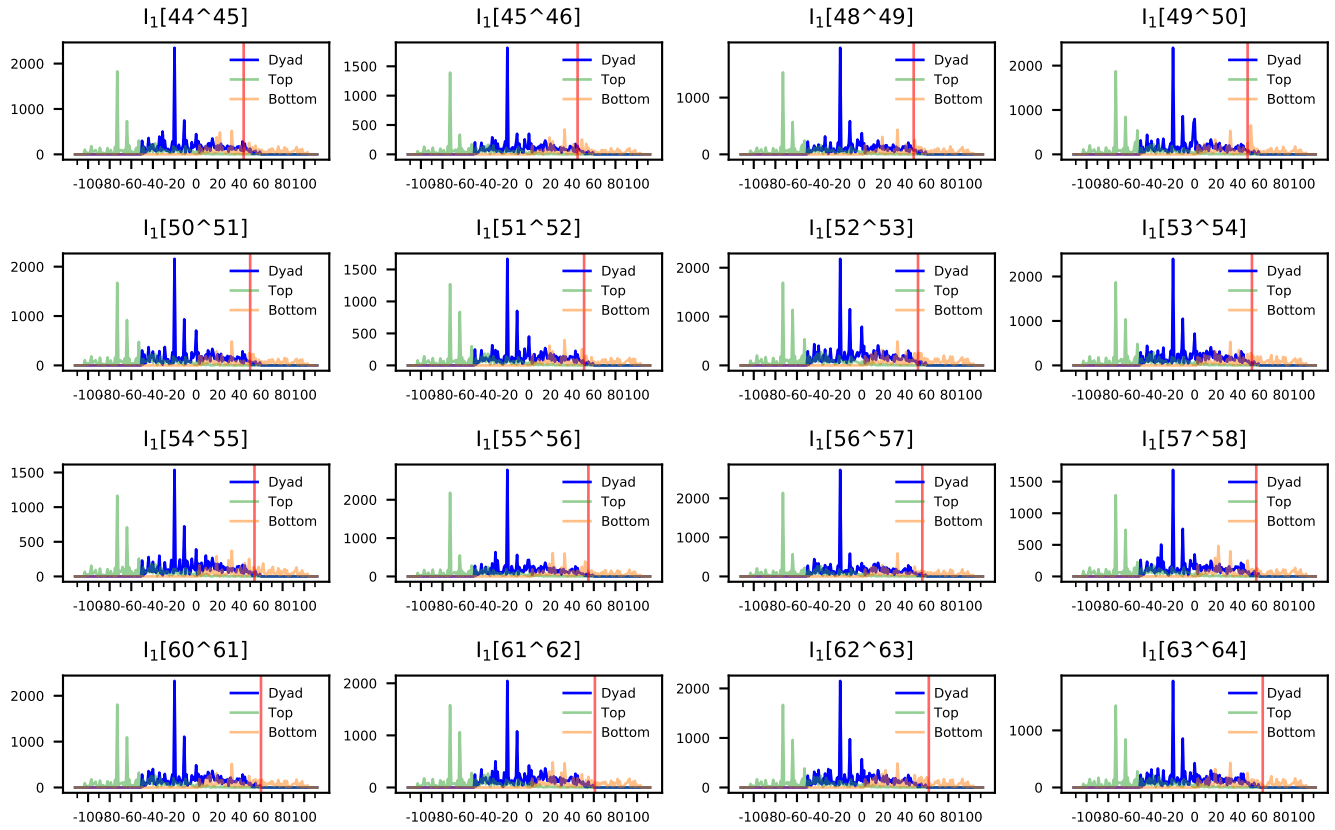

Supplement: gkad738_Supplemental_files [file gkad738_supplemental_files.zip › Supplementary Table 7 (601-Signals_I_after).pdf]

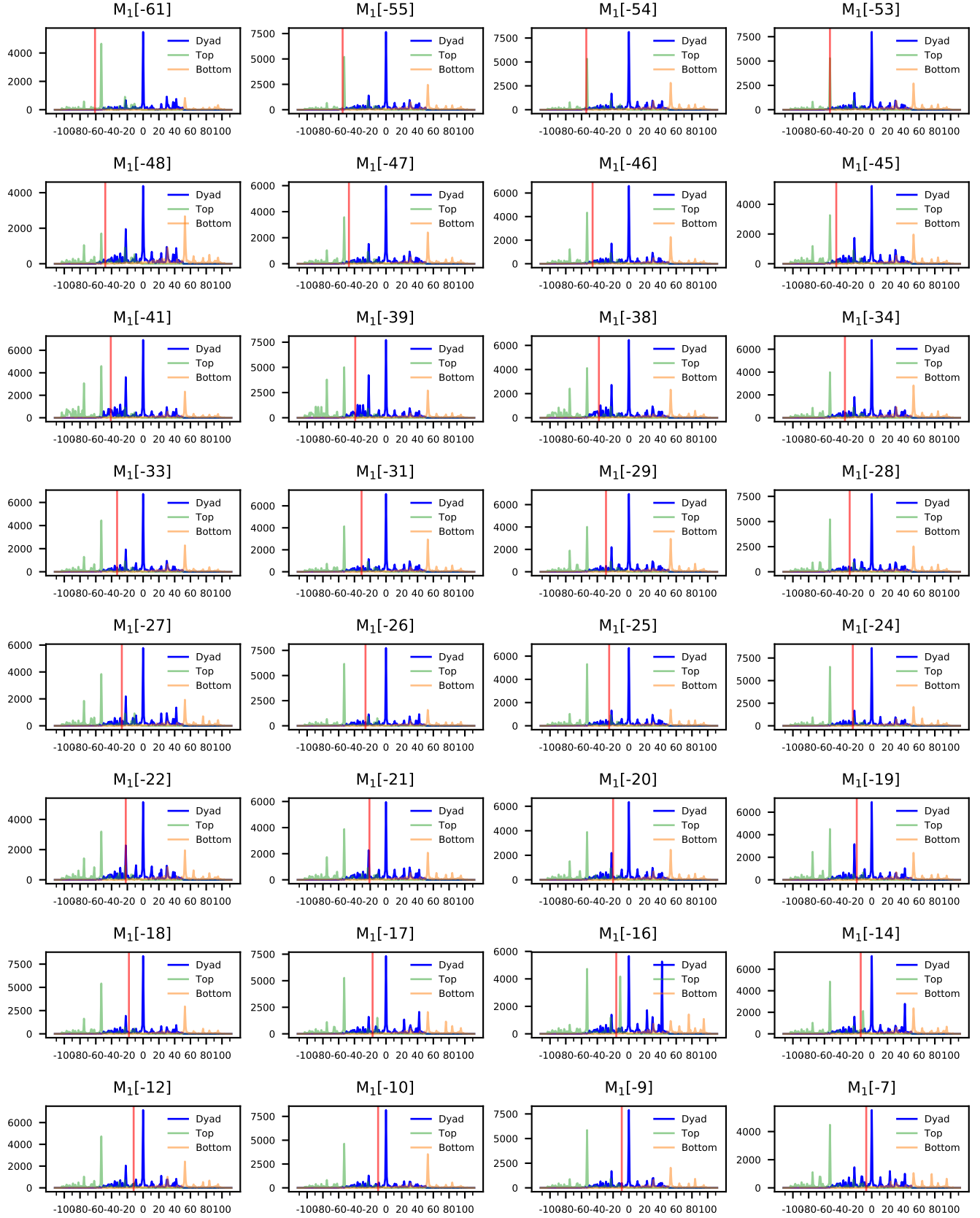

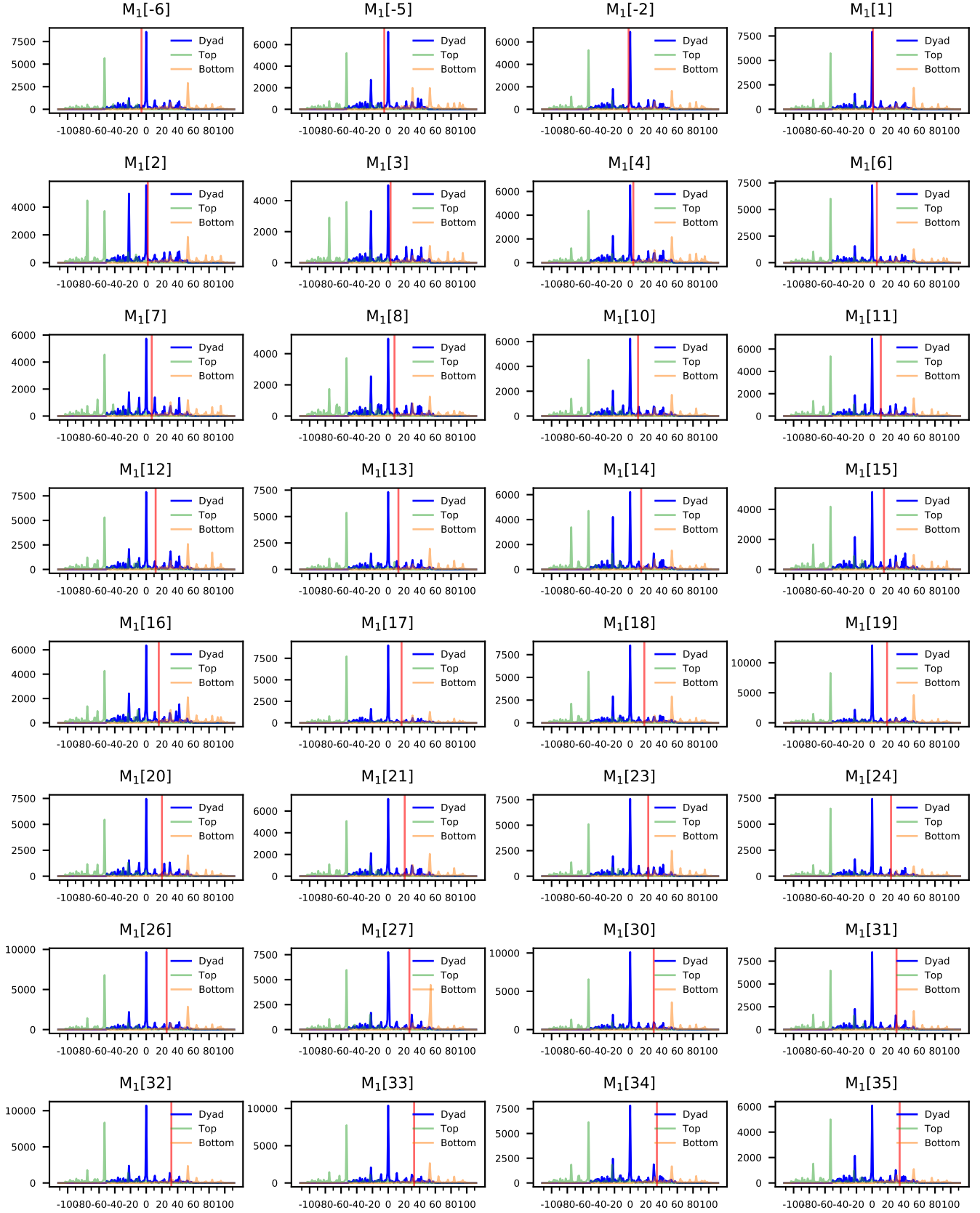

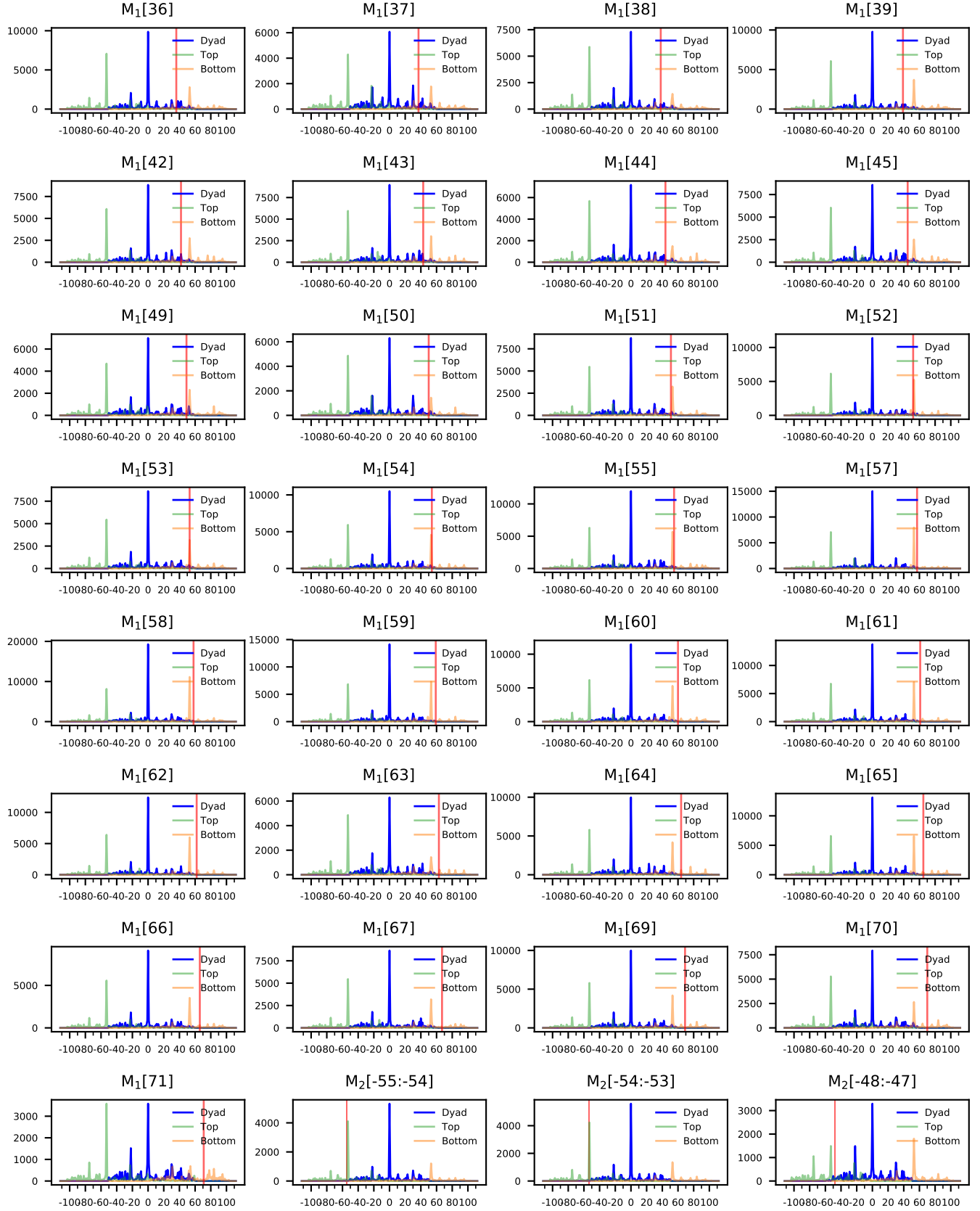

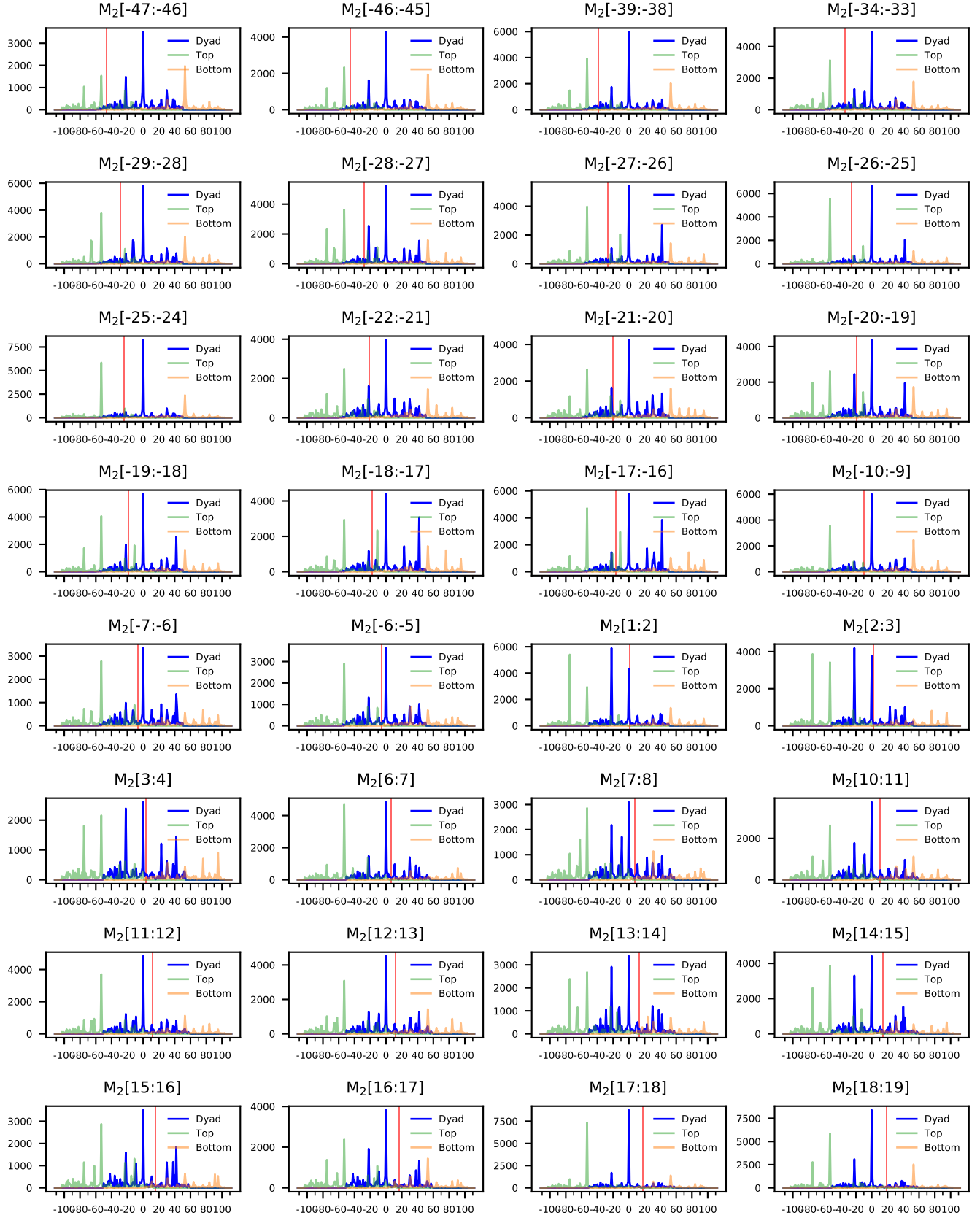

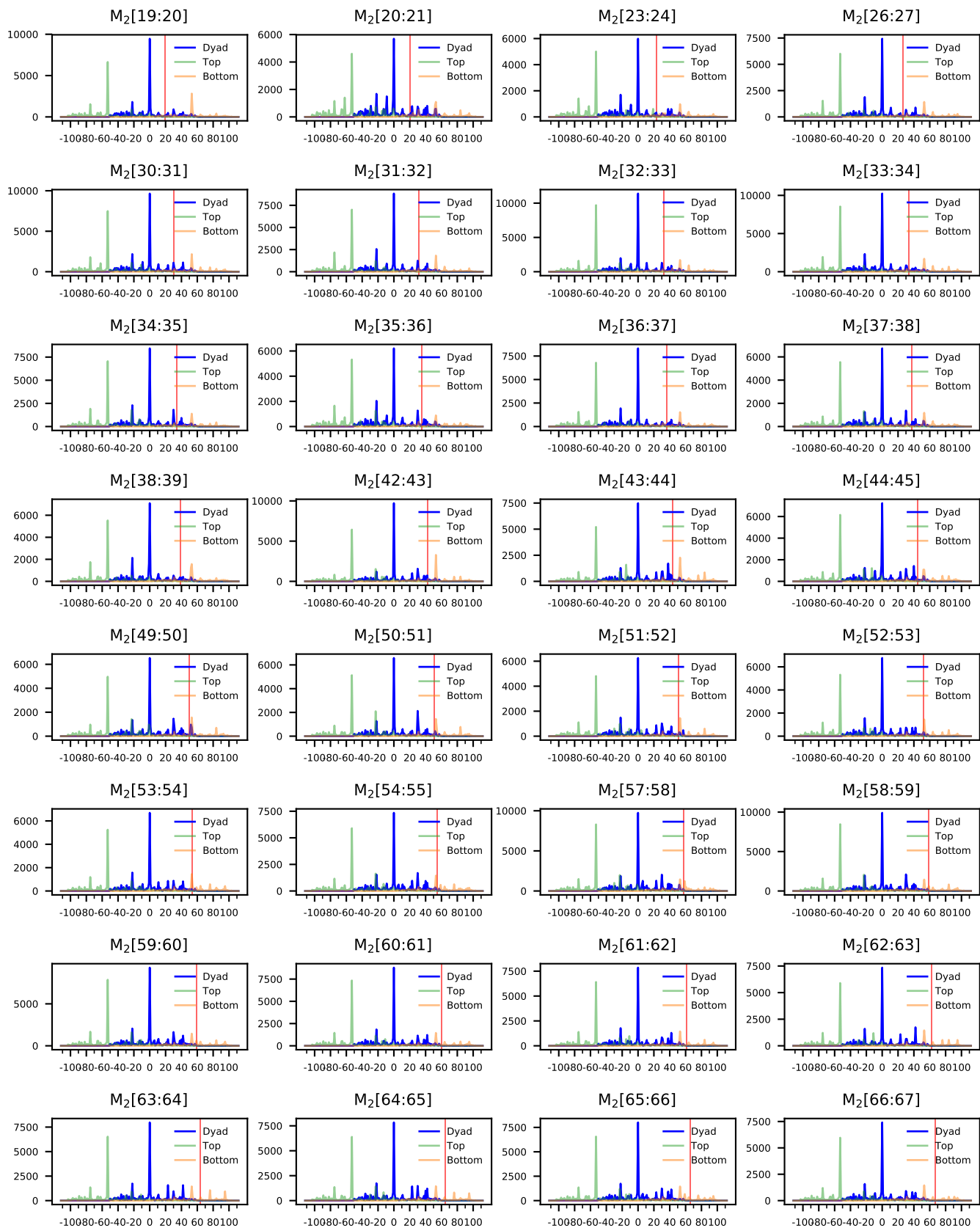

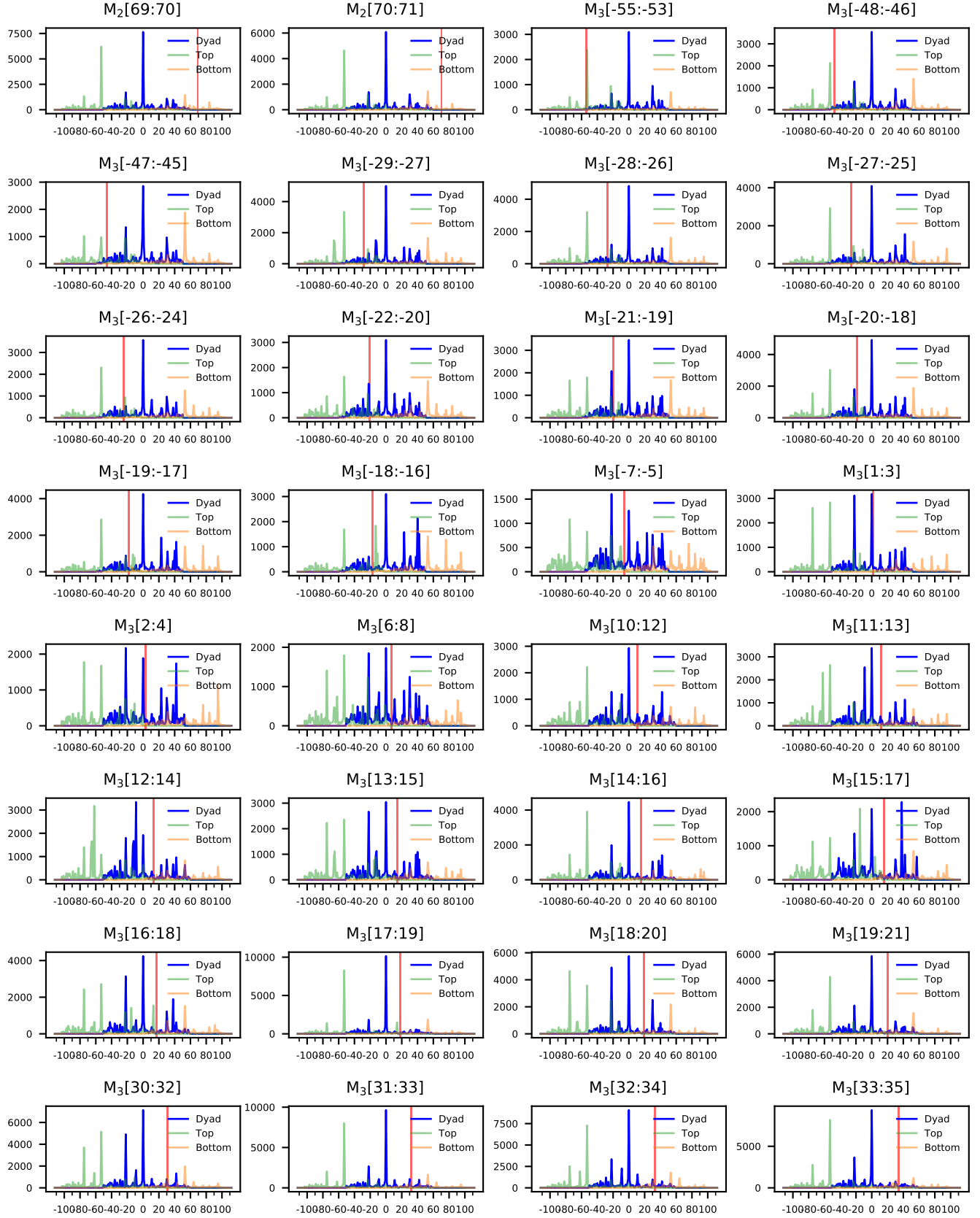

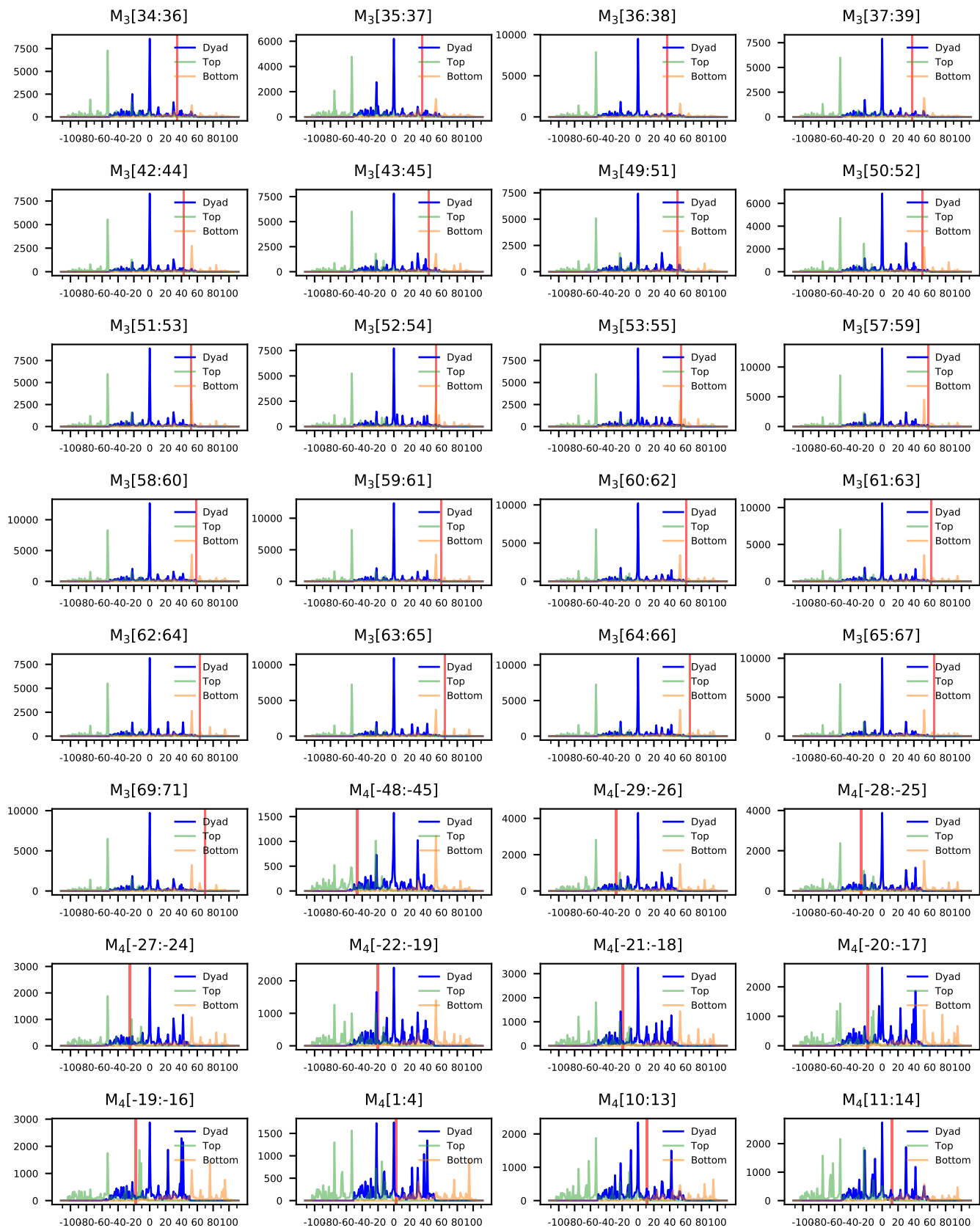

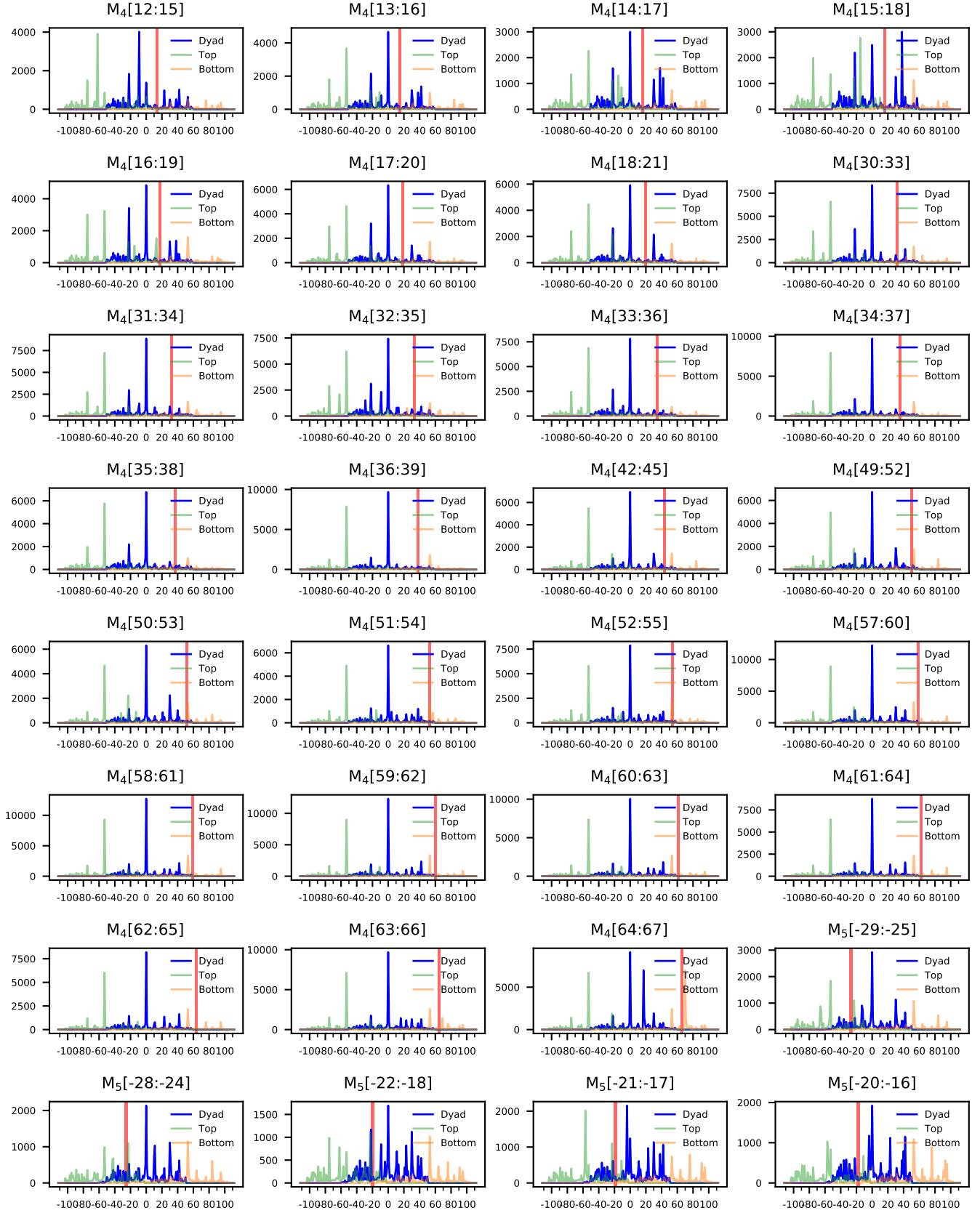

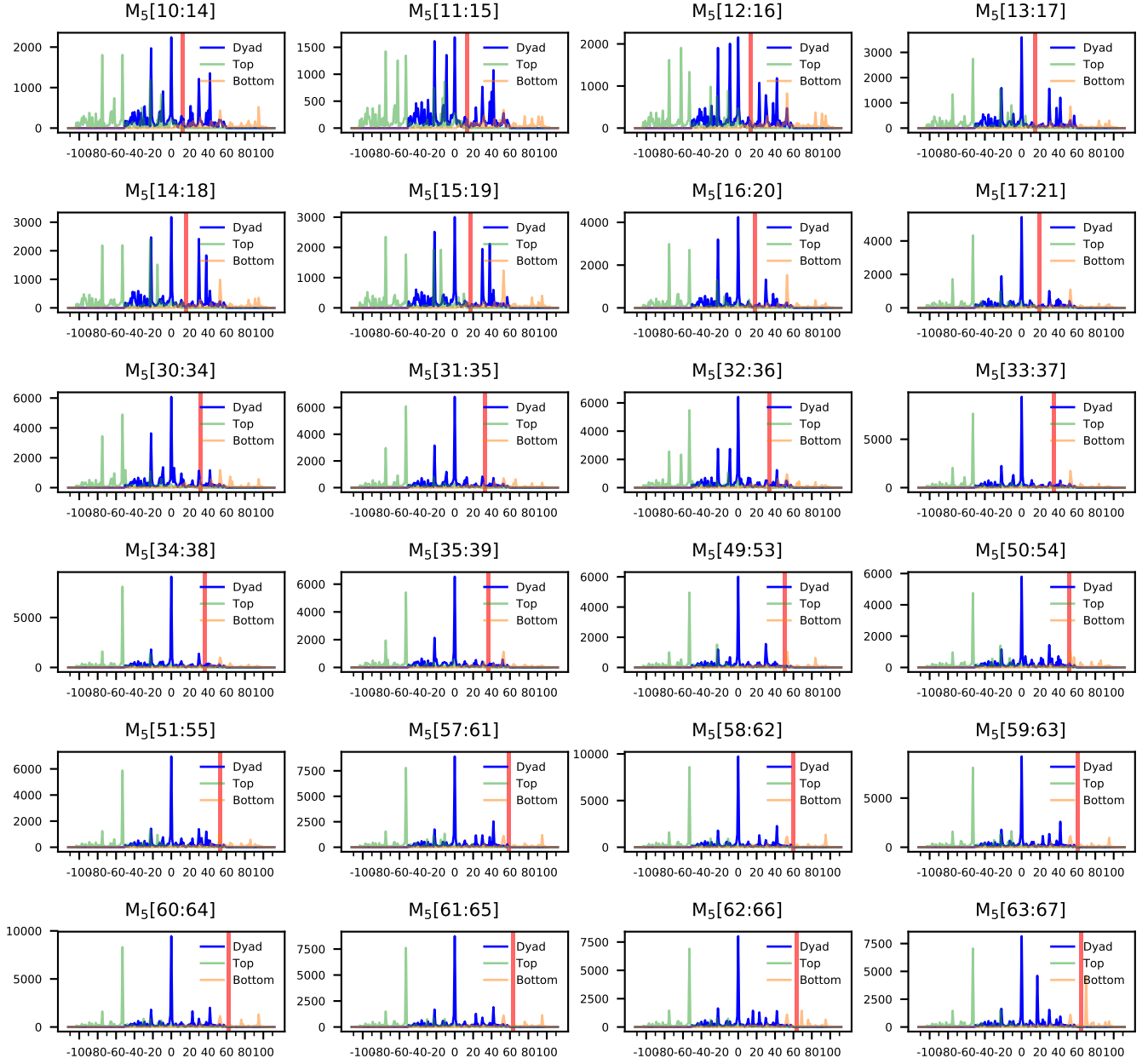

Supplement: gkad738_Supplemental_files [file gkad738_supplemental_files.zip › Supplementary Table 8 (SWH1-Signals_M_before).pdf]

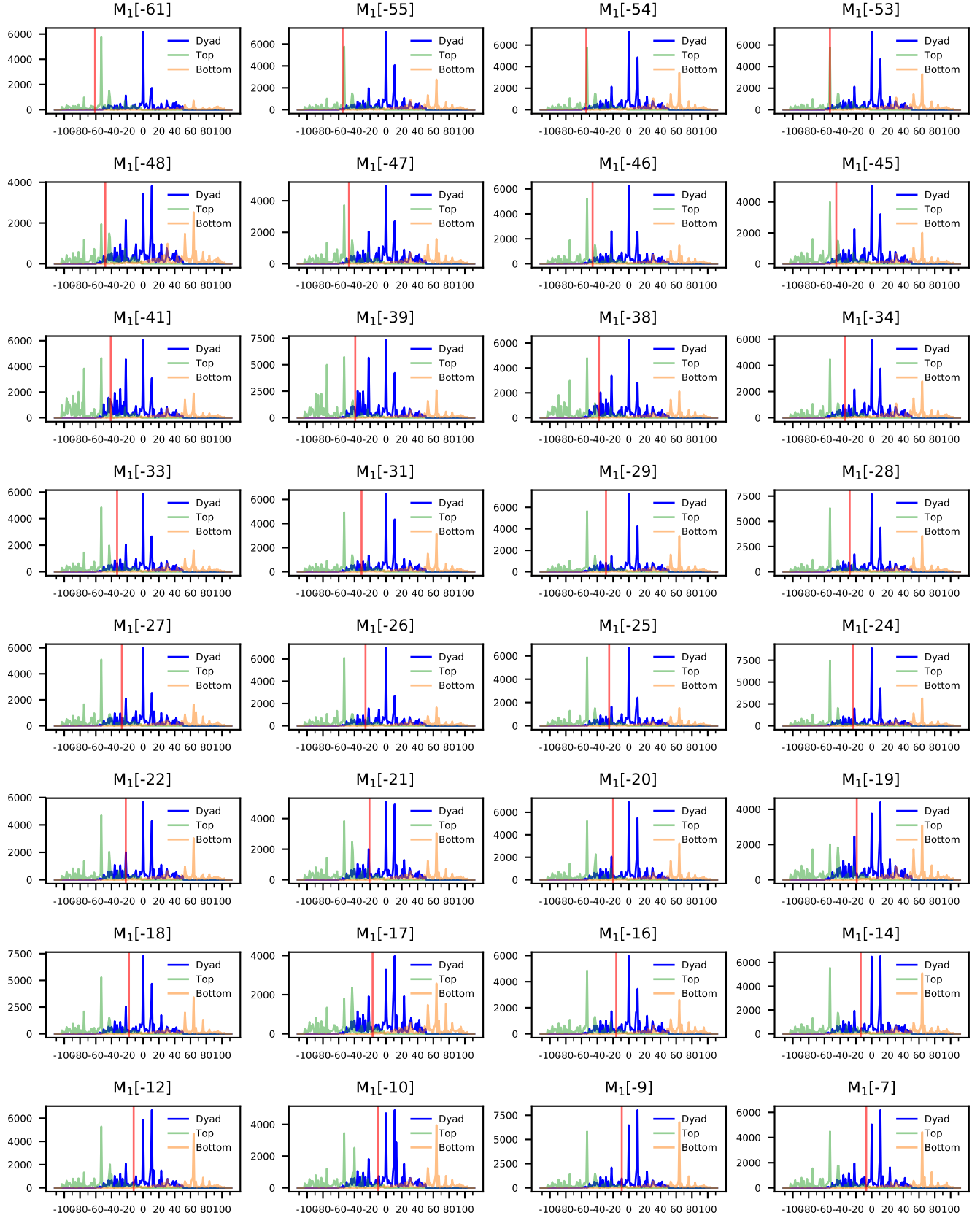

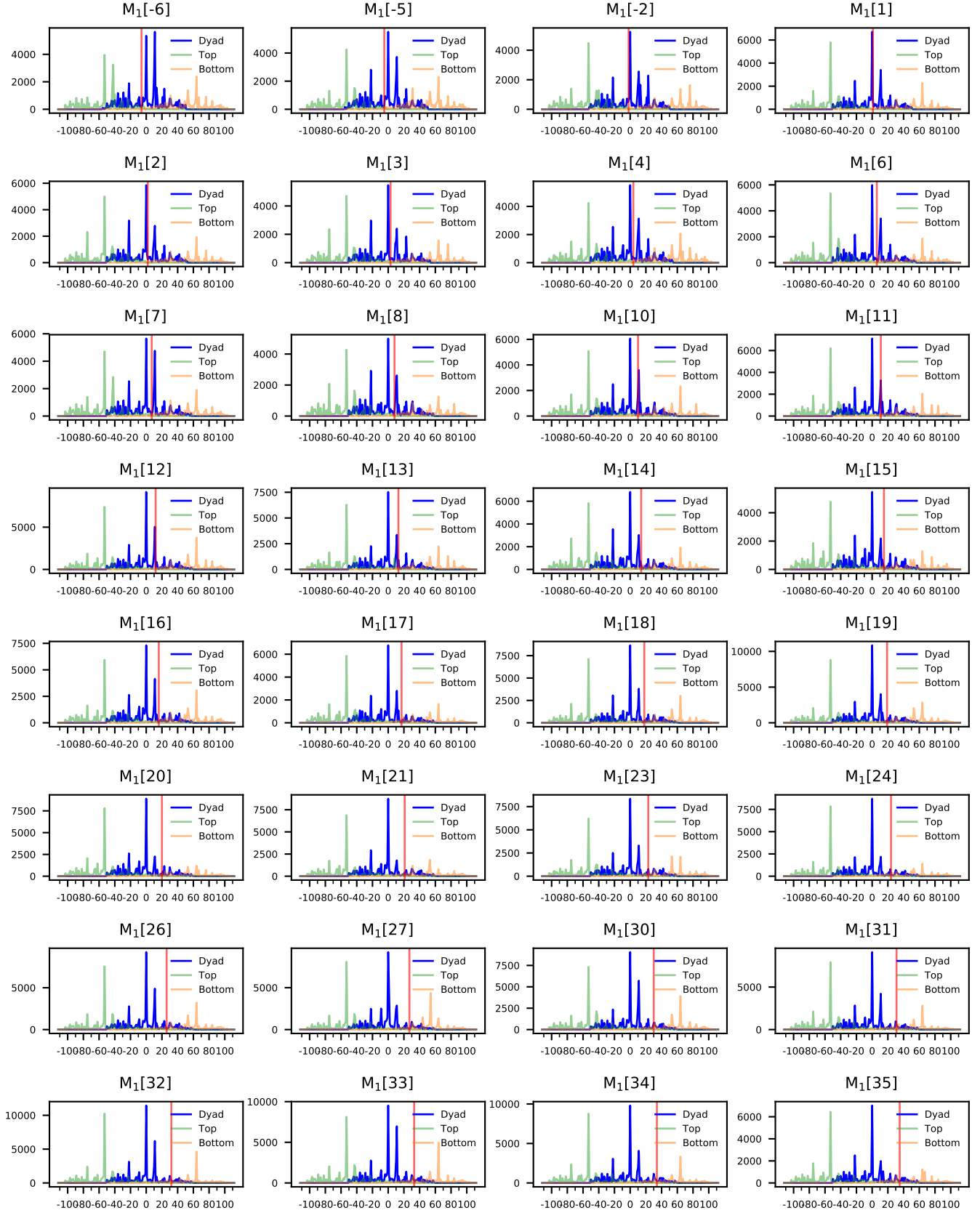

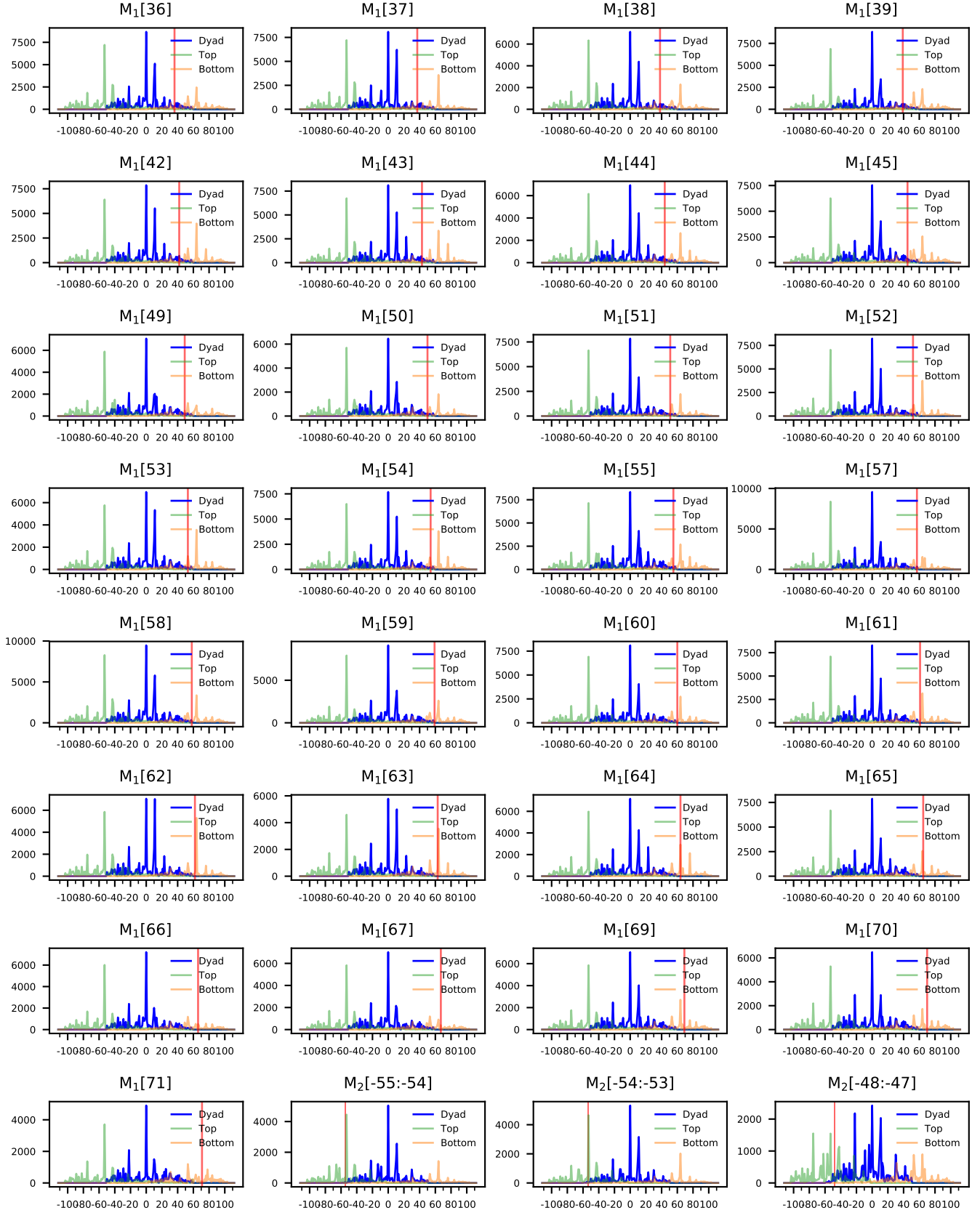

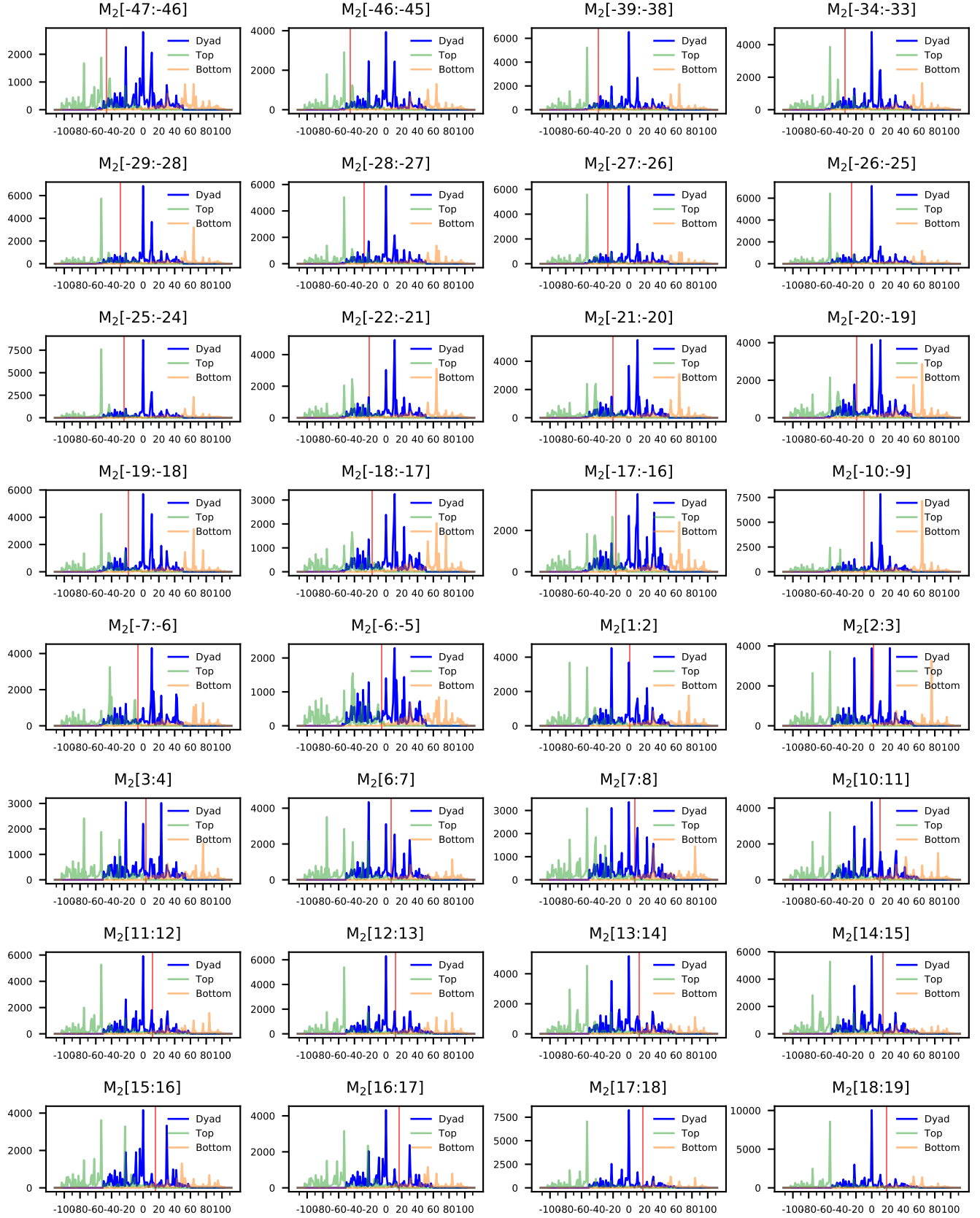

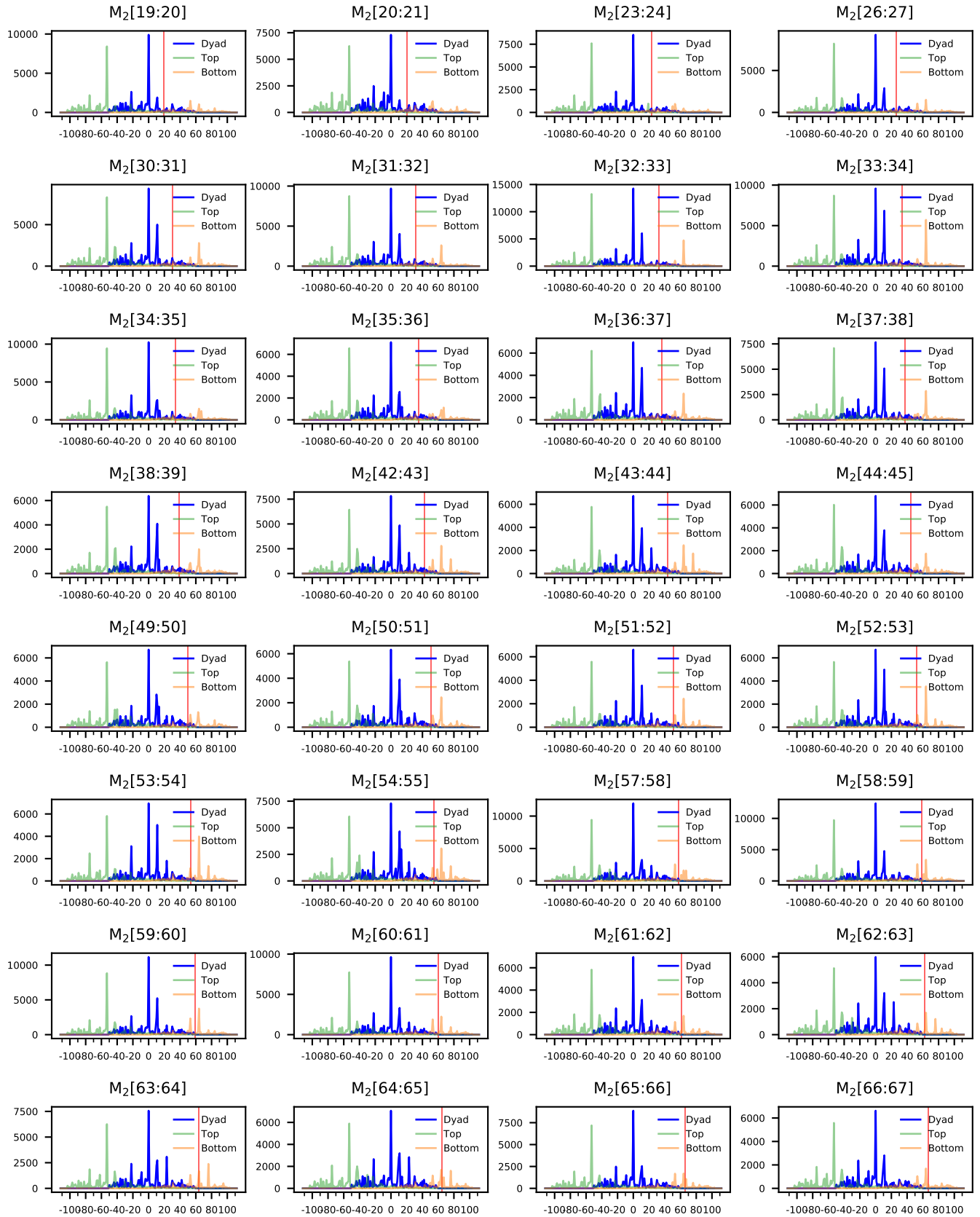

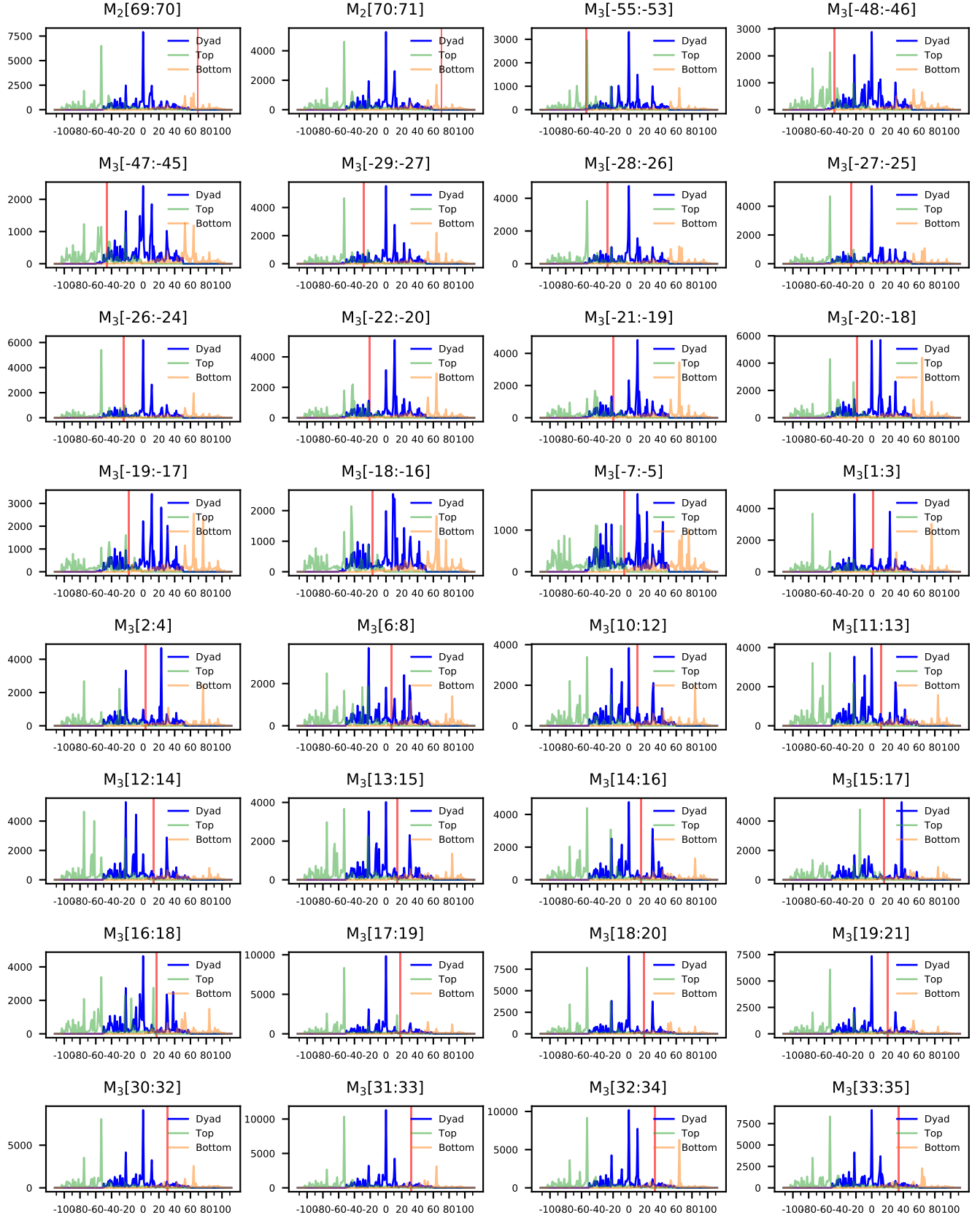

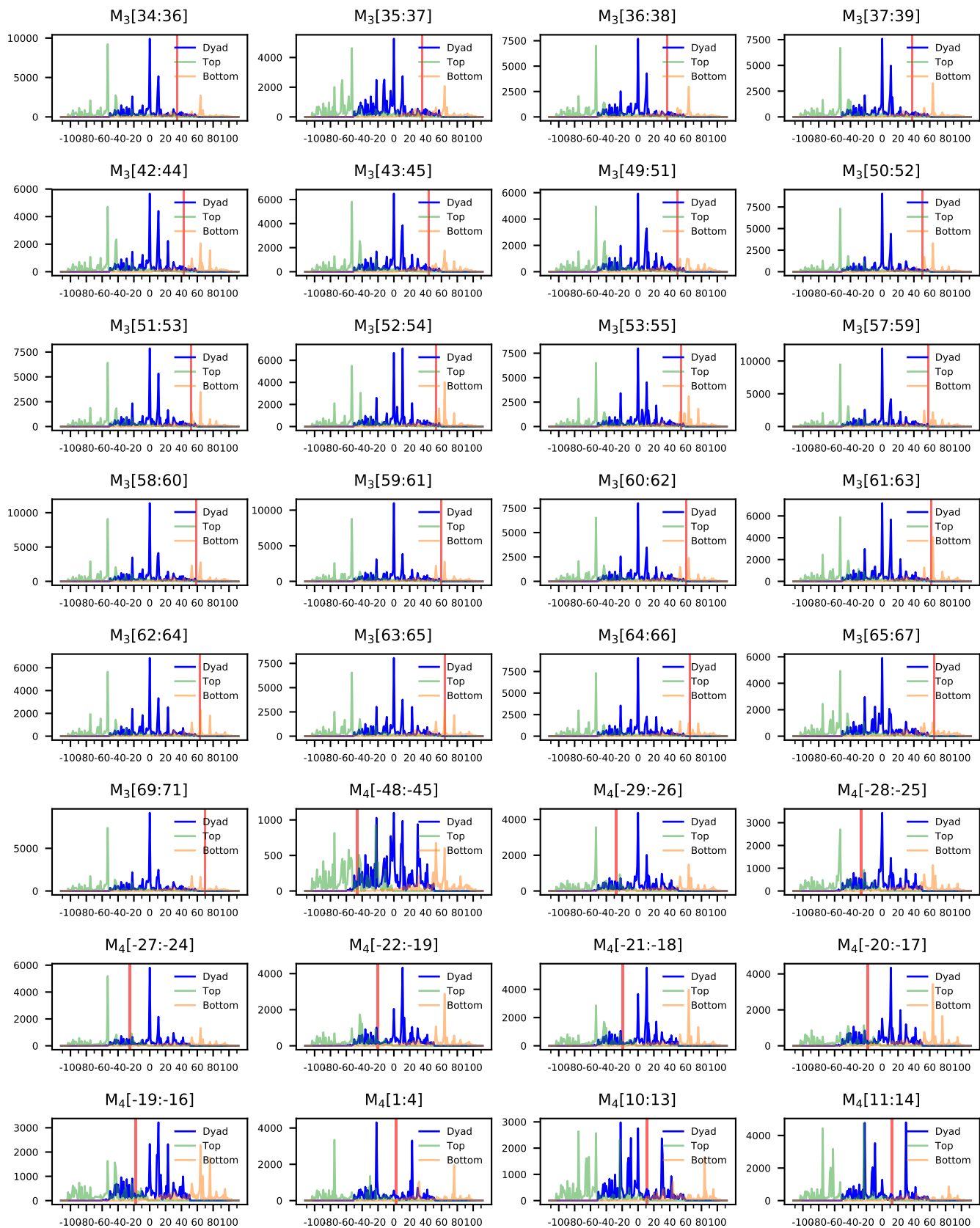

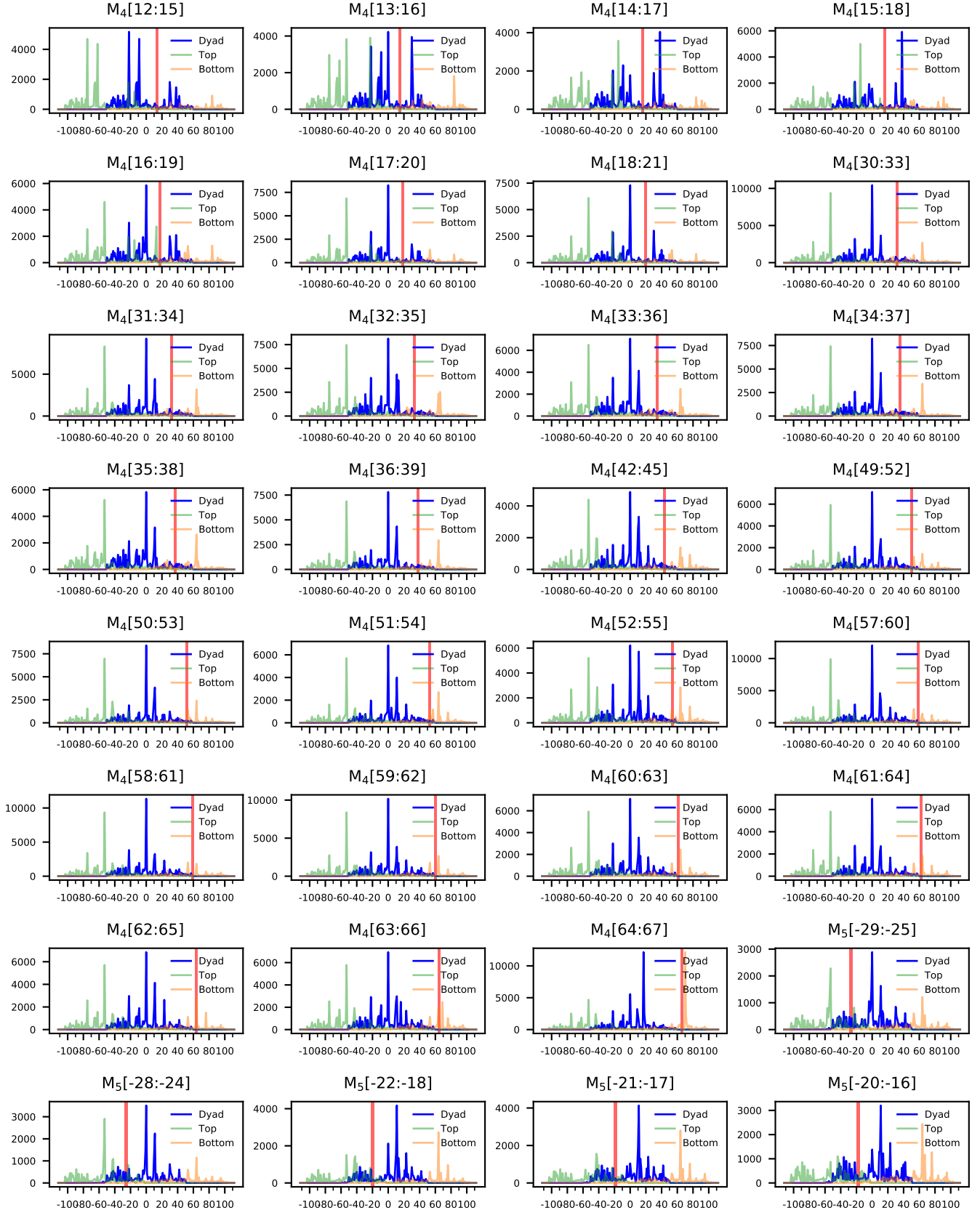

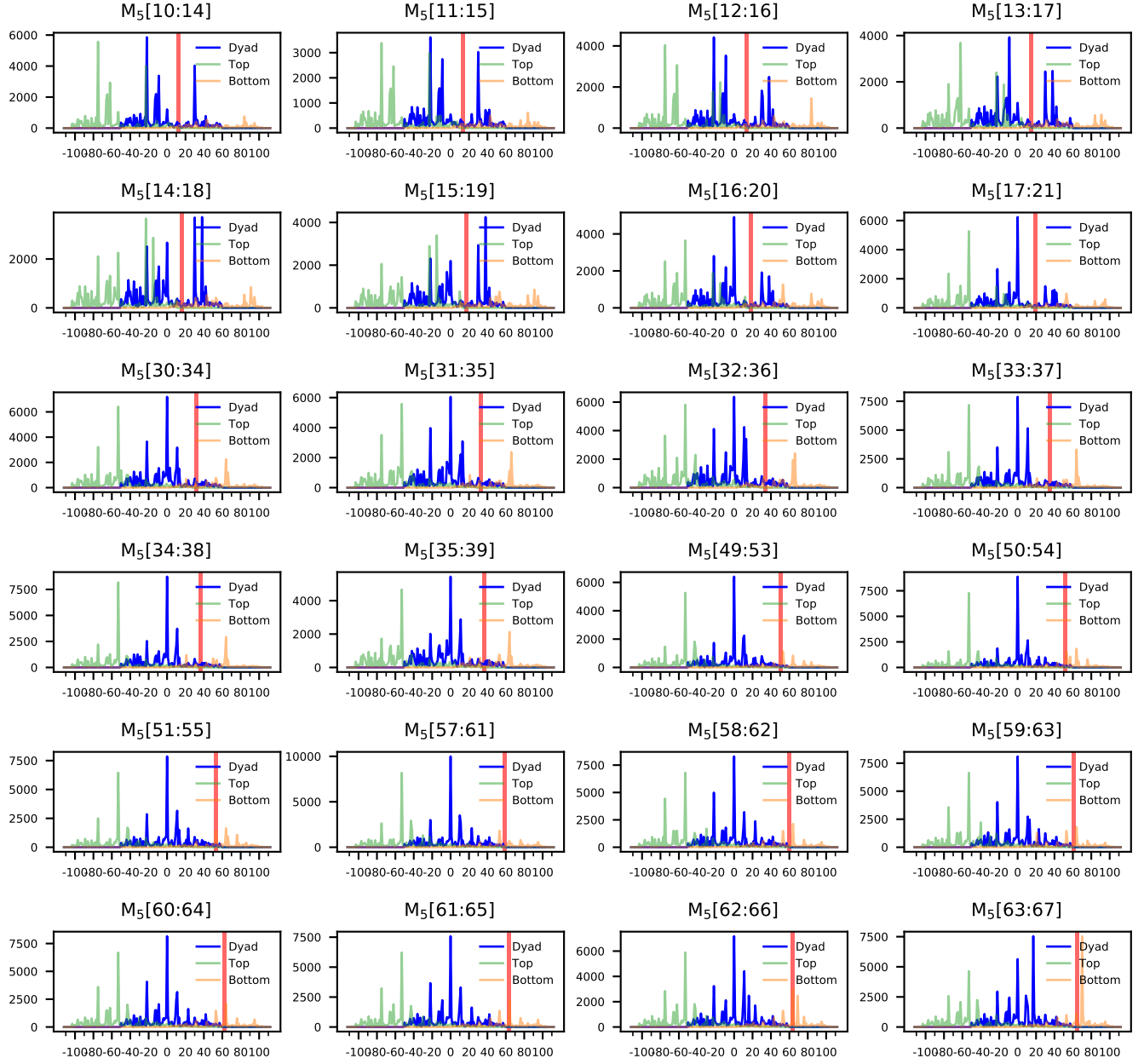

Supplement: gkad738_Supplemental_files [file gkad738_supplemental_files.zip › Supplementary Table 9 (SWH1-Signals_M_after).pdf]
